# Correction: Reconciliation and evolution of *Penicillium rubens* genome-scale metabolic networks–What about specialised metabolism?

**DOI:** 10.1371/journal.pone.0339632

**Published:** 2025-12-23

**Authors:** The PLOS One Staff

There are errors in the author affiliations. The correct affiliations are as follows:

Delphine Nègre^1,2^, Abdelhalim Larhlimi^2^, Samuel Bertrand^1^

1 Nantes Université, Institut des Substances et Organismes de la Mer, ISOMer, UR 2160, F-44000 Nantes, France, 2 Nantes Université, École Centrale Nantes, CNRS, LS2N, UMR 6004, F-44000 Nantes, France.

The publisher apologizes for the error.
