# Supplementary material for: Reconciliation and evolution of Penicillium rubens genome-scale metabolic networks–What about specialised metabolism?
Source: PLoS One. 2023 Aug 30;18(8):e0289757. doi: 10.1371/journal.pone.0289757 (PMC10468094; doi:10.1371/journal.pone.0289757)
Supplement: S7 File — This Livescript MATLAB showcases the characteristics of the reconstruction process and outlines the modifications required for generating the model. It provides a detailed analysis of the reconstruction and model development. (GZ) [file pone.0289757.s007.gz › Features_reconstruction_and_model.html]

Features of the iPrub22 reconstruction and model 

# Features of the iPrub22 reconstruction and model

Table of Contents

Introduction
0. Environment versions
1. Reconstruction
 1.1 Distribution format
 1.1.1 Format used consistency
 1.1.2 Recognised naming convention (▲)
 1.1.3 Reference information (▲)
 1.2. Metabolites
 1.2.1 Model compartmentation
 1.2.2 Human readable descriptive name (▲)
 1.2.3 Structure identifiers (▲)
 1.2.4 At least one database identifier from a reliable resource (▲)
 1.2.5 SBO terms (▲)
 1.3. Biochemical reactions
 1.3.1 Metadata (▲)
 1.3.2 At least one database identifier from a reliable resource
 1.3.3 Balance
 1.3.4 SBO terms (▲)
 1.3.5 Model preparation: objective function
 1.4. Genes
 1.4.1 Name (▲)
 1.4.2 Identifier (▲)
 1.4.3 SBO terms (▲)
 1.4.4 Gene products compartmentation
2. Model
 2.1 Reconstruction modifications: from reconstruction to model
 2.1.1 List of parameters
 2.1.2 Inconsistency during model loading
 2.1.3 Reversibility
 2.1.4 Reconciliation and Duplication
 2.1.5 Unbalanced reactions
 2.1.6 Other modifications
 2.2 Model Characteristics
 2.2.1 Numerical properties of a reconstruction
 2.2.2 Identify metabolic dead-ends
 2.2.3 Identify blocked reactions
 2.2.4 Find leakage or siphons in the heuristically internal part using the bounds given by the model
 2.2.5 Flux coupling analysis
 2.2.6 Cycle-free flux
 2.3 Flux simulation
 2.3.1 Default model
 2.3.2 Growth on different media

## Introduction

In the following document, we present the final features of the genome-scale metabolic network reconstruction of Penicillium rubens Wisconsin 54-1255. We also propose a model (i.e. one parameterisation proposition of the reconstruction) based on the similarity of a minimum culture medium to mimic the organism growth. The model is encoded in SBML format, and its detailed characteristics are outlined in the subsequent sections.

The design and implementation of these structures follow the community standards and recommendations set out in the "Community standards to facilitate development and address challenges in metabolic modeling" (https://doi.org/10.15252/msb.20199235). The purpose of this article is to standardise reconstruction practice by giving a "list as a guide to help standardize accessibility, content, and quality; however, more comprehensive documentation and more interpretable and accessible information can only improve the usability and biological relevance of the shared reconstruction." The recommendations suggested in this paper are followed by (▲).

## 0. Environment versions

To ensure the reproducibility of the data presented here, the versions of the tools used are:

- MATLAB version:

version()

ans = '9.5.0.1298439 (R2018b) Update 7'

- Cobra Toolbox version:

initCobraToolbox

\_\_\_\_\_ \_\_\_\_\_ \_\_\_\_\_ \_\_\_\_\_ \_\_\_\_\_ |
/ \_\_\_| / \_ \ | \_ \ | \_ \ / \_\_\_ \ | COnstraint-Based Reconstruction and Analysis
| | | | | | | |\_| | | |\_| | | |\_\_\_| | | The COBRA Toolbox - 2023
| | | | | | | \_ { | \_ / | \_\_\_ | |
| |\_\_\_ | |\_| | | |\_| | | | \ \ | | | | | Documentation:
\\_\_\_\_\_| \\_\_\_\_\_/ |\_\_\_\_\_/ |\_| \\_\ |\_| |\_| | http://opencobra.github.io/cobratoolbox
|
> Checking if git is installed ... Done (version: 2.17.1).
> Checking if the repository is tracked using git ... Done.
> Checking if curl is installed ... Done.
> Checking if remote can be reached ... (unsuccessful - no internet connection).
> Adding all the files of The COBRA Toolbox ... Done.
> Define CB map output... set to svg.
> TranslateSBML is installed and working properly.
> Configuring solver environment variables ...
- [-\*--] ILOG\_CPLEX\_PATH: /opt/ibm/ILOG/CPLEX\_Studio\_Community201/cplex/bin/x86-64\_linux
- [\*---] GUROBI\_PATH: /opt/gurobi903/linux64/matlab
- [----] TOMLAB\_PATH: --> set this path manually after installing the solver ( see instructions )
- [\*---] MOSEK\_PATH: /opt/mosek/9.2/
Done.
> Checking available solvers and solver interfaces ...gurobi
ibm\_cplex
tomlab\_cplex
glpk
mosek
rcode: 1001
rmsg: 'The license has expired.'
rcodestr: 'MSK\_RES\_ERR\_LICENSE\_EXPIRED'
matlab
pdco
quadMinos
dqqMinos
cplex\_direct
cplexlp
qpng
tomlab\_snopt
lp\_solve
Done.
> Setting default solvers ... Done.
> Saving the MATLAB path ... Done.
- The MATLAB path was saved as ~/pathdef.m.
> Summary of available solvers and solver interfaces
Support LP MILP QP MIQP NLP
----------------------------------------------------------------------
gurobi active 1 1 1 1 -
ibm\_cplex active 0 0 0 0 -
tomlab\_cplex active 0 0 0 0 -
glpk active 1 1 - - -
mosek active 1 - 1 - -
matlab active 0 - - - 0
pdco active 1 - 1 - -
quadMinos active 1 - - - -
dqqMinos active 1 - 1 - -
cplex\_direct active 0 0 0 - -
cplexlp active 0 - - - -
qpng passive - - 1 - -
tomlab\_snopt passive - - - - 0
lp\_solve legacy 1 - - - -
----------------------------------------------------------------------
Total - 7 2 5 1 0
+ Legend: - = not applicable, 0 = solver not compatible or not installed, 1 = solver installed.
> You can solve LP problems using: 'gurobi' - 'glpk' - 'mosek'
> You can solve MILP problems using: 'gurobi' - 'glpk'
> You can solve QP problems using: 'gurobi' - 'mosek'
> You can solve MIQP problems using: 'gurobi'
> You can solve NLP problems using:

- Gurobi version:

getCobraSolverVersion('gurobi') ;

> The version of GUROBI is 903.

## 1. Reconstruction

### 1.1 Distribution format

iPrub22.sbml (available here) is produced by the reconstruction process described in the Supplementary Material. It is available via BioModelsunder the identifier MODEL2306150001.

#### 1.1.1 Format used consistency

SBML (Systems Biology Markup Language) is the standard file format chosen for storing and sharing our reconstruction. First of all, the format used for the model distribution is checked for consistency with validateSBML.py (one of the example programs demonstrating the use of different libSBML API calls available on https://synonym.caltech.edu/software/libsbml/libsbml-docs/examples/). More information is contained in LibSBML: an API Library for SBML (https://doi.org/10.1093/bioinformatics/btn051)

The output below shows a single warning occurring 86 times:

- (99701 [Warning]) The SBOTerm used is not recognised by libSBML. Therefore, the appropriate parentage can not be checked. However, since libSBML is referring to a snapshot of the SBO tree, the term may now exist. Unknown SBO term 'SBO:0000672'. (detailed explanation here)

Once the metabolic model's internal structure is correct, it is loaded into MATLAB:

tic

iPrub22\_reconstruction = readCbModel('./Network/iPrub22.sbml')

iPrub22\_reconstruction = struct with fields:

S: [5464×5919 double]
mets: {5464×1 cell}
b: [5464×1 double]
csense: [5464×1 char]
rxns: {5919×1 cell}
lb: [5919×1 double]
ub: [5919×1 double]
c: [5919×1 double]
osenseStr: 'max'
genes: {6171×1 cell}
rules: {5919×1 cell}
geneNames: {6171×1 cell}
compNames: {2×1 cell}
comps: {2×1 cell}
proteins: {6171×1 cell}
metCharges: [5464×1 double]
metFormulas: {5464×1 cell}
metNames: {5464×1 cell}
metNotes: {5464×1 cell}
metHMDBID: {5464×1 cell}
metInChIString: {5464×1 cell}
metKEGGID: {5464×1 cell}
metChEBIID: {5464×1 cell}
metPubChemID: {5464×1 cell}
metMetaNetXID: {5464×1 cell}
metSEEDID: {5464×1 cell}
metBiGGID: {5464×1 cell}
metBioCycID: {5464×1 cell}
metLIPIDMAPSID: {5464×1 cell}
metReactomeID: {5464×1 cell}
metSABIORKID: {5464×1 cell}
metSLMID: {5464×1 cell}
metSBOTerms: {5464×1 cell}
geneEntrezID: {6171×1 cell}
geneRefSeqID: {6171×1 cell}
geneKEGGID: {6171×1 cell}
rxnNames: {5919×1 cell}
rxnNotes: {5919×1 cell}
rxnECNumbers: {5919×1 cell}
rxnKEGGID: {5919×1 cell}
rxnMetaNetXID: {5919×1 cell}
rxnBRENDAID: {5919×1 cell}
rxnBioCycID: {5919×1 cell}
rxnReactomeID: {5919×1 cell}
rxnRheaID: {5919×1 cell}
rxnBiGGID: {5919×1 cell}
rxnSBOTerms: {5919×1 cell}
subSystems: {5919×1 cell}
description: 'iPrub22.sbml'
modelVersion: [1×1 struct]
modelName: 'iPrub22'
modelID: 'iPrub22\_v1'
compisgoID: {2×1 cell}
compismetanetx\_\_46\_\_compartmentID: {2×1 cell}
metiscasID: {5464×1 cell}
metischemspiderID: {5464×1 cell}
metisdrugbankID: {5464×1 cell}
metisinchikeyID: {5464×1 cell}
metiskegg\_\_46\_\_drugID: {5464×1 cell}
metiskegg\_\_46\_\_glycanID: {5464×1 cell}
metisknapsackID: {5464×1 cell}
metismetabolightsID: {5464×1 cell}
metisumbbd\_\_46\_\_compoundID: {5464×1 cell}
modelAnnotation: '<annotation>↵ <rdf:RDF xmlns:rdf="http://www.w3.org/1999/02/22-rdf-syntax-ns#" xmlns:dcterms="http://purl.org/dc/terms/" xmlns:vCard="http://www.w3.org/2001/vcard-rdf/3.0#" xmlns:vCard4="http://www.w3.org/2006/vcard/ns#" xmlns:bqbiol="http://biomodels.net/biology-qualifiers/" xmlns:bqmodel="http://biomodels.net/model-qualifiers/">↵ <rdf:Description rdf:about="#meta\_iPrub22\_v1">↵ <bqbiol:hasTaxon>↵ <rdf:Bag>↵ <rdf:li rdf:resource="https://identifiers.org/taxonomy/500485"/>↵ </rdf:Bag>↵ </bqbiol:hasTaxon>↵ <bqbiol:is>↵ <rdf:Bag>↵ <rdf:li rdf:resource="https://identifiers.org/insdc.gca/GCA\_000226395.1"/>↵ </rdf:Bag>↵ </bqbiol:is>↵ </rdf:Description>↵ </rdf:RDF>↵</annotation>'
proteiniskegg\_\_46\_\_genesID: {6171×1 cell}
proteinisncbigeneID: {6171×1 cell}
proteinisncbiproteinID: {6171×1 cell}
proteinisrefseqID: {6171×1 cell}
proteinisuniprotID: {6171×1 cell}
rxnisseed\_\_46\_\_reactionID: {5919×1 cell}
rxnisuniprotID: {5919×1 cell}

%sparse format accelerates computations with large networks

iPrub22\_reconstruction.S = sparse(iPrub22\_reconstruction.S);

toc

Elapsed time is 172.196199 seconds.

#### 1.1.2 Recognised naming convention (▲)

The existing model iPrub22 follows the recommended practice for model identifiers described in "Community standards to facilitate development and address challenges in metabolic modeling" (https://doi.org/10.15252/msb.20199235)

%ModelID is available here:

disp(iPrub22\_reconstruction.modelID)

iPrub22\_v1

NB: iPrub22v1 = in silico Penicillium rubens reconstruction published in 2022, version 1

% and software versioning is documented here:

disp(iPrub22\_reconstruction.modelVersion)

SBML\_level: 3
SBML\_version: 1
fbc\_version: 2

#### 1.1.3 Reference information (▲)

As some model descriptors are not yet supported as model features by Cobra Toolbox, reference information is stored in the annotation section when it is possible to embed them via the MIRIAM annotation otherwise in the note section.

- Machine-readable reference to organisms and species embedded via MIRIAM annotation
- NCBI reference genome
- DOI
- Author(s) contact information

%Machine-readable reference to organism and species embedded via MIRIAM annotation

disp(iPrub22\_reconstruction.modelAnnotation)

<annotation>
<rdf:RDF xmlns:rdf="http://www.w3.org/1999/02/22-rdf-syntax-ns#" xmlns:dcterms="http://purl.org/dc/terms/" xmlns:vCard="http://www.w3.org/2001/vcard-rdf/3.0#" xmlns:vCard4="http://www.w3.org/2006/vcard/ns#" xmlns:bqbiol="http://biomodels.net/biology-qualifiers/" xmlns:bqmodel="http://biomodels.net/model-qualifiers/">
<rdf:Description rdf:about="#meta\_iPrub22\_v1">
<bqbiol:hasTaxon>
<rdf:Bag>
<rdf:li rdf:resource="https://identifiers.org/taxonomy/500485"/>
</rdf:Bag>
</bqbiol:hasTaxon>
<bqbiol:is>
<rdf:Bag>
<rdf:li rdf:resource="https://identifiers.org/insdc.gca/GCA\_000226395.1"/>
</rdf:Bag>
</bqbiol:is>
</rdf:Description>
</rdf:RDF>
</annotation>

### 1.2. Metabolites

#### 1.2.1 Model compartmentation

In the biological sense, the model is not compartmentalised (i.e. organelle modelling). Nevertheless, by convention, a GSMN is composed of an intracellular (cytosol) and extracellular compartment. This information is carried by a suffix label identifying the metabolites.

%Compartments characteristic

Name = iPrub22\_reconstruction.compNames ;

Id = iPrub22\_reconstruction.comps ;

MetaNetX = iPrub22\_reconstruction.compismetanetx\_\_46\_\_compartmentID ;

GOT = iPrub22\_reconstruction.compisgoID ;

[metId, comps] = strtok(iPrub22\_reconstruction.mets,'[') ;

Number\_of\_metabolite = [length(findMetFromCompartment(iPrub22\_reconstruction,"c")) ;length(findMetFromCompartment(iPrub22\_reconstruction,"e"))];

disp(table(Name,Id,MetaNetX,GOT,Number\_of\_metabolite))

**Name** **Id** **MetaNetX** **GOT** **Number\_of\_metabolite**
**\_\_\_\_\_\_\_\_\_\_\_\_\_\_\_** **\_\_\_** **\_\_\_\_\_\_\_\_** **\_\_\_\_\_\_\_\_\_\_\_\_** **\_\_\_\_\_\_\_\_\_\_\_\_\_\_\_\_\_\_\_\_**
'cytosol' 'c' 'MNXC3' 'GO:0005829' 5213
'extracellular' 'e' 'MNXC2' 'GO:0005576' 1404

fprintf('Total number of metabolites: <strong>%d</strong>\nNumber of unique metabolites: <strong>%d</strong> ',length(iPrub22\_reconstruction.mets),length(unique(metId)))

Total number of metabolites: **5464**
Number of unique metabolites: **5192**

#### 1.2.2 Human readable descriptive name (▲)

As the metabolite identifier is unique but not necessarily informative about the entity's nature, each metabolite is associated with a generic name to simplify the model understanding. The following example, representing the different penicillins present in the GSMN, illustrates this point. Of these four identifiers, only two are humanly understandable.

disp(iPrub22\_reconstruction.metNames(findMetIDs(iPrub22\_reconstruction,{'PENICILLIN-G[c]','CPD-9122[c]','PENICILLIN-N[c]','CPD-9196[c]'})));

'penicillin G'
'penicillin K'
'penicillin N'
'penicillin V'

#### 1.2.3 Structure identifiers (▲)

- InChI (▲) and InChIKey strings

InChI and InChIKey (hashed version of the full InChI) are unique descriptions and identifiers for chemical substances.

% Visualisation

for i = 1:5

fprintf('Metabolite name: <strong>%s</strong>\nInChi: %s\nInChiKey: %s\n\n',...

iPrub22\_reconstruction.metNames{i},iPrub22\_reconstruction.metInChIString{i},iPrub22\_reconstruction.metisinchikeyID{i})

end

Metabolite name: **&alpha;-D-glucopyranose 1-phosphate**
InChi: InChI=1S/C6H13O9P/c7-1-2-3(8)4(9)5(10)6(14-2)15-16(11,12)13/h2-10H,1H2,(H2,11,12,13)/p-2/t2-,3-,4+,5-,6-/m1/s1
InChiKey: HXXFSFRBOHSIMQ-VFUOTHLCSA-L
Metabolite name: **&alpha;-D-glucose 6-phosphate**
InChi: InChI=1S/C6H13O9P/c7-3-2(1-14-16(11,12)13)15-6(10)5(9)4(3)8/h2-10H,1H2,(H2,11,12,13)/p-2/t2-,3-,4+,5-,6+/m1/s1
InChiKey: NBSCHQHZLSJFNQ-DVKNGEFBSA-L
Metabolite name: **2-oleoylglycerol**
InChi: InChI=1S/C21H40O4/c1-2-3-4-5-6-7-8-9-10-11-12-13-14-15-16-17-21(24)25-20(18-22)19-23/h9-10,20,22-23H,2-8,11-19H2,1H3/b10-9-
InChiKey: UPWGQKDVAURUGE-KTKRTIGZSA-N
Metabolite name: **1,2-dioleoylglycerol**
InChi: InChI=1S/C39H72O5/c1-3-5-7-9-11-13-15-17-19-21-23-25-27-29-31-33-38(41)43-36-37(35-40)44-39(42)34-32-30-28-26-24-22-20-18-16-14-12-10-8-6-4-2/h17-20,37,40H,3-16,21-36H2,1-2H3/b19-17-,20-18-/t37-/m0/s1
InChiKey: AFSHUZFNMVJNKX-LLWMBOQKSA-N
Metabolite name: **coenzyme A**
InChi: InChI=1S/C21H36N7O16P3S/c1-21(2,16(31)19(32)24-4-3-12(29)23-5-6-48)8-41-47(38,39)44-46(36,37)40-7-11-15(43-45(33,34)35)14(30)20(42-11)28-10-27-13-17(22)25-9-26-18(13)28/h9-11,14-16,20,30-31,48H,3-8H2,1-2H3,(H,23,29)(H,24,32)(H,36,37)(H,38,39)(H2,22,25,26)(H2,33,34,35)/p-4/t11-,14-,15-,16+,20-/m1/s1
InChiKey: RGJOEKWQDUBAIZ-IBOSZNHHSA-J

%Sum up

fprintf(['Number of metabolites annotated by an InChI: %d (%.1f%%)\n',...

'Number of unique metabolites annotated by an InChI: %d (%.1f%%)\n',...

'Number of metabolites annotated by an InChIKey: %d (%.1f%%)\n',...

'Number of unique metabolites annotated by an InChIKey: %d (%.1f%%)\n',...

],...

length(iPrub22\_reconstruction.metInChIString(~cellfun('isempty',iPrub22\_reconstruction.metInChIString))),...

length(iPrub22\_reconstruction.metInChIString(~cellfun('isempty',iPrub22\_reconstruction.metInChIString)))\*100/length(iPrub22\_reconstruction.mets),...

length(iPrub22\_reconstruction.metInChIString(~cellfun('isempty',unique(iPrub22\_reconstruction.metInChIString)))),...

length(iPrub22\_reconstruction.metInChIString(~cellfun('isempty',unique(iPrub22\_reconstruction.metInChIString))))\*100/length(unique(iPrub22\_reconstruction.mets)),...

length(iPrub22\_reconstruction.metisinchikeyID(~cellfun('isempty',iPrub22\_reconstruction.metisinchikeyID))),...

length(iPrub22\_reconstruction.metisinchikeyID(~cellfun('isempty',iPrub22\_reconstruction.metisinchikeyID)))\*100/length(iPrub22\_reconstruction.mets),...

length(iPrub22\_reconstruction.metisinchikeyID(~cellfun('isempty',unique(iPrub22\_reconstruction.metisinchikeyID)))),...

length(iPrub22\_reconstruction.metisinchikeyID(~cellfun('isempty',unique(iPrub22\_reconstruction.metisinchikeyID))))\*100/length(unique(iPrub22\_reconstruction.mets))) ;

Number of metabolites annotated by an InChI: 3886 (71.1%)
Number of unique metabolites annotated by an InChI: 3648 (66.8%)
Number of metabolites annotated by an InChIKey: 3887 (71.1%)
Number of unique metabolites annotated by an InChIKey: 3651 (66.8%)

- Charge and chemical formula (▲)

% Visualisation

for i = 1:5

fprintf('Metabolite name: <strong>%s</strong>\nFormulae: %s\nCharge: %d\n\n',iPrub22\_reconstruction.metNames{i},iPrub22\_reconstruction.metFormulas{i},...

iPrub22\_reconstruction.metCharges(i))

end

Metabolite name: **&alpha;-D-glucopyranose 1-phosphate**
Formulae: C6H11O9P
Charge: -2
Metabolite name: **&alpha;-D-glucose 6-phosphate**
Formulae: C6H11O9P
Charge: -2
Metabolite name: **2-oleoylglycerol**
Formulae: C21H40O4
Charge: 0
Metabolite name: **1,2-dioleoylglycerol**
Formulae: C39H72O5
Charge: 0
Metabolite name: **coenzyme A**
Formulae: C21H32N7O16P3S
Charge: -4

%Sum up

fprintf(['Of the %d metabolites present in the reconstruction:\n\n',...

' %d have a <strong>chemical formula</strong> (%.1f%%)\n %d have a <strong>charge</strong> (%.1f%%)'],...

length(iPrub22\_reconstruction.mets),...

length(iPrub22\_reconstruction.metFormulas(~cellfun('isempty',iPrub22\_reconstruction.metFormulas))),...

length(iPrub22\_reconstruction.metFormulas(~cellfun('isempty',iPrub22\_reconstruction.metFormulas)))\*100/ length(iPrub22\_reconstruction.mets),...

sum(~isnan(iPrub22\_reconstruction.metCharges)),sum(~isnan(iPrub22\_reconstruction.metCharges))\*100/length(iPrub22\_reconstruction.mets)) ;

Of the 5464 metabolites present in the reconstruction:
5272 have a **chemical formula** (96.5%)
5436 have a **charge** (99.5%)

Searching for radicals in empirical formulas

chemicalElements = {'As';'Br';'C';'Ca';'Cd';'Cl';'Co';'Cu';'F';'Fe';'H';'Hg';'I';'K';'Mg';'Mn';'Mo';'N';'Na';'O';'P';'S';'Se';'Zn'} ;

for i = 1:length(chemicalElements)

fprintf('The chemical element <strong>%s</strong> is present in the empirical formula of <strong>%d metabolites</strong>\n',...

strjoin(chemicalElements(i)),length(iPrub22\_reconstruction.mets(~cellfun('isempty',(regexp(iPrub22\_reconstruction.metFormulas,chemicalElements(i))))))) ;

disp(iPrub22\_reconstruction.mets(~cellfun('isempty',(regexp(iPrub22\_reconstruction.metFormulas,chemicalElements(i)))))) ;

disp('-------------------------------------------------------------------------------')

end

The chemical element **As** is present in the empirical formula of **8 metabolites**

'CPD-763[c]'
'METHYLARSONATE[c]'
'DIMETHYLARSINATE[c]'
'METHYLARSONITE[c]'
'ARSENATE[c]'
'CPD-12152[c]'
'CPD-763[e]'
'RIBOSE-1-ARSENATE[c]'

-------------------------------------------------------------------------------

The chemical element **Br** is present in the empirical formula of **3 metabolites**

'CPD-12221[c]'
'BR-[c]'
'CPD-21209[c]'

-------------------------------------------------------------------------------

The chemical element **C** is present in the empirical formula of **5163 metabolites**

'GLC-1-P[c]'
'ALPHA-GLC-6-P[c]'
'CPD0-1812[c]'
'CPD-15977[c]'
'CO-A[c]'
'MELIBIOSE[c]'
'GALACTOSE[c]'
'ALPHA-GLUCOSE[c]'
'OLEOYL-COA[c]'
'CTP[c]'
'L-1-LYSOPHOSPHATIDATE[c]'
'CDP[c]'
'MELIBIOSE[e]'
'GALACTOSE[e]'
'ALPHA-GLUCOSE[e]'
'GLYCERALD[c]'
'NADP[c]'
'GLYCERATE[c]'
'NADPH[c]'
'D-GALACTONATE[c]'
'CPD-12575[c]'
'D-Glucosyl-12-diacyl-glycerols[c]'
'diacyl-3-O-glucl-1-6-gluc-sn-glycerol[c]'
'CPD-1070[c]'
'CPD-277[c]'
'MAL[c]'
'PYRUVATE[c]'
'CARBON-DIOXIDE[c]'
'ACETYL-COA[c]'
'GLUCOSAMINE-1P[c]'
'N-ACETYL-D-GLUCOSAMINE-1-P[c]'
'DIACYLGLYCEROL[c]'
'Phospholipids[c]'
'Triacylglycerols[c]'
'GLYCEROL[c]'
'HYDROXYPROPANAL[c]'
'ADENOSINE[c]'
'ATP[c]'
'Odd-Saturated-Fatty-Acyl-CoA[c]'
'INOSINE[c]'
'Odd-Straight-Chain-234-Sat-FA[c]'
'AMP[c]'
'ADP[c]'
'PHYTOL[c]'
'NAD[c]'
'NADH[c]'
'2E-5Z-tetradeca-2-5-dienoyl-ACPs[c]'
'PHOSPHORYL-CHOLINE[c]'
'RHAMNOSE[c]'
'CPD0-1112[c]'
'5Z-tetradec-5-enoyl-ACPs[c]'
'CPD-541[c]'
'D-MYO-INOSITOL-1-MONOPHOSPHATE[c]'
'CPD0-1122[c]'
'CPD0-1123[c]'
'CPD-171[c]'
'DOLICHOLP[c]'
'MANNOSE[c]'
'MALONYL-ACP[c]'
'Chondroitin-N-acetyl-galactosamines[c]'
'7Z-3-oxo-hexadec-7-enoyl-ACPs[c]'
'ACP[c]'
'CHONDROITIN-4-SULFATE[c]'
'Heparan-sulfate-L-iduronate[c]'
'Ubiquinones[c]'
'Heparan-sulfate-L-IdoA-2S[c]'
'Heparan-NAc-Glc-6S[c]'
'FERRICYTOCHROME-B5[c]'
'LINOLEIC\_ACID[c]'
'9-CIS11-TRANS-OCTADECADIENOATE[c]'
'CPD-8091[c]'
'FERROCYTOCHROME-B5[c]'
'CPD-8092[c]'
'LINOLENIC\_ACID[c]'
'CPD-2181[c]'
'LINOLENOYL-COA[c]'
'CPD-2182[c]'
'CPD-8088[c]'
'CPD-8093[c]'
'N-ALPHA-ACETYLORNITHINE[c]'
'L-ORNITHINE[c]'
'ACET[c]'
'2-KETOGLUTARATE[c]'
'GLT[c]'
'CPD-469[c]'
'CPD-612[c]'
'CPD-15972[c]'
'Glucopyranose[c]'
'CPD-12384[c]'
'CPD-4578[c]'
'CPD-12385[c]'
'NADH-P-OR-NOP[c]'
'S-ADENOSYLMETHIONINE[c]'
'2-OCTAPRENYL-6-HYDROXYPHENOL[c]'
'CPD-12388[c]'
'2-OCTAPRENYL-6-METHOXYPHENOL[c]'
'ADENOSYL-HOMO-CYS[c]'
'OCTAPRENYL-METHYL-METHOXY-BENZQ[c]'
'CPD-12387[c]'
'4-METHYL-824-CHOLESTADIENOL[c]'
'5-HYDROXY-CONIFERALDEHYDE[c]'
'OCTAPRENYL-METHOXY-BENZOQUINONE[c]'
'SINAPALDEHYDE[c]'
'CPD-4579[c]'
'2-OCTAPRENYLPHENOL[c]'
'CPD-12390[c]'
'CPD-12391[c]'
'CPD-4580[c]'
'CPD-12393[c]'
'CPD-4702[c]'
'CPD-12139[c]'
'CPD-19502[c]'
'CPD-19504[c]'
'CPD-19503[c]'
'3-PHENYLPROPIONATE[c]'
'CPD-14673[c]'
'Linear-Malto-Oligosaccharides[c]'
'CPD-3628[c]'
'CPD-3629[c]'
'Ox-NADPH-Hemoprotein-Reductases[c]'
'CPD-3630[c]'
'CPD-14741[c]'
'N-6-AMINOHEXANOYL-6-AMINOHEXANOATE[c]'
'CPD-884[c]'
'CYCLOARTENOL[c]'
'CPD-10689[c]'
'N1-ACETYLSPERMINE[c]'
'CPD-11271[c]'
'CPD-313[c]'
'UDP-GLUCURONATE[c]'
'CPD-11398[c]'
'UDP[c]'
'L-THYROXINE[c]'
'LIOTHYRONINE[c]'
'CPD-11400[c]'
'LYS[c]'
'8-AMINO-7-OXONONANOATE[c]'
'DIAMINONONANOATE[c]'
'R-2-HYDROXYGLUTARATE[c]'
'CPD-208[c]'
'PROTEIN-LIPOYLLYSINE[c]'
'MET[c]'
'CH33ADO[c]'
'Octanoylated-Gcv-H[c]'
'CPD-196[c]'
'a-pyruvate-dehydrogenase-E2-protein-Nsup[c]'
'pyruvate-dehydrogenase-E2-lipoyl-carrier[c]'
'DEOXYINOSINE[c]'
'HYPOXANTHINE[c]'
'DEOXY-D-RIBOSE-1-PHOSPHATE[c]'
'DNA-deoxycytidine-thymidine-dimer[c]'
'DNA-Cytidines[c]'
'DNA-thymidines[c]'
'ACETALD[c]'
'GAP[c]'
'DEOXY-RIBOSE-5P[c]'
'GLUTARATE[c]'
'GLUTARYL-COA[c]'
'MALONYL-COA[c]'
'HEXANOYL-COA[c]'
'CPD-14687[c]'
'CPD-280[c]'
'CPD-14615[c]'
'ETF-Oxidized[c]'
'CPD-11507[c]'
'GLUTACONYL-COA[c]'
'ETF-Reduced[c]'
'CPD-11506[c]'
'CPD-18[c]'
'Heparan-NAc-Glc[c]'
'CPD-235[c]'
'NAD-P-OR-NOP[c]'
'56-Dihydrouracil17-in-tRNAs[c]'
'Uracil17-in-tRNAs[c]'
'MALTOSE[c]'
'Glucose[c]'
'56-Dihydrouracil20-in-tRNAs[c]'
'LOGANATE[c]'
'Uracil20-in-tRNAs[c]'
'LOGANIN[c]'
'Isomaltose[c]'
'Long-Chain-234-Saturated-acyl-CoAs[c]'
'56-Dihydrouracil47-in-tRNAs[c]'
'Uracil47-in-tRNAs[c]'
'Long-Chain-Trans-23-Dehydroacyl-CoA[c]'
'biotin-L-lysine-in-BCCP-dimers[c]'
'HCO3[c]'
'carboxybiotin-L-lysine-in-BCCP-dimers[c]'
'CPD-8089[c]'
'CPD-8090[c]'
'BCCP-L-lysine[c]'
'BIOTIN[c]'
'BCCP-biotin-L-lysine[c]'
'1-183-2-183-SN-GLYCEROL-PHOSPHOCHOLINE[c]'
'CPD-8094[c]'
'BIPHENYL-23-DIOL[c]'
'CPD-613[c]'
'CPD-8098[c]'
'CPD-676[c]'
'3R-7Z-3-hydroxy-hexadec-7-enoyl-ACPs[c]'
'2E-7Z-hexadeca-2-7-dienoyl-ACPs[c]'
'7Z-hexadec-7-enoyl-ACPs[c]'
'9Z-3-oxo-octadec-9-enoyl-ACPs[c]'
'Ubiquinols[c]'
'CPD-4581[c]'
'UBIQUINOL-30[c]'
'UBIQUINONE-6[c]'
'Alpha-lactose[c]'
'ALLOLACTOSE[c]'
'ZYMOSTEROL[c]'
'CPD-381[c]'
'CPD0-1158[c]'
'CPD0-1162[c]'
'Oxo-glutarate-dehydrogenase-DH-lipoyl[c]'
'CPD0-1163[c]'
'5-ALPHA-CHOLESTA-724-DIEN-3-BETA-OL[c]'
'CPD-12394[c]'
'CPD-12397[c]'
'CPD-12396[c]'
'CPD-12399[c]'
'CPD-12400[c]'
'CPD-12402[c]'
'CPD-12403[c]'
'CPD-12405[c]'
'CPD-12406[c]'
'BCAA-dehydrogenase-DH-lipoyl[c]'
'PHENYLACETOTHIOHYDROXIMATE[c]'
'CPD-12607[c]'
'CPD-696[c]'
'GAMMA-BUTYROBETAINE[c]'
'CPD-3462[c]'
'SPERMIDINE[c]'
'SUCC-S-ALD[c]'
'TRIMETHYLAMINE[c]'
'CPD66-39[c]'
'Fatty-Aldehydes[c]'
'Glucosyl-ceramides[c]'
'2R-Hydroxy-Fatty-Acids[c]'
'N-ACETYLNEURAMINATE[c]'
'N-acetyl-D-mannosamine[c]'
'Ceramides[c]'
'PROTEIN-C-TERMINAL-S-ETC-CYSTEINE[c]'
'PROTEIN-C-TERMINAL-S-FARNESYL-L-CYSTEINE[c]'
'Short-Alpha-14-Glucans[c]'
'DIHYDROLIPOAMIDE[c]'
'CIS-ACONITATE[c]'
'LIPOAMIDE[c]'
'ITACONATE[c]'
'CIT[c]'
'6-DEMETHYLSTERIGMATOCYSTIN[c]'
'STERIGMATOCYSTIN[c]'
'STERIGMATOCYSTIN[e]'
'Fatty-Acids[c]'
'Ribonucleoside-Monophosphates[c]'
'CPD-11401[c]'
'CPD-11402[c]'
'CPD3O-4151[c]'
'PALMITYL-COA[c]'
'CPD-17621[c]'
'CROTONYL-COA[c]'
'GLUTATHIONE[c]'
'OXIDIZED-GLUTATHIONE[c]'
'R-4-PHOSPHOPANTOTHENOYL-L-CYSTEINE[c]'
'PANTETHEINE-P[c]'
'Pyruvate-dehydrogenase-lipoate[c]'
'a-2-oxoglutarate-dehydrogenase-E2-protei[c]'
'2-oxoglutarate-dehydrogenase-E2-lipoyl-c[c]'
'Oxo-glutarate-dehydrogenase-lipoyl[c]'
'SUC[c]'
'PHTYOSPHINGOSINE-1-P[c]'
'Rhodoquinols[c]'
'FUM[c]'
'Rhodoquinones[c]'
'SN-GLYCEROL-1-PHOSPHATE[c]'
'GLYCEROL-3P[c]'
'16-alpha-D-Mannosyloligosaccharides[c]'
'Unbranched-1-6-Mannan[c]'
'DEPHOSPHO-COA[c]'
'L-ALPHA-ALANINE[c]'
'CPD-630[c]'
'CPD-7000[c]'
'CPD-18077[c]'
'Glc2Man9GlcNAc2-proteins[c]'
'GLC[c]'
'CPD-14704[c]'
'1-4-alpha-D-Glucan[c]'
'MALTOTRIOSE[c]'
'Chitosan[c]'
'CPD-10806[c]'
'Chitosan-fragments[c]'
'ALPHA-MALTOSE[c]'
'CPD-14705[c]'
'PHOSPHORIBOSYL-CARBOXY-AMINOIMIDAZOLE[c]'
'DETHIOBIOTIN[c]'
'L-ASPARTATE[c]'
'Alpha-D-Glucuronides[c]'
'D-Glucopyranuronate[c]'
'CPD-14706[c]'
'GLY[c]'
'P-RIBOSYL-4-SUCCCARB-AMINOIMIDAZOLE[c]'
'CPD-19179[c]'
'GTP[c]'
'Guanine37-in-tRNA[c]'
'tRNA-Containing-N1-Methylguanine-37[c]'
'TMP[c]'
'ADENOSINE5TRIPHOSPHO5ADENOSINE[c]'
'DIHYDROFOLATE-GLU-N[c]'
'Guanine9-in-tRNA[c]'
'tRNA-Containing-N1-Methylguanine-9[c]'
'carbo-me-ur-34-tRNA[c]'
'5-2-me-oxy-2-oxo-et-ur-34-tRNA[c]'
'DPG[c]'
'PRECURSOR-Z[c]'
'23-DIPHOSPHOGLYCERATE[c]'
'CPD-4[c]'
'MPT-Synthase-small-subunits[c]'
'2-METHYL-3-HYDROXY-BUTYRYL-COA[c]'
'CPD-1083[c]'
'Thiocarboxylated-MPT-synthases[c]'
'tRNA-Adenine-58[c]'
'tRNA-Containing-N1-MethylAdenine-58[c]'
'BLASTICIDIN-S[c]'
'DEAMINOHYDROXYBLASTICIDIN-S[c]'
'Adenine57-Adenine58-tRNAs[c]'
'N1-MeAdenine57-MeAdenine58-tRNAs[c]'
'CPD-8122[c]'
'3R-9Z-3-hydroxy-octadec-9-enoyl-ACPs[c]'
'2E-9Z-octadeca-2-9-dienoyl-ACPs[c]'
'3-BETA-D-GLUCOSYLGLUCOSE[c]'
'CPD-1861[c]'
'CPD-4186[c]'
'CPD-4187[c]'
'METOH[c]'
'FORMATE[c]'
'CHOLESTEROL[c]'
'FORMALDEHYDE[c]'
'CPD-12449[c]'
'URACIL[c]'
'PHLORETIN[c]'
'PSEUDOURIDINE-5-P[c]'
'CPD-7727[c]'
'CPD-15317[c]'
'CPD66-21[c]'
'CPD-693[c]'
'LEUKOTRIENE-C4[c]'
'Amino-Acids-20[c]'
'5-L-GLUTAMYL-L-AMINO-ACID[c]'
'CPD-12451[c]'
'CPD-12452[c]'
'7E9E11Z14Z-5S6R-6-CYSTEIN-S-YL[c]'
'MYO-INOSITOL[c]'
'Long-Chain-Aldehydes[c]'
'Long-Chain-Acyl-CoAs[c]'
'CPD-4081[c]'
'ALPHA-METHYL-5-ALPHA-ERGOSTA[c]'
'L-ALLO-THREONINE[c]'
'CPD-4101[c]'
'AMINO-RIBOSYLAMINO-1H-3H-PYR-DIONE[c]'
'DIHYDROXY-BUTANONE-P[c]'
'CPD-19725[c]'
'CPD-7157[c]'
'PELARGONIDIN-CMPD[c]'
'CPD-591[c]'
'DNA-Cytosines[c]'
'DNA-N4-Methylcytosine[c]'
'CPD-19726[c]'
'S-SCOULERINE[c]'
'CPD-239[c]'
'4-P-PANTOTHENATE[c]'
'S-TETRAHYDROCOLUMBAMINE[c]'
'THREO-DS-ISO-CITRATE[c]'
'3-SULFINOALANINE[c]'
'L-CYSTEATE[c]'
'6-O-METHYLNORLAUDANOSOLINE[c]'
'CPD-15799[c]'
'Myelin-N-o-methyl-arginines[c]'
'R-3-Hydroxypalmitoyl-ACPs[c]'
'PAPS[c]'
'3-5-ADP[c]'
'CPD-11407[c]'
'CPD-11408[c]'
'3-oxo-palmitoyl-ACPs[c]'
'CPD-11409[c]'
'CPD-11403[c]'
'CYS[c]'
'CMP[c]'
'D-6-P-GLUCONO-DELTA-LACTONE[c]'
'RIBOSE[c]'
'RIBOSE-5P[c]'
'DGDP[c]'
'Carboxyadenylated-MPT-synthases[c]'
'L-Cysteine-Desulfurase-persulfide[c]'
'Cysteine-Desulfurase-L-cysteine[c]'
'D-altropyranoses[c]'
'PSICOSE[c]'
'CHITIN[c]'
'ETR-Quinones[c]'
'ETR-Quinols[c]'
'OXALACETIC\_ACID[c]'
'Chitodextrins[c]'
'ITP[c]'
'PHOSPHO-ENOL-PYRUVATE[c]'
'IDP[c]'
'Poly-ADP-Riboses[c]'
'ADENOSINE\_DIPHOSPHATE\_RIBOSE[c]'
'Xyloglucan[c]'
'Xyloglucan-oligosaccharides[c]'
'GLUCOSAMINE[c]'
'Peptidoglycans[c]'
'NAcMur-Peptide-Undecaprenols[c]'
'N-acetyl-D-glucosamine[c]'
'BUTANAL[c]'
'ALPHA-TOCOPHEROL[c]'
'GAMA-TOCOPHEROL[c]'
'RETINAL[c]'
'CPD-17278[c]'
'RETINOATE[c]'
'Alpha-linolenoyl-groups[c]'
'3-Oxo-Delta-4-Steroids[c]'
'3-Oxo-5-Alpha-Steroids[c]'
'CPD-8123[c]'
'Mercapturates[c]'
'S-Substituted-L-Cysteines[c]'
'L-GAMMA-GLUTAMYLCYSTEINE[c]'
'3-Beta-Hydroxysterols[c]'
'CPD-18246[c]'
'Malonyl-acp-methyl-ester[c]'
'3-Ketoglutaryl-ACP-methyl-ester[c]'
'ILE[c]'
'2-KETO-3-METHYL-VALERATE[c]'
'PROPIONYL-COA[c]'
'D-METHYL-MALONYL-COA[c]'
'LEU[c]'
'FADH2[c]'
'2K-4CH3-PENTANOATE[c]'
'FAD[c]'
'GLYCOLLATE[c]'
'Folates[c]'
'P-COUMAROYL-COA[c]'
'CAFFEOYL-COA[c]'
'CARNITINE[c]'
'CPD-19737[c]'
'CPD66-29[c]'
'BUTYRYL-COA[c]'
'CPD-19738[c]'
'3-BETA-HYDROXYANDROST-5-EN-17-ONE[c]'
'TETRADECANOYL-COA[c]'
'CPD-19740[c]'
'CPD-10267[c]'
'CPD-19741[c]'
'STEAROYL-COA[c]'
'CPD-19742[c]'
'17-BETA-HYDROXY-5ALPHA-ANDROSTAN-3-O[c]'
'Oleoyl-ACPs[c]'
'ACETYL-ACP[c]'
'11Z-3-oxo-icos-11-enoyl-ACPs[c]'
'CPD-4126[c]'
'L-GLYCERALDEHYDE-3-PHOSPHATE[c]'
'Cytidine-34-tRNAmet[c]'
'CPD-4125[c]'
'CPD-4127[c]'
'Elongator-tRNAMet-acetylcytidine[c]'
'CPD-4142[c]'
'DEHYDRO-3-DEOXY-L-RHAMNONATE[c]'
'CPD-706[c]'
'CPD-4141[c]'
'LACTALD[c]'
'CPD-4143[c]'
'CPD-707[c]'
'Red-NADPH-Hemoprotein-Reductases[c]'
'CPD-3943[c]'
'DIMETHYL-D-RIBITYL-LUMAZINE[c]'
'Myelin-L-arginines[c]'
'S-NORCOCLAURINE[c]'
'S-COCLAURINE[c]'
'DIHYDROSIROHYDROCHLORIN[c]'
'CPD-642[c]'
'METHIONINE-SYNTHASE-METHYLCOBALAMIN[c]'
'Methionine-synthase-cob-II-alamins[c]'
'2-Hexadecenoyl-ACPs[c]'
'Palmitoyl-ACPs[c]'
'GLYOX[c]'
'CPD-11411[c]'
'CPD-11404[c]'
'CPD-11412[c]'
'CPD-11410[c]'
'METHYLENE-THF-GLU-N[c]'
'5-10-METHENYL-THF-GLU-N[c]'
'CPD-1130[c]'
'CPD-3618[c]'
'L-DIHYDROXY-PHENYLALANINE[c]'
'COUMARATE[c]'
'Release-factor-L-glutamine[c]'
'Release-factor-N5-Methyl-L-glutamine[c]'
'CPD-15896[c]'
'CPD-17487[c]'
'DGTP[c]'
'CPD-674[c]'
'2-COUMARATE[c]'
'DEOXYGUANOSINE[c]'
'2-3-DIHYDROXYBENZOATE[c]'
'CPD-664[c]'
'DELTA1-PYRROLINE\_2-CARBOXYLATE[c]'
'3-Oxosteroids[c]'
'CPD-8124[c]'
'CPD-4161[c]'
'L-GULONO-1-4-LACTONE[c]'
'CPD-8134[c]'
'ASCORBATE[c]'
'CPD-8155[c]'
'CPD-7061[c]'
'4-AMINO-BUTYRATE[c]'
'CPD-8157[c]'
'CPD-8158[c]'
'ETOH[c]'
'TYR[c]'
'P-HYDROXY-PHENYLPYRUVATE[c]'
'Peptides-holder[c]'
'CPD-11495[c]'
'PHENYLACETATE[c]'
'CPD0-2244[c]'
'CPD0-2123[c]'
'Beta-D-glucosides[c]'
'5-METHYL-THF-GLU-N[c]'
'FORMYL-THF-GLU-N[c]'
'D-glucopyranose-6-phosphate[c]'
'Beta-D-Galactosides[c]'
'THF[c]'
'D-galactopyranose[c]'
'LAUROYLCOA-CPD[c]'
'LYS-tRNAs[c]'
'CPD-19743[c]'
'Charged-LYS-tRNAs[c]'
'CHOLINE[c]'
'GLYCOLALDEHYDE[c]'
'CPD-7682[c]'
'4-HYDROXY-BUTYRYL-COA[c]'
'OH-CROTONYL-COA[c]'
'3-Hydroxyglutaryl-ACP-methyl-ester[c]'
'3-P-SERINE[c]'
'CPD-19754[c]'
'ECTOINE[c]'
'CPD-19757[c]'
'CPD-10663[c]'
'CPD-19760[c]'
'2-Lysophosphatidylcholines[c]'
'HYDROQUINONE[c]'
'Enoylglutaryl-ACP-methyl-esters[c]'
'Carboxylates[c]'
'L-1-GLYCERO-PHOSPHORYLCHOLINE[c]'
'26-DICHLORO-P-HYDROQUINONE[c]'
'VAL[c]'
'2-KETO-ISOVALERATE[c]'
'PYRROLINE-HYDROXY-CARBOXYLATE[c]'
'4-HYDROXY-L-PROLINE[c]'
'ACYL-COA[c]'
'TESTOSTERONE[c]'
'3R-11Z-3-hydroxy-icos-11-enoyl-ACPs[c]'
'CPD66-23[c]'
'2E-11Z-icosa-2-11-dienoyl-ACPs[c]'
'11Z-icos-11-enoyl-ACPs[c]'
'CPD66-27[c]'
'CYS-tRNAs[c]'
'CPD-698[c]'
'ACETYLSERINE[c]'
'CPD-3945[c]'
'CPD-4162[c]'
'CPD-4181[c]'
'4-ALPHA-METHYL-5-ALPHA[c]'
'METHYLARSONATE[c]'
'DIMETHYLARSINATE[c]'
'METHYLARSONITE[c]'
'CPD-4124[c]'
'CPD-225[c]'
'E-2-METHOXYCARBONYLMETHYLBUTENEDIOAT[c]'
'L-1-PHOSPHATIDYL-ETHANOLAMINE[c]'
'CPD-10260[c]'
'DIHYDRO-DIOH-BENZOATE[c]'
'CPD-10261[c]'
'CPD-10262[c]'
'THF-GLU-N[c]'
'CPD-9956[c]'
'DIHYDRO-NEO-PTERIN[c]'
'DIHYDRONEOPTERIN-P3[c]'
'OCTAPRENYL-METHYL-OH-METHOXY-BENZQ[c]'
'DIHYDROFOLATE[c]'
'Stearoyl-ACPs[c]'
'12-DICHLOROETHANE[c]'
'2-CHLOROETHANOL[c]'
'CL-[c]'
'GERANYLGERANYL-PP[c]'
'LysW-L-glutamate[c]'
'CPD-12805[c]'
'LysW-L-glutamate-5-phosphate[c]'
'18-HYDROXYOLEATE[c]'
'LysW-L-glutamate-5-semialdehyde[c]'
'CPD-21340[c]'
'910-EPOXY-18-HYDROXYSTEARATE[c]'
'GERANYL-PP[c]'
'DELTA3-ISOPENTENYL-PP[c]'
'CPD-11444[c]'
'COPROPORPHYRINOGEN\_I[c]'
'LysW-L-ornithine[c]'
'ALL-TRANS-HEXAPRENYL-DIPHOSPHATE[c]'
'CPD-15900[c]'
'3-HYDROXYPIMELYL-COA[c]'
'CPD-11447[c]'
'CPD1F-114[c]'
'L-ARABINOSE[e]'
'CPD-7733[c]'
'L-ARABINOSE[c]'
'CPD-15913[c]'
'Cis-Delta5-dodecenoyl-ACPs[c]'
'L-GULONATE[c]'
'D-GLUCURONOLACTONE[c]'
'2-OXOBUTANOATE[c]'
'2-ACETO-2-HYDROXY-BUTYRATE[c]'
'Charged-GLT-tRNAs[c]'
'GLT-tRNAs[c]'
'5-HYDROXY-FERULIC-ACID[c]'
'SINAPATE[c]'
'DOPAMINE[c]'
'ARABINOSE-5P[c]'
'RIBULOSE-5P[c]'
'Saturated-Fatty-Acyl-CoA[c]'
'G3P[c]'
'CPD-8620[c]'
'CPD-8619[c]'
'Sulfhydryls[c]'
'CPD-8529[c]'
'CPD-8621[c]'
'NOREPINEPHRINE[c]'
'L-EPINEPHRINE[c]'
'Methyl-thioethers[c]'
'DCDP[c]'
'DCTP[c]'
'CPD-621[c]'
'CPD-8065[c]'
'CPD-170[c]'
'CPD-8066[c]'
'CPD-1099[c]'
'SUCROSE[c]'
'CPD-8073[c]'
'CPD-8074[c]'
'CPD-8075[c]'
'CPD-8076[c]'
'CPD-7496[c]'
'CPD-7524[c]'
'CPD-12335[c]'
'CPD-12336[c]'
'CPD-12334[c]'
'CPD-14468[c]'
'E-11-TETRADECENOYL-COA[c]'
'CPD-17811[c]'
'CPD-17814[c]'
'CPD-17813[c]'
'L-LACTATE[c]'
'CPD-17815[c]'
'3-OXOPALMITOYL-COA[c]'
'CPD-17464[c]'
'CPD-358[c]'
'tRNA-Containing-N2-Dimethylgua-26-Gua27[c]'
'tRNA-Containing-N2-Methylgua-26-Gua27[c]'
'tRNA-Containing-N2-Dimetgua-26-MeGua27[c]'
'tRNA-Containing-N2-DiMeGua-26-DiMeGua27[c]'
'Guanine26-Guanine27-in-tRNAs[c]'
'Dodecanoyl-ACPs[c]'
'3-oxo-myristoyl-ACPs[c]'
'CPD-15684[c]'
'CPD-15685[c]'
'CPD-19273[c]'
'CPD-15686[c]'
'CPD-15687[c]'
'CPD-15688[c]'
'CPD-15689[c]'
'CPD-15690[c]'
'NYSTOSE[c]'
'1-KESTOTRIOSE[c]'
'CPD-15692[c]'
'CPD-15691[c]'
'H2CO3[c]'
'IMIDAZOLE\_ACETALDEHYDE[c]'
'CPD-14465[c]'
'CPD-14459[c]'
'CPD-14466[c]'
'CPD-14464[c]'
'CPD-14467[c]'
'4-IMIDAZOLEACETATE[c]'
'CPD-10847[c]'
'CPD-12358[c]'
'CPD-14471[c]'
'CPD-10844[c]'
'ACETOACETYL-COA[c]'
'2-Oxo-carboxylates[c]'
'L-GLUTAMATE\_GAMMA-SEMIALDEHYDE[c]'
'INOSITOL-1-4-5-TRISPHOSPHATE[c]'
'INOSITOL-1-4-BISPHOSPHATE[c]'
'PRO[c]'
'D-MYO-INOSITOL-4-PHOSPHATE[c]'
'PHOSPHATIDYLINOSITOL-345-TRIPHOSPHATE[c]'
'PHOSPHATIDYL-MYO-INOSITOL-45-BISPHOSPHA[c]'
'GUANOSINE[c]'
'GUANINE[c]'
'BENZALDEHYDE[c]'
'CPD0-1065[c]'
'BENZOATE[c]'
'CADAVERINE[c]'
'5-METHYLTHIOADENOSINE[c]'
'ALPHA-L-GLUTAMYL-PHOSPHATE[c]'
'S-ADENOSYLMETHIONINAMINE[c]'
'CARBAMATE[c]'
'BENZOYLCOA[c]'
'CPD-20052[c]'
'3-CARBOXY-3-HYDROXY-ISOCAPROATE[c]'
'CPD-318[c]'
'CPD-20051[c]'
'DCMP[c]'
'DUMP[c]'
'GlgE-Glycogen[c]'
'2K-ADIPATE[c]'
'DAIDZEIN[c]'
'Thiopurine-Methylethers[c]'
'CPD-8646[c]'
'Thiopurines[c]'
'DESMOSTEROL-CPD[c]'
'Red-Thioredoxin[c]'
'CPD-465[c]'
'SQUALENE[c]'
'CPD-10204[c]'
'Ox-Thioredoxin[c]'
'GDP-MANNOSE[c]'
'GDP-L-GALACTOSE[c]'
'D-LACTATE[c]'
'4-hydroxybenzoate[c]'
'CPD-7875[c]'
'4-Hydroxy-3-polyprenylbenzoates[c]'
'Polyisoprenyl-Diphosphates[c]'
'2-3-CARBOXY-3-AMINOPROPYL-L-HISTIDINE[c]'
'DTDP-DEOH-DEOXY-GLUCOSE[c]'
'L-Galactopyranose[c]'
'CPD-13952[c]'
'2-3-CARBOXY-3-METHYLAMMONIOPROPYL-L-[c]'
'CPD-9326[c]'
'eEF-2-Histidines[c]'
'CPD-17870[c]'
'3-carboxy-3-dimethylammonio-propyl-L-his[c]'
'DIPHTINE[c]'
'CPD-17877[c]'
'EPOXYSQUALENE[c]'
'LANOSTEROL[c]'
'CPD-17876[c]'
'CPD-2189[c]'
'CPD-8078[c]'
'CPD-330[c]'
'CPD-8080[c]'
'CPD-2190[c]'
'CPD-8077[c]'
'CPD-8079[c]'
'CPD-6948[c]'
'CPD-9459[c]'
'CPD-259[c]'
'CPD-8130[c]'
'CPD-9777[c]'
'CPD-9775[c]'
'CPD-401[c]'
'CPD-1823[c]'
'B-ALANINE[c]'
'XANTHOSINE-5-PHOSPHATE[c]'
'XANTHINE[c]'
'PRPP[c]'
'URATE[c]'
'CPD-1103[c]'
'4-HYDROXYBENZALDEHYDE[c]'
'CPD-7207[c]'
'CPD-13371[c]'
'GERANIAL[c]'
'CPD-13376[c]'
'CPD-13375[c]'
'CPD-13377[c]'
'CELLULOSE[c]'
'Cellulose-D-glucono-1-5-lactone[c]'
'Cytochromes-B-Oxidized[c]'
'Cytochromes-B-Reduced[c]'
'Cellodextrins[c]'
'CPD-7043[c]'
'CPD-3746[c]'
'FMNH2[c]'
'FMN[c]'
'D-ALPHABETA-D-HEPTOSE-7-PHOSPHATE[c]'
'T2-DECENOYL-COA[c]'
'CPD-10849[c]'
'CPD-10845[c]'
'CPD-12777[c]'
'D-arabinofuranose[c]'
'D-arabinopyranose[c]'
'CPD-9152[c]'
'L-arabinofuranose[c]'
'CPD-10870[c]'
'L-arabinopyranose[c]'
'MANNOSE-6P[c]'
'CPD-15711[c]'
'CPD-1241[c]'
'CPD-15712[c]'
'CPD-10866[c]'
'CPD-9151[c]'
'DUTP[c]'
'D-CYSTEINE[c]'
'FORMONONETIN[c]'
'VESTITONE[c]'
'2-HYDROXYFORMONONETIN[c]'
'CPD-3441[c]'
'ACETYL-GLU[c]'
'CPD-217[c]'
'CPD-3402[c]'
'CPD-3502[c]'
'2-HYDROXYISOFLAVANONE[c]'
'OROTIDINE-5-PHOSPHATE[c]'
'GLN[c]'
'OROTATE[c]'
'2-DEOXY-D-GLUCOSE-6-PHOSPHATE[c]'
'2-DEOXY-D-GLUCOSE[c]'
'PYRIDOXAL[c]'
'D-BETA-D-HEPTOSE-1-P[c]'
'PYRIDOXAL\_PHOSPHATE[c]'
'CPD-9001[c]'
'CPD-9002[c]'
'CPD-85[c]'
'CPD-8999[c]'
'CPD-10637[c]'
'CPD0-1080[c]'
'CPD0-1081[c]'
'CPD0-1082[c]'
'2-METHYL-BUTYRYL-COA[c]'
'CPD-10642[c]'
'N-ACETYL-D-GLUCOSAMINE[c]'
'CPD0-882[c]'
'L-ALA-GAMMA-D-GLU-DAP[c]'
'D-ALANINE[c]'
'4-TRIMETHYLAMMONIOBUTANAL[c]'
'Benzoin[c]'
'CPD-9869[c]'
'3-HYDROXY-N6N6N6-TRIMETHYL-L-LYSINE[c]'
'CPD-9871[c]'
'PHENYLGLYOXYLATE[c]'
'HCN[c]'
'CPD-17873[c]'
'HSCN[c]'
'CPD-17874[c]'
'CPD-8082[c]'
'CPD-8084[c]'
'CPD-16968[c]'
'CPD-17881[c]'
'CPD-17880[c]'
'CPD-17882[c]'
'CPD-8081[c]'
'CPD-8083[c]'
'CPD-9873[c]'
'CPD-4568[c]'
'LEU-tRNAs[c]'
'Charged-LEU-tRNAs[c]'
'Sphingoids[c]'
'Sphingoid-1-phosphates[c]'
'CPD0-1083[c]'
'CPD-12349[c]'
'L-GLYCERALDEHYDE[c]'
'CPD-460[c]'
'CPD-12352[c]'
'CPD-14594[c]'
'LINAMARIN[c]'
'CPD-19388[c]'
'CPD-15277[c]'
'CYS-GLY[c]'
'GLYCYLGLYCINE[c]'
'CPD-19395[c]'
'CPD-13031[c]'
'CPD-12702[c]'
'PHENYLACETONITRILE[c]'
'INDOLE-3-ACETALDOXIME[c]'
'CPD-13378[c]'
'R-3-hydroxymyristoyl-ACPs[c]'
'RIBOSE-1P[c]'
'XANTHOSINE[c]'
'CPD-13417[c]'
'CPD-13418[c]'
'CPD0-1202[c]'
'XYLOSE[c]'
'NEUROSPORENE[c]'
'CPD-10868[c]'
'CPD-294[c]'
'VERY-LONG-CHAIN-FATTY-ACYL-COA[c]'
'CPD-15723[c]'
'CPD-10615[c]'
'3-phosphooligonucleotides[c]'
'3-Prime-Nucleoside-Monophosphates[c]'
'CPD-15728[c]'
'Oligonucleotides[c]'
'Nucleoside-Monophosphates[c]'
'24-DICHLOROPHENOL[c]'
'CPD-8924[c]'
'CPD-15741[c]'
'L-1-phosphatidyl-inositols[c]'
'CPD-1121[c]'
'ALTROSE[c]'
'CPD-15781[c]'
'CPD-397[c]'
'CPD-15801[c]'
'CPD-377[c]'
'25-DIDEHYDRO-D-GLUCONATE[c]'
'ALLO-THR[c]'
'DIHYDROXY-ACETONE-PHOSPHATE[c]'
'TARTRONATE-S-ALD[c]'
'DEOXYCYTIDINE[c]'
'BENZYL-ALCOHOL[c]'
'3-OH-BENZYL-ALCOHOL[c]'
'3-OH-BENZALDEHYDE[c]'
'LEUCOPELARGONIDIN-CMPD[c]'
'BETA-CYCLOPIAZONATE[c]'
'ALPHA-CYCLOPIAZONATE[c]'
'UMP[c]'
'ACETYLCHOLINE[c]'
'PHOSPHORIBULOSYL-FORMIMINO-AICAR-P[c]'
'NONANE-46-DIONE[c]'
'Acetate-esters[c]'
'D-ERYTHRO-IMIDAZOLE-GLYCEROL-P[c]'
'CPD-4573[c]'
'44-DIMETHYL-CHOLESTA-814-24-TRIENOL[c]'
'44-DIMETHYL-824-CHOLESTADIENOL[c]'
'CPD-622[c]'
'CPD-1136[c]'
'CPD-8087[c]'
'CPD-8086[c]'
'CATECHOL[c]'
'CPD-12356[c]'
'CPD-12357[c]'
'CPD-14460[c]'
'CPD-12359[c]'
'CPD0-1905[c]'
'CPD-12365[c]'
'D-XYLONATE[c]'
'2-DH-3-DO-D-ARABINONATE[c]'
'D-XYLULOSE[c]'
'XYLULOSE-5-PHOSPHATE[c]'
'Very-Long-Chain-Aldehydes[c]'
'Alkanes[c]'
'Secondary-Alcohols[c]'
'Very-Long-Chain-Alkanes[c]'
'CARBON-MONOXIDE[c]'
'CPD-12321[c]'
'CPD-15798[c]'
'CPD-12932[c]'
'CPD-19475[c]'
'2-OXO-5-METHYLTHIOPENTANOIC-ACID[c]'
'CPD-8347[c]'
'PALMITATE[c]'
'CPDQT-40[c]'
'CPD-14596[c]'
'CPD-15800[c]'
'CPD-10277[c]'
'CPD-15742[c]'
'CPDQT-39[c]'
'CPD-14601[c]'
'CPD-14602[c]'
'CPD-19488[c]'
'PHOSPHATIDYLCHOLINE[c]'
'CPD-14604[c]'
'CPDQT-38[c]'
'CPD-19489[c]'
'Tetradec-2-enoyl-ACPs[c]'
'DEOXYADENOSINE[c]'
'DAMP[c]'
'ADENINE[c]'
'DADP[c]'
'2-DEHYDRO-3-DEOXY-D-GLUCONATE[c]'
'2-KETO-3-DEOXY-6-P-GLUCONATE[c]'
'AICAR[c]'
'Charged-THR-tRNAs[c]'
'Cyclic-3-5-Nucleoside-Monophosphates[c]'
'THR[c]'
'CPD-35[c]'
'D-THREONINE[c]'
'Cyclic-2-3-Ribonucleoside-Monophosphates[c]'
'AMINO-OXOBUT[c]'
'2-Prime-Ribonucleoside-Monophosphates[c]'
'AMINO-ACETONE[c]'
'GLN-tRNAs[c]'
'3Z-dodec-3-enoyl-ACPs[c]'
'OXALATE[c]'
'OXALYL-COA[c]'
'CPD-1162[c]'
'GLC-6-P[c]'
'CPD-1181[c]'
'3R-5Z-3-hydroxy-tetradec-5-enoyl-ACPs[c]'
'5Z-3-oxo-tetradec-5-enoyl-ACPs[c]'
'CPD-173[c]'
'BUTYRIC\_ACID[c]'
'PENTAN-2-ONE[c]'
'Beta-D-Glucuronides[c]'
'Beta-Lactams[c]'
'CPD-8550[c]'
'CPD-448[c]'
'3-UREIDO-PROPIONATE[c]'
'N-ACETYL-GLUTAMYL-P[c]'
'CPD-4575[c]'
'CPD-4576[c]'
'CPD-667[c]'
'HOMO-CYS[c]'
'CPD-4577[c]'
'CPD-10254[c]'
'CPD-8892[c]'
'CPD-15[c]'
'245-DNOL[c]'
'SUC-COA[c]'
'25-DDOL[c]'
'3-KETO-ADIPYL-COA[c]'
'CPD-258[c]'
'CPD-320[c]'
'BENZENE-NO2[c]'
'CPD-12364[c]'
'PHE[c]'
'CPD-12363[c]'
'CPD-12521[c]'
'CPD-12595[c]'
'CPD-19490[c]'
'MALEATE[c]'
'CPDQT-37[c]'
'CPD-11268[c]'
'CPD-568[c]'
'CPD-10687[c]'
'CPDQT-36[c]'
'CPD-19491[c]'
'CPD-19492[c]'
'ACYL-ACP[c]'
'ACYL-SN-GLYCEROL-3P[c]'
'CPD-3740[c]'
'CPD-19493[c]'
'Omega-methylthio-alkyl-glucosinolates[c]'
'CPD-30[c]'
'omega-methylsulfinylalkylglucosinolate[c]'
'CPD-6082[c]'
'NORSPERMIDINE[c]'
'L-DELTA1-PYRROLINE\_5-CARBOXYLATE[c]'
'Protein-Phosphothreonines[c]'
'Proteins-L-Threonines[c]'
'CPD-15834[c]'
'CPD-11712[c]'
'ALLYSINE[c]'
'CAAL-proteins[c]'
'Geranylgeranylated-CAAL-proteins[c]'
'CPD-9539[c]'
'L-DEHYDRO-ASCORBATE[c]'
'OXAMATE[c]'
'DGMP[c]'
'CPD-389[c]'
'CARBAMOYL-P[c]'
'CPD-316[c]'
'RIBOFLAVIN[c]'
'CPD-9973[c]'
'LONG-CHAIN-KETONE[c]'
'EIF5A-HYPUSINE[c]'
'Charged-GLN-tRNAs[c]'
'CPD-3617[c]'
'O-PHOSPHO-L-HOMOSERINE[c]'
'THYMIDINE[c]'
'Myristoyl-ACPs[c]'
'THYMINE[c]'
'D-SEDOHEPTULOSE-7-P[c]'
'D-RIBULOSE[c]'
'CPD-15567[c]'
'CPD-15568[c]'
'PROPIONAMIDE[c]'
'CPD-8860[c]'
'BUTYRAMIDE[c]'
'CPD-12327[c]'
'CPD-3707[c]'
'PROPIONATE[c]'
'CPD-8548[c]'
'CPD-8549[c]'
'CPD-369[c]'
'CPD-15616[c]'
'TETRACHLOROHYDROQUINONE[c]'
'236-TRICHLOROHYDROQUINONE[c]'
'Cytochromes-C-Oxidized[c]'
'Cytochromes-C-Reduced[c]'
'CPD-13025[c]'
'CPD-13223[c]'
'SPERMIDINE[e]'
'TRANS-2-HEXENOL[c]'
'TRANS-2-HEXENAL[c]'
'PUTRESCINE[e]'
'CIS-3-HEXENAL[c]'
'PUTRESCINE[c]'
'CIS-3-HEXENOL[c]'
'Pullulans[c]'
'7-O-ACETYLSALUTARIDINOL[c]'
'CPD-7710[c]'
'CPD-7712[c]'
'CPD-7713[c]'
'CODEINONE[c]'
'MORPHINONE[c]'
'CPD-10802[c]'
'CPD-10783[c]'
'METOH[e]'
'MYO-INOSITOL[e]'
'CPD-7836[e]'
'CPD-10784[c]'
'N-ACETYL-D-GLUCOSAMINE[e]'
'CPD-10803[c]'
'NADH-P-OR-NOP[e]'
'NADP[e]'
'CPD-10780[c]'
'NADPH[e]'
'CPD-10804[c]'
'NIACINAMIDE[e]'
'CPD-10785[c]'
'NIACINE[e]'
'CPD-10805[c]'
'2-METHYL-6-SOLANYL-14-BENZOQUINONE[c]'
'MALONATE-S-ALD[c]'
'CPD-14553[c]'
'CPD0-935[c]'
'CPD0-936[c]'
'CPD0-937[c]'
'CPD0-938[c]'
'CPD-17722[c]'
'CPD-17747[c]'
'OLEATE-CPD[c]'
'CPD-14378[c]'
'CPD-17726[c]'
'CPD-17727[c]'
'CPD-17728[c]'
'Amino-Acids[c]'
'CPD-17729[c]'
'2-Oxo-Acids[c]'
'Deoxyhypusine-Synthase-Lysine[c]'
'N-4-aminobutylidene-enzyme-lysine[c]'
'CPD-17732[c]'
'EIF5A-LYSINE[c]'
'CPD-17733[c]'
'DODECANOATE[c]'
'N-4-aminobutylidene-eIF5A-lysine[c]'
'General-Protein-Substrates[c]'
'CPD-17741[c]'
'Decanoyl-ACPs[c]'
'DNA-with-3-prime-pp-5-prime-G-cap[c]'
'3-Prime-Phosphate-Terminated-DNAs[c]'
'GMP[c]'
'3-oxo-dodecanoyl-ACPs[c]'
'DNA-Ligase-L-lysine-adenylate[c]'
'DNA-Ligase-L-lysine[c]'
'CPD-845[c]'
'A-5-prime-PP-5-prime-DNA[c]'
'O-SUCCINYLBENZOATE[c]'
'CPD-9923[c]'
'GLC-D-LACTONE[c]'
'GLUCONATE[c]'
'HEPTADECANE-CPD[c]'
'CPD-13469[c]'
'CPD-55[c]'
'FRUCTOSE-6P[c]'
'CPD-8611[c]'
'2-ACETO-LACTATE[c]'
'CPD-231[c]'
'CPD-8612[c]'
'CPD-2750[c]'
'D-Xylopyranose[c]'
'CPD-2742[c]'
'CPD-360[c]'
'CPD-409[c]'
'1-2-Diglycerides[c]'
'CPD-2752[c]'
'CPD-468[c]'
'XYLITOL[c]'
'ANDROST4ENE[c]'
'CPD-8505[e]'
'CPD-10174[c]'
'CPD-10174[e]'
'CPD-195[e]'
'OLEATE-CPD[e]'
'OXALATE[e]'
'OXALACETIC\_ACID[e]'
'PALMITATE[e]'
'PANTOTHENATE[e]'
'CPD-8462[e]'
'CPD-10902[e]'
'PHENYLACETATE[e]'
'PLASTOQUINONE[c]'
'Plastoquinols[c]'
'CPD-13205[c]'
'CELLOBIOSE[c]'
'Phosphatase-2A-leucine[c]'
'Phosphatase-2A-leucine-methyl-ester[c]'
'SARCOSINE[c]'
'CODEINE[c]'
'MORPHINE[c]'
'CPD-4592[c]'
'CPD-4592[e]'
'ACETAMIDE[c]'
'D-Galactopyranuronate[c]'
'CPD-15633[c]'
'CPD-219[c]'
'CPD-15666[c]'
'2-KETO-6-AMINO-CAPROATE[c]'
'CPD-10809[c]'
'DIAMINO-OH-PHOSPHORIBOSYLAMINO-PYR[c]'
'CPD-1086[c]'
'Lignoceroyl-ACPs[c]'
'3-oxo-cerotoyl-ACPs[c]'
'TRINAPHTAL-CPD[c]'
'CPD-17743[c]'
'CPD-17730[c]'
'S-HYDROXYMETHYLGLUTATHIONE[c]'
'CPD-17744[c]'
'CPD-17746[c]'
'CPD-17750[c]'
'CPD-548[c]'
'CPD-17757[c]'
'CPD-13122[c]'
'CPD-702[c]'
'CPD-703[c]'
'FARNESYL-PP[c]'
'CPD0-1028[c]'
'MALTOHEXAOSE[c]'
'MALTOTETRAOSE[c]'
'CPD-12221[c]'
'CPD-3745[c]'
'CPD-201[c]'
'CPD-202[c]'
'DIACYLGLYCEROL-PYROPHOSPHATE[c]'
'L-PHOSPHATIDATE[c]'
'DNA-N[c]'
'CHOLATE[c]'
'CPD-14388[c]'
'3-Hydroxy-Terminated-DNAs[c]'
'Deacetylated-Peptidoglycan[c]'
'NICOTINAMIDE\_NUCLEOTIDE[c]'
'CPD-9776[c]'
'DNA-Ligase-L-lysine-guanylate[c]'
'CPD-14389[c]'
'CPD1F-140[c]'
'tRNA-precursors[c]'
'SS-Oligoribonucleotides[c]'
'CPD-236[c]'
'5-Phospho-terminated-DNAs[c]'
'CPD-14390[c]'
'CPD-2751[c]'
'D-GLUCOSAMINE-6-P[c]'
'CPD-205[e]'
'PROPIONATE[e]'
'N-ACETYL-D-GLUCOSAMINE-6-P[c]'
'CPD-2747[c]'
'PYRUVATE[e]'
'CPD-3188[c]'
'QUINATE[e]'
'CPD-1099[e]'
'CPD-8613[c]'
'Red-NADPH-Hemoprotein-Reductases[e]'
'CPD-2749[c]'
'RIBOFLAVIN[e]'
'R-3-hydroxydodecanoyl-ACPs[c]'
'OCTANOL[c]'
'CPD-371[c]'
'CPD-7616[c]'
'3-4-DIHYDROXYBENZOATE[c]'
'THZ-P[c]'
'AMINO-HYDROXYMETHYL-METHYLPYRIMIDINE-PP[c]'
'THIAMINE-P[c]'
'THIAMINE-PYROPHOSPHATE[c]'
'CPD-611[c]'
'THIAMINE[c]'
'PYRIDINE[c]'
'THZ[c]'
'2-CARBOXY-D-ARABINITOL[c]'
'2-CARBOXY-D-ARABINITOL-1-PHOSPHATASE[c]'
'ACETYL-P[c]'
'3-KETOBUTYRATE[c]'
'L-PIPECOLATE[c]'
'Alpha-1-3-Branched-Arabinans[c]'
'L-RHAMNONATE[c]'
'L-RHAMNONO-14-LACTONE[c]'
'CPD1F-129[c]'
'CPD-7850[c]'
'GERANIOL[c]'
'CPD-7857[c]'
'CPD-7860[c]'
'CPD-7849[c]'
'CPD-15637[c]'
'CPD-15653[c]'
'CPD-15668[c]'
'CPD-15667[c]'
'CPD-15654[c]'
'CPD-15655[c]'
'CPD-13665[c]'
'5-BETA-ANDROSTANE-317-DIONE[c]'
'CPD-125[c]'
'CPD-14077[c]'
'R-3-hydroxycerotoyl-ACPs[c]'
'NONAPRENYL-4-HYDROXYBENZOATE[c]'
'Trans-D2-hexacos-2-enoyl-ACPs[c]'
'CPD-107[c]'
'CPD-9863[c]'
'Cerotoyl-ACPs[c]'
'CPD-14392[c]'
'Sphingomyelins[e]'
'CPD-11541[c]'
'STEARIC\_ACID[e]'
'CPD-14018[c]'
'Sterols[e]'
'5Z8Z11Z14Z17Z-EICOSAPENTAENOATE[c]'
'Steryl-Esters[e]'
'SUC[e]'
'SUCROSE[e]'
'CPD-6951[c]'
'FERULOYL-COA[c]'
'CPD-501[c]'
'UTP[c]'
'UDP-D-GALACTURONATE[c]'
'CPD-8633[c]'
'CPD-8634[c]'
'CPD-12231[c]'
'CPD-12261[c]'
'CPD-15377[c]'
'CPD-9868[c]'
'CPD-12279[c]'
'CPD-108[c]'
'CPD-9866[c]'
'RNA-DNA-hybrids[c]'
'DNA-Holder[c]'
'CPD-2961[c]'
'RNA-Containing-Guanosine[c]'
'RNA-3prime-Guanosine-3prime-P[c]'
'5Prime-OH-Terminated-RNAs[c]'
'G-5-prime-PP-5-prime-DNA[c]'
'3-KETOACYL-COA[c]'
'CPD-9872[c]'
'CPD-9870[c]'
'RNA-Ligase-L-lysine[c]'
'L-3-HYDROXYACYL-COA[c]'
'RNA-Ligase-L-lysine-adenylate[c]'
'PORPHOBILINOGEN[c]'
'5-Phospho-RNA[c]'
'HYDROXYMETHYLBILANE[c]'
'A-5-prime-PP-5-prime-RNA[c]'
'RNA-Holder[c]'
'3Prime-OH-Terminated-RNAs[c]'
'DATP[c]'
'ERYTHROSE-4P[c]'
'3-DEOXY-D-ARABINO-HEPTULOSONATE-7-P[c]'
'Oxidized-Flavoproteins[c]'
'Reduced-Flavoproteins[c]'
'ALPHA-GLUCOSE-16-BISPHOSPHATE[c]'
'CPD-8614[c]'
'CPD-3483[c]'
'CPD-3481[c]'
'GLC-D-LACTONE[e]'
'Glucopyranose[e]'
'CPD-4184[c]'
'CPD-1826[c]'
'2-DEHYDROPANTOATE[c]'
'L-PANTOATE[c]'
'HMP[c]'
'CROTONATE[c]'
'Dodec-2-enoyl-ACPs[c]'
'Thiocarboxyadenylated-ThiS-Proteins[c]'
'CPD-13575[c]'
'ACETOIN[c]'
'L-RHAMNOFURANOSE[c]'
'DIACETYL[c]'
'TARTRATE[e]'
'THIAMINE[e]'
'Triacylglycerides[e]'
'UBIQUINONE-6[e]'
'UBIQUINONE-8[e]'
'URACIL[e]'
'CPD-10353[c]'
'UREA[e]'
'L-XYLULOSE[c]'
'URIDINE[e]'
'CPD-13357[c]'
'VALERATE[e]'
'CPD-7953[c]'
'CPD-7952[c]'
'CPD-7951[c]'
'STRICTOSIDINE-AGLYCONE[c]'
'CPD-21552[c]'
'GEISSOSCHIZINE[c]'
'POLYNEURIDINE-ALDEHYDE[c]'
'CPD-7117[c]'
'CPD-7139[c]'
'HIF-alpha-subunit-L-asparagines[c]'
'HIF-alpha-subunit-3S-OH-ASN[c]'
'CPD-8815[c]'
'CPD-15895[c]'
'4-METHYLCATECHOL[c]'
'CPD-12288[c]'
'CPD-10664[c]'
'Glucosyl-acyl-sphinganines[c]'
'Glucosyl-acyl-sphingosines[c]'
'R-6-HYDROXYNICOTINE[c]'
'3-Prime-Phosphate-Terminated-RNAs[c]'
'CPD-14407[c]'
'CPD-8120[c]'
'CPD0-2350[c]'
'Pre-tRNA-5-prime-half-molecules[c]'
'Pre-tRNA-3-prime-half-molecules[c]'
'CPD-17794[c]'
'CPD-17791[c]'
'CPD-14422[c]'
'CPD-14423[c]'
'CPD-14424[c]'
'CPD-17793[c]'
'Protein-Ser-or-Thr-phosphate[c]'
'Protein-L-serine-or-L-threonine[c]'
'CPD-14425[c]'
'CPD-14426[c]'
'CPD-13328[c]'
'SCOPOLETIN[c]'
'CPD-15656[c]'
'CPD-3041[c]'
'CPD-15657[c]'
'PYRIDOXAL[e]'
'CPD-3061[c]'
'Methylated-DNA-Bases[c]'
'CPD-15675[c]'
'MALONATE[c]'
'CPD-15651[c]'
'CPD-15652[c]'
'BUTANOL[c]'
'CPD-13346[c]'
'CPD-15677[c]'
'CPD-15676[c]'
'AMINO-HYDROXYMETHYL-METHYL-PYR-P[c]'
'DNA-3-methyladenines[c]'
'DNA-containing-aPurinic-Sites[c]'
'3-Methyl-Adenines[c]'
'QUINOLINATE[e]'
'CPD-57[c]'
'RNA-with-3-prime-pp-5-prime-A-cap[c]'
'Cyclic-Phosphate-Terminated-RNAs[c]'
'RNA-3-prime-P-cyclase-L-histidine[c]'
'RNA-3-prime-P-cyclase-L-His-adenylate[c]'
'Nucleosides[c]'
'Ribonucleosides[c]'
'CPD-8617[c]'
'CPD-8618[c]'
'N-Acylsphingosine[c]'
'CPD-15530[c]'
'XANTHINE[e]'
'Xylans[e]'
'XYLITOL[e]'
'Amino-Acids-20[e]'
'Nucleosides[e]'
'2-HYDROXY-2-METHYLPROPANENITRILE[c]'
'ACETONE[c]'
'1-KETO-2-METHYLVALERATE[c]'
'BETAINE\_ALDEHYDE[c]'
'S-2-Haloacids[c]'
'PALMITALDEHYDE[c]'
'CPD-292[c]'
'CPD-5164[c]'
'Thi-S[c]'
'DEOXYXYLULOSE-5P[c]'
'CPD-17883[c]'
'CPD-5165[c]'
'CPD-17884[c]'
'CPD-5166[c]'
'D-ALA-D-ALA[c]'
'CPD-17885[c]'
'CPD-5167[c]'
'S-ADENOSYL-4-METHYLTHIO-2-OXOBUTANOATE[c]'
'CPD-17799[c]'
'5-AMINOPENTANOATE[c]'
'CPD-17787[c]'
'CPD-17800[c]'
'CPD-17887[c]'
'CPD-17801[c]'
'CPD-12303[c]'
'UDP-N-ACETYL-D-GLUCOSAMINE[c]'
'CPD-12304[c]'
'CPD-394[c]'
'CPD-17802[c]'
'CPD-7993[c]'
'CPD-12258[c]'
'CPD-9646[c]'
'CPD-17805[c]'
'CPD-12311[c]'
'CPD-7994[c]'
'CPD-12310[c]'
'CPD-17888[c]'
'Z-11-TETRADECENOYL-COA[c]'
'NICOTINE[c]'
'CPD-2748[c]'
'CPD-5168[c]'
'CPD-7535[c]'
'CPD-17807[c]'
'CPD-17809[c]'
'CPD-7526[c]'
'COUMARIN[c]'
'DIHYDROCOUMARIN[c]'
'Uridine44-in-tRNA-Ser[c]'
'2-O-Methyluridine44-tRNASer[c]'
'Guanine10-in-tRNA[c]'
'tRNA-Containing-N2-Methylguanine-10[c]'
'Guanine26-in-tRNA[c]'
'tRNA-Containing-N2-Methylguanine-26[c]'
'tRNA-Containing-N2-dimethylguanine-26[c]'
'CPD-17858[c]'
'CPD-17894[c]'
'GDP[c]'
'L-CANALINE[c]'
'UREA[c]'
'CANAVANINE[c]'
'CPD-15661[c]'
'CPD-10825[c]'
'CPD-10826[c]'
'CPD-10832[c]'
'CPD-15662[c]'
'CPD-15678[c]'
'CPD-15663[c]'
'CPD-9407[c]'
'CPD-15658[c]'
'CPD0-1308[c]'
'CPD0-1074[c]'
'CPD-13792[c]'
'Short-Chain-Trans-23-Dehydroacyl-CoA[c]'
'Short-Chain-234-Saturated-acyl-CoAs[c]'
'Very-Long-Chain-Trans-23-Dehydroacyl-CoA[c]'
'CPD-19268[c]'
'Very-long-Chain-234-Saturated-acyl-CoAs[c]'
'12-DEHYDROTETRACYCLINE[c]'
'CPD-19274[c]'
'CPD-19272[c]'
'VAL-tRNAs[c]'
'Charged-VAL-tRNAs[c]'
'BETAINE[c]'
'DICARBOXYLIC-ACID-MONOAMIDES[c]'
'L-CITRULLINE[c]'
'CPD-19474[c]'
'Pyruvate-Dehydrogenase-Phosphoserine[c]'
'Pyruvate-dehydrogenase-L-serine[c]'
'CPD-535[c]'
'CPD-19486[c]'
'FRUCTOSE-2-PHOSPHATE[c]'
'CPD-22266[c]'
'HYDROXYBENZOQUINONE[c]'
'CPD-22267[c]'
'Methyl-esterified-homogalacturonan[c]'
'1-4-alpha-D-galacturonosyl[c]'
'CPD-19487[c]'
'TRYPANOTHIONE-DISULFIDE[c]'
'TRYPANOTHIONE[c]'
'Dihydro-Lipoyl-Proteins[c]'
'Lipoyl-Protein-N6-lipoyllysine[c]'
'D-GLT[c]'
'D-Amino-Acids[c]'
'N-ACETYL-D-AMINO-ACID[c]'
'CPD-7418[c]'
'CPD-7417[c]'
'3-Hydroxy-octanoyl-ACPs[c]'
'3-Oxo-octanoyl-ACPs[c]'
'PHYTOSPINGOSINE[c]'
'CPD-15382[e]'
'tRNAPhe-Containing-4-demethylwyosine-37[c]'
'D-GALACTONO-1-4-LACTONE[e]'
'ALPHA-D-GALACTOSE[e]'
'yW-86[c]'
'Octadec-2-enoyl-ACPs[c]'
'GLUCOSAMINE[e]'
'yW-58[c]'
'tRNAPhe-wybutosine[c]'
'yW-72[c]'
'CPD-15438[e]'
'n-Alkanals[c]'
'MANNITOL[e]'
'Alk-2-enals[c]'
'2-HEXAPRENYL-6-METHOXY-14-BENZOQUINOL[c]'
'MANNOSE[e]'
'2-HEXAPRENYL-3-METHYL-6-METHOXY-14-BENZ[c]'
'OHyWstar-tRNA[c]'
'OHyW-58-tRNAPhe[c]'
'HYDANTOIN[c]'
'N-CARBAMOYLGLYCINE[c]'
'BUTANEDIOL[c]'
'CPD-111[c]'
'CPD-8782[c]'
'CPD-8781[c]'
'SULFO-CYSTEINE[c]'
'GDP-4-DEHYDRO-6-DEOXY-D-MANNOSE[c]'
'CPD-11281[c]'
'421-DEHYDROGEISSOSCHIZINE[c]'
'N-5S-5-AMINO-5-CARBOXYPENTANOYL-L-CY[c]'
'Apo-Propionyl-CoA-CO2-ligases[c]'
'Propionyl-CoA-CO2-ligases[c]'
'3-methylcrotonoyl-CoA-carboxylase-lysine[c]'
'Biotin-EC6-4-1-4[c]'
'CPD-14280[c]'
'CPD-10279[c]'
'CPD-14281[c]'
'CPD-10280[c]'
'CPD-14282[c]'
'NMNH[c]'
'CPD0-881[c]'
'CPD1G-277[c]'
'CPD-14283[c]'
'CPDQT-520[c]'
'CPD-2183[c]'
'CPD-14300[c]'
'CANAVANINOSUCCINATE[c]'
'CPD-479[c]'
'2-2-METHYLTHIOETHYLMALIC-ACID[c]'
'3-2-METHYLTHIOETHYLMALIC-ACID[c]'
'HOMOMETHIONINE[c]'
'STEARIC\_ACID[c]'
'N-Ac-L-methionyl-L-tyrosinyl-Protein[c]'
'N-Ac-N-terminal-L-valine[c]'
'N-terminal-L-valine[c]'
'N-terminal-L-alanine[c]'
'N-terminal-N-Ac-L-alanine[c]'
'16-HYDROXYPALMITATE[c]'
'N-terminal-L-cysteine[c]'
'N-terminal-N-Ac-L-cysteine[c]'
'CPD-9406[c]'
'CPD-17635[c]'
'N-terminal-glycine[c]'
'N-terminal-N-Ac-glycine[c]'
'N-terminal-N-Ac-L-Serine[c]'
'N-terminal-L-Serine[c]'
'CoM[c]'
'2-OXOPROPYL-COM[c]'
'PRENAL[c]'
'S-PRENYL-L-CYSTEINE[c]'
'Protein-L-methionine-R-S-oxides[c]'
'Protein-L-methionine[c]'
'CPD-8989[c]'
'RIBOSE[e]'
'D-Xylose[e]'
'CPD-16017[e]'
'ETOH[e]'
'CPD-320[e]'
'FERROCYTOCHROME-B5[e]'
'FMN[e]'
'Folates[e]'
'FORMATE[e]'
'OHyW-tRNAPhe[c]'
'ARG[c]'
'CPD-7419[c]'
'CPD-15413[c]'
'25S-rRNA-adenine-2142[c]'
'25S-rRNA-N1-methyladenine-2142[c]'
'CPD-7422[c]'
'25S-rRNA-adenine-645[c]'
'25S-rRNA-N1-methyladenine-645[c]'
'S-palmitoyl-L-cysteine-in-proteins[c]'
'CPD-7554[c]'
'PROT-CYS[c]'
'CPD-7556[c]'
'2-Octenoyl-ACPs[c]'
'apo-Transcarboxylases[c]'
'CPD-355[c]'
'CPD-569[c]'
'L-aspartyl-tRNAAsn[c]'
'Octanoyl-ACPs[c]'
'Charged-ASN-tRNAs[c]'
'CPD-356[c]'
'L-glutamyl-tRNAGln[c]'
'CPD-220[c]'
'CPD-13174[c]'
'CPD-13172[c]'
'5-HYDROXY-FERULOYL-COA[c]'
'4-SULFOBENZALDEHYDE[c]'
'CPD-257[c]'
'CPD-12180[c]'
'CPD-6602[c]'
'O-Long-Chain-Acyl-L-Carnitines[c]'
'SINAPOYL-COA[c]'
'3-HYDROXY-CISCIS-MUCONATE[c]'
'18S-rRNA-pseudouridine-1191[c]'
'3-HEXAPRENYL-4-HYDROXYBENZOATE[c]'
'18S-rRNA-N1-methylpseudouridine-1191[c]'
'CPD-21768[c]'
'VLC-Alpha-hydroxyphytoceramides[c]'
'IPC[c]'
'VLC-MIPC[c]'
'CPD-14305[c]'
'DI-H-OROTATE[c]'
'CPD-21769[c]'
'CPD-21770[c]'
'CPD-14304[c]'
'2-HEXAPRENYL-3-METHYL-5-HYDROXY-6-METHOX[c]'
'CPD-1108[c]'
'CPD-633[c]'
'3-METHYLTHIOPROPANALDOXIME[c]'
'CPD-7546[c]'
'3-METHYLTHIOPROPYLHYDROXAMIC-ACID[c]'
'3-METHYLTHIOPROPYL-DESULFO-GLUCOSINOLATE[c]'
'3-METHYLTHIOPROPYL-GLUCOSINOLATE[c]'
'3-METHYLSULFINYLPROPYL-GLUCOSINOLATE[c]'
'2-PROPENYL-GLUCOSINOLATE[c]'
'CPD-8990[c]'
'FUM[e]'
'4-AMINO-BUTYRATE[e]'
'Glucose[e]'
'GLUTATHIONE[e]'
'GLYCEROL[e]'
'Xylans[c]'
'GLY[e]'
'P-BENZOQUINONE[c]'
'Single-Stranded-DNAs[c]'
'Ribonucleoside-Triphosphates[c]'
'Glycogens[e]'
'ssDNA-RNA-primer-hybrid[c]'
'GLYCOLLATE[e]'
'GUANINE[e]'
'APS[c]'
'CAMP[c]'
'CPD-7830[e]'
'L-ARABITOL[c]'
'CPD-1117[c]'
'CPD-1134[c]'
'O-UREIDOHOMOSERINE[c]'
'CPD-7652[c]'
'DIHYDROPTERIN-CH2OH-PP[c]'
'CPD-10766[c]'
'Protein-N-terminal-N-Ac-L-threonine[c]'
'Protein-N-terminal-L-threonine[c]'
'N-terminal-L-Serine-Histone-H2A[c]'
'N-terminal-N-Ac-L-Serine-Histone-2A[c]'
'Nucleotides[c]'
'N-terminal-L-Serine-Histone-H4[c]'
'N-terminal-N-Ac-L-Serine-Histone-4[c]'
'L-methionyl-L-lysyl-Protein[c]'
'N-Ac-L-methionyl-L-lysyl-Protein[c]'
'L-methionyl-L-valyl-Protein[c]'
'N-Ac-L-methionyl-L-valyl-Protein[c]'
'L-methionyl-L-alanyl-Protein[c]'
'N-Ac-L-methionyl-L-alanyl-Protein[c]'
'Nucleoside-Triphosphates[c]'
'FRUCTOSE-16-DIPHOSPHATE[c]'
'CPD-17640[c]'
'CPD-656[c]'
'CPD-17370[c]'
'CPD-7557[c]'
'CPD-7558[c]'
'CPD-15435[c]'
'N6-L-threonylcarbamoyladenine37-tRNAs[c]'
'tRNA-adenine-37[c]'
'CPD-15438[c]'
'L-rhamnopyranose[c]'
'CPD-15440[c]'
'CPD-15436[c]'
'Butanoyl-ACPs[c]'
'3-oxo-decanoyl-ACPs[c]'
'SULFOACETALDEHYDE[c]'
'CPD-10794[c]'
'3-HYDROXYADIPYL-COA[c]'
'TRANS-23-DEHYDROADIPYL-COA[c]'
'5-METHYLTHIOINOSINE[c]'
'1-PHOSPHATIDYL-1D-MYO-INOSITOL-34-BISPH[c]'
'2-PG[c]'
'CHOCOLA\_A[c]'
'CPD-13524[c]'
'CPD-13175[c]'
'MET[e]'
'CPD-452[c]'
'SALICYLALDEHYDE[c]'
'CPD-1113[c]'
'ERGOSTEROL[c]'
'CPD-11602[c]'
'CPD-882[c]'
'CPD-881[c]'
'CPD-14355[c]'
'CPD-160[c]'
'CPD-13955[c]'
'CPD-12199[c]'
'CPD-7619[e]'
'HYPOXANTHINE[e]'
'ARACHIDIC\_ACID[e]'
'Inulin[e]'
'THREO-DS-ISO-CITRATE[e]'
'CPD-7676[c]'
'CPD-468[e]'
'Alpha-lactose[e]'
'CPD-15972[e]'
'L-ALPHA-ALANINE[e]'
'L-ARABITOL[e]'
'ARG[e]'
'L-methionyl-L-seryl-Protein[c]'
'N-Ac-L-methionyl-L-seryl-Protein[c]'
'L-methionyl-L-threonyl-Protein[c]'
'N-Ac-L-methionyl-L-threonyl-Protein[c]'
'Aminopeptidase-Substrates[c]'
'L-methionyl-L-cysteinyl-Protein[c]'
'L-methionyl-glycyl-Protein[c]'
'CPD-8198[c]'
'IMINOASPARTATE[c]'
'L-Fucopyranoses[c]'
'METHYL-BETA-D-GALACTOSIDE[c]'
'CPD-730[c]'
'CPD-729[c]'
'1-3-beta-D-Glucans[c]'
'CPD-17642[c]'
'CPD-17644[c]'
'CPD-17641[c]'
'PHOSPHORIBOSYL-ATP[c]'
'CPD-17643[c]'
'CPD-17638[c]'
'CPD-17647[c]'
'CPD-9570[c]'
'CPD-357[c]'
'METHYL-GLYOXAL[c]'
'ASN[e]'
'CPD-7545[c]'
'L-ASPARTATE[e]'
'CPD1F-135[c]'
'CPD-15414[c]'
'DODECANOATE[e]'
'CPD-7424[c]'
'CPD-15423[c]'
'L-CITRULLINE[e]'
'CYS[e]'
'CPD-15502[c]'
'GLT[e]'
'CPD-15467[c]'
'GLN[e]'
'HIS[e]'
'INDOLE\_PYRUVATE[c]'
'TRP[c]'
'HOMO-CYS[e]'
'CPD-15522[c]'
'CPD-369[e]'
'CPD-15521[c]'
'CPD-7618[c]'
'CHOLANATE2[c]'
'LIPOIC-ACID[e]'
'CPD-10797[c]'
'CPD-10796[c]'
'CPD-15554[c]'
'CPD-335[c]'
'CPD-10781[c]'
'3-OXODODECANOATE[c]'
'TAGATOSE-1-6-DIPHOSPHATE[c]'
'CPD-7706[c]'
'O-Acyl-L-Carnitines[e]'
'O-Acyl-L-Carnitines[c]'
'Beta-hydroxydecanoyl-ACPs[c]'
'CPD-8606[c]'
'CPD-184[c]'
'4-OXALOMESACONATE[c]'
'Acetylxylan[c]'
'Feruloyl-polysaccharides[c]'
'4-OH-4-ACETYL-2-OXOGLUTARATE[c]'
'CPD-10600[c]'
'FERULIC-ACID[c]'
'CPD-13181[c]'
'CPD-13182[c]'
'CPD-3571[c]'
'HOMO-SER[c]'
'Enones[c]'
'DMPBQ[c]'
'CPD-13187[c]'
'CPD-13188[c]'
'16-EPIVELLOSIMINE[c]'
'MPBQ[c]'
'CPD-665[c]'
'DELTA-TOCOPHEROL[c]'
'CPD0-1470[c]'
'CPD-19217[c]'
'CPD-1763[c]'
'S-NITROSOGLUTATHIONE[c]'
'CPD-13617[c]'
'Charged-ARG-tRNAs[c]'
'L-Glutamyl-Peptides[c]'
'CPD-7671[c]'
'Lipoyl-Protein-L-Lysine[c]'
'CPD-195[c]'
'Octanoylated-domains[c]'
'N-ETHYLMALEIMIDE[c]'
'CPD0-903[c]'
'ILE[e]'
'LEU[e]'
'5-KETO-4-DEOXY-D-GLUCARATE[c]'
'D-GLUCARATE[c]'
'LYS[e]'
'Very-long-chain-fatty-acids[c]'
'L-ORNITHINE[e]'
'CPD-17624[c]'
'PHE[e]'
'CPD-15382[c]'
'PRO[e]'
'L-RIBULOSE[e]'
'OCTADEC-9-ENE-118-DIOIC-ACID[c]'
'SER[e]'
'Phosphoacetylglucosamine-Mutase[c]'
'Phosphoacetylglucosamine-Mutase-P[c]'
'N-ACETYL-D-GLUCOSAMINE-16-BIS-P[c]'
'CPD-9570[e]'
'THR[e]'
'CPD-17714[c]'
'CPD-17701[c]'
'CPD-8607[c]'
'CPD-8608[c]'
'ALPHA-HYDROXY-915-DIOXOPROSTANOATE[c]'
'HYDROXY-915-DIOXOPROSTA-13-ENOATE[c]'
'CPD-308[c]'
'CPD-15524[c]'
'CPD-15523[c]'
'CPD-33[c]'
'DEHYDFUC-CPD[c]'
'CPD-15566[c]'
'TAGATOSE-6-PHOSPHATE[c]'
'CPD-1118[c]'
'CPD-183[c]'
'TARTRATE[c]'
'CPD-66[c]'
'CPD-7620[c]'
'CPD-7619[c]'
'CPD-10799[c]'
'CPD-10782[c]'
'THIOHYDROXIMATE-O-SULFATES[c]'
'Nitriles[c]'
'CPD-558[c]'
'Alkenyl-Thiohydroximate-O-Sulfates[c]'
'EPITHIONITRILES[c]'
'CPD-10800[c]'
'CDPDIACYLGLYCEROL[c]'
'L-1-PHOSPHATIDYL-GLYCEROL[c]'
'CARDIOLIPIN[c]'
'CPD-10801[c]'
'S-RETICULINE[c]'
'12-DEHYDRORETICULINIUM[c]'
'ACROLEIN[c]'
'CPD-7600[c]'
'CPD-8847[c]'
'MEK[c]'
'CPD-13218[c]'
'CPD-13220[c]'
'CPD-13222[c]'
'TRP[e]'
'Acyl-homoserine-lactones[c]'
'Acyl-homoserines[c]'
'TYR[e]'
'VAL[e]'
'CPD-12364[e]'
'Deoxy-Ribonucleoside-Monophosphates[c]'
'Maltodextrins[e]'
'MALTOSE[e]'
'MALTOTRIOSE[e]'
'BETA-TOCOPHEROL[c]'
'CONIFERYL-ALCOHOL[c]'
'CONIFERYL-ALDEHYDE[c]'
'SINAPYL-ALCOHOL[c]'
'CPD-12829[c]'
'L-arginyl-L-Glutamyl-Peptides[c]'
'ARG-tRNAs[c]'
'Proteins-With-N-Terminal-Asp[c]'
'L-arginyl-L-aspartyl-Peptides[c]'
'TTP[c]'
'L-arginyl-3-sulfino-L-alaninyl-Peptides[c]'
'N-terminal-L-cysteine-sulfinate[c]'
'N-terminal-L-cysteine-sulfonate[c]'
'SER[c]'
'L-arginyl-3-sulfo-L-alaninyl-Peptides[c]'
'N-Ac-L-methionyl-L-asparaginyl-Protein[c]'
'N-terminal-asparagine[c]'
'CPD0-2015[c]'
'L-Glutaminyl-Peptides[c]'
'L-RIBULOSE-5-P[c]'
'N-Ac-L-methionyl-L-glutaminyl-Protein[c]'
'Trans-D2-decenoyl-ACPs[c]'
'L-RIBULOSE[c]'
'UROPORPHYRINOGEN-III[c]'
'DIMETHYL-GLYCINE[c]'
'5-DEHYDROGLUCONATE[c]'
'CPD-13473[c]'
'CPD-17723[c]'
'CPD-17724[c]'
'CPD-17725[c]'
'CPD-8609[c]'
'CPD-8610[c]'
'O-SUCCINYL-L-HOMOSERINE[c]'
'L-CYSTATHIONINE[c]'
'CPD-3187[c]'
'CPD-18798[c]'
'CPD-14092[c]'
'CPD-6972[c]'
'CPD-22025[c]'
'CPD-22027[c]'
'CPD-22028[c]'
'CPD-15189[c]'
'CPD-22029[c]'
'VLC-Ceramides[c]'
'CPD-10556[c]'
'Ultra-Long-Chain-Acyl-CoAs[c]'
'ULC-Cermaides[c]'
'CPD-13612[c]'
'CPD-22033[c]'
'CPD-18797[c]'
'3-OXO-5-BETA-CHOLANATE[c]'
'57222428-ERGOSTATETRAENOL[c]'
'CPD-18825[c]'
'ISO-PROPANOL[c]'
'SACCHAROPINE[c]'
'SECOLOGANIN-CPD[c]'
'CPD-7247[c]'
'ISOVALERYL-COA[c]'
'BCAA-dehydrogenase-3MB-DH-lipoyl[c]'
'CPD-18826[c]'
'CPD-18831[c]'
'CPD-18832[c]'
'CPD-12524[c]'
'CPD-17138[c]'
'CPD-9038[c]'
'Cytochromes-C-Reduced[e]'
'CPD-4211[c]'
'CPD-15265[c]'
'Cytochromes-C-Oxidized[e]'
'CPD-15263[c]'
'TREHALOSE[c]'
'CPD-1862[c]'
'HOP-2229-ENE[c]'
'CPD-15268[c]'
'ENT-COPALYL-DIPHOSPHATE[c]'
'CPD-15260[c]'
'CPD-602[c]'
'CPD-15259[c]'
'CPD-15261[c]'
'Ergothioneine[c]'
'CPD-19154[c]'
'CPD-19161[c]'
'CPD-11571[c]'
'CPD-19157[c]'
'CPD-12101[c]'
'CPD-19148[c]'
'3-oxo-hexanoyl-ACPs[c]'
'12-apo-Carotenals[c]'
'9-cis-Epoxycarotenoids[c]'
'CPD-7279[c]'
'CPD-7280[c]'
'CPD-7196[c]'
'3-OXOADIPATE-ENOL-LACTONE[c]'
'3-KETO-ADIPATE[c]'
'CPD1F-130[c]'
'R-3-hydroxyhexanoyl-ACPs[c]'
'CPD1F-131[c]'
'D-GALACTONO-1-4-LACTONE[c]'
'CPD-155[c]'
'GALACTOSE-1P[c]'
'N-SUCCINYL-2-AMINO-6-KETOPIMELATE[c]'
'N2-SUCCINYLORNITHINE[c]'
'CPD-822[c]'
'CPD-725[c]'
'L-methionyl-tRNAfmet[c]'
'NN-dimethyl-terminal-XPK[c]'
'NNN-trimethyl-terminal-XPK[c]'
'CPD-17434[c]'
'CYTIDINE[c]'
'D-Ribofuranose[c]'
'N-terminal-XPK[c]'
'CYTOSINE[c]'
'N-terminal-PPK[c]'
'NN-dimethyl-terminal-PPK[c]'
'CPD-17428[c]'
'N-methyl-terminal-PPK[c]'
'CPD-17052[c]'
'CPD-17453[c]'
'CPD66-40[c]'
'CPD-22034[c]'
'CPD-22035[c]'
'FECOSTEROL[c]'
'CPD-9965[c]'
'CPD-22036[c]'
'CPD-16352[c]'
'URIDINE[c]'
'CPD1F-133[c]'
'HISTIDINOL[c]'
'HIS[c]'
'CPD-7390[c]'
'CPD-7392[c]'
'Deoxy-Ribonucleoside-Diphosphates[c]'
'Ribonucleoside-Diphosphates[c]'
'COPROPORPHYRINOGEN\_III[c]'
'Oxidized-NrdH-Proteins[c]'
'Reduced-NrdH-Proteins[c]'
'CPD-205[c]'
'CPD-101[c]'
'CPD-15285[c]'
'CPD0-2298[c]'
'CPD-1091[c]'
'CPD-1863[c]'
'CPD-1881[c]'
'CU+[c]'
'CU+[e]'
'L-4-HYDROXYGLUTAMATE\_SEMIALDEHYDE[c]'
'3-DEHYDRO-SHIKIMATE[c]'
'UDP-D-GALACTO-14-FURANOSE[c]'
'L-Amino-Acids[c]'
'5-OXOPROLINE[c]'
'5-L-GLUTAMYL-PEPTIDE[c]'
'5-L-GLUTAMYL-AMINO-ACID[c]'
'Hex-2-enoyl-ACPs[c]'
'CPD-17455[c]'
'CPD-19150[c]'
'CPD-17053[c]'
'CPD-19151[c]'
'CPD-17457[c]'
'CPD-3631[c]'
'CPD-19153[c]'
'CPD-17458[c]'
'CPD-3944[c]'
'CPD-3942[c]'
'CPD-8678[c]'
'BETA-D-FRUCTOSE[c]'
'3-SULFINYL-PYRUVATE[c]'
'Kanamycin-3-phosphates[c]'
'Kanamycins[c]'
'DE-O-GLUCONATE[c]'
'DE-O-K-GLUCONATE[c]'
'CPD-16353[c]'
'CPD-22039[c]'
'CPD-14293[c]'
'CPD-22040[c]'
'D-3-HYDROXYACYL-COA[c]'
'CPD-22041[c]'
'CPD-14261[c]'
'CPD-10662[c]'
'CPD-14269[c]'
'CPD-14262[c]'
'CPD-22043[c]'
'CPD-15363[c]'
'3-oxo-stearoyl-ACPs[c]'
'HOMO-CIT[c]'
'HOMO-CIS-ACONITATE[c]'
'EPISTEROL[c]'
'CPD-307[c]'
'THIOMORPHOLINE-3-CARBOXYLATE[c]'
'34-DEHYDRO-14-THIOMORPHOLINE-3-CARBOXY[c]'
'R-RETICULINE[c]'
'CPD-2022[c]'
'CPD-102[c]'
'L-ERYTHRO-4-HYDROXY-GLUTAMATE[c]'
'CPD-637[c]'
'CPD-112[c]'
'CPD-15978[c]'
'CPD-15361[c]'
'L-LACTATE[e]'
'CPD-1072[c]'
'MAL[e]'
'1-3-beta-D-Glucans[e]'
'CPD-9446[e]'
'2-KETOGLUTARATE[e]'
'P-AMINO-BENZOATE[e]'
'CPD-1302[e]'
'6-DEMETHYLSTERIGMATOCYSTIN[e]'
'Red-Thioredoxin[e]'
'ACP[e]'
'SHIKIMATE[c]'
'CPDQT-273[c]'
'CPD-12140[c]'
'CPD1G-2[c]'
'CPD-12152[c]'
'CPD-12156[c]'
'NICOTINAMIDE\_RIBOSE[c]'
'Pyrimidine-Nucleosides[c]'
'Pyrimidine-Bases[c]'
'CPD-17614[c]'
'Vernolates[c]'
'2-Acylglycero-Phosphocholines[c]'
'NARINGIN[c]'
'NARINGENIN-7-O-BETA-D-GLUCOSIDE[c]'
'CPD-7075[c]'
'HYPOTAURINE[c]'
'CPD-7073[c]'
'FRU1P[c]'
'BCAA-dehydrogenase-lipoyl[c]'
'5-PHOSPHO-RIBOSYL-GLYCINEAMIDE[c]'
'5-P-RIBOSYL-N-FORMYLGLYCINEAMIDE[c]'
'CPD-14925[c]'
'CPD-10793[c]'
'3-P-HYDROXYPYRUVATE[c]'
'Hexanoyl-ACPs[c]'
'CPD-15364[c]'
'CPD-22044[c]'
'CPD-14268[c]'
'CPD-22045[c]'
'CPD-22048[c]'
'CPD-14271[c]'
'CPD-22050[c]'
'CPD-10283[c]'
'CPD-18491[c]'
'CPD-782[c]'
'Nucleoside-Diphosphates[c]'
'UDP-SULFOQUINOVOSE[c]'
'CPD-13171[c]'
'apo-Peptidyl-carrier-proteins[c]'
'ACET[e]'
'L-2-AMINOPENTANOIC-ACID[c]'
'ADENINE[e]'
'ADENOSINE[e]'
'TREHALOSE[e]'
'CPD-15699[e]'
'LINOLENIC\_ACID[e]'
'ETR-Quinols[e]'
'ETF-Oxidized[e]'
'ANTHRANILATE[e]'
'CPD-7400[c]'
'CYANURIC-ACID[c]'
'GLUTAMYL-GLX-TRNAS[c]'
'CPD-700[c]'
'GLX-tRNAs[c]'
'CPD-1075[c]'
'CPD-7409[c]'
'Phytosphingosines[c]'
'Very-Long-Chain-Phytoceramides[c]'
'CPD-15362[c]'
'CPD-17399[c]'
'CPD-17400[c]'
'CPD-15368[c]'
'CPD-3736[c]'
'Ferrihemoglobins[c]'
'Ferrohemoglobins[c]'
'CPD-19144[c]'
'CPD-19170[c]'
'AMINOMETHYLDIHYDROLIPOYL-GCVH[c]'
'Deoxy-Ribonucleoside-Triphosphates[c]'
'DIHYDROLIPOYL-GCVH[c]'
'3-KETOLACTOSE[c]'
'CPD-17496[c]'
'CPD-1242[c]'
'P-NITROPHENOL[c]'
'CPD-9000[c]'
'CPD-14274[c]'
'CPD-14273[c]'
'CPD-13381[c]'
'CPD-14275[c]'
'CPD-14736[c]'
'CPD-19168[c]'
'ANTHRANILATE[c]'
'MAP-Kinase-L-Tyr[c]'
'MAP-Kinase-L-Phosphotyrosine[c]'
'CPD-14276[c]'
'3-HYDROXY-L-KYNURENINE[c]'
'CPD-14277[c]'
'CELLULOSE[e]'
'CPD-321[c]'
'CPD-14133[c]'
'CPD-4462[c]'
'CPD-22265[c]'
'1-Acyl-sn-glycerols[c]'
'CPD-19186[c]'
'Protein-S-methyl-L-cysteine[c]'
'a-thymine-in-DNA[c]'
'CPD0-2500[c]'
'Protein-Red-Disulfides[c]'
'L-methionyl-L-asparaginyl-Protein[c]'
'Protein-Ox-Disulfides[c]'
'L-methionyl-L-glutaminyl-Protein[c]'
'N-Ac-L-methionyl-L-aspartyl-Protein[c]'
'Alkyl-acetyl-glycero-phosphocholines[c]'
'1-Alkyl-sn-glycero-3-phosphocholines[c]'
'CPD-19167[c]'
'CPD-1092[c]'
'3-OXOPIMELOYL-COA[c]'
'CPD-459[c]'
'Phytoceramides[c]'
'Alpha-hydroxyphytoceramides[c]'
'CPD-15369[c]'
'CPD-15370[c]'
'HYDRPHENYLAC-CPD[c]'
'CPD-15366[c]'
'CPD-15373[c]'
'CPD-12601[c]'
'Guanine37-in-tRNAPhe[c]'
'tRNAPhe-Containing-N1-Methylguanine-37[c]'
'3-UREIDO-ISOBUTYRATE[c]'
'CPD-471[c]'
'CPD-209[c]'
'C3[c]'
'DIHYDRO-THYMINE[c]'
'CH3-MALONATE-S-ALD[c]'
'CHITIN[e]'
'CPD-13545[e]'
'Chitosan[e]'
'CL-[e]'
'CHOLINE[e]'
'CARBON-DIOXIDE[e]'
'CPD-69[e]'
'CPD-355[e]'
'CPD-3617[e]'
'BETA-D-FRUCTOSE[e]'
'CPD-182[c]'
'CPD-14278[c]'
'CPD-181[c]'
'L-methionyl-L-aspartyl-Protein[c]'
'N-Ac-L-methionyl-L-glutamyl-Protein[c]'
'CPD3DJ-82[c]'
'L-methionyl-L-glutamyl-Protein[c]'
'L-methionyl-L-leucyl-Protein[c]'
'N-Ac-L-methionyl-L-leucyl-Protein[c]'
'Sphinga-4E-8E-dienine-Ceramides[c]'
'D-BETA-D-HEPTOSE-17-DIPHOSPHATE[c]'
'9-Methyl-sphing-4-8-dienine-ceramides[c]'
'L-methionyl-L-isoleucyl-Protein[c]'
'N-Ac-L-methionyl-L-isoleucyl-Protein[c]'
'L-methionyl-L-phenylalanyl-Protein[c]'
'ADP-D-GLYCERO-D-MANNO-HEPTOSE[c]'
'N-Ac-L-methionyl-L-phenylalanyl-Protein[c]'
'Delta5-Delta7-Steroids[c]'
'L-methionyl-L-tryptophanyl-Protein[c]'
'N-Ac-L-methionyl-L-tryptophanyl-Protein[c]'
'Delta7-Steroids[c]'
'L-methionyl-L-tyrosinyl-Protein[c]'
'CPD0-2474[c]'
'2-D-THREO-HYDROXY-3-CARBOXY-ISOCAPROATE[c]'
'UNDECAPRENYL-DIPHOSPHATE[c]'
'Aliphatic-Nitriles[c]'
'Primary-Aliphatic-Amides[c]'
'CPD-19172[c]'
'N5-Formyl-THF-Glu-N[c]'
'CPD-19171[c]'
'2-AMINOMUCONATE\_SEMIALDEHYDE[c]'
'2-AMINO-MUCONATE[c]'
'CPD-19169[c]'
'CPD-444[c]'
'CPD-10172[c]'
'CPD-1061[c]'
'CPD-9451[c]'
'CPD-10175[c]'
'CPD-10175[e]'
'CPD-10171[c]'
'ASP-tRNAs[c]'
'CPD-10176[c]'
'Charged-ASP-tRNAs[c]'
'CPD-4205[c]'
'CPD-4586[c]'
'CPD-302[c]'
'CPD-17312[c]'
'CPD-21823[c]'
'TRANS-D2-ENOYL-COA[c]'
'CPD-21826[c]'
'Trans-3-enoyl-CoAs[c]'
'CPD-21825[c]'
'GAMMA-LINOLENOYL-COA[c]'
'CPD-21816[c]'
'CPD-21828[c]'
'CPD-21817[c]'
'CPD-21814[c]'
'Long-Chain-Fatty-Acids[c]'
'ARACHIDONYL-COA[c]'
'CPD-21830[c]'
'Sterols[c]'
'TAURINE[c]'
'Charged-CYS-tRNAs[c]'
'VANILLATE[c]'
'CPD-15192[c]'
'CPD-15216[c]'
'Sterol-3-beta-D-glucosides[c]'
'Long-chain-cholesterol-esters[c]'
'CPD-629[c]'
'CPD-556[c]'
'CPD-255[c]'
'CPD-17387[c]'
'CPD-17386[c]'
'CPD-17388[c]'
'CPD-14165[c]'
'CPD-14447[c]'
'4-FUMARYL-ACETOACETATE[c]'
'CPD-17355[c]'
'CPD-10244[c]'
'INDOLE[c]'
'MONOMETHYL-ESTER-OF-TRANS-ACONITATE[c]'
'Behenoyl-ACPs[c]'
'3-oxo-lignoceroyl-ACPs[c]'
'R-3-hydroxylignoceroyl-ACPs[c]'
'trans-delta2-lignoceroyl-ACPs[c]'
'4-HYDROXYPHENYLACETATE[c]'
'NICOTINATE\_NUCLEOTIDE[c]'
'CPD-110[c]'
'VANILLIN[c]'
'CPD-581[c]'
'DIMETHYLAMINE[c]'
'CPD-4587[c]'
'CPD-4588[c]'
'CPD-10177[c]'
'CPD-402[c]'
'N-METHYLANTHRANILOYL-COA[c]'
'Long-Chain-Steryl-Esters[c]'
'3-Phosphomonucleotides[c]'
'CPD-22005[c]'
'CPD-22004[c]'
'3-OCTAPRENYL-4-HYDROXYBENZOATE[c]'
'CPD-22006[c]'
'CPD-22003[c]'
'3-HYDROXYBENZOATE[c]'
'CPD-22008[c]'
'CPD-22009[c]'
'3-MERCAPTO-PYRUVATE[c]'
'IMP[c]'
'L-ASPARTATE-SEMIALDEHYDE[c]'
'L-BETA-ASPARTYL-P[c]'
'CPD-5881[c]'
'CPD-14202[c]'
'TETRADEHYDROACYL-COA[c]'
'2-hydroxyacyl-glutathiones[c]'
'2-Hydroxy-carboxylates[c]'
'CPD-8050[c]'
'CPD-14808[c]'
'CPD0-934[c]'
'CPD-15237[c]'
'Demethylmenaquinols[c]'
'Menaquinols[c]'
'DNA-containing-a-Apyrimidinic-Sites[c]'
'N-ACETYL-5-METHOXY-TRYPTAMINE[c]'
'5-HYDROXYISOURATE[c]'
'CPD-12014[c]'
'N-ACETYL-SEROTONIN[c]'
'CPD-45[c]'
'STIPIT-CPD[c]'
'STRICTOSIDINE[c]'
'TRYPTAMINE[c]'
'2-Phenyloxirane[c]'
'PHENYLACETALDEHYDE[c]'
'CPD-8900[c]'
'CPD-17282[c]'
'Protein-L-lysine[c]'
'CPD-17381[c]'
'44-DIMETHYL-5ALPHA-CHOLEST-7-EN-3BET[c]'
'CPD-17392[c]'
'CPD-5846[c]'
'CPD1F-132[c]'
'CPD1F-95[c]'
'CPD-692[c]'
'DNA-with-Uracils[c]'
'CPD-787[c]'
'CPD-786[c]'
'PHOSPHORYL-ETHANOLAMINE[c]'
'CPD3DJ-11366[c]'
'SPHINGOSINE[c]'
'ISOCHORISMATE[c]'
'CHORISMATE[c]'
'CPD-22010[c]'
'CPD-22007[c]'
'CPD-7214[c]'
'CPD-7087[c]'
'3S-CITRYL-COA[c]'
'CPD-22012[c]'
'CPD-22013[c]'
'CPD-7221[c]'
'CPD-7222[c]'
'Retinols[c]'
'CPD-22014[c]'
'A-3-OXO-ACID[c]'
'CPD-10189[c]'
'CPD-10188[c]'
'B-KETOACYL-ACP[c]'
'OH-ACYL-ACP[c]'
'CPD-19163[c]'
'CARBAMYUL-L-ASPARTATE[c]'
'CPD-19159[c]'
'CPD-216[c]'
'Protein-L-serines[c]'
'Protein-D-serines[c]'
'CYSTINE[c]'
'THIOCYSTEINE[c]'
'CPD-15240[c]'
'Alpha-6-alpha-14-glucans[c]'
'Sulfur-Carrier-Proteins-ThiI[c]'
'Sulfurylated-ThiI[c]'
'CPD-578[c]'
'N-SUCCINYLLL-2-6-DIAMINOPIMELATE[c]'
'LL-DIAMINOPIMELATE[c]'
'CPD-12015[c]'
'CPD-12017[c]'
'CPD1F-128[c]'
'CPD-17395[c]'
'BCAA-dehydrogenase-2MP-DH-lipoyl[c]'
'CPD-17390[c]'
'CPD-17389[c]'
'CPD-17391[c]'
'CPD-17393[c]'
'CPD-17394[c]'
'ISOPENICILLIN-N[c]'
'5-HYDROXY-TRYPTOPHAN[c]'
'SEROTONIN[c]'
'CPD-7224[c]'
'CPD-22011[c]'
'CAPSORUBIN[c]'
'CPD-22016[c]'
'CPD-22017[c]'
'CAPSANTHIN[c]'
'CPD-22018[c]'
'CPD-22015[c]'
'CPD-22021[c]'
'PENICILLIN-N[c]'
'D-SERINE[c]'
'S-3-HYDROXYBUTANOYL-COA[c]'
'CPD-650[c]'
'ADENOSYL-P4[c]'
'ADP-L-GLYCERO-D-MANNO-HEPTOSE[c]'
'DEOXYURIDINE[c]'
'CPD-19160[c]'
'Acetoacetyl-ACPs[c]'
'CPD-10269[c]'
'CPD-19162[c]'
'Beta-3-hydroxybutyryl-ACPs[c]'
'CPD-15244[c]'
'CPD-12016[c]'
'CPD-12259[c]'
'CPD-12230[c]'
'CPD-12018[c]'
'CPD-12019[c]'
'Aldehydes[c]'
'CPD-425[c]'
'Alkanesulfonates[c]'
'CPD-18346[c]'
'CPD-1107[c]'
'CPD-1772[c]'
'PROPANOL[c]'
'OH-PYR[c]'
'CPD-17396[c]'
'CPD-17397[c]'
...

-------------------------------------------------------------------------------

The chemical element **Ca** is present in the empirical formula of **2 metabolites**

'CA+2[e]'
'CA+2[c]'

-------------------------------------------------------------------------------

The chemical element **Cd** is present in the empirical formula of **2 metabolites**

'CD+2[c]'
'CD+2[e]'

-------------------------------------------------------------------------------

The chemical element **Cl** is present in the empirical formula of **83 metabolites**

'CPD-10663[c]'
'26-DICHLORO-P-HYDROQUINONE[c]'
'12-DICHLOROETHANE[c]'
'2-CHLOROETHANOL[c]'
'CL-[c]'
'CPD-10847[c]'
'CPD-10844[c]'
'CPD-10849[c]'
'CPD-10845[c]'
'CPD-9152[c]'
'CPD-10870[c]'
'CPD-10866[c]'
'CPD-9151[c]'
'CPD-10637[c]'
'CPD-10868[c]'
'CPD-15723[c]'
'CPD-10615[c]'
'CPD-15728[c]'
'24-DICHLOROPHENOL[c]'
'CPD-15[c]'
'245-DNOL[c]'
'25-DDOL[c]'
'TETRACHLOROHYDROQUINONE[c]'
'236-TRICHLOROHYDROQUINONE[c]'
'CPD-845[c]'
'CPD-3483[c]'
'CPD-3481[c]'
'CPD-17793[c]'
'CPD-15524[c]'
'CPD-15523[c]'
'CL-[e]'
'CPD-10634[c]'
'CPD-10633[c]'
'CPD-10636[c]'
'CPD-19732[c]'
'CPD-10659[c]'
'CPD-10660[c]'
'35-DICHLOROCATECHOL[c]'
'CPD-3486[c]'
'CPD-10576[c]'
'CPD-17792[c]'
'CPD-17789[c]'
'CPD-17786[c]'
'CPD-13112[c]'
'CPD-13114[c]'
'CPD-9674[c]'
'CIS-2-CHLORO-4-CARBOXYMETHYLENEBUT-2-EN-[c]'
'CPD-21212[c]'
'2-CHLOROMALEYLACETATE[c]'
'CPD-10616[c]'
'CPD-279[c]'
'CPD-19731[c]'
'CHLOROACETALDEHYDE[c]'
'CHLOROACETIC-ACID[c]'
'CPD-21167[c]'
'CPD-21169[c]'
'CPD-1776[c]'
'ATRAZINE[c]'
'CPD-21178[c]'
'CPD-21168[c]'
'CPD-21192[c]'
'CPD-19779[c]'
'CPD-19778[c]'
'CHLORDECONE-ALCOHOL[c]'
'CHLORDECONE[c]'
'Chlorides[c]'
'1-CHLORO-24-DINITROBENZENE[c]'
'34-DICHLOROANILINE[c]'
'CPD-16760[c]'
'4-CHLOROPHENYLACETONITRILE[c]'
'CPD-1786[c]'
'CPD-12672[c]'
'CPD-12673[c]'
'CPD-12676[c]'
'CPD-12677[c]'
'CPD-11020[c]'
'CPD-15409[c]'
'CPD-20607[c]'
'CPD-20606[c]'
'CPD-19761[c]'
'CPD-19763[c]'
'CPD-681[c]'
'TRANS-2-CHLORO-4-CARBOXYMETHYLENEBUT-2-E[c]'

-------------------------------------------------------------------------------

The chemical element **Co** is present in the empirical formula of **4 metabolites**

'METHIONINE-SYNTHASE-METHYLCOBALAMIN[c]'
'Methionine-synthase-cob-II-alamins[c]'
'ADENOSYLCOBALAMIN-5-P[c]'
'ADENOSYLCOBALAMIN[c]'

-------------------------------------------------------------------------------

The chemical element **Cu** is present in the empirical formula of **2 metabolites**

'CU+[c]'
'CU+[e]'

-------------------------------------------------------------------------------

The chemical element **F** is present in the empirical formula of **62 metabolites**

'FERRICYTOCHROME-B5[c]'
'FERROCYTOCHROME-B5[c]'
'Oxidized-2Fe-2S-Ferredoxins[c]'
'Reduced-2Fe-2S-Ferredoxins[c]'
'Reduced-adrenal-ferredoxins[c]'
'Oxidized-adrenal-ferredoxins[c]'
'Reduced-ferredoxins[c]'
'Oxidized-ferredoxins[c]'
'Cytochromes-B-Oxidized[c]'
'Cytochromes-B-Reduced[c]'
'Cytochromes-C-Oxidized[c]'
'Cytochromes-C-Reduced[c]'
'Reduced-ferredoxins[e]'
'FE+2[e]'
'FE+3[e]'
'FERROCYTOCHROME-B5[e]'
'Cytochromes-C-Reduced[e]'
'Cytochromes-C-Oxidized[e]'
'Oxidized-ferredoxins[e]'
'Ferrihemoglobins[c]'
'Ferrohemoglobins[c]'
'FE+3[c]'
'CPD-10157[e]'
'CPD-10157[c]'
'Oxidized-Putidaredoxins[c]'
'Reduced-Putidaredoxins[c]'
'FE+2[c]'
'FERRIC-ENTEROBACTIN-COMPLEX[c]'
'SIROHEME[c]'
'Fe2-siderophores[c]'
'Fe3-siderophores[c]'
'CPD-17082[c]'
'CPD-17185[c]'
'CPD0-2241[c]'
'Oxidized-cytochromes-c553[c]'
'Reduced-cytochromes-c553[c]'
'CPD-17070[c]'
'PROTOHEME[c]'
'CPD-13734[c]'
'Oxidized-CycA1-cytochromes[e]'
'Reduced-CycA1-cytochromes[e]'
'Reduced-cytochromes-c551[e]'
'Oxidized-cytochromes-c551[e]'
'Cytochromes-c[c]'
'HEME\_C[c]'
'Oxidized-Rubredoxins[c]'
'Reduced-Rubredoxins[c]'
'CPD-17063[c]'
'CPD0-1327[c]'
'CPD-15121[c]'
'CPD-5923[c]'
'CPD-12706[c]'
'CPD-12709[c]'
'F-[c]'
'CPD-16500[c]'
'CPD0-2482[c]'
'Oxidized-NapC-proteins[e]'
'Reduced-NapC-proteins[e]'
'an-oxidized-NrfB-protein[e]'
'a-reduced-NrfB-protein[e]'
'CPD-17185[e]'
'CPD0-2241[e]'

-------------------------------------------------------------------------------

The chemical element **Fe** is present in the empirical formula of **56 metabolites**

'FERRICYTOCHROME-B5[c]'
'FERROCYTOCHROME-B5[c]'
'Oxidized-2Fe-2S-Ferredoxins[c]'
'Reduced-2Fe-2S-Ferredoxins[c]'
'Reduced-adrenal-ferredoxins[c]'
'Oxidized-adrenal-ferredoxins[c]'
'Reduced-ferredoxins[c]'
'Oxidized-ferredoxins[c]'
'Cytochromes-B-Oxidized[c]'
'Cytochromes-B-Reduced[c]'
'Cytochromes-C-Oxidized[c]'
'Cytochromes-C-Reduced[c]'
'Reduced-ferredoxins[e]'
'FE+2[e]'
'FE+3[e]'
'FERROCYTOCHROME-B5[e]'
'Cytochromes-C-Reduced[e]'
'Cytochromes-C-Oxidized[e]'
'Oxidized-ferredoxins[e]'
'Ferrihemoglobins[c]'
'Ferrohemoglobins[c]'
'FE+3[c]'
'CPD-10157[e]'
'CPD-10157[c]'
'Oxidized-Putidaredoxins[c]'
'Reduced-Putidaredoxins[c]'
'FE+2[c]'
'FERRIC-ENTEROBACTIN-COMPLEX[c]'
'SIROHEME[c]'
'Fe2-siderophores[c]'
'Fe3-siderophores[c]'
'CPD-17082[c]'
'CPD-17185[c]'
'CPD0-2241[c]'
'Oxidized-cytochromes-c553[c]'
'Reduced-cytochromes-c553[c]'
'CPD-17070[c]'
'PROTOHEME[c]'
'CPD-13734[c]'
'Oxidized-CycA1-cytochromes[e]'
'Reduced-CycA1-cytochromes[e]'
'Reduced-cytochromes-c551[e]'
'Oxidized-cytochromes-c551[e]'
'Cytochromes-c[c]'
'HEME\_C[c]'
'Oxidized-Rubredoxins[c]'
'Reduced-Rubredoxins[c]'
'CPD-17063[c]'
'CPD-16500[c]'
'CPD0-2482[c]'
'Oxidized-NapC-proteins[e]'
'Reduced-NapC-proteins[e]'
'an-oxidized-NrfB-protein[e]'
'a-reduced-NrfB-protein[e]'
'CPD-17185[e]'
'CPD0-2241[e]'

-------------------------------------------------------------------------------

The chemical element **H** is present in the empirical formula of **5180 metabolites**

'GLC-1-P[c]'
'ALPHA-GLC-6-P[c]'
'CPD0-1812[c]'
'CPD-15977[c]'
'CO-A[c]'
'WATER[c]'
'MELIBIOSE[c]'
'GALACTOSE[c]'
'ALPHA-GLUCOSE[c]'
'OLEOYL-COA[c]'
'CTP[c]'
'L-1-LYSOPHOSPHATIDATE[c]'
'CDP[c]'
'PROTON[c]'
'WATER[e]'
'MELIBIOSE[e]'
'GALACTOSE[e]'
'ALPHA-GLUCOSE[e]'
'GLYCERALD[c]'
'NADP[c]'
'GLYCERATE[c]'
'NADPH[c]'
'HYDROGEN-PEROXIDE[c]'
'D-GALACTONATE[c]'
'CPD-12575[c]'
'D-Glucosyl-12-diacyl-glycerols[c]'
'diacyl-3-O-glucl-1-6-gluc-sn-glycerol[c]'
'CPD-1070[c]'
'CPD-277[c]'
'MAL[c]'
'PYRUVATE[c]'
'ACETYL-COA[c]'
'GLUCOSAMINE-1P[c]'
'N-ACETYL-D-GLUCOSAMINE-1-P[c]'
'DIACYLGLYCEROL[c]'
'Phospholipids[c]'
'Triacylglycerols[c]'
'GLYCEROL[c]'
'HYDROXYPROPANAL[c]'
'ADENOSINE[c]'
'ATP[c]'
'Odd-Saturated-Fatty-Acyl-CoA[c]'
'INOSINE[c]'
'AMMONIUM[c]'
'Odd-Straight-Chain-234-Sat-FA[c]'
'AMP[c]'
'PPI[c]'
'ADP[c]'
'PHYTOL[c]'
'NAD[c]'
'NADH[c]'
'2E-5Z-tetradeca-2-5-dienoyl-ACPs[c]'
'PHOSPHORYL-CHOLINE[c]'
'RHAMNOSE[c]'
'CPD0-1112[c]'
'5Z-tetradec-5-enoyl-ACPs[c]'
'CPD-541[c]'
'D-MYO-INOSITOL-1-MONOPHOSPHATE[c]'
'CPD0-1122[c]'
'CPD0-1123[c]'
'CPD-171[c]'
'DOLICHOLP[c]'
'MANNOSE[c]'
'MALONYL-ACP[c]'
'Chondroitin-N-acetyl-galactosamines[c]'
'7Z-3-oxo-hexadec-7-enoyl-ACPs[c]'
'ACP[c]'
'CHONDROITIN-4-SULFATE[c]'
'Heparan-sulfate-L-iduronate[c]'
'Ubiquinones[c]'
'Heparan-sulfate-L-IdoA-2S[c]'
'Heparan-NAc-Glc-6S[c]'
'FERRICYTOCHROME-B5[c]'
'LINOLEIC\_ACID[c]'
'9-CIS11-TRANS-OCTADECADIENOATE[c]'
'CPD-8091[c]'
'FERROCYTOCHROME-B5[c]'
'CPD-8092[c]'
'LINOLENIC\_ACID[c]'
'CPD-2181[c]'
'LINOLENOYL-COA[c]'
'CPD-2182[c]'
'CPD-8088[c]'
'CPD-8093[c]'
'N-ALPHA-ACETYLORNITHINE[c]'
'L-ORNITHINE[c]'
'ACET[c]'
'2-KETOGLUTARATE[c]'
'GLT[c]'
'CPD-469[c]'
'CPD-612[c]'
'CPD-15972[c]'
'Glucopyranose[c]'
'CPD-12384[c]'
'CPD-4578[c]'
'CPD-12385[c]'
'NADH-P-OR-NOP[c]'
'S-ADENOSYLMETHIONINE[c]'
'2-OCTAPRENYL-6-HYDROXYPHENOL[c]'
'CPD-12388[c]'
'2-OCTAPRENYL-6-METHOXYPHENOL[c]'
'ADENOSYL-HOMO-CYS[c]'
'OCTAPRENYL-METHYL-METHOXY-BENZQ[c]'
'CPD-12387[c]'
'4-METHYL-824-CHOLESTADIENOL[c]'
'5-HYDROXY-CONIFERALDEHYDE[c]'
'OCTAPRENYL-METHOXY-BENZOQUINONE[c]'
'SINAPALDEHYDE[c]'
'CPD-4579[c]'
'2-OCTAPRENYLPHENOL[c]'
'CPD-12390[c]'
'CPD-12391[c]'
'CPD-4580[c]'
'CPD-12393[c]'
'CPD-4702[c]'
'CPD-12139[c]'
'CPD-19502[c]'
'CPD-19504[c]'
'CPD-19503[c]'
'3-PHENYLPROPIONATE[c]'
'CPD-14673[c]'
'Linear-Malto-Oligosaccharides[c]'
'CPD-3628[c]'
'CPD-3629[c]'
'Ox-NADPH-Hemoprotein-Reductases[c]'
'CPD-3630[c]'
'CPD-14741[c]'
'N-6-AMINOHEXANOYL-6-AMINOHEXANOATE[c]'
'CPD-884[c]'
'CYCLOARTENOL[c]'
'CPD-10689[c]'
'N1-ACETYLSPERMINE[c]'
'CPD-11271[c]'
'CPD-313[c]'
'UDP-GLUCURONATE[c]'
'CPD-11398[c]'
'UDP[c]'
'L-THYROXINE[c]'
'LIOTHYRONINE[c]'
'CPD-11400[c]'
'LYS[c]'
'8-AMINO-7-OXONONANOATE[c]'
'DIAMINONONANOATE[c]'
'R-2-HYDROXYGLUTARATE[c]'
'Donor-H2[c]'
'CPD-208[c]'
'PROTEIN-LIPOYLLYSINE[c]'
'MET[c]'
'CH33ADO[c]'
'Unsulfurated-Sulfur-Acceptors[c]'
'Octanoylated-Gcv-H[c]'
'Sulfurated-Sulfur-Acceptors[c]'
'CPD-196[c]'
'a-pyruvate-dehydrogenase-E2-protein-Nsup[c]'
'pyruvate-dehydrogenase-E2-lipoyl-carrier[c]'
'DEOXYINOSINE[c]'
'Pi[c]'
'HYPOXANTHINE[c]'
'DEOXY-D-RIBOSE-1-PHOSPHATE[c]'
'DNA-deoxycytidine-thymidine-dimer[c]'
'Light[c]'
'DNA-Cytidines[c]'
'DNA-thymidines[c]'
'ACETALD[c]'
'GAP[c]'
'DEOXY-RIBOSE-5P[c]'
'GLUTARATE[c]'
'GLUTARYL-COA[c]'
'MALONYL-COA[c]'
'HEXANOYL-COA[c]'
'CPD-14687[c]'
'CPD-280[c]'
'CPD-14615[c]'
'ETF-Oxidized[c]'
'CPD-11507[c]'
'GLUTACONYL-COA[c]'
'ETF-Reduced[c]'
'CPD-11506[c]'
'CPD-18[c]'
'Heparan-NAc-Glc[c]'
'CPD-235[c]'
'NAD-P-OR-NOP[c]'
'56-Dihydrouracil17-in-tRNAs[c]'
'Uracil17-in-tRNAs[c]'
'MALTOSE[c]'
'Glucose[c]'
'56-Dihydrouracil20-in-tRNAs[c]'
'LOGANATE[c]'
'Uracil20-in-tRNAs[c]'
'LOGANIN[c]'
'Isomaltose[c]'
'Long-Chain-234-Saturated-acyl-CoAs[c]'
'56-Dihydrouracil47-in-tRNAs[c]'
'Uracil47-in-tRNAs[c]'
'Long-Chain-Trans-23-Dehydroacyl-CoA[c]'
'biotin-L-lysine-in-BCCP-dimers[c]'
'HCO3[c]'
'carboxybiotin-L-lysine-in-BCCP-dimers[c]'
'CPD-8089[c]'
'CPD-8090[c]'
'BCCP-L-lysine[c]'
'BIOTIN[c]'
'BCCP-biotin-L-lysine[c]'
'1-183-2-183-SN-GLYCEROL-PHOSPHOCHOLINE[c]'
'CPD-8094[c]'
'BIPHENYL-23-DIOL[c]'
'CPD-613[c]'
'CPD-8098[c]'
'CPD-676[c]'
'3R-7Z-3-hydroxy-hexadec-7-enoyl-ACPs[c]'
'2E-7Z-hexadeca-2-7-dienoyl-ACPs[c]'
'7Z-hexadec-7-enoyl-ACPs[c]'
'9Z-3-oxo-octadec-9-enoyl-ACPs[c]'
'Ubiquinols[c]'
'CPD-4581[c]'
'UBIQUINOL-30[c]'
'UBIQUINONE-6[c]'
'Alpha-lactose[c]'
'ALLOLACTOSE[c]'
'ZYMOSTEROL[c]'
'CPD-381[c]'
'CPD0-1158[c]'
'CPD0-1162[c]'
'Oxo-glutarate-dehydrogenase-DH-lipoyl[c]'
'CPD0-1163[c]'
'5-ALPHA-CHOLESTA-724-DIEN-3-BETA-OL[c]'
'CPD-12394[c]'
'CPD-12397[c]'
'CPD-12396[c]'
'CPD-12399[c]'
'CPD-12400[c]'
'CPD-12402[c]'
'CPD-12403[c]'
'CPD-12405[c]'
'CPD-12406[c]'
'BCAA-dehydrogenase-DH-lipoyl[c]'
'PHENYLACETOTHIOHYDROXIMATE[c]'
'CPD-12607[c]'
'CPD-696[c]'
'GAMMA-BUTYROBETAINE[c]'
'CPD-3462[c]'
'SPERMIDINE[c]'
'SUCC-S-ALD[c]'
'TRIMETHYLAMINE[c]'
'CPD66-39[c]'
'Fatty-Aldehydes[c]'
'Alcohols[c]'
'Glucosyl-ceramides[c]'
'2R-Hydroxy-Fatty-Acids[c]'
'N-ACETYLNEURAMINATE[c]'
'N-acetyl-D-mannosamine[c]'
'Ceramides[c]'
'PROTEIN-C-TERMINAL-S-ETC-CYSTEINE[c]'
'PROTEIN-C-TERMINAL-S-FARNESYL-L-CYSTEINE[c]'
'Short-Alpha-14-Glucans[c]'
'DIHYDROLIPOAMIDE[c]'
'CIS-ACONITATE[c]'
'LIPOAMIDE[c]'
'ITACONATE[c]'
'CIT[c]'
'6-DEMETHYLSTERIGMATOCYSTIN[c]'
'STERIGMATOCYSTIN[c]'
'STERIGMATOCYSTIN[e]'
'Ribonucleoside-Monophosphates[c]'
'CPD-11401[c]'
'CPD-11402[c]'
'CPD3O-4151[c]'
'PALMITYL-COA[c]'
'CPD-17621[c]'
'CROTONYL-COA[c]'
'GLUTATHIONE[c]'
'OXIDIZED-GLUTATHIONE[c]'
'R-4-PHOSPHOPANTOTHENOYL-L-CYSTEINE[c]'
'PANTETHEINE-P[c]'
'Pyruvate-dehydrogenase-lipoate[c]'
'a-2-oxoglutarate-dehydrogenase-E2-protei[c]'
'2-oxoglutarate-dehydrogenase-E2-lipoyl-c[c]'
'Oxo-glutarate-dehydrogenase-lipoyl[c]'
'SUC[c]'
'PHTYOSPHINGOSINE-1-P[c]'
'Rhodoquinols[c]'
'FUM[c]'
'Rhodoquinones[c]'
'SN-GLYCEROL-1-PHOSPHATE[c]'
'GLYCEROL-3P[c]'
'16-alpha-D-Mannosyloligosaccharides[c]'
'Unbranched-1-6-Mannan[c]'
'DEPHOSPHO-COA[c]'
'L-ALPHA-ALANINE[c]'
'CPD-630[c]'
'CPD-7000[c]'
'CPD-18077[c]'
'Glc2Man9GlcNAc2-proteins[c]'
'GLC[c]'
'CPD-14704[c]'
'1-4-alpha-D-Glucan[c]'
'MALTOTRIOSE[c]'
'Chitosan[c]'
'CPD-10806[c]'
'Chitosan-fragments[c]'
'ALPHA-MALTOSE[c]'
'CPD-14705[c]'
'PHOSPHORIBOSYL-CARBOXY-AMINOIMIDAZOLE[c]'
'DETHIOBIOTIN[c]'
'L-ASPARTATE[c]'
'Alpha-D-Glucuronides[c]'
'D-Glucopyranuronate[c]'
'CPD-14706[c]'
'GLY[c]'
'P-RIBOSYL-4-SUCCCARB-AMINOIMIDAZOLE[c]'
'CPD-19179[c]'
'GTP[c]'
'Guanine37-in-tRNA[c]'
'tRNA-Containing-N1-Methylguanine-37[c]'
'TMP[c]'
'ADENOSINE5TRIPHOSPHO5ADENOSINE[c]'
'DIHYDROFOLATE-GLU-N[c]'
'Guanine9-in-tRNA[c]'
'tRNA-Containing-N1-Methylguanine-9[c]'
'carbo-me-ur-34-tRNA[c]'
'5-2-me-oxy-2-oxo-et-ur-34-tRNA[c]'
'DPG[c]'
'PRECURSOR-Z[c]'
'23-DIPHOSPHOGLYCERATE[c]'
'CPD-4[c]'
'MPT-Synthase-small-subunits[c]'
'2-METHYL-3-HYDROXY-BUTYRYL-COA[c]'
'CPD-1083[c]'
'Thiocarboxylated-MPT-synthases[c]'
'tRNA-Adenine-58[c]'
'tRNA-Containing-N1-MethylAdenine-58[c]'
'BLASTICIDIN-S[c]'
'DEAMINOHYDROXYBLASTICIDIN-S[c]'
'Adenine57-Adenine58-tRNAs[c]'
'N1-MeAdenine57-MeAdenine58-tRNAs[c]'
'CPD-8122[c]'
'3R-9Z-3-hydroxy-octadec-9-enoyl-ACPs[c]'
'2E-9Z-octadeca-2-9-dienoyl-ACPs[c]'
'3-BETA-D-GLUCOSYLGLUCOSE[c]'
'CPD-1861[c]'
'CPD-4186[c]'
'CPD-4187[c]'
'METOH[c]'
'FORMATE[c]'
'CHOLESTEROL[c]'
'FORMALDEHYDE[c]'
'CPD-12449[c]'
'URACIL[c]'
'PHLORETIN[c]'
'PSEUDOURIDINE-5-P[c]'
'CPD-7727[c]'
'CPD-15317[c]'
'CPD66-21[c]'
'CPD-693[c]'
'LEUKOTRIENE-C4[c]'
'Amino-Acids-20[c]'
'5-L-GLUTAMYL-L-AMINO-ACID[c]'
'CPD-12451[c]'
'CPD-12452[c]'
'7E9E11Z14Z-5S6R-6-CYSTEIN-S-YL[c]'
'MYO-INOSITOL[c]'
'Long-Chain-Aldehydes[c]'
'Long-Chain-Acyl-CoAs[c]'
'CPD-4081[c]'
'ALPHA-METHYL-5-ALPHA-ERGOSTA[c]'
'L-ALLO-THREONINE[c]'
'CPD-4101[c]'
'AMINO-RIBOSYLAMINO-1H-3H-PYR-DIONE[c]'
'DIHYDROXY-BUTANONE-P[c]'
'CPD-19725[c]'
'CPD-7157[c]'
'PELARGONIDIN-CMPD[c]'
'CPD-591[c]'
'DNA-Cytosines[c]'
'DNA-N4-Methylcytosine[c]'
'CPD-19726[c]'
'S-SCOULERINE[c]'
'CPD-239[c]'
'4-P-PANTOTHENATE[c]'
'S-TETRAHYDROCOLUMBAMINE[c]'
'THREO-DS-ISO-CITRATE[c]'
'3-SULFINOALANINE[c]'
'L-CYSTEATE[c]'
'6-O-METHYLNORLAUDANOSOLINE[c]'
'CPD-15799[c]'
'Myelin-N-o-methyl-arginines[c]'
'R-3-Hydroxypalmitoyl-ACPs[c]'
'PAPS[c]'
'3-5-ADP[c]'
'CPD-11407[c]'
'CPD-11408[c]'
'3-oxo-palmitoyl-ACPs[c]'
'CPD-11409[c]'
'CPD-11403[c]'
'CYS[c]'
'CMP[c]'
'D-6-P-GLUCONO-DELTA-LACTONE[c]'
'RIBOSE[c]'
'RIBOSE-5P[c]'
'DGDP[c]'
'Carboxyadenylated-MPT-synthases[c]'
'L-Cysteine-Desulfurase-persulfide[c]'
'Cysteine-Desulfurase-L-cysteine[c]'
'D-altropyranoses[c]'
'PSICOSE[c]'
'CHITIN[c]'
'ETR-Quinols[c]'
'OXALACETIC\_ACID[c]'
'Chitodextrins[c]'
'ITP[c]'
'PHOSPHO-ENOL-PYRUVATE[c]'
'IDP[c]'
'Poly-ADP-Riboses[c]'
'ADENOSINE\_DIPHOSPHATE\_RIBOSE[c]'
'Xyloglucan[c]'
'Xyloglucan-oligosaccharides[c]'
'GLUCOSAMINE[c]'
'Peptidoglycans[c]'
'NAcMur-Peptide-Undecaprenols[c]'
'N-acetyl-D-glucosamine[c]'
'BUTANAL[c]'
'ALPHA-TOCOPHEROL[c]'
'GAMA-TOCOPHEROL[c]'
'RETINAL[c]'
'CPD-17278[c]'
'RETINOATE[c]'
'Alpha-linolenoyl-groups[c]'
'3-Oxo-Delta-4-Steroids[c]'
'3-Oxo-5-Alpha-Steroids[c]'
'CPD-8123[c]'
'Mercapturates[c]'
'S-Substituted-L-Cysteines[c]'
'L-GAMMA-GLUTAMYLCYSTEINE[c]'
'3-Beta-Hydroxysterols[c]'
'CPD-18246[c]'
'Malonyl-acp-methyl-ester[c]'
'3-Ketoglutaryl-ACP-methyl-ester[c]'
'ILE[c]'
'2-KETO-3-METHYL-VALERATE[c]'
'PROPIONYL-COA[c]'
'D-METHYL-MALONYL-COA[c]'
'LEU[c]'
'FADH2[c]'
'2K-4CH3-PENTANOATE[c]'
'FAD[c]'
'GLYCOLLATE[c]'
'Folates[c]'
'P-COUMAROYL-COA[c]'
'CAFFEOYL-COA[c]'
'CARNITINE[c]'
'CPD-19737[c]'
'CPD66-29[c]'
'BUTYRYL-COA[c]'
'CPD-19738[c]'
'3-BETA-HYDROXYANDROST-5-EN-17-ONE[c]'
'TETRADECANOYL-COA[c]'
'CPD-19740[c]'
'CPD-10267[c]'
'CPD-19741[c]'
'STEAROYL-COA[c]'
'CPD-19742[c]'
'17-BETA-HYDROXY-5ALPHA-ANDROSTAN-3-O[c]'
'Oleoyl-ACPs[c]'
'ACETYL-ACP[c]'
'11Z-3-oxo-icos-11-enoyl-ACPs[c]'
'CPD-4126[c]'
'L-GLYCERALDEHYDE-3-PHOSPHATE[c]'
'Cytidine-34-tRNAmet[c]'
'CPD-4125[c]'
'CPD-4127[c]'
'Elongator-tRNAMet-acetylcytidine[c]'
'CPD-4142[c]'
'DEHYDRO-3-DEOXY-L-RHAMNONATE[c]'
'CPD-706[c]'
'CPD-4141[c]'
'LACTALD[c]'
'CPD-4143[c]'
'CPD-707[c]'
'Red-NADPH-Hemoprotein-Reductases[c]'
'CPD-3943[c]'
'DIMETHYL-D-RIBITYL-LUMAZINE[c]'
'Myelin-L-arginines[c]'
'S-NORCOCLAURINE[c]'
'S-COCLAURINE[c]'
'DIHYDROSIROHYDROCHLORIN[c]'
'CPD-642[c]'
'METHIONINE-SYNTHASE-METHYLCOBALAMIN[c]'
'Methionine-synthase-cob-II-alamins[c]'
'2-Hexadecenoyl-ACPs[c]'
'Palmitoyl-ACPs[c]'
'GLYOX[c]'
'CPD-11411[c]'
'CPD-11404[c]'
'CPD-11412[c]'
'CPD-11410[c]'
'METHYLENE-THF-GLU-N[c]'
'5-10-METHENYL-THF-GLU-N[c]'
'CPD-1130[c]'
'CPD-3618[c]'
'L-DIHYDROXY-PHENYLALANINE[c]'
'COUMARATE[c]'
'Release-factor-L-glutamine[c]'
'Release-factor-N5-Methyl-L-glutamine[c]'
'CPD-15896[c]'
'CPD-17487[c]'
'DGTP[c]'
'CPD-674[c]'
'2-COUMARATE[c]'
'DEOXYGUANOSINE[c]'
'2-3-DIHYDROXYBENZOATE[c]'
'CPD-664[c]'
'DELTA1-PYRROLINE\_2-CARBOXYLATE[c]'
'3-Oxosteroids[c]'
'CPD-8124[c]'
'CPD-4161[c]'
'L-GULONO-1-4-LACTONE[c]'
'CPD-8134[c]'
'ASCORBATE[c]'
'CPD-8155[c]'
'CPD-7061[c]'
'4-AMINO-BUTYRATE[c]'
'CPD-8157[c]'
'CPD-8158[c]'
'ETOH[c]'
'TYR[c]'
'P-HYDROXY-PHENYLPYRUVATE[c]'
'Peptides-holder[c]'
'CPD-11495[c]'
'PHENYLACETATE[c]'
'CPD0-2244[c]'
'CPD0-2123[c]'
'Beta-D-glucosides[c]'
'Non-Glucosylated-Glucose-Acceptors[c]'
'5-METHYL-THF-GLU-N[c]'
'FORMYL-THF-GLU-N[c]'
'D-glucopyranose-6-phosphate[c]'
'Beta-D-Galactosides[c]'
'Non-Galactosylated-Galactose-Acceptors[c]'
'THF[c]'
'D-galactopyranose[c]'
'LAUROYLCOA-CPD[c]'
'LYS-tRNAs[c]'
'CPD-19743[c]'
'Charged-LYS-tRNAs[c]'
'CHOLINE[c]'
'GLYCOLALDEHYDE[c]'
'CPD-7682[c]'
'4-HYDROXY-BUTYRYL-COA[c]'
'OH-CROTONYL-COA[c]'
'3-Hydroxyglutaryl-ACP-methyl-ester[c]'
'3-P-SERINE[c]'
'CPD-19754[c]'
'ECTOINE[c]'
'CPD-19757[c]'
'CPD-10663[c]'
'CPD-19760[c]'
'2-Lysophosphatidylcholines[c]'
'HYDROQUINONE[c]'
'Enoylglutaryl-ACP-methyl-esters[c]'
'L-1-GLYCERO-PHOSPHORYLCHOLINE[c]'
'26-DICHLORO-P-HYDROQUINONE[c]'
'VAL[c]'
'2-KETO-ISOVALERATE[c]'
'PYRROLINE-HYDROXY-CARBOXYLATE[c]'
'4-HYDROXY-L-PROLINE[c]'
'ACYL-COA[c]'
'TESTOSTERONE[c]'
'3R-11Z-3-hydroxy-icos-11-enoyl-ACPs[c]'
'CPD66-23[c]'
'2E-11Z-icosa-2-11-dienoyl-ACPs[c]'
'11Z-icos-11-enoyl-ACPs[c]'
'CPD66-27[c]'
'CYS-tRNAs[c]'
'CPD-698[c]'
'ACETYLSERINE[c]'
'CPD-3945[c]'
'CPD-4162[c]'
'HS[c]'
'CPD-4181[c]'
'4-ALPHA-METHYL-5-ALPHA[c]'
'CPD-763[c]'
'METHYLARSONATE[c]'
'DIMETHYLARSINATE[c]'
'METHYLARSONITE[c]'
'CPD-4124[c]'
'CPD-225[c]'
'E-2-METHOXYCARBONYLMETHYLBUTENEDIOAT[c]'
'L-1-PHOSPHATIDYL-ETHANOLAMINE[c]'
'CPD-10260[c]'
'DIHYDRO-DIOH-BENZOATE[c]'
'CPD-10261[c]'
'CPD-10262[c]'
'THF-GLU-N[c]'
'CPD-9956[c]'
'DIHYDRO-NEO-PTERIN[c]'
'DIHYDRONEOPTERIN-P3[c]'
'OCTAPRENYL-METHYL-OH-METHOXY-BENZQ[c]'
'DIHYDROFOLATE[c]'
'Stearoyl-ACPs[c]'
'12-DICHLOROETHANE[c]'
'2-CHLOROETHANOL[c]'
'GERANYLGERANYL-PP[c]'
'LysW-L-glutamate[c]'
'CPD-12805[c]'
'LysW-L-glutamate-5-phosphate[c]'
'18-HYDROXYOLEATE[c]'
'LysW-L-glutamate-5-semialdehyde[c]'
'Lipid-hydroxy-fatty-acids[c]'
'CPD-21340[c]'
'A-LIPID-HYDROPEROXIDE[c]'
'910-EPOXY-18-HYDROXYSTEARATE[c]'
'GERANYL-PP[c]'
'DELTA3-ISOPENTENYL-PP[c]'
'CPD-11444[c]'
'COPROPORPHYRINOGEN\_I[c]'
'LysW-L-ornithine[c]'
'ALL-TRANS-HEXAPRENYL-DIPHOSPHATE[c]'
'CPD-15900[c]'
'3-HYDROXYPIMELYL-COA[c]'
'CPD-11447[c]'
'CPD1F-114[c]'
'PROTON[e]'
'L-ARABINOSE[e]'
'CPD-7733[c]'
'L-ARABINOSE[c]'
'CPD-15913[c]'
'Cis-Delta5-dodecenoyl-ACPs[c]'
'L-GULONATE[c]'
'D-GLUCURONOLACTONE[c]'
'2-OXOBUTANOATE[c]'
'2-ACETO-2-HYDROXY-BUTYRATE[c]'
'Charged-GLT-tRNAs[c]'
'GLT-tRNAs[c]'
'5-HYDROXY-FERULIC-ACID[c]'
'SINAPATE[c]'
'DOPAMINE[c]'
'ARABINOSE-5P[c]'
'RIBULOSE-5P[c]'
'Saturated-Fatty-Acyl-CoA[c]'
'G3P[c]'
'CPD-8620[c]'
'CPD-8619[c]'
'Sulfhydryls[c]'
'CPD-8529[c]'
'CPD-8621[c]'
'NOREPINEPHRINE[c]'
'L-EPINEPHRINE[c]'
'Methyl-thioethers[c]'
'DCDP[c]'
'DCTP[c]'
'Thiols[c]'
'CPD-621[c]'
'CPD-8065[c]'
'CPD-170[c]'
'CPD-8066[c]'
'CPD-1099[c]'
'SUCROSE[c]'
'CPD-8073[c]'
'CPD-8074[c]'
'CPD-8075[c]'
'CPD-8076[c]'
'CPD-7496[c]'
'CPD-7524[c]'
'CPD-12335[c]'
'CPD-12336[c]'
'CPD-12334[c]'
'CPD-14468[c]'
'E-11-TETRADECENOYL-COA[c]'
'CPD-17811[c]'
'CPD-17814[c]'
'CPD-17813[c]'
'L-LACTATE[c]'
'CPD-17815[c]'
'3-OXOPALMITOYL-COA[c]'
'CPD-17464[c]'
'CPD-358[c]'
'tRNA-Containing-N2-Dimethylgua-26-Gua27[c]'
'tRNA-Containing-N2-Methylgua-26-Gua27[c]'
'tRNA-Containing-N2-Dimetgua-26-MeGua27[c]'
'tRNA-Containing-N2-DiMeGua-26-DiMeGua27[c]'
'Guanine26-Guanine27-in-tRNAs[c]'
'Dodecanoyl-ACPs[c]'
'3-oxo-myristoyl-ACPs[c]'
'CPD-15684[c]'
'CPD-15685[c]'
'CPD-19273[c]'
'CPD-15686[c]'
'CPD-15687[c]'
'CPD-15688[c]'
'S2O3[c]'
'CPD-15689[c]'
'CPD-15690[c]'
'NYSTOSE[c]'
'1-KESTOTRIOSE[c]'
'CPD-15692[c]'
'CPD-15691[c]'
'H2CO3[c]'
'IMIDAZOLE\_ACETALDEHYDE[c]'
'CPD-14465[c]'
'CPD-14459[c]'
'CPD-14466[c]'
'CPD-14464[c]'
'CPD-14467[c]'
'4-IMIDAZOLEACETATE[c]'
'CPD-10847[c]'
'CPD-12358[c]'
'CPD-14471[c]'
'CPD-10844[c]'
'ACETOACETYL-COA[c]'
'2-Oxo-carboxylates[c]'
'L-GLUTAMATE\_GAMMA-SEMIALDEHYDE[c]'
'INOSITOL-1-4-5-TRISPHOSPHATE[c]'
'INOSITOL-1-4-BISPHOSPHATE[c]'
'PRO[c]'
'D-MYO-INOSITOL-4-PHOSPHATE[c]'
'PHOSPHATIDYLINOSITOL-345-TRIPHOSPHATE[c]'
'PHOSPHATIDYL-MYO-INOSITOL-45-BISPHOSPHA[c]'
'GUANOSINE[c]'
'GUANINE[c]'
'BENZALDEHYDE[c]'
'CPD0-1065[c]'
'BENZOATE[c]'
'CADAVERINE[c]'
'5-METHYLTHIOADENOSINE[c]'
'ALPHA-L-GLUTAMYL-PHOSPHATE[c]'
'S-ADENOSYLMETHIONINAMINE[c]'
'CARBAMATE[c]'
'BENZOYLCOA[c]'
'CPD-20052[c]'
'3-CARBOXY-3-HYDROXY-ISOCAPROATE[c]'
'CPD-318[c]'
'CPD-20051[c]'
'DCMP[c]'
'DUMP[c]'
'GlgE-Glycogen[c]'
'2K-ADIPATE[c]'
'DAIDZEIN[c]'
'Thiopurine-Methylethers[c]'
'CPD-8646[c]'
'Thiopurines[c]'
'DESMOSTEROL-CPD[c]'
'Red-Thioredoxin[c]'
'CPD-465[c]'
'SQUALENE[c]'
'CPD-10204[c]'
'Ox-Thioredoxin[c]'
'GDP-MANNOSE[c]'
'GDP-L-GALACTOSE[c]'
'D-LACTATE[c]'
'4-hydroxybenzoate[c]'
'CPD-7875[c]'
'4-Hydroxy-3-polyprenylbenzoates[c]'
'Polyisoprenyl-Diphosphates[c]'
'2-3-CARBOXY-3-AMINOPROPYL-L-HISTIDINE[c]'
'DTDP-DEOH-DEOXY-GLUCOSE[c]'
'L-Galactopyranose[c]'
'CPD-13952[c]'
'2-3-CARBOXY-3-METHYLAMMONIOPROPYL-L-[c]'
'CPD-9326[c]'
'eEF-2-Histidines[c]'
'CPD-17870[c]'
'3-carboxy-3-dimethylammonio-propyl-L-his[c]'
'DIPHTINE[c]'
'CPD-17877[c]'
'EPOXYSQUALENE[c]'
'LANOSTEROL[c]'
'CPD-17876[c]'
'CPD-2189[c]'
'CPD-8078[c]'
'CPD-330[c]'
'CPD-8080[c]'
'CPD-2190[c]'
'CPD-8077[c]'
'CPD-8079[c]'
'CPD-6948[c]'
'CPD-9459[c]'
'CPD-259[c]'
'CPD-8130[c]'
'CPD-9777[c]'
'CPD-9775[c]'
'CPD-401[c]'
'CPD-1823[c]'
'B-ALANINE[c]'
'XANTHOSINE-5-PHOSPHATE[c]'
'XANTHINE[c]'
'PRPP[c]'
'URATE[c]'
'CPD-1103[c]'
'4-HYDROXYBENZALDEHYDE[c]'
'CPD-7207[c]'
'CPD-13371[c]'
'GERANIAL[c]'
'CPD-13376[c]'
'CPD-13375[c]'
'CPD-13377[c]'
'CELLULOSE[c]'
'Cellulose-D-glucono-1-5-lactone[c]'
'Cytochromes-B-Oxidized[c]'
'Cytochromes-B-Reduced[c]'
'Cellodextrins[c]'
'CPD-7043[c]'
'CPD-3746[c]'
'FMNH2[c]'
'FMN[c]'
'D-ALPHABETA-D-HEPTOSE-7-PHOSPHATE[c]'
'T2-DECENOYL-COA[c]'
'CPD-10849[c]'
'CPD-10845[c]'
'CPD-12777[c]'
'D-arabinofuranose[c]'
'D-arabinopyranose[c]'
'CPD-9152[c]'
'L-arabinofuranose[c]'
'CPD-10870[c]'
'L-arabinopyranose[c]'
'MANNOSE-6P[c]'
'CPD-15711[c]'
'CPD-1241[c]'
'CPD-15712[c]'
'CPD-10866[c]'
'CPD-9151[c]'
'DUTP[c]'
'D-CYSTEINE[c]'
'FORMONONETIN[c]'
'VESTITONE[c]'
'2-HYDROXYFORMONONETIN[c]'
'CPD-3441[c]'
'ACETYL-GLU[c]'
'CPD-217[c]'
'CPD-3402[c]'
'CPD-3502[c]'
'2-HYDROXYISOFLAVANONE[c]'
'OROTIDINE-5-PHOSPHATE[c]'
'GLN[c]'
'OROTATE[c]'
'2-DEOXY-D-GLUCOSE-6-PHOSPHATE[c]'
'2-DEOXY-D-GLUCOSE[c]'
'PYRIDOXAL[c]'
'D-BETA-D-HEPTOSE-1-P[c]'
'PYRIDOXAL\_PHOSPHATE[c]'
'CPD-9001[c]'
'CPD-9002[c]'
'CPD-85[c]'
'CPD-8999[c]'
'CPD-10637[c]'
'CPD0-1080[c]'
'CPD0-1081[c]'
'CPD0-1082[c]'
'2-METHYL-BUTYRYL-COA[c]'
'CPD-10642[c]'
'N-ACETYL-D-GLUCOSAMINE[c]'
'CPD0-882[c]'
'L-ALA-GAMMA-D-GLU-DAP[c]'
'D-ALANINE[c]'
'4-TRIMETHYLAMMONIOBUTANAL[c]'
'Benzoin[c]'
'CPD-9869[c]'
'3-HYDROXY-N6N6N6-TRIMETHYL-L-LYSINE[c]'
'CPD-9871[c]'
'PHENYLGLYOXYLATE[c]'
'HCN[c]'
'CPD-17873[c]'
'CPD-17874[c]'
'CPD-8082[c]'
'CPD-8084[c]'
'CPD-16968[c]'
'CPD-17881[c]'
'CPD-17880[c]'
'CPD-17882[c]'
'CPD-8081[c]'
'CPD-8083[c]'
'CPD-9873[c]'
'CPD-4568[c]'
'LEU-tRNAs[c]'
'Charged-LEU-tRNAs[c]'
'Sphingoids[c]'
'Sphingoid-1-phosphates[c]'
'CPD0-1083[c]'
'CPD-12349[c]'
'L-GLYCERALDEHYDE[c]'
'CPD-460[c]'
'CPD-12352[c]'
'CPD-14594[c]'
'LINAMARIN[c]'
'CPD-19388[c]'
'CPD-15277[c]'
'CYS-GLY[c]'
'GLYCYLGLYCINE[c]'
'CPD-19395[c]'
'CPD-13031[c]'
'CPD-12702[c]'
'PHENYLACETONITRILE[c]'
'INDOLE-3-ACETALDOXIME[c]'
'CPD-13378[c]'
'R-3-hydroxymyristoyl-ACPs[c]'
'RIBOSE-1P[c]'
'XANTHOSINE[c]'
'CPD-13417[c]'
'CPD-13418[c]'
'CPD0-1202[c]'
'XYLOSE[c]'
'NEUROSPORENE[c]'
'CPD-10868[c]'
'CPD-294[c]'
'VERY-LONG-CHAIN-FATTY-ACYL-COA[c]'
'CPD-15723[c]'
'CPD-10615[c]'
'3-phosphooligonucleotides[c]'
'3-Prime-Nucleoside-Monophosphates[c]'
'CPD-15728[c]'
'Oligonucleotides[c]'
'Nucleoside-Monophosphates[c]'
'24-DICHLOROPHENOL[c]'
'CPD-8924[c]'
'CPD-15741[c]'
'L-1-phosphatidyl-inositols[c]'
'CPD-1121[c]'
'ALTROSE[c]'
'CPD-15781[c]'
'CPD-397[c]'
'CPD-15801[c]'
'CPD-377[c]'
'25-DIDEHYDRO-D-GLUCONATE[c]'
'ALLO-THR[c]'
'DIHYDROXY-ACETONE-PHOSPHATE[c]'
'TARTRONATE-S-ALD[c]'
'DEOXYCYTIDINE[c]'
'BENZYL-ALCOHOL[c]'
'3-OH-BENZYL-ALCOHOL[c]'
'3-OH-BENZALDEHYDE[c]'
'LEUCOPELARGONIDIN-CMPD[c]'
'BETA-CYCLOPIAZONATE[c]'
'ALPHA-CYCLOPIAZONATE[c]'
'UMP[c]'
'ACETYLCHOLINE[c]'
'PHOSPHORIBULOSYL-FORMIMINO-AICAR-P[c]'
'NONANE-46-DIONE[c]'
'Acetate-esters[c]'
'D-ERYTHRO-IMIDAZOLE-GLYCEROL-P[c]'
'CPD-4573[c]'
'44-DIMETHYL-CHOLESTA-814-24-TRIENOL[c]'
'44-DIMETHYL-824-CHOLESTADIENOL[c]'
'CPD-622[c]'
'CPD-1136[c]'
'CPD-8087[c]'
'CPD-8086[c]'
'CATECHOL[c]'
'CPD-12356[c]'
'CPD-12357[c]'
'CPD-14460[c]'
'CPD-12359[c]'
'CPD0-1905[c]'
'CPD-12365[c]'
'D-XYLONATE[c]'
'2-DH-3-DO-D-ARABINONATE[c]'
'D-XYLULOSE[c]'
'XYLULOSE-5-PHOSPHATE[c]'
'Very-Long-Chain-Aldehydes[c]'
'Alkanes[c]'
'Secondary-Alcohols[c]'
'Very-Long-Chain-Alkanes[c]'
'CPD-12321[c]'
'CPD-15798[c]'
'CPD-12932[c]'
'CPD-19475[c]'
'2-OXO-5-METHYLTHIOPENTANOIC-ACID[c]'
'CPD-8347[c]'
'PALMITATE[c]'
'CPDQT-40[c]'
'CPD-14596[c]'
'CPD-15800[c]'
'CPD-10277[c]'
'CPD-15742[c]'
'CPDQT-39[c]'
'CPD-14601[c]'
'CPD-14602[c]'
'CPD-19488[c]'
'PHOSPHATIDYLCHOLINE[c]'
'CPD-14604[c]'
'CPDQT-38[c]'
'CPD-19489[c]'
'Tetradec-2-enoyl-ACPs[c]'
'DEOXYADENOSINE[c]'
'DAMP[c]'
'ADENINE[c]'
'DADP[c]'
'2-DEHYDRO-3-DEOXY-D-GLUCONATE[c]'
'2-KETO-3-DEOXY-6-P-GLUCONATE[c]'
'AICAR[c]'
'Charged-THR-tRNAs[c]'
'Cyclic-3-5-Nucleoside-Monophosphates[c]'
'THR[c]'
'CPD-35[c]'
'D-THREONINE[c]'
'Cyclic-2-3-Ribonucleoside-Monophosphates[c]'
'AMINO-OXOBUT[c]'
'2-Prime-Ribonucleoside-Monophosphates[c]'
'AMINO-ACETONE[c]'
'GLN-tRNAs[c]'
'3Z-dodec-3-enoyl-ACPs[c]'
'OXALYL-COA[c]'
'CPD-1162[c]'
'GLC-6-P[c]'
'CPD-1181[c]'
'3R-5Z-3-hydroxy-tetradec-5-enoyl-ACPs[c]'
'5Z-3-oxo-tetradec-5-enoyl-ACPs[c]'
'CPD-173[c]'
'BUTYRIC\_ACID[c]'
'PENTAN-2-ONE[c]'
'Beta-D-Glucuronides[c]'
'Beta-Lactams[c]'
'CPD-8550[c]'
'CPD-448[c]'
'3-UREIDO-PROPIONATE[c]'
'N-ACETYL-GLUTAMYL-P[c]'
'CPD-4575[c]'
'CPD-4576[c]'
'CPD-667[c]'
'HOMO-CYS[c]'
'CPD-4577[c]'
'CPD-10254[c]'
'CPD-8892[c]'
'CPD-15[c]'
'245-DNOL[c]'
'SUC-COA[c]'
'25-DDOL[c]'
'3-KETO-ADIPYL-COA[c]'
'CPD-258[c]'
'CPD-320[c]'
'BENZENE-NO2[c]'
'CPD-12364[c]'
'PHE[c]'
'CPD-12363[c]'
'CPD-12521[c]'
'CPD-12595[c]'
'CPD-12377[c]'
'CPD-19490[c]'
'MALEATE[c]'
'CPDQT-37[c]'
'CPD-11268[c]'
'CPD-568[c]'
'CPD-10687[c]'
'CPDQT-36[c]'
'CPD-19491[c]'
'CPD-19492[c]'
'ACYL-ACP[c]'
'ACYL-SN-GLYCEROL-3P[c]'
'CPD-3740[c]'
'CPD-19493[c]'
'Omega-methylthio-alkyl-glucosinolates[c]'
'CPD-30[c]'
'omega-methylsulfinylalkylglucosinolate[c]'
'CPD-6082[c]'
'NORSPERMIDINE[c]'
'L-DELTA1-PYRROLINE\_5-CARBOXYLATE[c]'
'Protein-Phosphothreonines[c]'
'Proteins-L-Threonines[c]'
'CPD-15834[c]'
'CPD-11712[c]'
'ALLYSINE[c]'
'CAAL-proteins[c]'
'Geranylgeranylated-CAAL-proteins[c]'
'CPD-9539[c]'
'L-DEHYDRO-ASCORBATE[c]'
'OXAMATE[c]'
'DGMP[c]'
'CPD-389[c]'
'CARBAMOYL-P[c]'
'CPD-316[c]'
'RIBOFLAVIN[c]'
'CPD-9973[c]'
'LONG-CHAIN-KETONE[c]'
'EIF5A-HYPUSINE[c]'
'Charged-GLN-tRNAs[c]'
'CPD-3617[c]'
'O-PHOSPHO-L-HOMOSERINE[c]'
'THYMIDINE[c]'
'Myristoyl-ACPs[c]'
'THYMINE[c]'
'D-SEDOHEPTULOSE-7-P[c]'
'D-RIBULOSE[c]'
'CPD-15567[c]'
'CPD-15568[c]'
'PROPIONAMIDE[c]'
'CPD-8860[c]'
'BUTYRAMIDE[c]'
'CPD-12327[c]'
'CPD-3707[c]'
'PROPIONATE[c]'
'CPD-8548[c]'
'CPD-8549[c]'
'CPD-369[c]'
'CPD-15616[c]'
'236-TRICHLOROHYDROQUINONE[c]'
'Cytochromes-C-Oxidized[c]'
'Cytochromes-C-Reduced[c]'
'CPD-13025[c]'
'CPD-13223[c]'
'SPERMIDINE[e]'
'TRANS-2-HEXENOL[c]'
'TRANS-2-HEXENAL[c]'
'PUTRESCINE[e]'
'CIS-3-HEXENAL[c]'
'PUTRESCINE[c]'
'CIS-3-HEXENOL[c]'
'Pullulans[c]'
'7-O-ACETYLSALUTARIDINOL[c]'
'CPD-7710[c]'
'CPD-7712[c]'
'CPD-7713[c]'
'CODEINONE[c]'
'MORPHINONE[c]'
'CPD-10802[c]'
'CPD-10783[c]'
'METOH[e]'
'MYO-INOSITOL[e]'
'CPD-7836[e]'
'CPD-10784[c]'
'N-ACETYL-D-GLUCOSAMINE[e]'
'CPD-10803[c]'
'NADH-P-OR-NOP[e]'
'NADP[e]'
'CPD-10780[c]'
'NADPH[e]'
'CPD-10804[c]'
'AMMONIA[e]'
'NIACINAMIDE[e]'
'CPD-10785[c]'
'NIACINE[e]'
'CPD-10805[c]'
'2-METHYL-6-SOLANYL-14-BENZOQUINONE[c]'
'MALONATE-S-ALD[c]'
'CPD-14553[c]'
'CPD0-935[c]'
'CPD0-936[c]'
'CPD0-937[c]'
'CPD0-938[c]'
'CPD-17722[c]'
'CPD-17747[c]'
'OLEATE-CPD[c]'
'CPD-14378[c]'
'CPD-17726[c]'
'CPD-17727[c]'
'CPD-17728[c]'
'Amino-Acids[c]'
'CPD-17729[c]'
'2-Oxo-Acids[c]'
'Deoxyhypusine-Synthase-Lysine[c]'
'N-4-aminobutylidene-enzyme-lysine[c]'
'CPD-17732[c]'
'EIF5A-LYSINE[c]'
'CPD-17733[c]'
'DODECANOATE[c]'
'N-4-aminobutylidene-eIF5A-lysine[c]'
'General-Protein-Substrates[c]'
'CPD-17741[c]'
'Decanoyl-ACPs[c]'
'DNA-with-3-prime-pp-5-prime-G-cap[c]'
'3-Prime-Phosphate-Terminated-DNAs[c]'
'GMP[c]'
'3-oxo-dodecanoyl-ACPs[c]'
'DNA-Ligase-L-lysine-adenylate[c]'
'DNA-Ligase-L-lysine[c]'
'CPD-845[c]'
'A-5-prime-PP-5-prime-DNA[c]'
'O-SUCCINYLBENZOATE[c]'
'CPD-9923[c]'
'GLC-D-LACTONE[c]'
'GLUCONATE[c]'
'HEPTADECANE-CPD[c]'
'CPD-13469[c]'
'CPD-55[c]'
'FRUCTOSE-6P[c]'
'CPD-8611[c]'
'2-ACETO-LACTATE[c]'
'CPD-231[c]'
'CPD-8612[c]'
'CPD-2750[c]'
'D-Xylopyranose[c]'
'CPD-2742[c]'
'CPD-360[c]'
'CPD-409[c]'
'1-2-Diglycerides[c]'
'CPD-2752[c]'
'CPD-468[c]'
'XYLITOL[c]'
'ANDROST4ENE[c]'
'CPD-8505[e]'
'CPD-10174[c]'
'CPD-10174[e]'
'CPD-195[e]'
'OLEATE-CPD[e]'
'OXALACETIC\_ACID[e]'
'PALMITATE[e]'
'PANTOTHENATE[e]'
'CPD-8462[e]'
'CPD-10902[e]'
'PHENYLACETATE[e]'
'PLASTOQUINONE[c]'
'Plastoquinols[c]'
'CPD-13205[c]'
'CELLOBIOSE[c]'
'Phosphatase-2A-leucine[c]'
'Phosphatase-2A-leucine-methyl-ester[c]'
'SARCOSINE[c]'
'CODEINE[c]'
'MORPHINE[c]'
'CPD-4592[c]'
'CPD-4592[e]'
'ACETAMIDE[c]'
'D-Galactopyranuronate[c]'
'CPD-15633[c]'
'CPD-219[c]'
'CPD-15666[c]'
'2-KETO-6-AMINO-CAPROATE[c]'
'CPD-10809[c]'
'DIAMINO-OH-PHOSPHORIBOSYLAMINO-PYR[c]'
'CPD-1086[c]'
'Lignoceroyl-ACPs[c]'
'3-oxo-cerotoyl-ACPs[c]'
'TRINAPHTAL-CPD[c]'
'CPD-17743[c]'
'CPD-17730[c]'
'S-HYDROXYMETHYLGLUTATHIONE[c]'
'CPD-17744[c]'
'CPD-17746[c]'
'CPD-17750[c]'
'CPD-548[c]'
'CPD-17757[c]'
'CPD-13122[c]'
'CPD-702[c]'
'CPD-703[c]'
'FARNESYL-PP[c]'
'CPD0-1028[c]'
'MALTOHEXAOSE[c]'
'MALTOTETRAOSE[c]'
'CPD-12221[c]'
'CPD-3745[c]'
'CPD-201[c]'
'CPD-202[c]'
'DIACYLGLYCEROL-PYROPHOSPHATE[c]'
'L-PHOSPHATIDATE[c]'
'DNA-N[c]'
'CHOLATE[c]'
'CPD-14388[c]'
'3-Hydroxy-Terminated-DNAs[c]'
'Deacetylated-Peptidoglycan[c]'
'NICOTINAMIDE\_NUCLEOTIDE[c]'
'CPD-9776[c]'
'DNA-Ligase-L-lysine-guanylate[c]'
'CPD-14389[c]'
'CPD1F-140[c]'
'tRNA-precursors[c]'
'SS-Oligoribonucleotides[c]'
'CPD-236[c]'
'5-Phospho-terminated-DNAs[c]'
'CPD-14390[c]'
'Pi[e]'
'CPD-2751[c]'
'D-GLUCOSAMINE-6-P[c]'
'CPD-205[e]'
'PROPIONATE[e]'
'N-ACETYL-D-GLUCOSAMINE-6-P[c]'
'CPD-2747[c]'
'PYRUVATE[e]'
'CPD-3188[c]'
'QUINATE[e]'
'CPD-1099[e]'
'CPD-8613[c]'
'Red-NADPH-Hemoprotein-Reductases[e]'
'CPD-2749[c]'
'RIBOFLAVIN[e]'
'R-3-hydroxydodecanoyl-ACPs[c]'
'OCTANOL[c]'
'CPD-371[c]'
'CPD-7616[c]'
'3-4-DIHYDROXYBENZOATE[c]'
'THZ-P[c]'
'AMINO-HYDROXYMETHYL-METHYLPYRIMIDINE-PP[c]'
'THIAMINE-P[c]'
'THIAMINE-PYROPHOSPHATE[c]'
'CPD-611[c]'
'THIAMINE[c]'
'PYRIDINE[c]'
'THZ[c]'
'2-CARBOXY-D-ARABINITOL[c]'
'2-CARBOXY-D-ARABINITOL-1-PHOSPHATASE[c]'
'ACETYL-P[c]'
'3-KETOBUTYRATE[c]'
'L-PIPECOLATE[c]'
'Alpha-1-3-Branched-Arabinans[c]'
'L-RHAMNONATE[c]'
'L-RHAMNONO-14-LACTONE[c]'
'CPD1F-129[c]'
'CPD-7850[c]'
'GERANIOL[c]'
'CPD-7857[c]'
'CPD-7860[c]'
'CPD-7849[c]'
'CPD-15637[c]'
'CPD-15653[c]'
'CPD-15668[c]'
'CPD-15667[c]'
'CPD-15654[c]'
'CPD-15655[c]'
'CPD-13665[c]'
'5-BETA-ANDROSTANE-317-DIONE[c]'
'CPD-125[c]'
'CPD-14077[c]'
'R-3-hydroxycerotoyl-ACPs[c]'
'NONAPRENYL-4-HYDROXYBENZOATE[c]'
'Trans-D2-hexacos-2-enoyl-ACPs[c]'
'CPD-107[c]'
'CPD-9863[c]'
'Cerotoyl-ACPs[c]'
'CPD-14392[c]'
'Sphingomyelins[e]'
'CPD-11541[c]'
'STEARIC\_ACID[e]'
'CPD-14018[c]'
'Sterols[e]'
'5Z8Z11Z14Z17Z-EICOSAPENTAENOATE[c]'
'Steryl-Esters[e]'
'SUC[e]'
'SUCROSE[e]'
'CPD-6951[c]'
'HS[e]'
'FERULOYL-COA[c]'
'CPD-501[c]'
'UTP[c]'
'UDP-D-GALACTURONATE[c]'
'CPD-8633[c]'
'CPD-8634[c]'
'CPD-12231[c]'
'CPD-12261[c]'
'CPD-15377[c]'
'CPD-9868[c]'
'CPD-12279[c]'
'CPD-108[c]'
'CPD-9866[c]'
'RNA-DNA-hybrids[c]'
'DNA-Holder[c]'
'CPD-2961[c]'
'RNA-Containing-Guanosine[c]'
'RNA-3prime-Guanosine-3prime-P[c]'
'5Prime-OH-Terminated-RNAs[c]'
'G-5-prime-PP-5-prime-DNA[c]'
'3-KETOACYL-COA[c]'
'CPD-9872[c]'
'CPD-9870[c]'
'RNA-Ligase-L-lysine[c]'
'L-3-HYDROXYACYL-COA[c]'
'RNA-Ligase-L-lysine-adenylate[c]'
'PORPHOBILINOGEN[c]'
'5-Phospho-RNA[c]'
'HYDROXYMETHYLBILANE[c]'
'A-5-prime-PP-5-prime-RNA[c]'
'RNA-Holder[c]'
'3Prime-OH-Terminated-RNAs[c]'
'DATP[c]'
'ERYTHROSE-4P[c]'
'3-DEOXY-D-ARABINO-HEPTULOSONATE-7-P[c]'
'Oxidized-Flavoproteins[c]'
'Reduced-Flavoproteins[c]'
'ALPHA-GLUCOSE-16-BISPHOSPHATE[c]'
'CPD-8614[c]'
'CPD-3483[c]'
'CPD-3481[c]'
'GLC-D-LACTONE[e]'
'Glucopyranose[e]'
'CPD-4184[c]'
'CPD-1826[c]'
'2-DEHYDROPANTOATE[c]'
'L-PANTOATE[c]'
'HMP[c]'
'CROTONATE[c]'
'Dodec-2-enoyl-ACPs[c]'
'Thiocarboxyadenylated-ThiS-Proteins[c]'
'CPD-13575[c]'
'ACETOIN[c]'
'L-RHAMNOFURANOSE[c]'
'DIACETYL[c]'
'TARTRATE[e]'
'THIAMINE[e]'
'Triacylglycerides[e]'
'UBIQUINONE-6[e]'
'UBIQUINONE-8[e]'
'URACIL[e]'
'CPD-10353[c]'
'UREA[e]'
'L-XYLULOSE[c]'
'URIDINE[e]'
'CPD-13357[c]'
'VALERATE[e]'
'CPD-7953[c]'
'CPD-7952[c]'
'CPD-7951[c]'
'STRICTOSIDINE-AGLYCONE[c]'
'CPD-21552[c]'
'GEISSOSCHIZINE[c]'
'POLYNEURIDINE-ALDEHYDE[c]'
'CPD-7117[c]'
'CPD-7139[c]'
'HIF-alpha-subunit-L-asparagines[c]'
'HIF-alpha-subunit-3S-OH-ASN[c]'
'CPD-8815[c]'
'CPD-15895[c]'
'4-METHYLCATECHOL[c]'
'CPD-12288[c]'
'CPD-10664[c]'
'Glucosyl-acyl-sphinganines[c]'
'Glucosyl-acyl-sphingosines[c]'
'R-6-HYDROXYNICOTINE[c]'
'3-Prime-Phosphate-Terminated-RNAs[c]'
'CPD-14407[c]'
'CPD-8120[c]'
'CPD0-2350[c]'
'Pre-tRNA-5-prime-half-molecules[c]'
'Pre-tRNA-3-prime-half-molecules[c]'
'CPD-17794[c]'
'CPD-17791[c]'
'CPD-14422[c]'
'CPD-14423[c]'
'CPD-14424[c]'
'CPD-17793[c]'
'Protein-Ser-or-Thr-phosphate[c]'
'Protein-L-serine-or-L-threonine[c]'
'CPD-14425[c]'
'CPD-14426[c]'
'CPD-13328[c]'
'SCOPOLETIN[c]'
'CPD-15656[c]'
'CPD-3041[c]'
'CPD-15657[c]'
'PYRIDOXAL[e]'
'Nucleobases-in-DNA[c]'
'CPD-3061[c]'
'Methylated-DNA-Bases[c]'
'CPD-15675[c]'
'MALONATE[c]'
'CPD-15651[c]'
'CPD-15652[c]'
'BUTANOL[c]'
'CPD-13346[c]'
'CPD-15677[c]'
'CPD-15676[c]'
'AMINO-HYDROXYMETHYL-METHYL-PYR-P[c]'
'DNA-3-methyladenines[c]'
'DNA-containing-aPurinic-Sites[c]'
'3-Methyl-Adenines[c]'
'QUINOLINATE[e]'
'CPD-57[c]'
'RNA-with-3-prime-pp-5-prime-A-cap[c]'
'Cyclic-Phosphate-Terminated-RNAs[c]'
'RNA-3-prime-P-cyclase-L-histidine[c]'
'RNA-3-prime-P-cyclase-L-His-adenylate[c]'
'Nucleosides[c]'
'Ribonucleosides[c]'
'CPD-8617[c]'
'CPD-8618[c]'
'N-Acylsphingosine[c]'
'CPD-15530[c]'
'XANTHINE[e]'
'Xylans[e]'
'XYLITOL[e]'
'Amino-Acids-20[e]'
'Nucleosides[e]'
'2-HYDROXY-2-METHYLPROPANENITRILE[c]'
'ACETONE[c]'
'1-KETO-2-METHYLVALERATE[c]'
'BETAINE\_ALDEHYDE[c]'
'S-2-Haloacids[c]'
'PALMITALDEHYDE[c]'
'CPD-292[c]'
'CPD-5164[c]'
'Thi-S[c]'
'DEOXYXYLULOSE-5P[c]'
'CPD-17883[c]'
'CPD-5165[c]'
'CPD-17884[c]'
'CPD-5166[c]'
'D-ALA-D-ALA[c]'
'CPD-17885[c]'
'AMMONIA[c]'
'CPD-5167[c]'
'S-ADENOSYL-4-METHYLTHIO-2-OXOBUTANOATE[c]'
'CPD-17799[c]'
'5-AMINOPENTANOATE[c]'
'CPD-17787[c]'
'CPD-17800[c]'
'CPD-17887[c]'
'CPD-17801[c]'
'CPD-12303[c]'
'UDP-N-ACETYL-D-GLUCOSAMINE[c]'
'CPD-12304[c]'
'CPD-394[c]'
'CPD-17802[c]'
'CPD-7993[c]'
'CPD-12258[c]'
'CPD-9646[c]'
'CPD-17805[c]'
'CPD-12311[c]'
'CPD-7994[c]'
'CPD-12310[c]'
'CPD-17888[c]'
'Z-11-TETRADECENOYL-COA[c]'
'NICOTINE[c]'
'CPD-2748[c]'
'CPD-5168[c]'
'CPD-7535[c]'
'CPD-17807[c]'
'CPD-17809[c]'
'CPD-7526[c]'
'COUMARIN[c]'
'DIHYDROCOUMARIN[c]'
'Uridine44-in-tRNA-Ser[c]'
'2-O-Methyluridine44-tRNASer[c]'
'Guanine10-in-tRNA[c]'
'tRNA-Containing-N2-Methylguanine-10[c]'
'Guanine26-in-tRNA[c]'
'tRNA-Containing-N2-Methylguanine-26[c]'
'tRNA-Containing-N2-dimethylguanine-26[c]'
'CPD-17858[c]'
'CPD-17894[c]'
'GDP[c]'
'L-CANALINE[c]'
'UREA[c]'
'CANAVANINE[c]'
'CPD-15661[c]'
'CPD-10825[c]'
'CPD-10826[c]'
'CPD-10832[c]'
'CPD-15662[c]'
'CPD-15678[c]'
'CPD-15663[c]'
'CPD-9407[c]'
'CPD-15658[c]'
'CPD0-1308[c]'
'CPD0-1074[c]'
'CPD-13792[c]'
'Short-Chain-Trans-23-Dehydroacyl-CoA[c]'
'Short-Chain-234-Saturated-acyl-CoAs[c]'
'Very-Long-Chain-Trans-23-Dehydroacyl-CoA[c]'
'CPD-19268[c]'
'Very-long-Chain-234-Saturated-acyl-CoAs[c]'
'12-DEHYDROTETRACYCLINE[c]'
'CPD-19274[c]'
'CPD-19272[c]'
'VAL-tRNAs[c]'
'Charged-VAL-tRNAs[c]'
'BETAINE[c]'
'DICARBOXYLIC-ACID-MONOAMIDES[c]'
'L-CITRULLINE[c]'
'CPD-19474[c]'
'Pyruvate-Dehydrogenase-Phosphoserine[c]'
'Pyruvate-dehydrogenase-L-serine[c]'
'CPD-535[c]'
'CPD-19486[c]'
'FRUCTOSE-2-PHOSPHATE[c]'
'CPD-22266[c]'
'HYDROXYBENZOQUINONE[c]'
'CPD-22267[c]'
'Methyl-esterified-homogalacturonan[c]'
'1-4-alpha-D-galacturonosyl[c]'
'CPD-19487[c]'
'TRYPANOTHIONE-DISULFIDE[c]'
'TRYPANOTHIONE[c]'
'Dihydro-Lipoyl-Proteins[c]'
'Lipoyl-Protein-N6-lipoyllysine[c]'
'D-GLT[c]'
'D-Amino-Acids[c]'
'N-ACETYL-D-AMINO-ACID[c]'
'CPD-7418[c]'
'CPD-7417[c]'
'3-Hydroxy-octanoyl-ACPs[c]'
'3-Oxo-octanoyl-ACPs[c]'
'PHYTOSPINGOSINE[c]'
'CPD-15382[e]'
'tRNAPhe-Containing-4-demethylwyosine-37[c]'
'D-GALACTONO-1-4-LACTONE[e]'
'ALPHA-D-GALACTOSE[e]'
'yW-86[c]'
'Octadec-2-enoyl-ACPs[c]'
'GLUCOSAMINE[e]'
'yW-58[c]'
'tRNAPhe-wybutosine[c]'
'yW-72[c]'
'CPD-15438[e]'
'n-Alkanals[c]'
'MANNITOL[e]'
'Alk-2-enals[c]'
'2-HEXAPRENYL-6-METHOXY-14-BENZOQUINOL[c]'
'MANNOSE[e]'
'2-HEXAPRENYL-3-METHYL-6-METHOXY-14-BENZ[c]'
'Donor-H2[e]'
'OHyWstar-tRNA[c]'
'OHyW-58-tRNAPhe[c]'
'HYDANTOIN[c]'
'N-CARBAMOYLGLYCINE[c]'
'BUTANEDIOL[c]'
'CPD-111[c]'
'CPD-8782[c]'
'CPD-8781[c]'
'SULFO-CYSTEINE[c]'
'GDP-4-DEHYDRO-6-DEOXY-D-MANNOSE[c]'
'CPD-11281[c]'
'421-DEHYDROGEISSOSCHIZINE[c]'
'N-5S-5-AMINO-5-CARBOXYPENTANOYL-L-CY[c]'
'Apo-Propionyl-CoA-CO2-ligases[c]'
'Propionyl-CoA-CO2-ligases[c]'
'3-methylcrotonoyl-CoA-carboxylase-lysine[c]'
'Biotin-EC6-4-1-4[c]'
'CPD-14280[c]'
'CPD-10279[c]'
'CPD-14281[c]'
'CPD-10280[c]'
'CPD-14282[c]'
'NMNH[c]'
'CPD0-881[c]'
'CPD1G-277[c]'
'CPD-14283[c]'
'CPDQT-520[c]'
'CPD-2183[c]'
'CPD-14300[c]'
'CANAVANINOSUCCINATE[c]'
'CPD-479[c]'
'2-2-METHYLTHIOETHYLMALIC-ACID[c]'
'3-2-METHYLTHIOETHYLMALIC-ACID[c]'
'HOMOMETHIONINE[c]'
'STEARIC\_ACID[c]'
'N-Ac-L-methionyl-L-tyrosinyl-Protein[c]'
'N-Ac-N-terminal-L-valine[c]'
'N-terminal-L-valine[c]'
'N-terminal-L-alanine[c]'
'N-terminal-N-Ac-L-alanine[c]'
'16-HYDROXYPALMITATE[c]'
'N-terminal-L-cysteine[c]'
'N-terminal-N-Ac-L-cysteine[c]'
'CPD-9406[c]'
'CPD-17635[c]'
'N-terminal-glycine[c]'
'N-terminal-N-Ac-glycine[c]'
'N-terminal-N-Ac-L-Serine[c]'
'N-terminal-L-Serine[c]'
'CoM[c]'
'2-OXOPROPYL-COM[c]'
'PRENAL[c]'
'S-PRENYL-L-CYSTEINE[c]'
'Protein-L-methionine-R-S-oxides[c]'
'Protein-L-methionine[c]'
'CPD-8989[c]'
'RIBOSE[e]'
'D-Xylose[e]'
'CPD-16017[e]'
'ETOH[e]'
'CPD-320[e]'
'FERROCYTOCHROME-B5[e]'
'FMN[e]'
'Folates[e]'
'FORMATE[e]'
'OHyW-tRNAPhe[c]'
'ARG[c]'
'CPD-7419[c]'
'CPD-15413[c]'
'25S-rRNA-adenine-2142[c]'
'25S-rRNA-N1-methyladenine-2142[c]'
'CPD-7422[c]'
'25S-rRNA-adenine-645[c]'
'25S-rRNA-N1-methyladenine-645[c]'
'S-palmitoyl-L-cysteine-in-proteins[c]'
'CPD-7554[c]'
'PROT-CYS[c]'
'CPD-7556[c]'
'2-Octenoyl-ACPs[c]'
'apo-Transcarboxylases[c]'
'CPD-355[c]'
'CPD-569[c]'
'L-aspartyl-tRNAAsn[c]'
'Octanoyl-ACPs[c]'
'Charged-ASN-tRNAs[c]'
'CPD-356[c]'
'L-glutamyl-tRNAGln[c]'
'CPD-220[c]'
'CPD-13174[c]'
'CPD-13172[c]'
'5-HYDROXY-FERULOYL-COA[c]'
'4-SULFOBENZALDEHYDE[c]'
'CPD-257[c]'
'CPD-12180[c]'
'CPD-6602[c]'
'O-Long-Chain-Acyl-L-Carnitines[c]'
'SINAPOYL-COA[c]'
'3-HYDROXY-CISCIS-MUCONATE[c]'
'18S-rRNA-pseudouridine-1191[c]'
'3-HEXAPRENYL-4-HYDROXYBENZOATE[c]'
'18S-rRNA-N1-methylpseudouridine-1191[c]'
'CPD-21768[c]'
'VLC-Alpha-hydroxyphytoceramides[c]'
'IPC[c]'
'VLC-MIPC[c]'
'CPD-14305[c]'
'DI-H-OROTATE[c]'
'CPD-21769[c]'
'CPD-21770[c]'
'CPD-14304[c]'
'2-HEXAPRENYL-3-METHYL-5-HYDROXY-6-METHOX[c]'
'CPD-1108[c]'
'CPD-633[c]'
'3-METHYLTHIOPROPANALDOXIME[c]'
'CPD-7546[c]'
'3-METHYLTHIOPROPYLHYDROXAMIC-ACID[c]'
'3-METHYLTHIOPROPYL-DESULFO-GLUCOSINOLATE[c]'
'3-METHYLTHIOPROPYL-GLUCOSINOLATE[c]'
'3-METHYLSULFINYLPROPYL-GLUCOSINOLATE[c]'
'2-PROPENYL-GLUCOSINOLATE[c]'
'CPD-8990[c]'
'FUM[e]'
'4-AMINO-BUTYRATE[e]'
'Glucose[e]'
'GLUTATHIONE[e]'
'GLYCEROL[e]'
'Xylans[c]'
'GLY[e]'
'P-BENZOQUINONE[c]'
'Single-Stranded-DNAs[c]'
'Ribonucleoside-Triphosphates[c]'
'Glycogens[e]'
'ssDNA-RNA-primer-hybrid[c]'
'GLYCOLLATE[e]'
'GUANINE[e]'
'APS[c]'
'CAMP[c]'
'HYDROGEN-PEROXIDE[e]'
'CPD-7830[e]'
'L-ARABITOL[c]'
'CPD-1117[c]'
'CPD-1134[c]'
'O-UREIDOHOMOSERINE[c]'
'CPD-7652[c]'
'DIHYDROPTERIN-CH2OH-PP[c]'
'CPD-10766[c]'
'Protein-N-terminal-N-Ac-L-threonine[c]'
'Protein-N-terminal-L-threonine[c]'
'N-terminal-L-Serine-Histone-H2A[c]'
'N-terminal-N-Ac-L-Serine-Histone-2A[c]'
'Nucleotides[c]'
'N-terminal-L-Serine-Histone-H4[c]'
'N-terminal-N-Ac-L-Serine-Histone-4[c]'
'L-methionyl-L-lysyl-Protein[c]'
'N-Ac-L-methionyl-L-lysyl-Protein[c]'
'L-methionyl-L-valyl-Protein[c]'
'N-Ac-L-methionyl-L-valyl-Protein[c]'
'L-methionyl-L-alanyl-Protein[c]'
'N-Ac-L-methionyl-L-alanyl-Protein[c]'
'Nucleoside-Triphosphates[c]'
'FRUCTOSE-16-DIPHOSPHATE[c]'
'CPD-17640[c]'
'CPD-656[c]'
'CPD-17370[c]'
'CPD-7557[c]'
'CPD-7558[c]'
'CPD-15435[c]'
'N6-L-threonylcarbamoyladenine37-tRNAs[c]'
'tRNA-adenine-37[c]'
'CPD-15438[c]'
'L-rhamnopyranose[c]'
'CPD-15440[c]'
'CPD-15436[c]'
'Butanoyl-ACPs[c]'
'3-oxo-decanoyl-ACPs[c]'
'SULFOACETALDEHYDE[c]'
'CPD-10794[c]'
'3-HYDROXYADIPYL-COA[c]'
'TRANS-23-DEHYDROADIPYL-COA[c]'
'5-METHYLTHIOINOSINE[c]'
'1-PHOSPHATIDYL-1D-MYO-INOSITOL-34-BISPH[c]'
'2-PG[c]'
'CHOCOLA\_A[c]'
'CPD-13524[c]'
'CPD-13175[c]'
'MET[e]'
'CPD-452[c]'
'SALICYLALDEHYDE[c]'
'CPD-1113[c]'
'ERGOSTEROL[c]'
'CPD-11602[c]'
'CPD-882[c]'
'CPD-881[c]'
'CPD-14355[c]'
'CPD-160[c]'
'CPD-13955[c]'
'CPD-12199[c]'
'CPD-7619[e]'
'HYPOXANTHINE[e]'
'ARACHIDIC\_ACID[e]'
'Inulin[e]'
'THREO-DS-ISO-CITRATE[e]'
'CPD-7676[c]'
'CPD-468[e]'
'Alpha-lactose[e]'
'CPD-15972[e]'
'L-ALPHA-ALANINE[e]'
'L-ARABITOL[e]'
'ARG[e]'
'L-methionyl-L-seryl-Protein[c]'
'N-Ac-L-methionyl-L-seryl-Protein[c]'
'L-methionyl-L-threonyl-Protein[c]'
'N-Ac-L-methionyl-L-threonyl-Protein[c]'
'Aminopeptidase-Substrates[c]'
'L-methionyl-L-cysteinyl-Protein[c]'
'L-methionyl-glycyl-Protein[c]'
'CPD-8198[c]'
'IMINOASPARTATE[c]'
'L-Fucopyranoses[c]'
'METHYL-BETA-D-GALACTOSIDE[c]'
'CPD-730[c]'
'CPD-729[c]'
'1-3-beta-D-Glucans[c]'
'CPD-17642[c]'
'CPD-17644[c]'
'CPD-17641[c]'
'PHOSPHORIBOSYL-ATP[c]'
'CPD-17643[c]'
'CPD-17638[c]'
'CPD-17647[c]'
'CPD-9570[c]'
'CPD-357[c]'
'METHYL-GLYOXAL[c]'
'ASN[e]'
'CPD-7545[c]'
'L-ASPARTATE[e]'
'CPD1F-135[c]'
'CPD-15414[c]'
'DODECANOATE[e]'
'CPD-7424[c]'
'CPD-15423[c]'
'L-CITRULLINE[e]'
'CYS[e]'
'CPD-15502[c]'
'GLT[e]'
'CPD-15467[c]'
'GLN[e]'
'HIS[e]'
'INDOLE\_PYRUVATE[c]'
'TRP[c]'
'HOMO-CYS[e]'
'CPD-15522[c]'
'CPD-369[e]'
'CPD-15521[c]'
'CPD-7618[c]'
'CHOLANATE2[c]'
'LIPOIC-ACID[e]'
'CPD-10797[c]'
'CPD-10796[c]'
'CPD-15554[c]'
'CPD-335[c]'
'CPD-10781[c]'
'3-OXODODECANOATE[c]'
'TAGATOSE-1-6-DIPHOSPHATE[c]'
'CPD-7706[c]'
'O-Acyl-L-Carnitines[e]'
'O-Acyl-L-Carnitines[c]'
'Beta-hydroxydecanoyl-ACPs[c]'
'CPD-8606[c]'
'CPD-184[c]'
'4-OXALOMESACONATE[c]'
'Acetylxylan[c]'
'Feruloyl-polysaccharides[c]'
'4-OH-4-ACETYL-2-OXOGLUTARATE[c]'
'CPD-10600[c]'
'FERULIC-ACID[c]'
'CPD-13181[c]'
'CPD-13182[c]'
'CPD-3571[c]'
'HOMO-SER[c]'
'Enones[c]'
'DMPBQ[c]'
'CPD-13187[c]'
'CPD-13188[c]'
'16-EPIVELLOSIMINE[c]'
'MPBQ[c]'
'CPD-665[c]'
'DELTA-TOCOPHEROL[c]'
'CPD0-1470[c]'
'CPD-19217[c]'
'CPD-1763[c]'
'S-NITROSOGLUTATHIONE[c]'
'CPD-13617[c]'
'Charged-ARG-tRNAs[c]'
'L-Glutamyl-Peptides[c]'
'CPD-7671[c]'
'Lipoyl-Protein-L-Lysine[c]'
'CPD-195[c]'
'Octanoylated-domains[c]'
'N-ETHYLMALEIMIDE[c]'
'CPD0-903[c]'
'ILE[e]'
'LEU[e]'
'5-KETO-4-DEOXY-D-GLUCARATE[c]'
'D-GLUCARATE[c]'
'LYS[e]'
'L-ORNITHINE[e]'
'CPD-17624[c]'
'PHE[e]'
'CPD-15382[c]'
'PRO[e]'
'L-RIBULOSE[e]'
'OCTADEC-9-ENE-118-DIOIC-ACID[c]'
'SER[e]'
'Phosphoacetylglucosamine-Mutase[c]'
'Phosphoacetylglucosamine-Mutase-P[c]'
'N-ACETYL-D-GLUCOSAMINE-16-BIS-P[c]'
'CPD-9570[e]'
'THR[e]'
'CPD-17714[c]'
'CPD-17701[c]'
'CPD-8607[c]'
'CPD-8608[c]'
'ALPHA-HYDROXY-915-DIOXOPROSTANOATE[c]'
'HYDROXY-915-DIOXOPROSTA-13-ENOATE[c]'
'CPD-308[c]'
'CPD-15524[c]'
'CPD-15523[c]'
'CPD-33[c]'
'DEHYDFUC-CPD[c]'
'CPD-15566[c]'
'TAGATOSE-6-PHOSPHATE[c]'
'CPD-1118[c]'
'CPD-183[c]'
'TARTRATE[c]'
'CPD-66[c]'
'CPD-7620[c]'
'CPD-7619[c]'
'CPD-10799[c]'
'CPD-10782[c]'
'THIOHYDROXIMATE-O-SULFATES[c]'
'CPD-558[c]'
'Alkenyl-Thiohydroximate-O-Sulfates[c]'
'EPITHIONITRILES[c]'
'CPD-10800[c]'
'CDPDIACYLGLYCEROL[c]'
'L-1-PHOSPHATIDYL-GLYCEROL[c]'
'CARDIOLIPIN[c]'
'CPD-10801[c]'
'S-RETICULINE[c]'
'12-DEHYDRORETICULINIUM[c]'
'ACROLEIN[c]'
'CPD-7600[c]'
'CPD-8847[c]'
'MEK[c]'
'CPD-13218[c]'
'CPD-13220[c]'
'CPD-13222[c]'
'TRP[e]'
'Acyl-homoserine-lactones[c]'
'Acyl-homoserines[c]'
'TYR[e]'
'VAL[e]'
'CPD-12364[e]'
'Deoxy-Ribonucleoside-Monophosphates[c]'
'Maltodextrins[e]'
'MALTOSE[e]'
'MALTOTRIOSE[e]'
'BETA-TOCOPHEROL[c]'
'CONIFERYL-ALCOHOL[c]'
'CONIFERYL-ALDEHYDE[c]'
'SINAPYL-ALCOHOL[c]'
'CPD-12829[c]'
'L-arginyl-L-Glutamyl-Peptides[c]'
'ARG-tRNAs[c]'
'Proteins-With-N-Terminal-Asp[c]'
'L-arginyl-L-aspartyl-Peptides[c]'
'TTP[c]'
'L-arginyl-3-sulfino-L-alaninyl-Peptides[c]'
'N-terminal-L-cysteine-sulfinate[c]'
'N-terminal-L-cysteine-sulfonate[c]'
'SER[c]'
'L-arginyl-3-sulfo-L-alaninyl-Peptides[c]'
'N-Ac-L-methionyl-L-asparaginyl-Protein[c]'
'N-terminal-asparagine[c]'
'CPD0-2015[c]'
'L-Glutaminyl-Peptides[c]'
'L-RIBULOSE-5-P[c]'
'N-Ac-L-methionyl-L-glutaminyl-Protein[c]'
'Trans-D2-decenoyl-ACPs[c]'
'L-RIBULOSE[c]'
'UROPORPHYRINOGEN-III[c]'
'DIMETHYL-GLYCINE[c]'
'5-DEHYDROGLUCONATE[c]'
'CPD-13473[c]'
'CPD-17723[c]'
'CPD-17724[c]'
'CPD-17725[c]'
'CPD-8609[c]'
'CPD-8610[c]'
'O-SUCCINYL-L-HOMOSERINE[c]'
'L-CYSTATHIONINE[c]'
'CPD-3187[c]'
'CPD-18798[c]'
'CPD-14092[c]'
'CPD-6972[c]'
'CPD-22025[c]'
'CPD-22027[c]'
'CPD-22028[c]'
'CPD-15189[c]'
'CPD-22029[c]'
'VLC-Ceramides[c]'
'CPD-10556[c]'
'Ultra-Long-Chain-Acyl-CoAs[c]'
'ULC-Cermaides[c]'
'CPD-13612[c]'
'CPD-22033[c]'
'CPD-18797[c]'
'3-OXO-5-BETA-CHOLANATE[c]'
'57222428-ERGOSTATETRAENOL[c]'
'CPD-18825[c]'
'ISO-PROPANOL[c]'
'SACCHAROPINE[c]'
'SECOLOGANIN-CPD[c]'
'CPD-7247[c]'
'ISOVALERYL-COA[c]'
'BCAA-dehydrogenase-3MB-DH-lipoyl[c]'
'CPD-18826[c]'
'CPD-18831[c]'
'CPD-18832[c]'
'CPD-12524[c]'
'CPD-17138[c]'
'CPD-9038[c]'
'Cytochromes-C-Reduced[e]'
'CPD-4211[c]'
'CPD-15265[c]'
'Cytochromes-C-Oxidized[e]'
'CPD-15263[c]'
'TREHALOSE[c]'
'CPD-1862[c]'
'HOP-2229-ENE[c]'
'CPD-15268[c]'
'ENT-COPALYL-DIPHOSPHATE[c]'
'CPD-15260[c]'
'CPD-602[c]'
'CPD-15259[c]'
'CPD-15261[c]'
'Ergothioneine[c]'
'CPD-19154[c]'
'CPD-19161[c]'
'CPD-11571[c]'
'CPD-19157[c]'
'CPD-12101[c]'
'CPD-19148[c]'
'3-oxo-hexanoyl-ACPs[c]'
'12-apo-Carotenals[c]'
'9-cis-Epoxycarotenoids[c]'
'CPD-7279[c]'
'CPD-7280[c]'
'CPD-7196[c]'
'3-OXOADIPATE-ENOL-LACTONE[c]'
'3-KETO-ADIPATE[c]'
'CPD1F-130[c]'
'R-3-hydroxyhexanoyl-ACPs[c]'
'CPD1F-131[c]'
'D-GALACTONO-1-4-LACTONE[c]'
'CPD-155[c]'
'GALACTOSE-1P[c]'
'N-SUCCINYL-2-AMINO-6-KETOPIMELATE[c]'
'N2-SUCCINYLORNITHINE[c]'
'CPD-822[c]'
'CPD-725[c]'
'L-methionyl-tRNAfmet[c]'
'NN-dimethyl-terminal-XPK[c]'
'NNN-trimethyl-terminal-XPK[c]'
'CPD-17434[c]'
'CYTIDINE[c]'
'D-Ribofuranose[c]'
'N-terminal-XPK[c]'
'CYTOSINE[c]'
'N-terminal-PPK[c]'
'NN-dimethyl-terminal-PPK[c]'
'CPD-17428[c]'
'N-methyl-terminal-PPK[c]'
'CPD-17052[c]'
'CPD-17453[c]'
'CPD66-40[c]'
'CPD-22034[c]'
'CPD-22035[c]'
'FECOSTEROL[c]'
'CPD-9965[c]'
'CPD-22036[c]'
'CPD-16352[c]'
'URIDINE[c]'
'CPD1F-133[c]'
'HISTIDINOL[c]'
'HIS[c]'
'CPD-7390[c]'
'CPD-7392[c]'
'Deoxy-Ribonucleoside-Diphosphates[c]'
'Ribonucleoside-Diphosphates[c]'
'COPROPORPHYRINOGEN\_III[c]'
'Oxidized-NrdH-Proteins[c]'
'Reduced-NrdH-Proteins[c]'
'CPD-205[c]'
'CPD-101[c]'
'CPD-15285[c]'
'CPD0-2298[c]'
'CPD-1091[c]'
'CPD-1863[c]'
'CPD-1881[c]'
'L-4-HYDROXYGLUTAMATE\_SEMIALDEHYDE[c]'
'3-DEHYDRO-SHIKIMATE[c]'
'UDP-D-GALACTO-14-FURANOSE[c]'
'L-Amino-Acids[c]'
'5-OXOPROLINE[c]'
'5-L-GLUTAMYL-PEPTIDE[c]'
'5-L-GLUTAMYL-AMINO-ACID[c]'
'Hex-2-enoyl-ACPs[c]'
'CPD-17455[c]'
'CPD-19150[c]'
'CPD-17053[c]'
'CPD-19151[c]'
'CPD-17457[c]'
'CPD-3631[c]'
'CPD-19153[c]'
'CPD-17458[c]'
'CPD-3944[c]'
'CPD-3942[c]'
'CPD-8678[c]'
'BETA-D-FRUCTOSE[c]'
'3-SULFINYL-PYRUVATE[c]'
'Kanamycin-3-phosphates[c]'
'Kanamycins[c]'
'DE-O-GLUCONATE[c]'
'DE-O-K-GLUCONATE[c]'
'CPD-16353[c]'
'CPD-22039[c]'
'CPD-14293[c]'
'CPD-22040[c]'
'D-3-HYDROXYACYL-COA[c]'
'CPD-22041[c]'
'CPD-14261[c]'
'CPD-10662[c]'
'CPD-14269[c]'
'CPD-14262[c]'
'CPD-22043[c]'
'CPD-15363[c]'
'3-oxo-stearoyl-ACPs[c]'
'HOMO-CIT[c]'
'HOMO-CIS-ACONITATE[c]'
'EPISTEROL[c]'
'CPD-307[c]'
'THIOMORPHOLINE-3-CARBOXYLATE[c]'
'34-DEHYDRO-14-THIOMORPHOLINE-3-CARBOXY[c]'
'R-RETICULINE[c]'
'CPD-2022[c]'
'CPD-102[c]'
'L-ERYTHRO-4-HYDROXY-GLUTAMATE[c]'
'CPD-637[c]'
'CPD-112[c]'
'CPD-15978[c]'
'CPD-15361[c]'
'L-LACTATE[e]'
'CPD-1072[c]'
'MAL[e]'
'1-3-beta-D-Glucans[e]'
'CPD-9446[e]'
'2-KETOGLUTARATE[e]'
'P-AMINO-BENZOATE[e]'
'CPD-1302[e]'
'6-DEMETHYLSTERIGMATOCYSTIN[e]'
'Red-Thioredoxin[e]'
'ACP[e]'
'SHIKIMATE[c]'
'CPDQT-273[c]'
'CPD-12140[c]'
'ARSENATE[c]'
'CPD1G-2[c]'
'CPD-12152[c]'
'CPD-12156[c]'
'NICOTINAMIDE\_RIBOSE[c]'
'Pyrimidine-Nucleosides[c]'
'Pyrimidine-Bases[c]'
'CPD-17614[c]'
'Vernolates[c]'
'2-Acylglycero-Phosphocholines[c]'
'NARINGIN[c]'
'NARINGENIN-7-O-BETA-D-GLUCOSIDE[c]'
'CPD-7075[c]'
'HYPOTAURINE[c]'
'CPD-7073[c]'
'FRU1P[c]'
'BCAA-dehydrogenase-lipoyl[c]'
'5-PHOSPHO-RIBOSYL-GLYCINEAMIDE[c]'
'5-P-RIBOSYL-N-FORMYLGLYCINEAMIDE[c]'
'CPD-14925[c]'
'CPD-10793[c]'
'3-P-HYDROXYPYRUVATE[c]'
'Hexanoyl-ACPs[c]'
'CPD-15364[c]'
'CPD-22044[c]'
'CPD-14268[c]'
'CPD-22045[c]'
'CPD-22048[c]'
'CPD-14271[c]'
'CPD-22050[c]'
'CPD-10283[c]'
'CPD-18491[c]'
'CPD-782[c]'
'Nucleoside-Diphosphates[c]'
'UDP-SULFOQUINOVOSE[c]'
'CPD-13171[c]'
'apo-Peptidyl-carrier-proteins[c]'
'ACET[e]'
'L-2-AMINOPENTANOIC-ACID[c]'
'ADENINE[e]'
'ADENOSINE[e]'
'TREHALOSE[e]'
'CPD-15699[e]'
'LINOLENIC\_ACID[e]'
'AMMONIUM[e]'
'ETR-Quinols[e]'
'ETF-Oxidized[e]'
'ANTHRANILATE[e]'
'CPD-7400[c]'
'CYANURIC-ACID[c]'
'GLUTAMYL-GLX-TRNAS[c]'
'CPD-700[c]'
'GLX-tRNAs[c]'
'CPD-1075[c]'
'CPD-7409[c]'
'Phytosphingosines[c]'
'Very-Long-Chain-Phytoceramides[c]'
'CPD-15362[c]'
'CPD-17399[c]'
'CPD-17400[c]'
'CPD-15368[c]'
'CPD-3736[c]'
'Ferrihemoglobins[c]'
'Ferrohemoglobins[c]'
'CPD-19144[c]'
'CPD-19170[c]'
'AMINOMETHYLDIHYDROLIPOYL-GCVH[c]'
'Deoxy-Ribonucleoside-Triphosphates[c]'
'DIHYDROLIPOYL-GCVH[c]'
'3-KETOLACTOSE[c]'
'CPD-17496[c]'
'CPD-1242[c]'
'P-NITROPHENOL[c]'
'CPD-9000[c]'
'CPD-14274[c]'
'CPD-14273[c]'
'CPD-13381[c]'
'CPD-14275[c]'
'CPD-14736[c]'
'CPD-19168[c]'
'ANTHRANILATE[c]'
'MAP-Kinase-L-Tyr[c]'
'MAP-Kinase-L-Phosphotyrosine[c]'
'CPD-14276[c]'
'3-HYDROXY-L-KYNURENINE[c]'
'CPD-14277[c]'
'CELLULOSE[e]'
'CPD-321[c]'
'CPD-14133[c]'
'CPD-4462[c]'
'CPD-22265[c]'
'1-Acyl-sn-glycerols[c]'
'CPD-19186[c]'
'Protein-S-methyl-L-cysteine[c]'
'a-thymine-in-DNA[c]'
'CPD0-2500[c]'
'Protein-Red-Disulfides[c]'
'L-methionyl-L-asparaginyl-Protein[c]'
'Protein-Ox-Disulfides[c]'
'L-methionyl-L-glutaminyl-Protein[c]'
'N-Ac-L-methionyl-L-aspartyl-Protein[c]'
'Alkyl-acetyl-glycero-phosphocholines[c]'
'1-Alkyl-sn-glycero-3-phosphocholines[c]'
'CPD-19167[c]'
'CPD-1092[c]'
'3-OXOPIMELOYL-COA[c]'
'CPD-459[c]'
'Phytoceramides[c]'
'Alpha-hydroxyphytoceramides[c]'
'CPD-15369[c]'
'CPD-15370[c]'
'HYDRPHENYLAC-CPD[c]'
'CPD-15366[c]'
'CPD-15373[c]'
'CPD-12601[c]'
'Guanine37-in-tRNAPhe[c]'
'tRNAPhe-Containing-N1-Methylguanine-37[c]'
'3-UREIDO-ISOBUTYRATE[c]'
'CPD-471[c]'
'CPD-209[c]'
'C3[c]'
'DIHYDRO-THYMINE[c]'
'CH3-MALONATE-S-ALD[c]'
'CHITIN[e]'
'CPD-13545[e]'
'Chitosan[e]'
'CHOLINE[e]'
'CPD-355[e]'
'CPD-3617[e]'
'BETA-D-FRUCTOSE[e]'
'CPD-182[c]'
'CPD-14278[c]'
'CPD-181[c]'
'L-methionyl-L-aspartyl-Protein[c]'
'N-Ac-L-methionyl-L-glutamyl-Protein[c]'
'CPD3DJ-82[c]'
'L-methionyl-L-glutamyl-Protein[c]'
'L-methionyl-L-leucyl-Protein[c]'
'N-Ac-L-methionyl-L-leucyl-Protein[c]'
'Sphinga-4E-8E-dienine-Ceramides[c]'
'D-BETA-D-HEPTOSE-17-DIPHOSPHATE[c]'
'9-Methyl-sphing-4-8-dienine-ceramides[c]'
'L-methionyl-L-isoleucyl-Protein[c]'
'N-Ac-L-methionyl-L-isoleucyl-Protein[c]'
'L-methionyl-L-phenylalanyl-Protein[c]'
'ADP-D-GLYCERO-D-MANNO-HEPTOSE[c]'
'N-Ac-L-methionyl-L-phenylalanyl-Protein[c]'
'Delta5-Delta7-Steroids[c]'
'L-methionyl-L-tryptophanyl-Protein[c]'
'N-Ac-L-methionyl-L-tryptophanyl-Protein[c]'
'Delta7-Steroids[c]'
'L-methionyl-L-tyrosinyl-Protein[c]'
'CPD0-2474[c]'
'2-D-THREO-HYDROXY-3-CARBOXY-ISOCAPROATE[c]'
'UNDECAPRENYL-DIPHOSPHATE[c]'
'Primary-Aliphatic-Amides[c]'
'CPD-19172[c]'
'N5-Formyl-THF-Glu-N[c]'
'CPD-19171[c]'
'2-AMINOMUCONATE\_SEMIALDEHYDE[c]'
'2-AMINO-MUCONATE[c]'
'CPD-19169[c]'
'CPD-444[c]'
'CPD-10172[c]'
'CPD-1061[c]'
'CPD-9451[c]'
'CPD-10175[c]'
'CPD-10175[e]'
'CPD-10171[c]'
'ASP-tRNAs[c]'
'CPD-10176[c]'
'Charged-ASP-tRNAs[c]'
'CPD-4205[c]'
'CPD-4586[c]'
'CPD-302[c]'
'CPD-17312[c]'
'CPD-21823[c]'
'TRANS-D2-ENOYL-COA[c]'
'CPD-21826[c]'
'Trans-3-enoyl-CoAs[c]'
'CPD-21825[c]'
'GAMMA-LINOLENOYL-COA[c]'
'CPD-21816[c]'
'CPD-21828[c]'
'CPD-21817[c]'
'CPD-21814[c]'
'Long-Chain-Fatty-Acids[c]'
'ARACHIDONYL-COA[c]'
'CPD-21830[c]'
'Sterols[c]'
'TAURINE[c]'
'Charged-CYS-tRNAs[c]'
'VANILLATE[c]'
'CPD-15192[c]'
'CPD-15216[c]'
'Sterol-3-beta-D-glucosides[c]'
'RH-Group[c]'
'Long-chain-cholesterol-esters[c]'
'CPD-629[c]'
'CPD-556[c]'
'CPD-255[c]'
'CPD-17387[c]'
'CPD-17386[c]'
'CPD-17388[c]'
'CPD-14165[c]'
'CPD-14447[c]'
'4-FUMARYL-ACETOACETATE[c]'
'CPD-17355[c]'
'CPD-10244[c]'
'Glycerolipids[c]'
'INDOLE[c]'
'MONOMETHYL-ESTER-OF-TRANS-ACONITATE[c]'
'Behenoyl-ACPs[c]'
'3-oxo-lignoceroyl-ACPs[c]'
'R-3-hydroxylignoceroyl-ACPs[c]'
'trans-delta2-lignoceroyl-ACPs[c]'
'4-HYDROXYPHENYLACETATE[c]'
'NICOTINATE\_NUCLEOTIDE[c]'
'CPD-110[c]'
'VANILLIN[c]'
'CPD-581[c]'
'DIMETHYLAMINE[c]'
'CPD-4587[c]'
'CPD-4588[c]'
'CPD-10177[c]'
'CPD-402[c]'
'N-METHYLANTHRANILOYL-COA[c]'
'Long-Chain-Steryl-Esters[c]'
'3-Phosphomonucleotides[c]'
'CPD-22005[c]'
'CPD-22004[c]'
'3-OCTAPRENYL-4-HYDROXYBENZOATE[c]'
'CPD-22006[c]'
'CPD-22003[c]'
'3-HYDROXYBENZOATE[c]'
'CPD-22008[c]'
'CPD-22009[c]'
'3-MERCAPTO-PYRUVATE[c]'
'IMP[c]'
'L-ASPARTATE-SEMIALDEHYDE[c]'
'L-BETA-ASPARTYL-P[c]'
'CPD-5881[c]'
'CPD-14202[c]'
'TETRADEHYDROACYL-COA[c]'
'2-hydroxyacyl-glutathiones[c]'
'2-Hydroxy-carboxylates[c]'
'CPD-8050[c]'
'CPD-14808[c]'
'CPD0-934[c]'
'CPD-15237[c]'
'Demethylmenaquinols[c]'
'Menaquinols[c]'
'DNA-containing-a-Apyrimidinic-Sites[c]'
'N-ACETYL-5-METHOXY-TRYPTAMINE[c]'
'5-HYDROXYISOURATE[c]'
'CPD-12014[c]'
'N-ACETYL-SEROTONIN[c]'
'CPD-45[c]'
'STIPIT-CPD[c]'
'STRICTOSIDINE[c]'
'TRYPTAMINE[c]'
'2-Phenyloxirane[c]'
'PHENYLACETALDEHYDE[c]'
'CPD-8900[c]'
'CPD-17282[c]'
'Protein-L-lysine[c]'
'CPD-17381[c]'
'44-DIMETHYL-5ALPHA-CHOLEST-7-EN-3BET[c]'
'CPD-17392[c]'
'CPD-5846[c]'
'CPD1F-132[c]'
'CPD1F-95[c]'
'CPD-692[c]'
'DNA-with-Uracils[c]'
'CPD-787[c]'
'CPD-786[c]'
'PHOSPHORYL-ETHANOLAMINE[c]'
'CPD3DJ-11366[c]'
'SPHINGOSINE[c]'
'ISOCHORISMATE[c]'
'CHORISMATE[c]'
'CPD-22010[c]'
'CPD-22007[c]'
'CPD-7214[c]'
'CPD-7087[c]'
'3S-CITRYL-COA[c]'
'CPD-22012[c]'
'CPD-22013[c]'
'CPD-7221[c]'
'CPD-7222[c]'
'Retinols[c]'
'CPD-22014[c]'
'A-3-OXO-ACID[c]'
'CPD-10189[c]'
'CPD-10188[c]'
'B-KETOACYL-ACP[c]'
'OH-ACYL-ACP[c]'
'CPD-19163[c]'
'CARBAMYUL-L-ASPARTATE[c]'
'CPD-19159[c]'
'CPD-216[c]'
'Protein-L-serines[c]'
'Protein-D-serines[c]'
'CYSTINE[c]'
'THIOCYSTEINE[c]'
'CPD-15240[c]'
'Alpha-6-alpha-14-glucans[c]'
'Sulfur-Carrier-Proteins-ThiI[c]'
'Sulfurylated-ThiI[c]'
'CPD-578[c]'
'N-SUCCINYLLL-2-6-DIAMINOPIMELATE[c]'
'LL-DIAMINOPIMELATE[c]'
'CPD-12015[c]'
'CPD-12017[c]'
'CPD1F-128[c]'
'CPD-17395[c]'
'BCAA-dehydrogenase-2MP-DH-lipoyl[c]'
'CPD-17390[c]'
'CPD-17389[c]'
'CPD-17391[c]'
'CPD-17393[c]'
'CPD-17394[c]'
'ISOPENICILLIN-N[c]'
'5-HYDROXY-TRYPTOPHAN[c]'
'SEROTONIN[c]'
'CPD-7224[c]'
'CPD-22011[c]'
'CAPSORUBIN[c]'
'CPD-22016[c]'
'CPD-22017[c]'
'CAPSANTHIN[c]'
'CPD-22018[c]'
'CPD-22015[c]'
'CPD-22021[c]'
'PENICILLIN-N[c]'
'D-SERINE[c]'
'S-3-HYDROXYBUTANOYL-COA[c]'
'CPD-650[c]'
'ADENOSYL-P4[c]'
'ADP-L-GLYCERO-D-MANNO-HEPTOSE[c]'
'DEOXYURIDINE[c]'
'CPD-19160[c]'
'Acetoacetyl-ACPs[c]'
'CPD-10269[c]'
'CPD-19162[c]'
'Bet...

-------------------------------------------------------------------------------

The chemical element **Hg** is present in the empirical formula of **5 metabolites**

'CPD-21194[c]'
'CPD-21193[c]'
'CPD-21195[c]'
'HG0[c]'
'HG+2[c]'

-------------------------------------------------------------------------------

The chemical element **I** is present in the empirical formula of **17 metabolites**

'CPD-11398[c]'
'L-THYROXINE[c]'
'LIOTHYRONINE[c]'
'CPD-11400[c]'
'CPD-11401[c]'
'CPD-11402[c]'
'CPD-11407[c]'
'CPD-11408[c]'
'CPD-11409[c]'
'CPD-11403[c]'
'CPD-11411[c]'
'CPD-11404[c]'
'CPD-11412[c]'
'CPD-11410[c]'
'CPD-387[c]'
'CPD-12288[c]'
'CPD-7676[c]'

-------------------------------------------------------------------------------

The chemical element **K** is present in the empirical formula of **2 metabolites**

'K+[e]'
'K+[c]'

-------------------------------------------------------------------------------

The chemical element **Mg** is present in the empirical formula of **2 metabolites**

'MG+2[e]'
'MG+2[c]'

-------------------------------------------------------------------------------

The chemical element **Mn** is present in the empirical formula of **2 metabolites**

'MN+2[e]'
'MN+2[c]'

-------------------------------------------------------------------------------

The chemical element **Mo** is present in the empirical formula of **3 metabolites**

'CPD-3[c]'
'CPD-8123[c]'
'CPD-8124[c]'

-------------------------------------------------------------------------------

The chemical element **N** is present in the empirical formula of **2946 metabolites**

'CO-A[c]'
'OLEOYL-COA[c]'
'CTP[c]'
'CDP[c]'
'NADP[c]'
'NADPH[c]'
'CPD-12575[c]'
'ACETYL-COA[c]'
'GLUCOSAMINE-1P[c]'
'N-ACETYL-D-GLUCOSAMINE-1-P[c]'
'ADENOSINE[c]'
'ATP[c]'
'Odd-Saturated-Fatty-Acyl-CoA[c]'
'INOSINE[c]'
'AMMONIUM[c]'
'AMP[c]'
'ADP[c]'
'NAD[c]'
'NADH[c]'
'2E-5Z-tetradeca-2-5-dienoyl-ACPs[c]'
'PHOSPHORYL-CHOLINE[c]'
'5Z-tetradec-5-enoyl-ACPs[c]'
'CPD0-1122[c]'
'CPD0-1123[c]'
'MALONYL-ACP[c]'
'Chondroitin-N-acetyl-galactosamines[c]'
'7Z-3-oxo-hexadec-7-enoyl-ACPs[c]'
'ACP[c]'
'CHONDROITIN-4-SULFATE[c]'
'Heparan-sulfate-L-iduronate[c]'
'Heparan-sulfate-L-IdoA-2S[c]'
'Heparan-NAc-Glc-6S[c]'
'FERRICYTOCHROME-B5[c]'
'CPD-8091[c]'
'FERROCYTOCHROME-B5[c]'
'CPD-8092[c]'
'CPD-2181[c]'
'LINOLENOYL-COA[c]'
'CPD-2182[c]'
'CPD-8088[c]'
'CPD-8093[c]'
'N-ALPHA-ACETYLORNITHINE[c]'
'L-ORNITHINE[c]'
'GLT[c]'
'CPD-469[c]'
'CPD-12384[c]'
'CPD-12385[c]'
'NADH-P-OR-NOP[c]'
'S-ADENOSYLMETHIONINE[c]'
'CPD-12388[c]'
'ADENOSYL-HOMO-CYS[c]'
'CPD-12387[c]'
'CPD-12390[c]'
'CPD-12391[c]'
'CPD-12393[c]'
'CPD-12139[c]'
'CPD-19502[c]'
'CPD-19504[c]'
'CPD-19503[c]'
'CPD-14673[c]'
'Ox-NADPH-Hemoprotein-Reductases[c]'
'N-6-AMINOHEXANOYL-6-AMINOHEXANOATE[c]'
'CPD-884[c]'
'CPD-10689[c]'
'N1-ACETYLSPERMINE[c]'
'CPD-11271[c]'
'CPD-313[c]'
'UDP-GLUCURONATE[c]'
'CPD-11398[c]'
'UDP[c]'
'L-THYROXINE[c]'
'LIOTHYRONINE[c]'
'CPD-11400[c]'
'LYS[c]'
'8-AMINO-7-OXONONANOATE[c]'
'DIAMINONONANOATE[c]'
'CPD-208[c]'
'PROTEIN-LIPOYLLYSINE[c]'
'MET[c]'
'CH33ADO[c]'
'Octanoylated-Gcv-H[c]'
'CPD-196[c]'
'a-pyruvate-dehydrogenase-E2-protein-Nsup[c]'
'pyruvate-dehydrogenase-E2-lipoyl-carrier[c]'
'DEOXYINOSINE[c]'
'HYPOXANTHINE[c]'
'DNA-deoxycytidine-thymidine-dimer[c]'
'DNA-Cytidines[c]'
'DNA-thymidines[c]'
'GLUTARYL-COA[c]'
'MALONYL-COA[c]'
'HEXANOYL-COA[c]'
'CPD-14687[c]'
'CPD-14615[c]'
'ETF-Oxidized[c]'
'GLUTACONYL-COA[c]'
'ETF-Reduced[c]'
'CPD-18[c]'
'Heparan-NAc-Glc[c]'
'NAD-P-OR-NOP[c]'
'56-Dihydrouracil17-in-tRNAs[c]'
'Uracil17-in-tRNAs[c]'
'56-Dihydrouracil20-in-tRNAs[c]'
'Uracil20-in-tRNAs[c]'
'Long-Chain-234-Saturated-acyl-CoAs[c]'
'56-Dihydrouracil47-in-tRNAs[c]'
'Uracil47-in-tRNAs[c]'
'Long-Chain-Trans-23-Dehydroacyl-CoA[c]'
'biotin-L-lysine-in-BCCP-dimers[c]'
'carboxybiotin-L-lysine-in-BCCP-dimers[c]'
'CPD-8089[c]'
'CPD-8090[c]'
'BCCP-L-lysine[c]'
'BIOTIN[c]'
'BCCP-biotin-L-lysine[c]'
'1-183-2-183-SN-GLYCEROL-PHOSPHOCHOLINE[c]'
'3R-7Z-3-hydroxy-hexadec-7-enoyl-ACPs[c]'
'2E-7Z-hexadeca-2-7-dienoyl-ACPs[c]'
'7Z-hexadec-7-enoyl-ACPs[c]'
'9Z-3-oxo-octadec-9-enoyl-ACPs[c]'
'CPD0-1158[c]'
'CPD0-1162[c]'
'Oxo-glutarate-dehydrogenase-DH-lipoyl[c]'
'CPD0-1163[c]'
'CPD-12394[c]'
'CPD-12397[c]'
'CPD-12396[c]'
'CPD-12399[c]'
'CPD-12400[c]'
'CPD-12402[c]'
'CPD-12403[c]'
'CPD-12405[c]'
'CPD-12406[c]'
'BCAA-dehydrogenase-DH-lipoyl[c]'
'PHENYLACETOTHIOHYDROXIMATE[c]'
'CPD-12607[c]'
'GAMMA-BUTYROBETAINE[c]'
'CPD-3462[c]'
'SPERMIDINE[c]'
'TRIMETHYLAMINE[c]'
'Glucosyl-ceramides[c]'
'N-ACETYLNEURAMINATE[c]'
'N-acetyl-D-mannosamine[c]'
'Ceramides[c]'
'PROTEIN-C-TERMINAL-S-ETC-CYSTEINE[c]'
'PROTEIN-C-TERMINAL-S-FARNESYL-L-CYSTEINE[c]'
'DIHYDROLIPOAMIDE[c]'
'LIPOAMIDE[c]'
'CPD-11401[c]'
'CPD-11402[c]'
'PALMITYL-COA[c]'
'CPD-17621[c]'
'CROTONYL-COA[c]'
'GLUTATHIONE[c]'
'OXIDIZED-GLUTATHIONE[c]'
'R-4-PHOSPHOPANTOTHENOYL-L-CYSTEINE[c]'
'PANTETHEINE-P[c]'
'Pyruvate-dehydrogenase-lipoate[c]'
'a-2-oxoglutarate-dehydrogenase-E2-protei[c]'
'2-oxoglutarate-dehydrogenase-E2-lipoyl-c[c]'
'Oxo-glutarate-dehydrogenase-lipoyl[c]'
'PHTYOSPHINGOSINE-1-P[c]'
'Rhodoquinols[c]'
'Rhodoquinones[c]'
'DEPHOSPHO-COA[c]'
'L-ALPHA-ALANINE[c]'
'CPD-630[c]'
'CPD-18077[c]'
'Glc2Man9GlcNAc2-proteins[c]'
'CPD-14704[c]'
'Chitosan[c]'
'Chitosan-fragments[c]'
'CPD-14705[c]'
'PHOSPHORIBOSYL-CARBOXY-AMINOIMIDAZOLE[c]'
'DETHIOBIOTIN[c]'
'L-ASPARTATE[c]'
'CPD-14706[c]'
'GLY[c]'
'P-RIBOSYL-4-SUCCCARB-AMINOIMIDAZOLE[c]'
'CPD-19179[c]'
'GTP[c]'
'Guanine37-in-tRNA[c]'
'tRNA-Containing-N1-Methylguanine-37[c]'
'TMP[c]'
'ADENOSINE5TRIPHOSPHO5ADENOSINE[c]'
'DIHYDROFOLATE-GLU-N[c]'
'Guanine9-in-tRNA[c]'
'tRNA-Containing-N1-Methylguanine-9[c]'
'carbo-me-ur-34-tRNA[c]'
'5-2-me-oxy-2-oxo-et-ur-34-tRNA[c]'
'PRECURSOR-Z[c]'
'CPD-4[c]'
'MPT-Synthase-small-subunits[c]'
'2-METHYL-3-HYDROXY-BUTYRYL-COA[c]'
'CPD-1083[c]'
'Thiocarboxylated-MPT-synthases[c]'
'tRNA-Adenine-58[c]'
'tRNA-Containing-N1-MethylAdenine-58[c]'
'BLASTICIDIN-S[c]'
'DEAMINOHYDROXYBLASTICIDIN-S[c]'
'Adenine57-Adenine58-tRNAs[c]'
'N1-MeAdenine57-MeAdenine58-tRNAs[c]'
'CPD-8122[c]'
'3R-9Z-3-hydroxy-octadec-9-enoyl-ACPs[c]'
'2E-9Z-octadeca-2-9-dienoyl-ACPs[c]'
'CPD-1861[c]'
'CPD-12449[c]'
'URACIL[c]'
'PSEUDOURIDINE-5-P[c]'
'CPD66-21[c]'
'LEUKOTRIENE-C4[c]'
'Amino-Acids-20[c]'
'5-L-GLUTAMYL-L-AMINO-ACID[c]'
'7E9E11Z14Z-5S6R-6-CYSTEIN-S-YL[c]'
'Long-Chain-Acyl-CoAs[c]'
'L-ALLO-THREONINE[c]'
'AMINO-RIBOSYLAMINO-1H-3H-PYR-DIONE[c]'
'DNA-Cytosines[c]'
'DNA-N4-Methylcytosine[c]'
'S-SCOULERINE[c]'
'CPD-239[c]'
'4-P-PANTOTHENATE[c]'
'S-TETRAHYDROCOLUMBAMINE[c]'
'3-SULFINOALANINE[c]'
'L-CYSTEATE[c]'
'6-O-METHYLNORLAUDANOSOLINE[c]'
'CPD-15799[c]'
'Myelin-N-o-methyl-arginines[c]'
'R-3-Hydroxypalmitoyl-ACPs[c]'
'PAPS[c]'
'3-5-ADP[c]'
'CPD-11407[c]'
'CPD-11408[c]'
'3-oxo-palmitoyl-ACPs[c]'
'CYS[c]'
'CMP[c]'
'DGDP[c]'
'Carboxyadenylated-MPT-synthases[c]'
'L-Cysteine-Desulfurase-persulfide[c]'
'Cysteine-Desulfurase-L-cysteine[c]'
'CHITIN[c]'
'Chitodextrins[c]'
'ITP[c]'
'IDP[c]'
'Poly-ADP-Riboses[c]'
'ADENOSINE\_DIPHOSPHATE\_RIBOSE[c]'
'GLUCOSAMINE[c]'
'Peptidoglycans[c]'
'NAcMur-Peptide-Undecaprenols[c]'
'N-acetyl-D-glucosamine[c]'
'CPD-8123[c]'
'Mercapturates[c]'
'S-Substituted-L-Cysteines[c]'
'L-GAMMA-GLUTAMYLCYSTEINE[c]'
'Malonyl-acp-methyl-ester[c]'
'3-Ketoglutaryl-ACP-methyl-ester[c]'
'ILE[c]'
'PROPIONYL-COA[c]'
'D-METHYL-MALONYL-COA[c]'
'LEU[c]'
'FADH2[c]'
'FAD[c]'
'Folates[c]'
'P-COUMAROYL-COA[c]'
'CAFFEOYL-COA[c]'
'CARNITINE[c]'
'CPD-19737[c]'
'BUTYRYL-COA[c]'
'CPD-19738[c]'
'TETRADECANOYL-COA[c]'
'CPD-19740[c]'
'CPD-10267[c]'
'CPD-19741[c]'
'STEAROYL-COA[c]'
'CPD-19742[c]'
'Oleoyl-ACPs[c]'
'ACETYL-ACP[c]'
'11Z-3-oxo-icos-11-enoyl-ACPs[c]'
'Cytidine-34-tRNAmet[c]'
'Elongator-tRNAMet-acetylcytidine[c]'
'Red-NADPH-Hemoprotein-Reductases[c]'
'DIMETHYL-D-RIBITYL-LUMAZINE[c]'
'Myelin-L-arginines[c]'
'S-NORCOCLAURINE[c]'
'S-COCLAURINE[c]'
'DIHYDROSIROHYDROCHLORIN[c]'
'CPD-642[c]'
'METHIONINE-SYNTHASE-METHYLCOBALAMIN[c]'
'Methionine-synthase-cob-II-alamins[c]'
'2-Hexadecenoyl-ACPs[c]'
'Palmitoyl-ACPs[c]'
'METHYLENE-THF-GLU-N[c]'
'5-10-METHENYL-THF-GLU-N[c]'
'L-DIHYDROXY-PHENYLALANINE[c]'
'Release-factor-L-glutamine[c]'
'Release-factor-N5-Methyl-L-glutamine[c]'
'CPD-15896[c]'
'CPD-17487[c]'
'DGTP[c]'
'DEOXYGUANOSINE[c]'
'CPD-664[c]'
'DELTA1-PYRROLINE\_2-CARBOXYLATE[c]'
'CPD-8124[c]'
'CPD-8155[c]'
'CPD-7061[c]'
'4-AMINO-BUTYRATE[c]'
'CPD-8157[c]'
'CPD-8158[c]'
'TYR[c]'
'Peptides-holder[c]'
'CPD0-2244[c]'
'CPD0-2123[c]'
'5-METHYL-THF-GLU-N[c]'
'FORMYL-THF-GLU-N[c]'
'THF[c]'
'LAUROYLCOA-CPD[c]'
'LYS-tRNAs[c]'
'CPD-19743[c]'
'Charged-LYS-tRNAs[c]'
'CHOLINE[c]'
'CPD-7682[c]'
'4-HYDROXY-BUTYRYL-COA[c]'
'OH-CROTONYL-COA[c]'
'3-Hydroxyglutaryl-ACP-methyl-ester[c]'
'3-P-SERINE[c]'
'CPD-19754[c]'
'ECTOINE[c]'
'CPD-19757[c]'
'CPD-19760[c]'
'2-Lysophosphatidylcholines[c]'
'Enoylglutaryl-ACP-methyl-esters[c]'
'L-1-GLYCERO-PHOSPHORYLCHOLINE[c]'
'VAL[c]'
'PYRROLINE-HYDROXY-CARBOXYLATE[c]'
'4-HYDROXY-L-PROLINE[c]'
'ACYL-COA[c]'
'3R-11Z-3-hydroxy-icos-11-enoyl-ACPs[c]'
'2E-11Z-icosa-2-11-dienoyl-ACPs[c]'
'11Z-icos-11-enoyl-ACPs[c]'
'CYS-tRNAs[c]'
'ACETYLSERINE[c]'
'L-1-PHOSPHATIDYL-ETHANOLAMINE[c]'
'CPD-10260[c]'
'CPD-10261[c]'
'CPD-10262[c]'
'THF-GLU-N[c]'
'DIHYDRO-NEO-PTERIN[c]'
'DIHYDRONEOPTERIN-P3[c]'
'DIHYDROFOLATE[c]'
'Stearoyl-ACPs[c]'
'LysW-L-glutamate[c]'
'LysW-L-glutamate-5-phosphate[c]'
'LysW-L-glutamate-5-semialdehyde[c]'
'CPD-11444[c]'
'COPROPORPHYRINOGEN\_I[c]'
'LysW-L-ornithine[c]'
'CPD-15900[c]'
'3-HYDROXYPIMELYL-COA[c]'
'CPD-7733[c]'
'CPD-15913[c]'
'NA+[c]'
'Cis-Delta5-dodecenoyl-ACPs[c]'
'Charged-GLT-tRNAs[c]'
'GLT-tRNAs[c]'
'DOPAMINE[c]'
'Saturated-Fatty-Acyl-CoA[c]'
'NOREPINEPHRINE[c]'
'L-EPINEPHRINE[c]'
'DCDP[c]'
'DCTP[c]'
'CPD-12336[c]'
'CPD-14468[c]'
'E-11-TETRADECENOYL-COA[c]'
'CPD-17814[c]'
'CPD-17813[c]'
'CPD-17815[c]'
'3-OXOPALMITOYL-COA[c]'
'CPD-17464[c]'
'tRNA-Containing-N2-Dimethylgua-26-Gua27[c]'
'tRNA-Containing-N2-Methylgua-26-Gua27[c]'
'tRNA-Containing-N2-Dimetgua-26-MeGua27[c]'
'tRNA-Containing-N2-DiMeGua-26-DiMeGua27[c]'
'Guanine26-Guanine27-in-tRNAs[c]'
'Dodecanoyl-ACPs[c]'
'3-oxo-myristoyl-ACPs[c]'
'CPD-15684[c]'
'CPD-15685[c]'
'CPD-19273[c]'
'CPD-15686[c]'
'CPD-15687[c]'
'CPD-15688[c]'
'CPD-15689[c]'
'CPD-15690[c]'
'CPD-15692[c]'
'CPD-15691[c]'
'IMIDAZOLE\_ACETALDEHYDE[c]'
'CPD-14465[c]'
'CPD-14459[c]'
'CPD-14466[c]'
'CPD-14464[c]'
'CPD-14467[c]'
'4-IMIDAZOLEACETATE[c]'
'CPD-12358[c]'
'CPD-14471[c]'
'ACETOACETYL-COA[c]'
'L-GLUTAMATE\_GAMMA-SEMIALDEHYDE[c]'
'PRO[c]'
'GUANOSINE[c]'
'GUANINE[c]'
'CPD0-1065[c]'
'CADAVERINE[c]'
'5-METHYLTHIOADENOSINE[c]'
'ALPHA-L-GLUTAMYL-PHOSPHATE[c]'
'S-ADENOSYLMETHIONINAMINE[c]'
'CARBAMATE[c]'
'BENZOYLCOA[c]'
'CPD-20052[c]'
'CPD-20051[c]'
'DCMP[c]'
'DUMP[c]'
'Thiopurine-Methylethers[c]'
'Thiopurines[c]'
'Red-Thioredoxin[c]'
'Ox-Thioredoxin[c]'
'GDP-MANNOSE[c]'
'GDP-L-GALACTOSE[c]'
'2-3-CARBOXY-3-AMINOPROPYL-L-HISTIDINE[c]'
'DTDP-DEOH-DEOXY-GLUCOSE[c]'
'CPD-13952[c]'
'2-3-CARBOXY-3-METHYLAMMONIOPROPYL-L-[c]'
'CPD-9326[c]'
'eEF-2-Histidines[c]'
'CPD-17870[c]'
'3-carboxy-3-dimethylammonio-propyl-L-his[c]'
'DIPHTINE[c]'
'CPD-17877[c]'
'CPD-17876[c]'
'CPD-259[c]'
'CPD-9777[c]'
'CPD-9775[c]'
'CPD-401[c]'
'CPD-1823[c]'
'B-ALANINE[c]'
'XANTHOSINE-5-PHOSPHATE[c]'
'XANTHINE[c]'
'URATE[c]'
'CPD-1103[c]'
'Cytochromes-B-Oxidized[c]'
'Cytochromes-B-Reduced[c]'
'FMNH2[c]'
'FMN[c]'
'T2-DECENOYL-COA[c]'
'CPD-12777[c]'
'DUTP[c]'
'D-CYSTEINE[c]'
'ACETYL-GLU[c]'
'CPD-217[c]'
'OROTIDINE-5-PHOSPHATE[c]'
'GLN[c]'
'OROTATE[c]'
'PYRIDOXAL[c]'
'PYRIDOXAL\_PHOSPHATE[c]'
'CPD0-1080[c]'
'CPD0-1081[c]'
'CPD0-1082[c]'
'2-METHYL-BUTYRYL-COA[c]'
'N-ACETYL-D-GLUCOSAMINE[c]'
'CPD0-882[c]'
'L-ALA-GAMMA-D-GLU-DAP[c]'
'D-ALANINE[c]'
'4-TRIMETHYLAMMONIOBUTANAL[c]'
'3-HYDROXY-N6N6N6-TRIMETHYL-L-LYSINE[c]'
'HCN[c]'
'CPD-17873[c]'
'HSCN[c]'
'CPD-17874[c]'
'CPD-16968[c]'
'CPD-17881[c]'
'CPD-17880[c]'
'CPD-17882[c]'
'LEU-tRNAs[c]'
'Charged-LEU-tRNAs[c]'
'Sphingoids[c]'
'Sphingoid-1-phosphates[c]'
'CPD-460[c]'
'CPD-12352[c]'
'CPD-14594[c]'
'LINAMARIN[c]'
'CPD-19388[c]'
'CPD-15277[c]'
'CYS-GLY[c]'
'GLYCYLGLYCINE[c]'
'CPD-19395[c]'
'CPD-13031[c]'
'CPD-12702[c]'
'PHENYLACETONITRILE[c]'
'INDOLE-3-ACETALDOXIME[c]'
'R-3-hydroxymyristoyl-ACPs[c]'
'XANTHOSINE[c]'
'VERY-LONG-CHAIN-FATTY-ACYL-COA[c]'
'CPD-8924[c]'
'CPD-15741[c]'
'CPD-15781[c]'
'CPD-397[c]'
'CPD-15801[c]'
'ALLO-THR[c]'
'DEOXYCYTIDINE[c]'
'BETA-CYCLOPIAZONATE[c]'
'ALPHA-CYCLOPIAZONATE[c]'
'UMP[c]'
'ACETYLCHOLINE[c]'
'PHOSPHORIBULOSYL-FORMIMINO-AICAR-P[c]'
'D-ERYTHRO-IMIDAZOLE-GLYCEROL-P[c]'
'NITRITE[c]'
'CPD-12356[c]'
'CPD-12357[c]'
'CPD-14460[c]'
'CPD-12359[c]'
'CPD0-1905[c]'
'CPD-12365[c]'
'CPD-15798[c]'
'CPD-19475[c]'
'CPD-8347[c]'
'CPD-14596[c]'
'CPD-15800[c]'
'CPD-10277[c]'
'CPD-15742[c]'
'PHOSPHATIDYLCHOLINE[c]'
'Tetradec-2-enoyl-ACPs[c]'
'DEOXYADENOSINE[c]'
'DAMP[c]'
'ADENINE[c]'
'DADP[c]'
'AICAR[c]'
'Charged-THR-tRNAs[c]'
'THR[c]'
'CPD-35[c]'
'D-THREONINE[c]'
'AMINO-OXOBUT[c]'
'AMINO-ACETONE[c]'
'GLN-tRNAs[c]'
'3Z-dodec-3-enoyl-ACPs[c]'
'OXALYL-COA[c]'
'3R-5Z-3-hydroxy-tetradec-5-enoyl-ACPs[c]'
'5Z-3-oxo-tetradec-5-enoyl-ACPs[c]'
'Beta-Lactams[c]'
'CPD-8550[c]'
'3-UREIDO-PROPIONATE[c]'
'N-ACETYL-GLUTAMYL-P[c]'
'CPD-667[c]'
'HOMO-CYS[c]'
'CPD-10254[c]'
'SUC-COA[c]'
'3-KETO-ADIPYL-COA[c]'
'CPD-258[c]'
'CPD-320[c]'
'BENZENE-NO2[c]'
'CPD-12364[c]'
'PHE[c]'
'CPD-12363[c]'
'CPD-11268[c]'
'CPD-568[c]'
'CPD-10687[c]'
'ACYL-ACP[c]'
'CPD-3740[c]'
'CPD-19493[c]'
'Omega-methylthio-alkyl-glucosinolates[c]'
'CPD-30[c]'
'omega-methylsulfinylalkylglucosinolate[c]'
'CPD-6082[c]'
'NORSPERMIDINE[c]'
'L-DELTA1-PYRROLINE\_5-CARBOXYLATE[c]'
'Protein-Phosphothreonines[c]'
'Proteins-L-Threonines[c]'
'ALLYSINE[c]'
'CAAL-proteins[c]'
'Geranylgeranylated-CAAL-proteins[c]'
'OXAMATE[c]'
'DGMP[c]'
'CPD-389[c]'
'CARBAMOYL-P[c]'
'CPD-316[c]'
'RIBOFLAVIN[c]'
'CPD-9973[c]'
'EIF5A-HYPUSINE[c]'
'Charged-GLN-tRNAs[c]'
'O-PHOSPHO-L-HOMOSERINE[c]'
'THYMIDINE[c]'
'Myristoyl-ACPs[c]'
'THYMINE[c]'
'CPD-15567[c]'
'CPD-15568[c]'
'PROPIONAMIDE[c]'
'CPD-8860[c]'
'BUTYRAMIDE[c]'
'CPD-12327[c]'
'CPD-3707[c]'
'Cytochromes-C-Oxidized[c]'
'Cytochromes-C-Reduced[c]'
'CPD-13025[c]'
'SPERMIDINE[e]'
'PUTRESCINE[e]'
'PUTRESCINE[c]'
'7-O-ACETYLSALUTARIDINOL[c]'
'CPD-7710[c]'
'CPD-7712[c]'
'CPD-7713[c]'
'CODEINONE[c]'
'MORPHINONE[c]'
'CPD-10802[c]'
'CPD-10783[c]'
'CPD-10784[c]'
'N-ACETYL-D-GLUCOSAMINE[e]'
'CPD-10803[c]'
'NADH-P-OR-NOP[e]'
'NADP[e]'
'CPD-10780[c]'
'NADPH[e]'
'CPD-10804[c]'
'AMMONIA[e]'
'NIACINAMIDE[e]'
'CPD-10785[c]'
'NIACINE[e]'
'NITRATE[e]'
'CPD-10805[c]'
'CPD-14553[c]'
'CPD0-935[c]'
'CPD0-936[c]'
'CPD0-937[c]'
'CPD0-938[c]'
'CPD-17722[c]'
'CPD-17747[c]'
'CPD-14378[c]'
'Amino-Acids[c]'
'CPD-17729[c]'
'Deoxyhypusine-Synthase-Lysine[c]'
'N-4-aminobutylidene-enzyme-lysine[c]'
'CPD-17732[c]'
'EIF5A-LYSINE[c]'
'CPD-17733[c]'
'N-4-aminobutylidene-eIF5A-lysine[c]'
'General-Protein-Substrates[c]'
'CPD-17741[c]'
'Decanoyl-ACPs[c]'
'DNA-with-3-prime-pp-5-prime-G-cap[c]'
'GMP[c]'
'3-oxo-dodecanoyl-ACPs[c]'
'DNA-Ligase-L-lysine-adenylate[c]'
'DNA-Ligase-L-lysine[c]'
'A-5-prime-PP-5-prime-DNA[c]'
'CPD-13469[c]'
'CPD-2750[c]'
'CPD-2742[c]'
'CPD-2752[c]'
'CPD-468[c]'
'NITRITE[e]'
'PANTOTHENATE[e]'
'Phosphatase-2A-leucine[c]'
'Phosphatase-2A-leucine-methyl-ester[c]'
'SARCOSINE[c]'
'CODEINE[c]'
'MORPHINE[c]'
'ACETAMIDE[c]'
'CPD-219[c]'
'CPD-15666[c]'
'2-KETO-6-AMINO-CAPROATE[c]'
'CPD-10809[c]'
'DIAMINO-OH-PHOSPHORIBOSYLAMINO-PYR[c]'
'CPD-1086[c]'
'Lignoceroyl-ACPs[c]'
'3-oxo-cerotoyl-ACPs[c]'
'CPD-17743[c]'
'CPD-17730[c]'
'S-HYDROXYMETHYLGLUTATHIONE[c]'
'CPD-17744[c]'
'CPD-17746[c]'
'CPD-17750[c]'
'CPD-548[c]'
'CPD-17757[c]'
'CPD-702[c]'
'CPD-703[c]'
'CPD-201[c]'
'CPD-202[c]'
'Deacetylated-Peptidoglycan[c]'
'NICOTINAMIDE\_NUCLEOTIDE[c]'
'CPD-9776[c]'
'DNA-Ligase-L-lysine-guanylate[c]'
'CPD-14389[c]'
'CPD-14390[c]'
'CPD-2751[c]'
'D-GLUCOSAMINE-6-P[c]'
'N-ACETYL-D-GLUCOSAMINE-6-P[c]'
'CPD-2747[c]'
'CPD-3188[c]'
'Red-NADPH-Hemoprotein-Reductases[e]'
'CPD-2749[c]'
'RIBOFLAVIN[e]'
'R-3-hydroxydodecanoyl-ACPs[c]'
'THZ-P[c]'
'AMINO-HYDROXYMETHYL-METHYLPYRIMIDINE-PP[c]'
'THIAMINE-P[c]'
'THIAMINE-PYROPHOSPHATE[c]'
'CPD-611[c]'
'THIAMINE[c]'
'PYRIDINE[c]'
'THZ[c]'
'L-PIPECOLATE[c]'
'CPD-15637[c]'
'CPD-15653[c]'
'CPD-15668[c]'
'CPD-15667[c]'
'CPD-15654[c]'
'CPD-15655[c]'
'CPD-13665[c]'
'CPD-125[c]'
'CPD-14077[c]'
'R-3-hydroxycerotoyl-ACPs[c]'
'Trans-D2-hexacos-2-enoyl-ACPs[c]'
'CPD-107[c]'
'Cerotoyl-ACPs[c]'
'NA+[e]'
'CPD-14392[c]'
'Sphingomyelins[e]'
'CPD-14018[c]'
'FERULOYL-COA[c]'
'UTP[c]'
'UDP-D-GALACTURONATE[c]'
'CPD-12231[c]'
'CPD-12261[c]'
'CPD-12279[c]'
'RNA-Containing-Guanosine[c]'
'RNA-3prime-Guanosine-3prime-P[c]'
'G-5-prime-PP-5-prime-DNA[c]'
'3-KETOACYL-COA[c]'
'RNA-Ligase-L-lysine[c]'
'L-3-HYDROXYACYL-COA[c]'
'RNA-Ligase-L-lysine-adenylate[c]'
'PORPHOBILINOGEN[c]'
'HYDROXYMETHYLBILANE[c]'
'A-5-prime-PP-5-prime-RNA[c]'
'DATP[c]'
'Oxidized-Flavoproteins[c]'
'Reduced-Flavoproteins[c]'
'CPD-3483[c]'
'CPD-3481[c]'
'CPD-1826[c]'
'HMP[c]'
'Dodec-2-enoyl-ACPs[c]'
'Thiocarboxyadenylated-ThiS-Proteins[c]'
'CPD-13575[c]'
'THIAMINE[e]'
'URACIL[e]'
'UREA[e]'
'URIDINE[e]'
'STRICTOSIDINE-AGLYCONE[c]'
'CPD-21552[c]'
'GEISSOSCHIZINE[c]'
'POLYNEURIDINE-ALDEHYDE[c]'
'HIF-alpha-subunit-L-asparagines[c]'
'HIF-alpha-subunit-3S-OH-ASN[c]'
'CPD-12288[c]'
'Glucosyl-acyl-sphinganines[c]'
'Glucosyl-acyl-sphingosines[c]'
'R-6-HYDROXYNICOTINE[c]'
'CPD-14407[c]'
'CPD-17794[c]'
'CPD-14422[c]'
'CPD-14423[c]'
'CPD-14424[c]'
'Protein-Ser-or-Thr-phosphate[c]'
'Protein-L-serine-or-L-threonine[c]'
'CPD-14425[c]'
'CPD-14426[c]'
'CPD-13328[c]'
'CPD-15656[c]'
'CPD-15657[c]'
'PYRIDOXAL[e]'
'CPD-15675[c]'
'CPD-15651[c]'
'CPD-15652[c]'
'CPD-15677[c]'
'CPD-15676[c]'
'AMINO-HYDROXYMETHYL-METHYL-PYR-P[c]'
'DNA-3-methyladenines[c]'
'3-Methyl-Adenines[c]'
'QUINOLINATE[e]'
'RNA-with-3-prime-pp-5-prime-A-cap[c]'
'RNA-3-prime-P-cyclase-L-histidine[c]'
'RNA-3-prime-P-cyclase-L-His-adenylate[c]'
'N-Acylsphingosine[c]'
'XANTHINE[e]'
'Amino-Acids-20[e]'
'2-HYDROXY-2-METHYLPROPANENITRILE[c]'
'BETAINE\_ALDEHYDE[c]'
'CPD-5164[c]'
'Thi-S[c]'
'CPD-17883[c]'
'CPD-5165[c]'
'CPD-17884[c]'
'CPD-5166[c]'
'D-ALA-D-ALA[c]'
'CPD-17885[c]'
'AMMONIA[c]'
'CPD-5167[c]'
'S-ADENOSYL-4-METHYLTHIO-2-OXOBUTANOATE[c]'
'5-AMINOPENTANOATE[c]'
'CPD-17887[c]'
'CPD-12303[c]'
'UDP-N-ACETYL-D-GLUCOSAMINE[c]'
'CPD-12304[c]'
'CPD-394[c]'
'CPD-17802[c]'
'CPD-7993[c]'
'CPD-12258[c]'
'CPD-12311[c]'
'CPD-7994[c]'
'CPD-12310[c]'
'CPD-17888[c]'
'Z-11-TETRADECENOYL-COA[c]'
'NICOTINE[c]'
'CPD-2748[c]'
'CPD-5168[c]'
'Uridine44-in-tRNA-Ser[c]'
'2-O-Methyluridine44-tRNASer[c]'
'Guanine10-in-tRNA[c]'
'tRNA-Containing-N2-Methylguanine-10[c]'
'Guanine26-in-tRNA[c]'
'tRNA-Containing-N2-Methylguanine-26[c]'
'tRNA-Containing-N2-dimethylguanine-26[c]'
'GDP[c]'
'L-CANALINE[c]'
'UREA[c]'
'CANAVANINE[c]'
'CPD-15661[c]'
'CPD-10832[c]'
'CPD-15662[c]'
'CPD-15678[c]'
'CPD-15663[c]'
'CPD-9407[c]'
'CPD-15658[c]'
'CPD0-1308[c]'
'CPD0-1074[c]'
'Short-Chain-Trans-23-Dehydroacyl-CoA[c]'
'Short-Chain-234-Saturated-acyl-CoAs[c]'
'Very-Long-Chain-Trans-23-Dehydroacyl-CoA[c]'
'CPD-19268[c]'
'Very-long-Chain-234-Saturated-acyl-CoAs[c]'
'12-DEHYDROTETRACYCLINE[c]'
'CPD-19274[c]'
'CPD-19272[c]'
'VAL-tRNAs[c]'
'Charged-VAL-tRNAs[c]'
'BETAINE[c]'
'DICARBOXYLIC-ACID-MONOAMIDES[c]'
'L-CITRULLINE[c]'
'CPD-19474[c]'
'Pyruvate-Dehydrogenase-Phosphoserine[c]'
'Pyruvate-dehydrogenase-L-serine[c]'
'CPD-22266[c]'
'CPD-22267[c]'
'NITRATE[c]'
'TRYPANOTHIONE-DISULFIDE[c]'
'TRYPANOTHIONE[c]'
'Dihydro-Lipoyl-Proteins[c]'
'Lipoyl-Protein-N6-lipoyllysine[c]'
'D-GLT[c]'
'D-Amino-Acids[c]'
'N-ACETYL-D-AMINO-ACID[c]'
'3-Hydroxy-octanoyl-ACPs[c]'
'3-Oxo-octanoyl-ACPs[c]'
'PHYTOSPINGOSINE[c]'
'tRNAPhe-Containing-4-demethylwyosine-37[c]'
'yW-86[c]'
'Octadec-2-enoyl-ACPs[c]'
'GLUCOSAMINE[e]'
'yW-58[c]'
'tRNAPhe-wybutosine[c]'
'yW-72[c]'
'OHyWstar-tRNA[c]'
'OHyW-58-tRNAPhe[c]'
'HYDANTOIN[c]'
'N-CARBAMOYLGLYCINE[c]'
'SULFO-CYSTEINE[c]'
'GDP-4-DEHYDRO-6-DEOXY-D-MANNOSE[c]'
'CPD-11281[c]'
'421-DEHYDROGEISSOSCHIZINE[c]'
'N-5S-5-AMINO-5-CARBOXYPENTANOYL-L-CY[c]'
'Apo-Propionyl-CoA-CO2-ligases[c]'
'Propionyl-CoA-CO2-ligases[c]'
'3-methylcrotonoyl-CoA-carboxylase-lysine[c]'
'Biotin-EC6-4-1-4[c]'
'CPD-14280[c]'
'CPD-10279[c]'
'CPD-14281[c]'
'CPD-10280[c]'
'CPD-14282[c]'
'NMNH[c]'
'CPD0-881[c]'
'CPD1G-277[c]'
'CPD-14283[c]'
'CPD-14300[c]'
'CANAVANINOSUCCINATE[c]'
'HOMOMETHIONINE[c]'
'N-Ac-L-methionyl-L-tyrosinyl-Protein[c]'
'N-Ac-N-terminal-L-valine[c]'
'N-terminal-L-valine[c]'
'N-terminal-L-alanine[c]'
'N-terminal-N-Ac-L-alanine[c]'
'N-terminal-L-cysteine[c]'
'N-terminal-N-Ac-L-cysteine[c]'
'CPD-9406[c]'
'CPD-17635[c]'
'N-terminal-glycine[c]'
'N-terminal-N-Ac-glycine[c]'
'N-terminal-N-Ac-L-Serine[c]'
'N-terminal-L-Serine[c]'
'S-PRENYL-L-CYSTEINE[c]'
'Protein-L-methionine-R-S-oxides[c]'
'Protein-L-methionine[c]'
'CPD-8989[c]'
'CPD-320[e]'
'FERROCYTOCHROME-B5[e]'
'FMN[e]'
'Folates[e]'
'OHyW-tRNAPhe[c]'
'ARG[c]'
'CPD-15413[c]'
'25S-rRNA-adenine-2142[c]'
'25S-rRNA-N1-methyladenine-2142[c]'
'25S-rRNA-adenine-645[c]'
'25S-rRNA-N1-methyladenine-645[c]'
'S-palmitoyl-L-cysteine-in-proteins[c]'
'PROT-CYS[c]'
'2-Octenoyl-ACPs[c]'
'apo-Transcarboxylases[c]'
'CPD-569[c]'
'L-aspartyl-tRNAAsn[c]'
'Octanoyl-ACPs[c]'
'Charged-ASN-tRNAs[c]'
'L-glutamyl-tRNAGln[c]'
'CPD-220[c]'
'5-HYDROXY-FERULOYL-COA[c]'
'CPD-12180[c]'
'O-Long-Chain-Acyl-L-Carnitines[c]'
'SINAPOYL-COA[c]'
'18S-rRNA-pseudouridine-1191[c]'
'18S-rRNA-N1-methylpseudouridine-1191[c]'
'VLC-Alpha-hydroxyphytoceramides[c]'
'IPC[c]'
'VLC-MIPC[c]'
'DI-H-OROTATE[c]'
'3-METHYLTHIOPROPANALDOXIME[c]'
'CPD-7546[c]'
'3-METHYLTHIOPROPYLHYDROXAMIC-ACID[c]'
'3-METHYLTHIOPROPYL-DESULFO-GLUCOSINOLATE[c]'
'3-METHYLTHIOPROPYL-GLUCOSINOLATE[c]'
'3-METHYLSULFINYLPROPYL-GLUCOSINOLATE[c]'
'2-PROPENYL-GLUCOSINOLATE[c]'
'CPD-8990[c]'
'4-AMINO-BUTYRATE[e]'
'GLUTATHIONE[e]'
'GLY[e]'
'GUANINE[e]'
'APS[c]'
'CAMP[c]'
'O-UREIDOHOMOSERINE[c]'
'CPD-7652[c]'
'DIHYDROPTERIN-CH2OH-PP[c]'
'CPD-10766[c]'
'Protein-N-terminal-N-Ac-L-threonine[c]'
'Protein-N-terminal-L-threonine[c]'
'N-terminal-L-Serine-Histone-H2A[c]'
'N-terminal-N-Ac-L-Serine-Histone-2A[c]'
'N-terminal-L-Serine-Histone-H4[c]'
'N-terminal-N-Ac-L-Serine-Histone-4[c]'
'L-methionyl-L-lysyl-Protein[c]'
'N-Ac-L-methionyl-L-lysyl-Protein[c]'
'L-methionyl-L-valyl-Protein[c]'
'N-Ac-L-methionyl-L-valyl-Protein[c]'
'L-methionyl-L-alanyl-Protein[c]'
'N-Ac-L-methionyl-L-alanyl-Protein[c]'
'CPD-656[c]'
'CPD-17370[c]'
'CPD-15435[c]'
'N6-L-threonylcarbamoyladenine37-tRNAs[c]'
'tRNA-adenine-37[c]'
'CPD-15436[c]'
'Butanoyl-ACPs[c]'
'3-oxo-decanoyl-ACPs[c]'
'CPD-10794[c]'
'3-HYDROXYADIPYL-COA[c]'
'TRANS-23-DEHYDROADIPYL-COA[c]'
'5-METHYLTHIOINOSINE[c]'
'MET[e]'
'CPD-452[c]'
'CPD-1113[c]'
'CPD-160[c]'
'CPD-12199[c]'
'HYPOXANTHINE[e]'
'CPD-468[e]'
'L-ALPHA-ALANINE[e]'
'ARG[e]'
'L-methionyl-L-seryl-Protein[c]'
'N-Ac-L-methionyl-L-seryl-Protein[c]'
'L-methionyl-L-threonyl-Protein[c]'
'N-Ac-L-methionyl-L-threonyl-Protein[c]'
'Aminopeptidase-Substrates[c]'
'L-methionyl-L-cysteinyl-Protein[c]'
'L-methionyl-glycyl-Protein[c]'
'IMINOASPARTATE[c]'
'CPD-17641[c]'
'PHOSPHORIBOSYL-ATP[c]'
'CPD-17638[c]'
'ASN[e]'
'CPD-7545[c]'
'L-ASPARTATE[e]'
'CPD-15414[c]'
'L-CITRULLINE[e]'
'CYS[e]'
'GLT[e]'
'GLN[e]'
'HIS[e]'
'INDOLE\_PYRUVATE[c]'
'TRP[c]'
'HOMO-CYS[e]'
'CPD-15554[c]'
'CPD-10781[c]'
'O-Acyl-L-Carnitines[e]'
'O-Acyl-L-Carnitines[c]'
'Beta-hydroxydecanoyl-ACPs[c]'
'CPD-10600[c]'
'CPD-13181[c]'
'CPD-13182[c]'
'HOMO-SER[c]'
'16-EPIVELLOSIMINE[c]'
'CPD0-1470[c]'
'CPD-19217[c]'
'S-NITROSOGLUTATHIONE[c]'
'Charged-ARG-tRNAs[c]'
'L-Glutamyl-Peptides[c]'
'Lipoyl-Protein-L-Lysine[c]'
'Octanoylated-domains[c]'
'N-ETHYLMALEIMIDE[c]'
'CPD0-903[c]'
'ILE[e]'
'LEU[e]'
'LYS[e]'
'L-ORNITHINE[e]'
'CPD-17624[c]'
'PHE[e]'
'PRO[e]'
'SER[e]'
'Phosphoacetylglucosamine-Mutase[c]'
'Phosphoacetylglucosamine-Mutase-P[c]'
'N-ACETYL-D-GLUCOSAMINE-16-BIS-P[c]'
'THR[e]'
'CPD-17714[c]'
'CPD-17701[c]'
'CPD-308[c]'
'CPD-15566[c]'
'CPD-10782[c]'
'THIOHYDROXIMATE-O-SULFATES[c]'
'Nitriles[c]'
'CPD-558[c]'
'Alkenyl-Thiohydroximate-O-Sulfates[c]'
'EPITHIONITRILES[c]'
'CPD-10800[c]'
'CDPDIACYLGLYCEROL[c]'
'CPD-10801[c]'
'S-RETICULINE[c]'
'12-DEHYDRORETICULINIUM[c]'
'TRP[e]'
'Acyl-homoserine-lactones[c]'
'Acyl-homoserines[c]'
'TYR[e]'
'VAL[e]'
'CPD-12364[e]'
'L-arginyl-L-Glutamyl-Peptides[c]'
'ARG-tRNAs[c]'
'Proteins-With-N-Terminal-Asp[c]'
'L-arginyl-L-aspartyl-Peptides[c]'
'TTP[c]'
'L-arginyl-3-sulfino-L-alaninyl-Peptides[c]'
'N-terminal-L-cysteine-sulfinate[c]'
'N-terminal-L-cysteine-sulfonate[c]'
'SER[c]'
'L-arginyl-3-sulfo-L-alaninyl-Peptides[c]'
'N-Ac-L-methionyl-L-asparaginyl-Protein[c]'
'N-terminal-asparagine[c]'
'CPD0-2015[c]'
'L-Glutaminyl-Peptides[c]'
'N-Ac-L-methionyl-L-glutaminyl-Protein[c]'
'Trans-D2-decenoyl-ACPs[c]'
'UROPORPHYRINOGEN-III[c]'
'DIMETHYL-GLYCINE[c]'
'CPD-13473[c]'
'CPD-17723[c]'
'CPD-17724[c]'
'CPD-17725[c]'
'O-SUCCINYL-L-HOMOSERINE[c]'
'L-CYSTATHIONINE[c]'
'CPD-3187[c]'
'CPD-14092[c]'
'CPD-6972[c]'
'CPD-22025[c]'
'CPD-22027[c]'
'CPD-22028[c]'
'CPD-22029[c]'
'VLC-Ceramides[c]'
'CPD-10556[c]'
'Ultra-Long-Chain-Acyl-CoAs[c]'
'ULC-Cermaides[c]'
'CPD-13612[c]'
'CPD-22033[c]'
'SACCHAROPINE[c]'
'ISOVALERYL-COA[c]'
'BCAA-dehydrogenase-3MB-DH-lipoyl[c]'
'CPD-18831[c]'
'CPD-18832[c]'
'CPD-9038[c]'
'Cytochromes-C-Reduced[e]'
'CPD-15265[c]'
'Cytochromes-C-Oxidized[e]'
'CPD-15263[c]'
'CPD-1862[c]'
'CPD-15268[c]'
'CPD-15260[c]'
'CPD-602[c]'
'CPD-15259[c]'
'CPD-15261[c]'
'Ergothioneine[c]'
'CPD-19154[c]'
'CPD-19161[c]'
'CPD-11571[c]'
'CPD-19157[c]'
'CPD-12101[c]'
'CPD-19148[c]'
'3-oxo-hexanoyl-ACPs[c]'
'R-3-hydroxyhexanoyl-ACPs[c]'
'N-SUCCINYL-2-AMINO-6-KETOPIMELATE[c]'
'N2-SUCCINYLORNITHINE[c]'
'CPD-822[c]'
'L-methionyl-tRNAfmet[c]'
'NN-dimethyl-terminal-XPK[c]'
'NNN-trimethyl-terminal-XPK[c]'
'CPD-17434[c]'
'CYTIDINE[c]'
'N-terminal-XPK[c]'
'CYTOSINE[c]'
'N-terminal-PPK[c]'
'NN-dimethyl-terminal-PPK[c]'
'CPD-17428[c]'
'N-methyl-terminal-PPK[c]'
'CPD-17052[c]'
'CPD-17453[c]'
'CPD66-40[c]'
'CPD-22034[c]'
'CPD-22035[c]'
'CPD-9965[c]'
'CPD-22036[c]'
'CPD-16352[c]'
'URIDINE[c]'
'HISTIDINOL[c]'
'HIS[c]'
'COPROPORPHYRINOGEN\_III[c]'
'Oxidized-NrdH-Proteins[c]'
'Reduced-NrdH-Proteins[c]'
'CPD-15285[c]'
'CPD0-2298[c]'
'CPD-1091[c]'
'CPD-1863[c]'
'CPD-1881[c]'
'L-4-HYDROXYGLUTAMATE\_SEMIALDEHYDE[c]'
'UDP-D-GALACTO-14-FURANOSE[c]'
'L-Amino-Acids[c]'
'5-OXOPROLINE[c]'
'5-L-GLUTAMYL-PEPTIDE[c]'
'5-L-GLUTAMYL-AMINO-ACID[c]'
'Hex-2-enoyl-ACPs[c]'
'CPD-17455[c]'
'CPD-19150[c]'
'CPD-17053[c]'
'CPD-19151[c]'
'CPD-19153[c]'
'Kanamycin-3-phosphates[c]'
'Kanamycins[c]'
'CPD-16353[c]'
'CPD-22039[c]'
'CPD-14293[c]'
'CPD-22040[c]'
'D-3-HYDROXYACYL-COA[c]'
'CPD-22041[c]'
'CPD-14269[c]'
'CPD-22043[c]'
'CPD-15363[c]'
'3-oxo-stearoyl-ACPs[c]'
'CPD-307[c]'
'THIOMORPHOLINE-3-CARBOXYLATE[c]'
'34-DEHYDRO-14-THIOMORPHOLINE-3-CARBOXY[c]'
'R-RETICULINE[c]'
'CPD-2022[c]'
'L-ERYTHRO-4-HYDROXY-GLUTAMATE[c]'
'CPD-15361[c]'
'P-AMINO-BENZOATE[e]'
'CPD-1302[e]'
'Red-Thioredoxin[e]'
'ACP[e]'
'CPDQT-273[c]'
'CPD-12140[c]'
'CPD1G-2[c]'
'CPD-12152[c]'
'CPD-12156[c]'
'NICOTINAMIDE\_RIBOSE[c]'
'Pyrimidine-Bases[c]'
'2-Acylglycero-Phosphocholines[c]'
'HYPOTAURINE[c]'
'BCAA-dehydrogenase-lipoyl[c]'
'5-PHOSPHO-RIBOSYL-GLYCINEAMIDE[c]'
'5-P-RIBOSYL-N-FORMYLGLYCINEAMIDE[c]'
'CPD-14925[c]'
'Hexanoyl-ACPs[c]'
'CPD-15364[c]'
'CPD-22044[c]'
'CPD-22045[c]'
'CPD-22048[c]'
'CPD-14271[c]'
'CPD-22050[c]'
'CPD-10283[c]'
'CPD-18491[c]'
'UDP-SULFOQUINOVOSE[c]'
'apo-Peptidyl-carrier-proteins[c]'
'L-2-AMINOPENTANOIC-ACID[c]'
'ADENINE[e]'
'ADENOSINE[e]'
'AMMONIUM[e]'
'ETF-Oxidized[e]'
'ANTHRANILATE[e]'
'CPD-7400[c]'
'CYANURIC-ACID[c]'
'GLUTAMYL-GLX-TRNAS[c]'
'GLX-tRNAs[c]'
'Phytosphingosines[c]'
'Very-Long-Chain-Phytoceramides[c]'
'CPD-15362[c]'
'CPD-15368[c]'
'CPD-3736[c]'
'Ferrihemoglobins[c]'
'Ferrohemoglobins[c]'
'CPD-19144[c]'
'CPD-19170[c]'
'AMINOMETHYLDIHYDROLIPOYL-GCVH[c]'
'DIHYDROLIPOYL-GCVH[c]'
'CPD-17496[c]'
'P-NITROPHENOL[c]'
'CPD-9000[c]'
'CPD-14274[c]'
'CPD-14273[c]'
'CPD-13381[c]'
'CPD-14275[c]'
'CPD-14736[c]'
'CPD-19168[c]'
'ANTHRANILATE[c]'
'MAP-Kinase-L-Tyr[c]'
'MAP-Kinase-L-Phosphotyrosine[c]'
'CPD-14276[c]'
'3-HYDROXY-L-KYNURENINE[c]'
'CPD-14277[c]'
'CPD-321[c]'
'CPD-14133[c]'
'CPD-22265[c]'
'CPD-19186[c]'
'Protein-S-methyl-L-cysteine[c]'
'a-thymine-in-DNA[c]'
'CPD0-2500[c]'
'Protein-Red-Disulfides[c]'
'L-methionyl-L-asparaginyl-Protein[c]'
'Protein-Ox-Disulfides[c]'
'L-methionyl-L-glutaminyl-Protein[c]'
'N-Ac-L-methionyl-L-aspartyl-Protein[c]'
'Alkyl-acetyl-glycero-phosphocholines[c]'
'1-Alkyl-sn-glycero-3-phosphocholines[c]'
'CPD-19167[c]'
'3-OXOPIMELOYL-COA[c]'
'Phytoceramides[c]'
'Alpha-hydroxyphytoceramides[c]'
'CPD-15369[c]'
'CPD-15370[c]'
'CPD-15366[c]'
'Guanine37-in-tRNAPhe[c]'
'tRNAPhe-Containing-N1-Methylguanine-37[c]'
'3-UREIDO-ISOBUTYRATE[c]'
'CPD-471[c]'
'CPD-209[c]'
'C3[c]'
'DIHYDRO-THYMINE[c]'
'CHITIN[e]'
'CPD-13545[e]'
'Chitosan[e]'
'CHOLINE[e]'
'CPD-69[e]'
'CPD-14278[c]'
'L-methionyl-L-aspartyl-Protein[c]'
'N-Ac-L-methionyl-L-glutamyl-Protein[c]'
'CPD3DJ-82[c]'
'L-methionyl-L-glutamyl-Protein[c]'
'L-methionyl-L-leucyl-Protein[c]'
'N-Ac-L-methionyl-L-leucyl-Protein[c]'
'Sphinga-4E-8E-dienine-Ceramides[c]'
'9-Methyl-sphing-4-8-dienine-ceramides[c]'
'L-methionyl-L-isoleucyl-Protein[c]'
'N-Ac-L-methionyl-L-isoleucyl-Protein[c]'
'L-methionyl-L-phenylalanyl-Protein[c]'
'ADP-D-GLYCERO-D-MANNO-HEPTOSE[c]'
'N-Ac-L-methionyl-L-phenylalanyl-Protein[c]'
'L-methionyl-L-tryptophanyl-Protein[c]'
'N-Ac-L-methionyl-L-tryptophanyl-Protein[c]'
'L-methionyl-L-tyrosinyl-Protein[c]'
'CPD0-2474[c]'
'NITRIC-OXIDE[c]'
'Aliphatic-Nitriles[c]'
'Primary-Aliphatic-Amides[c]'
'CPD-19172[c]'
'N5-Formyl-THF-Glu-N[c]'
'CPD-19171[c]'
'2-AMINOMUCONATE\_SEMIALDEHYDE[c]'
'2-AMINO-MUCONATE[c]'
'CPD-19169[c]'
'CPD-1061[c]'
'ASP-tRNAs[c]'
'Charged-ASP-tRNAs[c]'
'CPD-4205[c]'
'CPD-302[c]'
'CPD-17312[c]'
'TRANS-D2-ENOYL-COA[c]'
'Trans-3-enoyl-CoAs[c]'
'GAMMA-LINOLENOYL-COA[c]'
'CPD-21828[c]'
'ARACHIDONYL-COA[c]'
'TAURINE[c]'
'Charged-CYS-tRNAs[c]'
'CPD-15192[c]'
'CPD-15216[c]'
'CPD-629[c]'
'CPD-17387[c]'
'CPD-17386[c]'
'CPD-17388[c]'
'INDOLE[c]'
'Behenoyl-ACPs[c]'
'3-oxo-lignoceroyl-ACPs[c]'
'R-3-hydroxylignoceroyl-ACPs[c]'
'trans-delta2-lignoceroyl-ACPs[c]'
'NICOTINATE\_NUCLEOTIDE[c]'
'CPD-581[c]'
'DIMETHYLAMINE[c]'
'CPD-402[c]'
'N-METHYLANTHRANILOYL-COA[c]'
'CPD-22005[c]'
'CPD-22004[c]'
'CPD-22006[c]'
'CPD-22003[c]'
'CPD-22008[c]'
'CPD-22009[c]'
'IMP[c]'
'L-ASPARTATE-SEMIALDEHYDE[c]'
'L-BETA-ASPARTYL-P[c]'
'CPD-5881[c]'
'CPD-14202[c]'
'TETRADEHYDROACYL-COA[c]'
'2-hydroxyacyl-glutathiones[c]'
'CPD0-934[c]'
'CPD-15237[c]'
'N-ACETYL-5-METHOXY-TRYPTAMINE[c]'
'5-HYDROXYISOURATE[c]'
'CPD-12014[c]'
'N-ACETYL-SEROTONIN[c]'
'STRICTOSIDINE[c]'
'TRYPTAMINE[c]'
'CPD-8900[c]'
'Protein-L-lysine[c]'
'CPD-17381[c]'
'CPD-17392[c]'
'DNA-with-Uracils[c]'
'PHOSPHORYL-ETHANOLAMINE[c]'
'CPD3DJ-11366[c]'
'SPHINGOSINE[c]'
'CPD-22010[c]'
'CPD-22007[c]'
'3S-CITRYL-COA[c]'
'CPD-22012[c]'
'CPD-22013[c]'
'CPD-7221[c]'
'CPD-7222[c]'
'CPD-22014[c]'
'CPD-10189[c]'
'CPD-10188[c]'
'B-KETOACYL-ACP[c]'
'OH-ACYL-ACP[c]'
'CPD-19163[c]'
'CARBAMYUL-L-ASPARTATE[c]'
'CPD-19159[c]'
'CPD-216[c]'
'Protein-L-serines[c]'
'Protein-D-serines[c]'
'CYSTINE[c]'
'THIOCYSTEINE[c]'
'CPD-15240[c]'
'Sulfur-Carrier-Proteins-ThiI[c]'
'Sulfurylated-ThiI[c]'
'CPD-578[c]'
'N-SUCCINYLLL-2-6-DIAMINOPIMELATE[c]'
'LL-DIAMINOPIMELATE[c]'
'CPD-12015[c]'
'CPD-12017[c]'
'CPD-17395[c]'
'BCAA-dehydrogenase-2MP-DH-lipoyl[c]'
'CPD-17390[c]'
'CPD-17389[c]'
'CPD-17391[c]'
'CPD-17393[c]'
'CPD-17394[c]'
'ISOPENICILLIN-N[c]'
'5-HYDROXY-TRYPTOPHAN[c]'
'SEROTONIN[c]'
'CPD-7224[c]'
'CPD-22011[c]'
'CPD-22016[c]'
'CPD-22017[c]'
'CPD-22018[c]'
'CPD-22015[c]'
'CPD-22021[c]'
'PENICILLIN-N[c]'
'D-SERINE[c]'
'S-3-HYDROXYBUTANOYL-COA[c]'
'CPD-650[c]'
'ADENOSYL-P4[c]'
'ADP-L-GLYCERO-D-MANNO-HEPTOSE[c]'
'DEOXYURIDINE[c]'
'CPD-19160[c]'
'Acetoacetyl-ACPs[c]'
'CPD-10269[c]'
'CPD-19162[c]'
'Beta-3-hydroxybutyryl-ACPs[c]'
'CPD-15244[c]'
'CPD-12016[c]'
'CPD-12259[c]'
'CPD-12230[c]'
'CPD-12018[c]'
'CPD-12019[c]'
'CPD-425[c]'
'CPD-18346[c]'
'CPD-1772[c]'
'CPD-15365[c]'
'CPD-17401[c]'
'UDP-D-XYLOSE[c]'
'DI-H-URACIL[c]'
'CPD-22022[c]'
'CPD-22023[c]'
'CPD-22020[c]'
'ILE-tRNAs[c]'
'Charged-ILE-tRNAs[c]'
'CPD-22026[c]'
'S-2-METHYLACYL-COA[c]'
'R-2-METHYLACYL-COA[c]'
'Cis-delta-3-decenoyl-ACPs[c]'
'Crotonyl-ACPs[c]'
'CPD-15254[c]'
'CPD-4822[c]'
'Saturated-Fatty-Acyl-ACPs[c]'
'CPD-15266[c]'
'CPD-15267[c]'
'CPD-19158[c]'
'CPD-19155[c]'
'CPD-19147[c]'
'N-METHYLTRYPTOPHAN[c]'
'CPD-12991[c]'
'CPD-17402[c]'
'CPD-17403[c]'
'CPD-15367[c]'
'Initiation-tRNAmet[c]'
'N-ACETYL-D-MANNOSAMINE-6P[c]'
'3-oxo-arachidoyl-ACPs[c]'
'Arachidoyl-ACPs[c]'
'trans-delta2-arachidoyl-ACPs[c]'
'Gcv-H[c]'
'cis-cis-19-31-dicyclopropyl-C52-ACPs[c]'
'LIPOYL-AMP[c]'
'CPD1G-124[c]'
'PARATHION[c]'
'4-AMINO-BUTYRALDEHYDE[c]'
'CPD-17338[c]'
'CDP-ETHANOLAMINE[c]'
'1-Alkyl-2-acyl-glycerol-P-Etn[c]'
'CPD-514[c]'
'CPD0-2171[c]'
'CPD0-2105[c]'
'4-Phosphooxy-L-aspartyl-tRNAAsn[c]'
'CPD-14916[c]'
'CPD0-2106[c]'
'5-Phosphooxy-L-glutamyl-tRNAGlln[c]'
'CPD0-2108[c]'
'CPD-17866[c]'
'5-PHOSPHORIBOSYL-N-FORMYLGLYCINEAMIDINE[c]'
'Reduced-flavodoxins[c]'
'Oxidized-flavodoxins[c]'
'CREATININE[c]'
'DITP[c]'
'N-METHYLHYDANTOIN[c]'
'DIMP[c]'
'XTP[c]'
'5-BETA-L-THREO-PENTAPYRANOSYL-4-ULOSE-[c]'
'NIACINE[c]'
'CPD-14074[c]'
'CPD-14075[c]'
'CPD-14076[c]'
'CPD-867[c]'
'CPD-868[c]'
'TRANS-D2-ENOYL-ACP[c]'
'23-Diaminopropanoate[c]'
'THREO-3-HYDROXY-L-ASPARTATE[c]'
'CPD-17346[c]'
'CDP-CHOLINE[c]'
'Plasmanylcholine[c]'
'CPD-17347[c]'
'Alkyl-enyl-acyl-gly-P-EtOH-amines[c]'
'CPD-17348[c]'
'CPD1G-120[c]'
'CPD-12646[c]'
'1-Alkenylglycerophosphoethanolamines[c]'
'cis-19-CP-37-Mex-38-Me-C59-ACPs[c]'
'CPD1G-204[c]'
'N-FORMYLKYNURENINE[c]'
'CPD-657[c]'
'CPD0-2121[c]'
'FORMAMIDE[c]'
'UDP-4-AMINO-4-DEOXY-L-ARABINOSE[c]'
'SPERMINE[c]'
'CPD-10088[c]'
'CPD-10091[c]'
'CPD1G-332[c]'
'S-Substituted-Glutathione[c]'
'CPD-10157[e]'
'R-3-hydroxy-cis-vaccenoyl-ACPs[c]'
'S-Substituted-Glutathione[e]'
'CPD-124[c]'
'METHYLAMINE[c]'
'ACRYLYL-COA[c]'
'CPD-19953[c]'
'CPD-60[c]'
'N-FORMIMINO-GLYCINE[c]'
'CPD-20[c]'
'CPD-19953[e]'
'CPD-14795[c]'
'GLUCOSAMINATE[c]'
'DEACETYLISOIPECOSIDE[c]'
'CPD-68[c]'
'10-FORMYL-DIHYDROFOLATE-GLU-N[c]'
'DEACETOXYCEPHALOSPORIN-C[c]'
'DEAMIDO-NAD[c]'
'CPD-17324[c]'
'CPD-17367[c]'
'CPD-17368[c]'
'CPD-17323[c]'
'CPD-71[c]'
'CPD-7275[c]'
'NIACINAMIDE[c]'
'PLASMENYLCHOLINE[c]'
'CPD-563[c]'
'ASN[c]'
'Protein-S-Acyl-Cysteines[c]'
'3-Oxoacyl-CoAs[c]'
'Protein-S-Acetyl-Cysteines[c]'
'CPD-649[c]'
'CPD-659[c]'
'CPD-10157[c]'
'VibB[c]'
'holo-VibB[c]'
'CARBOXYPHENYLAMINO-DEOXYRIBULOSE-P[c]'
'INDOLE-3-GLYCEROL-P[c]'
'4-IMIDAZOLONE-5-PROPIONATE[c]'
'N-FORMIMINO-L-GLUTAMATE[c]'
'b-Keto-cis-D5-dodecenoyl-ACPs[c]'
'CPD-17371[c]'
'2-KETO-GLUTARAMATE[c]'
'CPD-14096[c]'
'2-5-DIHYDROXYPYRIDINE[c]'
'CPD-17378[c]'
'CPD-14100[c]'
'CPD-17379[c]'
'UDP-OHMYR-ACETYLGLUCOSAMINE[c]'
'CPD-12173[c]'
'CPD-396[c]'
'ETHANOL-AMINE[c]'
'ISOBUTYRYL-COA[c]'
'CPD-21754[c]'
'CPD-158[c]'
'IMIDAZOLE-ACETOL-P[c]'
'CPD-11975[c]'
'CPD1G-1[c]'
'Sphingomyelins[c]'
'CPD-17380[c]'
'b-Hydroxy-cis-D5-dodecenoyl-ACPs[c]'
'Trans-D3-cis-D5-dodecenoyl-ACPs[c]'
'CPD-17382[c]'
'CPD-17332[c]'
'CPD-17383[c]'
'tRNA-Sec[c]'
'cis-cis-D19-31-C50-2-ACPs[c]'
'UDP-N-ACETYLMURAMATE[c]'
'UDP-ACETYL-CARBOXYVINYL-GLUCOSAMINE[c]'
'CPD-14122[c]'
'3-oxo-behenoyl-ACPs[c]'
'CPD-14123[c]'
'DUDP[c]'
'CPD-13811[c]'
'CPD-6365[c]'
'R-3-hydroxybehenoyl-ACPs[c]'
'ASN-tRNAs[c]'
'5-AMINO-LEVULINATE[c]'
'CPD-69[c]'
'PHOSPHORIBOSYL-FORMAMIDO-CARBOXAMIDE[c]'
'CPD-248[c]'
'2-trans-4-cis-dienoyl-CoAs[c]'
'INDOLE\_ACETATE\_AUXIN[c]'
'PENTANOYLCOA-CPD[c]'
'CPD-13851[c]'
'CPD-15190[c]'
'P-AMINO-BENZOATE[c]'
'CPD-15191[c]'
'trans-delta2-behenoyl-ACPs[c]'
'CPD-11997[c]'
'CPD-11998[c]'
'L-seryl-SEC-tRNAs[c]'
'CPD-17331[c]'
'CPD-17385[c]'
'2-AMINOMALONATE-SEMIALDEHYDE[c]'
'3-METHYL-CROTONYL-COA[c]'
'CPD-19029[c]'
'CPD-19031[c]'
'CPD-19032[c]'
'CPD-14394[c]'
'CPD-8259[c]'
'CPD1G-0[c]'
'PROTOPORPHYRIN\_IX[c]'
'PROTOPORPHYRINOGEN[c]'
'CPD0-2231[c]'
'Very-Long-Chain-oxoacyl-CoAs[c]'
'Very-Long-Chain-3-Hydroxyacyl-CoAs[c]'
'S-NORLAUDANOSOLINE[c]'
'3-HYDROXY-3-4-METHYLPENT-3-EN-1-YLG-COA[c]'
'CPD-12897[c]'
'RNA-3-Guanosine-23-Cyclophosphate[c]'
'N-CYCLOHEXYLFORMAMIDE[c]'
'Peptide-with-C-terminal-Lysine[c]'
'CYCLOHEXYL-ISOCYANIDE[c]'
'CPD-7243[c]'
'SER-tRNAs[c]'
'Charged-SER-tRNAs[c]'
'Protein-N-terminal-L-Arginine[c]'
'L-ARGININO-SUCCINATE[c]'
'CPD-14405[c]'
'CPD-14406[c]'
'CPD-17378[e]'
'CPD-17379[e]'
'CPD-17380[e]'
'CPD-17381[e]'
'CPD-17389[e]'
'CPD-113[c]'
'CPD-123[c]'
'3-HYDROXY-ANTHRANILATE[c]'
'2-AMINO-3-3-OXOPROP-2-ENYL-BUT-2-ENEDI[c]'
'CPD-143[c]'
'CPD-145[c]'
'CPD-19042[c]'
'5-P-BETA-D-RIBOSYL-AMINE[c]'
'C18-Phytoceramides[c]'
'CPD-17210[c]'
'CPD-17313[c]'
'Reduced-Cys2-Peroxiredoxins[c]'
'2Cys-Peroxiredoxins-With-HydroxyCys[c]'
'Cys2-Peroxiredoxin-Disulfide[c]'
'Medium-Chain-Acyl-CoAs[c]'
'Medium-Chain-234-Saturated-acyl-CoAs[c]'
'dihomogammalinolenoyl-acp[c]'
'DEHYDROSPHINGANINE[c]'
'CPD-14419[c]'
'CPD-14420[c]'
'Unfolded-Proteins[c]'
'CPD-12647[c]'
'CPD-17392[e]'
'CPD-17393[e]'
'CPD-17394[e]'
'CPD-17395[e]'
'CPD-17390[e]'
'CPD-17391[e]'
'CPD-206[c]'
'CPD-144[c]'
'2-HYDROXYPHYTANOYL-COA[c]'
'PROCOLLAGEN-L-PROLINE[c]'
'CPD-6321[c]'
'DEACETYLCEPHALOSPORIN-C[c]'
'3-HYDROXY-L-PROLINE[c]'
'cis-cis-D19-37-C56-2-ACPs[c]'
'cis-D19-37-OH-38-Me-C57-1-ACPs[c]'
'N-5-PHOSPHORIBOSYL-ANTHRANILATE[c]'
'Phosphoserines[c]'
'Serines[c]'
'Protein-Arginine-Aminocarbinol[c]'
'CYTOSINE[e]'
'Ubiquitin-activating-protein-E1-L-cys[c]'
'pppGp-his-tRNAs[c]'
'pGp-his-tRNAs[c]'
'VOMILENINE[c]'
'CPDMETA-13651[c]'
'CPD-21572[c]'
'Geranylgeranylated-XXCC-proteins[c]'
'CPD-9999[c]'
'L-PHOSPHINOTHRICIN[c]'
'3-HYDROXY-DOCOSAPENTAENOYL-ACP[c]'
'3-OXO-EICOSAPENTAENOYL-ACP[c]'
'CPD-648[c]'
'3-HYDROXY-3-METHYL-GLUTARYL-COA[c]'
'ACRYLONITRILE[c]'
'cis-D19-37-MOH-38-Me-C57-1-ACPs[c]'
'R-3-hydroxyarachidoyl-ACPs[c]'
'trans-D18-37-OH-38-Me-C58-1-ACPs[c]'
'trans-D18-37-MOH-38-Me-C58-1-ACPs[c]'
'trans-18-CP-37-Mex-38-Me-C60-ACPs[c]'
'QUININE[c]'
'3-HYDROXYQUININE[c]'
'4-PHOSPHONOOXY-THREONINE[c]'
'CPD-497[c]'
'3-HYDROXY-ISOVALERYL-COA[c]'
'CPD-10284[c]'
'XXCC-proteins[c]'
'Protein-Cysteine-Hemithioacetal[c]'
'XCXC-proteins[c]'
'Geranylgeranylated-XCXC-proteins[c]'
'CPD-13691[c]'
'CPD-19066[c]'
'CCXX-proteins[c]'
'Geranylgeranylated-CCXX-proteins[c]'
'ENTEROBACTIN[c]'
'FERRIC-ENTEROBACTIN-COMPLEX[c]'
'CPD-12990[c]'
'CINNAMOYL-COA[c]'
'CPD-19105[c]'
'CPD-19106[c]'
'CPD-12874[c]'
'Oxo-glutarate-dehydro-suc-DH-lipoyl[c]'
'tRNA-uridines[c]'
'tRNA-Dihydrouridines[c]'
'7-AMINOMETHYL-7-DEAZAGUANINE[c]'
'tRNA-with-7-aminomethyl-7-deazaguanine[c]'
'Guanine34-in-tRNAs[c]'
'SIROHYDROCHLORIN[c]'
'SIROHEME[c]'
'CPD-11561[c]'
'CPD-11932[c]'
'ACRYLAMIDE[c]'
'3-CYANOPYRIDINE[c]'
'DOCOSAPENTAENOYL-ACP[c]'
'cis-cis-D21-39-C58-2-ACPs[c]'
'cis-D21-39-OH-40-Me-C59-1-ACPs[c]'
'VINORINE[c]'
'cis-D21-39-oxo-40-Me-C59-1-ACPs[c]'
'cis-21-CP-39-keto-40-Me-C60-ACPs[c]'
'trans-D20-39-oxo-40-Me-C60-1-ACPs[c]'
'trans-20-CP-22-Me-39-keto-40-Me-C61-ACPs[c]'
'Purine-Bases[c]'
'CPD-653[c]'
'Aryl-Amines[c]'
'CPD-9522[c]'
'CPD-3762[c]'
'CPD0-2232[c]'
'CPD0-2117[c]'
'CPD-17082[c]'
'N-23-DIHYDROXYBENZOYL-L-SERINE[c]'
'Adenylated-ThiS-Proteins[c]'
'UDP-MANNAC[c]'
'UDP-MANNACA[c]'
'S-ubiquitinyl-UAP-E1-L-cysteine[c]'
'Ubiquitin-C-Terminal-Glycine[c]'
'NN-DIMETHYLANILINE[c]'
'NN-DIMETHYLANILINE-N-OXIDE[c]'
'CPD-2202[c]'
'CREATINE[c]'
'L-HYOSCYAMINE[c]'
'CPD-194[c]'
'CARBAMOYL-SARCOSINE[c]'
'CPD-21345[c]'
'QUEUINE[c]'
'CPD-18762[c]'
'tRNAs-with-queuine[c]'
'CPD-11877[c]'
'TRANS-3-METHYL-GLUTACONYL-COA[c]'
'CPD-17186[c]'
'CPD-17185[c]'
'CPD-13109[c]'
'CPD-17193[c]'
'7-METHYLGUANOSINE-5-PHOSPHATE[c]'
'm7G5-pppR-mRNAs[c]'
'ADENYLOSUCC[c]'
'ANHYDROTETRACYCLINE[c]'
'QUINOLINATE[c]'
'CPD-17206[c]'
'CPD-17207[c]'
'CPD-13108[c]'
'CPD-9116[c]'
'CPD-13294[c]'
'CPD0-2241[c]'
'TDP[c]'
'CPD-1302[c]'
'CPD-1301[c]'
'2-AMINOBENZOYL-COA[c]'
'2-AMINO-5-OXOCYCLOHEX-1-ENECARBOXYL-COA[c]'
'CPD-265[c]'
'CPD-961[c]'
'CPD-17262[c]'
'CPD-17263[c]'
'CPD-17264[c]'
'CPD-5662[c]'
'CPD-21416[c]'
'CPD-21415[c]'
'CPD-21417[c]'
'CPD-21418[c]'
'Cis-2-enoyl-CoAs[c]'
'GLUCOSYL-GLYCOGENIN[c]'
'CPD-7010[c]'
'POLY-GLUCOSYLATED-GLYCOGENINS[c]'
'CPD-375[c]'
'CPD-1112[c]'
'Oxidized-cytochromes-c553[c]'
'Reduced-cytochromes-c553[c]'
'CPD-211[c]'
'2-AMINOBENZENESULFONATE[c]'
'CPD-12565[c]'
'N-methyl-terminal-XPK[c]'
'CPD0-2338[c]'
'Protein-tyrosine-phosphates[c]'
'CPD-13927[c]'
'Protein-Tyrosines[c]'
'CPD-13930[c]'
'2-Arylethylamines[c]'
'N-Acetyl-2-Arylethylamines[c]'
'CPD-470[c]'
'COPROPORPHYRIN\_III[c]'
'N-ACETYL-L-24-DIAMINOBUTANOATE[c]'
'CPD-17070[c]'
'HYDROXYATRAZINE[c]'
'N-ISOPROPYLAMMELIDE[c]'
'ETHANAMINE[c]'
'CPD-21419[c]'
'CPD-21420[c]'
'BIOTIN[e]'
'SALUTARIDINE[c]'
'4-DEOXY-BETA-D-GLUC-4-ENURONOSYL-6S[c]'
'CPD-36[c]'
'N-acetyl-D-galactosamine[c]'
'S-TETRAHYDROBERBERINE[c]'
'CPD-50[c]'
'INDOLE\_ACETALDEHYDE[c]'
'CPD-824[c]'
'AGMATHINE[c]'
'7-8-DIHYDROPTEROATE[c]'
'Farnesylated-CAAX-proteins[c]'
'AMINO-OH-HYDROXYMETHYL-DIHYDROPTERIDINE[c]'
'CAAX-proteins[c]'
'PROTOHEME[c]'
'BETAINE-ALDEHYDE-HYDRATE[c]'
'CPD-21526[c]'
'CPD-825[c]'
'L-SELENOCYSTEINE[c]'
'CPD-374[c]'
'OLEANDOMYCIN[c]'
'CPD-13949[c]'
'CPD-13954[c]'
'CPD-11915[c]'
'PRO-tRNAs[c]'
'Charged-PRO-tRNAs[c]'
'D-PROLINE[c]'
'Monocarboxylic-Acid-Amides[c]'
'CPD-9700[c]'
'PHENYLETHYLAMINE[c]'
'CPD-9699[c]'
'CPD-207[c]'
'CPD-11715[c]'
'6-HYDROXY-NICOTINATE[c]'
'3-METHYLXANTHINE[c]'
'CPD-17328[c]'
'CPD-18492[c]'
'CPD-18493[c]'
'CPD-17365[c]'
'CPD-18494[c]'
'Diphthine-methyl-ester-EF2[c]'
'HISTAMINE[c]'
'N-METHYL-HISTAMINE[c]'
'CPD-15280[c]'
'L-HISTIDINOL-P[c]'
'Acetoacetyl-ACP[c]'
'PHOSPHORIBOSYL-AMP[c]'
'PHOSPHORIBOSYL-FORMIMINO-AICAR-P[c]'
'CPD-21199[c]'
'CPD-21199[e]'
'CPD-21162[c]'
'Long-Chain-3S-Hydroxyacyl-CoAs[c]'
'CPD-21201[c]'
'CPD-21202[c]'
'Long-Chain-oxoacyl-CoAs[c]'
'S-METHYLGLUTATHIONE[c]'
'CPD0-2472[c]'
'Mitogen-Activated-Protein-Kinase-L-Thr[c]'
'MAP-Kinase-L-Phosphothreonine[c]'
'Protein-Histidines[c]'
'CPD-13775[c]'
'L-1-PHOSPHATIDYL-SERINE[c]'
'CPD-13734[c]'
'CPD-9771[c]'
'Aliphatic-Amines[c]'
'CPD-9772[c]'
'CPD-9773[c]'
'CPD-9774[c]'
'CPD-10011[c]'
'CPD-11746[c]'
'CPD-1771[c]'
'3-7-DIMETHYLXANTHINE[c]'
'Cytidine-34-tRNAIle2[c]'
'Lysidine-tRNA-Ile2[c]'
'CPD-21210[c]'
'CPD-21209[c]'
'CPD-21212[c]'
'CPD-17072[c]'
'PURINE[e]'
'PURINE[c]'
'Oxidized-CycA1-cytochromes[e]'
'Reduced-CycA1-cytochromes[e]'
'Protein-L-Arginines[c]'
'Protein-N-Nprime-omega-dimethyl-arginine[c]'
'SALUTARIDINOL[c]'
'HIS-tRNAs[c]'
'Protein-N-omega-dimethyl-arginine[c]'
'Charged-HIS-tRNAs[c]'
'UROCANATE[c]'
'CIS-DELTA3-ENOYL-COA[c]'
'CPD-13776[c]'
'CPD-13777[c]'
'CPD-472[c]'
'CPD-17697[c]'
'CPD-18641[c]'
'Actinorhodin-Intermediate-2[c]'
'Hepta-oxo-hexadecanoyl-ACPs[c]'
'ADP-D-GLUCOSE[c]'
'N-Acylated-Aliphatic-Amino-Acids[c]'
'Aliphatic-L-Amino-Acids[c]'
'CPD-11770[c]'
'CPD-11496[c]'
'CPD-18539[c]'
'CPD-18532[c]'
'Aryl-Carrier-Proteins[c]'
'QXC-ACP[c]'
'CPD-18550[c]'
'CPD-18561[c]'
'AcDMPT-L-Alanyl-PhsB[c]'
'PhsC[c]'
'CPD0-2018[c]'
'CARNITINE[e]'
'CPD-21241[c]'
'CPD-17093[c]'
'Reduced-cytochromes-c551[e]'
'CPD-21134[c]'
'NITRIC-OXIDE[e]'
'Oxidized-cytochromes-c551[e]'
'CPD-21271[c]'
'HISTIDINAL[c]'
'Guanine1575-in-18StRNAs[c]'
'Apo-EntB[c]'
'Holo-EntB[c]'
'CPD-10505[c]'
'Red-Glutaredoxins[c]'
'Ox-Glutaredoxins[c]'
'AcDMPT-L-Alanyl-L-Alanyl-PhsC[c]'
'PhsB[c]'
'AcDMPT-L-Alanyl-L-Leucyl-PhsC[c]'
'PYRIDOXAMINE[c]'
'N-Substituted-Aminoacyl-tRNA[c]'
'N-Acylphosphatidylethanolamines[c]'
'CPD-11852[c]'
'N-Acylethanolamines[c]'
'CPD-13081[c]'
'CPD-13080[c]'
'CPD-11875[c]'
'N7-methylGuanine1575-in-18StRNAs[c]'
'Cytosine2870-in-25S-rRNA[c]'
'5-methylcytosine2870-in-25S-rRNA[c]'
'Cytosine2278-in-25S-rRNA[c]'
'5-methylcytosine2278-in-25S-rRNA[c]'
'25S-rRNA-uracil-2843[c]'
'25S-rRNA-N3-methyl-uracil-2843[c]'
'25S-rRNA-uracil-2634[c]'
'25S-rRNA-N3-methyl-uracil-2634[c]'
'Persulfurated-L-cysteine-desulfurases[c]'
'L-Cysteine-Desulfurases[c]'
'Histone-L-lysine[c]'
'Histone-Acetyl-Lysine[c]'
'CPD-10588[c]'
'CPD-72[c]'
'CPD-10589[c]'
'CPD-10590[c]'
'CPD-10591[c]'
'CPD-606[c]'
'CPD-609[c]'
'UDP-sugar[c]'
'CPD-13781[c]'
'CPD-13841[c]'
'CPD-13783[c]'
'CPD-13845[c]'
'CIS-4-HYDROXY-D-PROLINE[c]'
'CPD-13846[c]'
'CPD-13847[c]'
'CPD-13853[c]'
'CPD-601[c]'
'S-Alkyl-L-Cysteines[c]'
'PROTEIN-L-CITRULLINE[c]'
'tRNAs[c]'
'N-Substituted-Amino-Acids[c]'
'N6-ACETYLKANAMYCIN-B[c]'
'AMINO-PARATHION[c]'
'CPD-597[c]'
'Protein-pi-phospho-L-histidines[c]'
'CPD-18733[c]'
'ALA-tRNAs[c]'
'glycyl-tRNAAla[c]'
'D-tyrosyl-tRNA-Tyr[c]'
'D-TYROSINE[c]'
'TYR-tRNAs[c]'
'D-aspartyl-tRNA-Asp[c]'
'D-Tryptophanyl-tRNA-Trp[c]'
'D-TRYPTOPHAN[c]'
'TRP-tRNAs[c]'
'CPD-21344[c]'
'Apo-EntF[c]'
'Holo-EntF[c]'
'CPD-17136[c]'
'CPD-13855[c]'
'CPD-190[c]'
'LYSOSOMAL-ENZYME-N-ETCETERA-MANNOSE[c]'
'All-apo-ACPs[c]'
'All-holo-ACPs[c]'
'Cytochromes-c[c]'
'HEME\_C[c]'
'CPD0-1137[c]'
'CPD-607[c]'
'CPD-11665[c]'
'CPD-21118[c]'
'CPD-13713[c]'
'G5-pppR-mRNAs[c]'
'CPD-15683[c]'
'B-ALANINE[e]'
'6-AMINOPENICILLANATE[c]'
'6-AMINOPENICILLANATE[e]'
'CPD-12699[c]'
'CPDQT-281[c]'
'5-PHOSPHORIBOSYL-5-AMINOIMIDAZOLE[c]'
'CPDQT-280[c]'
'CPDQT-284[c]'
'CPDQT-420[c]'
'CPDQT-288[c]'
'CPDQT-295[c]'
'CPDQT-296[c]'
'CPDQT-299[c]'
'Protein-ribulosamines[c]'
'Protein-phospho-ribulosamines[c]'
'CPDQT-286[c]'
'CPD-9007[c]'
'CPD-15635[c]'
'CPD-17045[c]'
'CPD-17046[c]'
'CPD-17047[c]'
'CPD-17048[c]'
'CPD-17049[c]'
'CPD-14723[c]'
'CPD-17050[c]'
'CPD-17051[c]'
'ALLANTOATE[c]'
'S-ALLANTOIN[c]'
'GDP-TP[c]'
'GUANOSINE-5DP-3DP[c]'
'5-HYDROXYINDOLE\_ACETALDEHYDE[c]'
'CPD-14724[c]'
'tRNA-uridine-38-39[c]'
'tRNA-pseudouridine-38-39[c]'
'PENICILLIN-N[e]'
'CPD-13717[c]'
'SELENOHOMOCYSTEINE[c]'
'CPD-21122[c]'
'2-AMINOACRYLATE[c]'
'CPD-21126[c]'
'CPD-9300[c]'
'CPD-21160[c]'
'CPD-21166[c]'
'CPDQT-300[c]'
'CPDQT-340[c]'
'L-1-GLYCEROPHOSPHORYLETHANOL-AMINE[c]'
'Iron-Sulfur-Cluster-Scaffold-Proteins[c]'
'CPDQT-429[c]'
'Protein-psicosamines[c]'
'Protein-phospho-psicosamines[c]'
'Protein-fructosamines[c]'
'Protein-phospho-fructosamines[c]'
'Protein-phospho-erythrulosamines[c]'
'Protein-erythrulosamines[c]'
'CPD-13006[c]'
'GLUCOTROPEOLIN[c]'
'Rhodopsins[c]'
'CPD-14432[c]'
'2-methylbutanoyl-LovF[c]'
'Holo-LovF[c]'
'CPD-7302[c]'
'DIHYDRONEOPTERIN-P[c]'
'5-HYDROXYINDOLE\_ACETATE[c]'
'General-Protein-Substrates[e]'
'CPD-11671[c]'
'CPD-8563[c]'
'CPD-8564[c]'
'RBR-Ubiquitin-carrier-protein-E3-L-cys[c]'
'Ubiquitin-carrier-protein-E2-L-cysteine[c]'
'S-ubiquitinyl-UCP-RBR-E3-L-cysteine[c]'
'SELENOMETHIONINE[c]'
'CPD-13754[c]'
'CPD-13755[c]'
'CPD-21167[c]'
'CPDQT-434[c]'
'PROTEIN-N-UBIQUITYL-LYSINE[c]'
'CPD-21172[c]'
'CPD-21171[c]'
'CPD-21170[c]'
'S-ubiquitinyl-UCP-E2-L-cysteine[c]'
'CPD-21169[c]'
'CPD-20867[c]'
'CPD-20943[c]'
'Citrate-Lyase-Citryl-Form[c]'
'CITRATE-LYASE[c]'
'N1-METHYLADENINE[c]'
'an-iNisup1sup-ethyladenine-in-DNA[c]'
'CARBOXYMETHYL-HYDROXYPHENYLPROPCOA[c]'
'DNA-Adenines[c]'
'E-PHENYLITACONYL-COA[c]'
'CPD-18640[c]'
'CPD-65[c]'
'UDP-AA-GLUTAMATE[c]'
'CPD-13018[c]'
'CPD-16953[c]'
'CPD-16954[c]'
'PANTOTHENATE[c]'
'CPD-1776[c]'
'Beta-adrenergic-receptors[c]'
'ATRAZINE[c]'
'CPD-11674[c]'
'CPD-11673[c]'
'PENICILLIN-G[c]'
'4-MHA-THR-VAL-AcmD-Proteins[c]'
'4-HMA-AcmD-Proteins[c]'
'CPD-10303[c]'
'CPD-21178[c]'
'CPD-7547[c]'
'CPD-21179[c]'
'CPD-20053[c]'
'CPD-3705[c]'
'CPD-3709[c]'
'CPD-3713[c]'
'CPD-3710[c]'
'CPD-3723[c]'
'CPD-3725[c]'
'CPD-18639[c]'
'CPD-17696[c]'
'CPD-9517[c]'
'CPD-18763[c]'
'CPD-9518[c]'
'N-5S-5-AMINO-5-CARBOXYPENTANOYL-L-CY[e]'
'CPD-9521[c]'
'CPD-1772[e]'
'CPD-264[c]'
'PENICILLIN-G[e]'
'CPD-16955[c]'
'CPD-16743[c]'
'CPD-16961[c]'
'CPD-16962[c]'
'2-METHYL-ACETO-ACETYL-COA[c]'
'Protein-L-Ser-or-L-Thr-P-L-Pro[c]'
'Protein-L-Ser-or-L-Thr-L-Pro[c]'
'MAPKK-L-serine-or-L-threonine[c]'
'MAPKK-Ser-or-Thr-phosphate[c]'
'Receptor-protein-Ser-or-Thr[c]'
'Receptor-Protein-Ser-or-Thr-phosphate[c]'
'CGMP[c]'
'CPD-17063[c]'
'PROCOLLAGEN-5-HYDROXY-L-LYSINE[c]'
'PROCOLLAGEN-5-GALACTOSYLOXY-L-LYSINE[c]'
'CPD-13026[c]'
'PHENYLACETALDOXIME[c]'
'DOPAQUINONE[c]'
'BENZOYLSUCCINYL-COA[c]'
'BETAINE[e]'
'CPD-21168[c]'
'ISOPENICILLIN-N[e]'
'CPD-21192[c]'
'CPD-21194[c]'
'CYSTINE[e]'
'CPD-9122[e]'
'CPD-9196[e]'
'CPD-21195[c]'
'CPD-21107[c]'
'CPD-237[c]'
'INDOLEYL-CPD[c]'
'PYRIDOXINE-5P[c]'
'PYRIDOXINE[c]'
'CPD-13757[c]'
'CPD-13758[c]'
'CPD-9196[c]'
'Long-Chain-Acyl-ACPs[c]'
'CPD-17329[c]'
'CPD-21411[c]'
'CPD-18489[c]'
'CPD-18490[c]'
'CPD-16969[c]'
'CPD-16970[c]'
'Myosin-heavy-chains[c]'
'Myosin-heavy-chain-phosphates[c]'
'CPD-16971[c]'
'Protein-Phosphoserines[c]'
'CPD-19219[c]'
'CPD-19221[c]'
'CPD-341[c]'
'CPD-13644[c]'
'P-Nitrophenyl-5-Nucleotides[c]'
'CPDMETA-13652[c]'
'GUANIDOACETIC\_ACID[c]'
'CPD0-2107[c]'
'CPDQT-404[c]'
'CPDQT-349[c]'
'CPD-3724[c]'
'CPDQT-256[c]'
'CPD-466[c]'
'CPDQT-277[c]'
'DTDP-D-GLUCOSE[c]'
'Protein-N-acetyl-D-glucosamin-L-serine[c]'
'Protein-N-acetyl-D-glucosamine-L-thr[c]'
'CPD-12541[c]'
'CPD-9134[c]'
'Cyclic-N6-threonylcarbamoyl-A37-tRNAs[c]'
'CPD-9148[c]'
'CPD-9150[c]'
'CPD-19779[c]'
'CPD-6262[c]'
'CPD-19778[c]'
'Palmitoleoyl-ACPs[c]'
'Cis-vaccenoyl-ACPs[c]'
'Protein-N-terminal-5-oxo-prolines[c]'
'Peptide-Holder-Alternative[c]'
'CPDQT-400[c]'
'CPD-17449[c]'
'CPDQT-341[c]'
'CPDQT-343[c]'
'CPDQT-405[c]'
'N-terminal-specific-UCP-E2-L-cysteine[c]'
'S-ubi-N-term-specific-UCP-E2-L-cysteine[c]'
'N-terminal-Amino-Acids[c]'
'N-terminal-ubiquitinyl-proteins[c]'
'CPD-7062[c]'
'CPD-8178[c]'
'4-GUANIDO-BUTYRAMIDE[c]'
'CPD-592[c]'
'CPD-20952[c]'
'CPD-20958[c]'
'CPD-20959[c]'
'METHYL-MALONYL-COA[c]'
'CPD-20961[c]'
'BCAA-dehydrogenase-2MB-DH-lipoyl[c]'
'CPD-1106[c]'
'CPD-1105[c]'
'Aromatic-Amino-Acids[c]'
'CPD-12869[c]'
'CPD-12847[c]'
'CPD-12896[c]'
'CPD-1827[c]'
'CPD-12902[c]'
'CPD-15650[c]'
'6-PYRUVOYL-5678-TETRAHYDROPTERIN[c]'
'CPD-14053[c]'
'BIO-5-AMP[c]'
'Polar-amino-acids[e]'
'Polar-amino-acids[c]'
'OLIGOPEPTIDES[e]'
'OLIGOPEPTIDES[c]'
'CPD-13812[c]'
'CPD-6562[c]'
'CPD-6402[c]'
'CPD-6401[c]'
'INDOXYL[c]'
'CPD-16817[c]'
'PYRIDOXAMINE-5P[c]'
'CPDQT-350[c]'
'CPDQT-262[c]'
'CPDQT-406[c]'
'CPDQT-407[c]'
'CPD-20960[c]'
'CPD-20962[c]'
'CPD-3708[c]'
'CPD0-2189[c]'
'CPD-3706[c]'
'CPD-543[c]'
'CPD0-1327[c]'
'CPD-9122[c]'
'CPD-476[c]'
'CPD-12904[c]'
'CPD-12905[c]'
'CPD-12906[c]'
'Aminated-Amine-Donors[c]'
'CPD-9327[c]'
'ISOPROPYLAMINE[c]'
'CPD-9372[c]'
'CPDQT-408[c]'
'CPDQT-409[c]'
'CPDQT-410[c]'
'CPDQT-411[c]'
'CPDQT-412[c]'
'CPDQT-413[c]'
'CPD-13692[c]'
'CPD-13694[c]'
'CPD-13695[c]'
'CPD-13696[c]'
'CPD0-2253[c]'
'CPD-18195[c]'
'CPD-15121[c]'
'CPD0-2030[c]'
'CPD-18171[c]'
'1-Acyl-sn-glycero-3-phosphocholines[c]'
'1-ACYL-2-OLEOYL-SN-GLYCERO-3-PHOSPHOCHOL[c]'
'Peptides-holder[e]'
'Acylcholines[c]'
'holo-Peptidyl-carrier-proteins[c]'
'CPD-20036[c]'
'CPD-9387[c]'
'CPD-14205[c]'
'CPD-627[c]'
'CPD-6124[c]'
'CPDQT-414[c]'
'CPDQT-415[c]'
'CPDQT-416[c]'
'CPDQT-285[c]'
'CPDQT-417[c]'
'CPDQT-418[c]'
'METHYLENETETRAHYDROMETHANOPTERIN[c]'
'THMPT[c]'
'CPD-13698[c]'
'CPD-13699[c]'
'CPD-13700[c]'
'ATP[e]'
'ADP[e]'
'Alpha-factor[c]'
'Alpha-factor[e]'
'Apo-Aryl-Carrier-Proteins[c]'
'Holo-Aryl-Carrier-Proteins[c]'
'CPD-21068[c]'
'CPD-21108[c]'
'CPD-21113[c]'
'C4[c]'
'CPD-7695[c]'
'CPD-1137[c]'
'CPD0-181[c]'
'CPD-6366[c]'
'CPD-16899[c]'
'CPD-17043[c]'
'CPD-17414[c]'
'CPDQT-419[c]'
'CPD-13578[c]'
'C5[c]'
'C1[c]'
'5-Methylcytosine-DNA[c]'
'CPD-5923[c]'
'CPD-7649[c]'
'GlcA-Gal-Gal-Xyl-Proteins[c]'
'GlcNAc-GlcA-Gal-Gal-Xyl-Protein[c]'
'Proteins-with-N-Terminal-L-Glutamine[c]'
'Lactosyl-Ceramides[c]'
'D-GALACTOSYL-14-D-GALACTOSYL-14-D-[c]'
'CPD-20681[c]'
'Man8GlcNAc2-protein-A123B13[c]'
'Man-16-Man8GlcNAc2-proteins[c]'
'CPD-20682[c]'
'Charged-TYR-tRNAs[c]'
'CPD-12480[c]'
'CPD-16697[c]'
'CPD-10490[c]'
'N-formyl-L-methionyl-tRNAfmet[c]'
'CHITOBIOSE[c]'
'ACETYL-ETCETERA-L-ASPARAGINE[c]'
'N-ACETYL-BETA-GLUCOSAMINYLAMINE[c]'
'CPD-13227[c]'
'CPD-8541[c]'
'Protein-L-Aspartates[c]'
'CPD-571[c]'
'CPD-18240[c]'
'CPD-18265[c]'
'Charged-ALA-tRNAs[c]'
'CPD-18209[c]'
'CPD-18266[c]'
'ALA-GLY[c]'
'CPD-13401[c]'
'CPD-13398[c]'
'CPD-11552[c]'
'CPD-21119[c]'
'CPD-21120[c]'
'1-ACYL-2-LINOLEOYL-SN-GLYCERO-3-PHOSPHOC[c]'
'CPD-20684[c]'
'CPD-20735[c]'
'CPD-20741[c]'
'TYRAMINE[c]'
'DNA-pyrimidines[c]'
'CPD-20742[c]'
'CPD-21121[c]'
'GDP-D-GLUCOSE[c]'
'2-2-HYDROXYACYLSPHINGOSINE[c]'
'1-BETA-D-GALACTOSYL-2-2-HYDROXYACYLS[c]'
'2-ALPHA-HYDROXYETHYL-THPP[c]'
'CPD-12763[c]'
'CPD-16736[c]'
'CPD-16737[c]'
'CPD-16738[c]'
'GLUTAMATE-1-SEMIALDEHYDE[c]'
'Glutamine-synthetase-Tyr[c]'
'Glutamine-synthetase-adenylyl-Tyr[c]'
'CPD-921[c]'
'CPD-10699[c]'
'CPD-8973[c]'
'CPD-13397[...

-------------------------------------------------------------------------------

The chemical element **Na** is present in the empirical formula of **2 metabolites**

'NA+[c]'
'NA+[e]'

-------------------------------------------------------------------------------

The chemical element **O** is present in the empirical formula of **5048 metabolites**

'GLC-1-P[c]'
'ALPHA-GLC-6-P[c]'
'CPD0-1812[c]'
'CPD-15977[c]'
'CO-A[c]'
'WATER[c]'
'MELIBIOSE[c]'
'GALACTOSE[c]'
'ALPHA-GLUCOSE[c]'
'OLEOYL-COA[c]'
'CTP[c]'
'L-1-LYSOPHOSPHATIDATE[c]'
'CDP[c]'
'WATER[e]'
'MELIBIOSE[e]'
'GALACTOSE[e]'
'ALPHA-GLUCOSE[e]'
'GLYCERALD[c]'
'NADP[c]'
'GLYCERATE[c]'
'NADPH[c]'
'OXYGEN-MOLECULE[c]'
'HYDROGEN-PEROXIDE[c]'
'D-GALACTONATE[c]'
'CPD-12575[c]'
'D-Glucosyl-12-diacyl-glycerols[c]'
'diacyl-3-O-glucl-1-6-gluc-sn-glycerol[c]'
'CPD-1070[c]'
'CPD-277[c]'
'MAL[c]'
'PYRUVATE[c]'
'CARBON-DIOXIDE[c]'
'ACETYL-COA[c]'
'GLUCOSAMINE-1P[c]'
'N-ACETYL-D-GLUCOSAMINE-1-P[c]'
'DIACYLGLYCEROL[c]'
'Phospholipids[c]'
'Triacylglycerols[c]'
'GLYCEROL[c]'
'HYDROXYPROPANAL[c]'
'ADENOSINE[c]'
'ATP[c]'
'Odd-Saturated-Fatty-Acyl-CoA[c]'
'INOSINE[c]'
'Odd-Straight-Chain-234-Sat-FA[c]'
'AMP[c]'
'PPI[c]'
'ADP[c]'
'PHYTOL[c]'
'NAD[c]'
'NADH[c]'
'2E-5Z-tetradeca-2-5-dienoyl-ACPs[c]'
'PHOSPHORYL-CHOLINE[c]'
'RHAMNOSE[c]'
'CPD0-1112[c]'
'5Z-tetradec-5-enoyl-ACPs[c]'
'CPD-541[c]'
'D-MYO-INOSITOL-1-MONOPHOSPHATE[c]'
'CPD0-1122[c]'
'CPD0-1123[c]'
'CPD-171[c]'
'DOLICHOLP[c]'
'MANNOSE[c]'
'SULFATE[c]'
'MALONYL-ACP[c]'
'Chondroitin-N-acetyl-galactosamines[c]'
'7Z-3-oxo-hexadec-7-enoyl-ACPs[c]'
'ACP[c]'
'CHONDROITIN-4-SULFATE[c]'
'Heparan-sulfate-L-iduronate[c]'
'Ubiquinones[c]'
'Heparan-sulfate-L-IdoA-2S[c]'
'Heparan-NAc-Glc-6S[c]'
'FERRICYTOCHROME-B5[c]'
'LINOLEIC\_ACID[c]'
'9-CIS11-TRANS-OCTADECADIENOATE[c]'
'CPD-8091[c]'
'FERROCYTOCHROME-B5[c]'
'CPD-8092[c]'
'LINOLENIC\_ACID[c]'
'CPD-2181[c]'
'LINOLENOYL-COA[c]'
'CPD-2182[c]'
'CPD-8088[c]'
'CPD-8093[c]'
'N-ALPHA-ACETYLORNITHINE[c]'
'L-ORNITHINE[c]'
'ACET[c]'
'2-KETOGLUTARATE[c]'
'GLT[c]'
'CPD-469[c]'
'CPD-612[c]'
'CPD-15972[c]'
'Glucopyranose[c]'
'CPD-12384[c]'
'CPD-4578[c]'
'CPD-12385[c]'
'NADH-P-OR-NOP[c]'
'S-ADENOSYLMETHIONINE[c]'
'2-OCTAPRENYL-6-HYDROXYPHENOL[c]'
'CPD-12388[c]'
'2-OCTAPRENYL-6-METHOXYPHENOL[c]'
'ADENOSYL-HOMO-CYS[c]'
'OCTAPRENYL-METHYL-METHOXY-BENZQ[c]'
'CPD-12387[c]'
'4-METHYL-824-CHOLESTADIENOL[c]'
'5-HYDROXY-CONIFERALDEHYDE[c]'
'OCTAPRENYL-METHOXY-BENZOQUINONE[c]'
'SINAPALDEHYDE[c]'
'CPD-4579[c]'
'2-OCTAPRENYLPHENOL[c]'
'CPD-12390[c]'
'CPD-12391[c]'
'CPD-4580[c]'
'CPD-12393[c]'
'CPD-4702[c]'
'CPD-12139[c]'
'CPD-19503[c]'
'3-PHENYLPROPIONATE[c]'
'Linear-Malto-Oligosaccharides[c]'
'CPD-3628[c]'
'CPD-3629[c]'
'Ox-NADPH-Hemoprotein-Reductases[c]'
'CPD-3630[c]'
'CPD-14741[c]'
'N-6-AMINOHEXANOYL-6-AMINOHEXANOATE[c]'
'CPD-884[c]'
'CYCLOARTENOL[c]'
'N1-ACETYLSPERMINE[c]'
'CPD-11271[c]'
'UDP-GLUCURONATE[c]'
'CPD-11398[c]'
'UDP[c]'
'L-THYROXINE[c]'
'LIOTHYRONINE[c]'
'CPD-11400[c]'
'LYS[c]'
'8-AMINO-7-OXONONANOATE[c]'
'DIAMINONONANOATE[c]'
'R-2-HYDROXYGLUTARATE[c]'
'CPD-208[c]'
'PROTEIN-LIPOYLLYSINE[c]'
'MET[c]'
'CH33ADO[c]'
'Octanoylated-Gcv-H[c]'
'CPD-196[c]'
'a-pyruvate-dehydrogenase-E2-protein-Nsup[c]'
'pyruvate-dehydrogenase-E2-lipoyl-carrier[c]'
'DEOXYINOSINE[c]'
'Pi[c]'
'HYPOXANTHINE[c]'
'DEOXY-D-RIBOSE-1-PHOSPHATE[c]'
'DNA-deoxycytidine-thymidine-dimer[c]'
'DNA-Cytidines[c]'
'DNA-thymidines[c]'
'ACETALD[c]'
'GAP[c]'
'DEOXY-RIBOSE-5P[c]'
'GLUTARATE[c]'
'GLUTARYL-COA[c]'
'MALONYL-COA[c]'
'HEXANOYL-COA[c]'
'CPD-14687[c]'
'CPD-280[c]'
'CPD-14615[c]'
'ETF-Oxidized[c]'
'CPD-11507[c]'
'GLUTACONYL-COA[c]'
'ETF-Reduced[c]'
'CPD-11506[c]'
'CPD-18[c]'
'Heparan-NAc-Glc[c]'
'CPD-235[c]'
'NAD-P-OR-NOP[c]'
'56-Dihydrouracil17-in-tRNAs[c]'
'Uracil17-in-tRNAs[c]'
'MALTOSE[c]'
'Glucose[c]'
'56-Dihydrouracil20-in-tRNAs[c]'
'LOGANATE[c]'
'Uracil20-in-tRNAs[c]'
'LOGANIN[c]'
'Isomaltose[c]'
'Long-Chain-234-Saturated-acyl-CoAs[c]'
'56-Dihydrouracil47-in-tRNAs[c]'
'Uracil47-in-tRNAs[c]'
'Long-Chain-Trans-23-Dehydroacyl-CoA[c]'
'biotin-L-lysine-in-BCCP-dimers[c]'
'HCO3[c]'
'carboxybiotin-L-lysine-in-BCCP-dimers[c]'
'CPD-8089[c]'
'CPD-8090[c]'
'BCCP-L-lysine[c]'
'BIOTIN[c]'
'BCCP-biotin-L-lysine[c]'
'1-183-2-183-SN-GLYCEROL-PHOSPHOCHOLINE[c]'
'CPD-8094[c]'
'BIPHENYL-23-DIOL[c]'
'CPD-613[c]'
'CPD-8098[c]'
'CPD-676[c]'
'3R-7Z-3-hydroxy-hexadec-7-enoyl-ACPs[c]'
'2E-7Z-hexadeca-2-7-dienoyl-ACPs[c]'
'7Z-hexadec-7-enoyl-ACPs[c]'
'9Z-3-oxo-octadec-9-enoyl-ACPs[c]'
'Ubiquinols[c]'
'CPD-4581[c]'
'UBIQUINOL-30[c]'
'UBIQUINONE-6[c]'
'Alpha-lactose[c]'
'ALLOLACTOSE[c]'
'ZYMOSTEROL[c]'
'CPD-381[c]'
'CPD0-1158[c]'
'CPD0-1162[c]'
'Oxo-glutarate-dehydrogenase-DH-lipoyl[c]'
'CPD0-1163[c]'
'5-ALPHA-CHOLESTA-724-DIEN-3-BETA-OL[c]'
'CPD-12394[c]'
'CPD-12397[c]'
'CPD-12396[c]'
'CPD-12399[c]'
'CPD-12400[c]'
'CPD-12402[c]'
'CPD-12403[c]'
'CPD-12405[c]'
'CPD-12406[c]'
'BCAA-dehydrogenase-DH-lipoyl[c]'
'PHENYLACETOTHIOHYDROXIMATE[c]'
'CPD-12607[c]'
'CPD-696[c]'
'GAMMA-BUTYROBETAINE[c]'
'CPD-3462[c]'
'SUCC-S-ALD[c]'
'CPD66-39[c]'
'Fatty-Aldehydes[c]'
'Orthophosphoric-Monoesters[c]'
'Alcohols[c]'
'Glucosyl-ceramides[c]'
'2R-Hydroxy-Fatty-Acids[c]'
'N-ACETYLNEURAMINATE[c]'
'N-acetyl-D-mannosamine[c]'
'Ceramides[c]'
'PROTEIN-C-TERMINAL-S-ETC-CYSTEINE[c]'
'PROTEIN-C-TERMINAL-S-FARNESYL-L-CYSTEINE[c]'
'Short-Alpha-14-Glucans[c]'
'DIHYDROLIPOAMIDE[c]'
'CIS-ACONITATE[c]'
'LIPOAMIDE[c]'
'ITACONATE[c]'
'CIT[c]'
'6-DEMETHYLSTERIGMATOCYSTIN[c]'
'STERIGMATOCYSTIN[c]'
'STERIGMATOCYSTIN[e]'
'Fatty-Acids[c]'
'Ribonucleoside-Monophosphates[c]'
'CPD-11401[c]'
'CPD-11402[c]'
'CPD3O-4151[c]'
'PALMITYL-COA[c]'
'CPD-17621[c]'
'CROTONYL-COA[c]'
'GLUTATHIONE[c]'
'OXIDIZED-GLUTATHIONE[c]'
'R-4-PHOSPHOPANTOTHENOYL-L-CYSTEINE[c]'
'PANTETHEINE-P[c]'
'Pyruvate-dehydrogenase-lipoate[c]'
'a-2-oxoglutarate-dehydrogenase-E2-protei[c]'
'2-oxoglutarate-dehydrogenase-E2-lipoyl-c[c]'
'Oxo-glutarate-dehydrogenase-lipoyl[c]'
'SUC[c]'
'PHTYOSPHINGOSINE-1-P[c]'
'Rhodoquinols[c]'
'FUM[c]'
'Rhodoquinones[c]'
'SN-GLYCEROL-1-PHOSPHATE[c]'
'GLYCEROL-3P[c]'
'16-alpha-D-Mannosyloligosaccharides[c]'
'Unbranched-1-6-Mannan[c]'
'DEPHOSPHO-COA[c]'
'L-ALPHA-ALANINE[c]'
'CPD-7000[c]'
'CPD-18077[c]'
'Glc2Man9GlcNAc2-proteins[c]'
'GLC[c]'
'CPD-14704[c]'
'1-4-alpha-D-Glucan[c]'
'MALTOTRIOSE[c]'
'Chitosan[c]'
'CPD-10806[c]'
'Chitosan-fragments[c]'
'ALPHA-MALTOSE[c]'
'CPD-14705[c]'
'PHOSPHORIBOSYL-CARBOXY-AMINOIMIDAZOLE[c]'
'DETHIOBIOTIN[c]'
'L-ASPARTATE[c]'
'Alpha-D-Glucuronides[c]'
'D-Glucopyranuronate[c]'
'CPD-14706[c]'
'GLY[c]'
'P-RIBOSYL-4-SUCCCARB-AMINOIMIDAZOLE[c]'
'CPD-19179[c]'
'GTP[c]'
'Guanine37-in-tRNA[c]'
'tRNA-Containing-N1-Methylguanine-37[c]'
'TMP[c]'
'ADENOSINE5TRIPHOSPHO5ADENOSINE[c]'
'DIHYDROFOLATE-GLU-N[c]'
'Guanine9-in-tRNA[c]'
'tRNA-Containing-N1-Methylguanine-9[c]'
'carbo-me-ur-34-tRNA[c]'
'5-2-me-oxy-2-oxo-et-ur-34-tRNA[c]'
'DPG[c]'
'PRECURSOR-Z[c]'
'23-DIPHOSPHOGLYCERATE[c]'
'CPD-4[c]'
'MPT-Synthase-small-subunits[c]'
'2-METHYL-3-HYDROXY-BUTYRYL-COA[c]'
'CPD-1083[c]'
'Thiocarboxylated-MPT-synthases[c]'
'tRNA-Adenine-58[c]'
'tRNA-Containing-N1-MethylAdenine-58[c]'
'BLASTICIDIN-S[c]'
'DEAMINOHYDROXYBLASTICIDIN-S[c]'
'Adenine57-Adenine58-tRNAs[c]'
'N1-MeAdenine57-MeAdenine58-tRNAs[c]'
'CPD-8122[c]'
'3R-9Z-3-hydroxy-octadec-9-enoyl-ACPs[c]'
'2E-9Z-octadeca-2-9-dienoyl-ACPs[c]'
'3-BETA-D-GLUCOSYLGLUCOSE[c]'
'CPD-1861[c]'
'CPD-4186[c]'
'CPD-4187[c]'
'METOH[c]'
'FORMATE[c]'
'CHOLESTEROL[c]'
'FORMALDEHYDE[c]'
'CPD-12449[c]'
'URACIL[c]'
'PHLORETIN[c]'
'PSEUDOURIDINE-5-P[c]'
'CPD-7727[c]'
'CPD-15317[c]'
'CPD66-21[c]'
'CPD-693[c]'
'LEUKOTRIENE-C4[c]'
'Amino-Acids-20[c]'
'5-L-GLUTAMYL-L-AMINO-ACID[c]'
'CPD-12451[c]'
'CPD-12452[c]'
'7E9E11Z14Z-5S6R-6-CYSTEIN-S-YL[c]'
'MYO-INOSITOL[c]'
'Long-Chain-Aldehydes[c]'
'Long-Chain-Acyl-CoAs[c]'
'CPD-4081[c]'
'ALPHA-METHYL-5-ALPHA-ERGOSTA[c]'
'L-ALLO-THREONINE[c]'
'CPD-4101[c]'
'AMINO-RIBOSYLAMINO-1H-3H-PYR-DIONE[c]'
'DIHYDROXY-BUTANONE-P[c]'
'CPD-19725[c]'
'CPD-7157[c]'
'PELARGONIDIN-CMPD[c]'
'CPD-591[c]'
'DNA-Cytosines[c]'
'DNA-N4-Methylcytosine[c]'
'CPD-19726[c]'
'S-SCOULERINE[c]'
'4-P-PANTOTHENATE[c]'
'S-TETRAHYDROCOLUMBAMINE[c]'
'THREO-DS-ISO-CITRATE[c]'
'3-SULFINOALANINE[c]'
'L-CYSTEATE[c]'
'6-O-METHYLNORLAUDANOSOLINE[c]'
'CPD-15799[c]'
'Myelin-N-o-methyl-arginines[c]'
'R-3-Hydroxypalmitoyl-ACPs[c]'
'PAPS[c]'
'3-5-ADP[c]'
'CPD-11407[c]'
'CPD-11408[c]'
'3-oxo-palmitoyl-ACPs[c]'
'CPD-11409[c]'
'CPD-11403[c]'
'CYS[c]'
'CMP[c]'
'D-6-P-GLUCONO-DELTA-LACTONE[c]'
'RIBOSE[c]'
'RIBOSE-5P[c]'
'DGDP[c]'
'Carboxyadenylated-MPT-synthases[c]'
'L-Cysteine-Desulfurase-persulfide[c]'
'Cysteine-Desulfurase-L-cysteine[c]'
'D-altropyranoses[c]'
'PSICOSE[c]'
'CHITIN[c]'
'ETR-Quinones[c]'
'ETR-Quinols[c]'
'OXALACETIC\_ACID[c]'
'Chitodextrins[c]'
'ITP[c]'
'PHOSPHO-ENOL-PYRUVATE[c]'
'IDP[c]'
'Poly-ADP-Riboses[c]'
'ADENOSINE\_DIPHOSPHATE\_RIBOSE[c]'
'Xyloglucan[c]'
'Xyloglucan-oligosaccharides[c]'
'GLUCOSAMINE[c]'
'Peptidoglycans[c]'
'NAcMur-Peptide-Undecaprenols[c]'
'N-acetyl-D-glucosamine[c]'
'BUTANAL[c]'
'ALPHA-TOCOPHEROL[c]'
'GAMA-TOCOPHEROL[c]'
'RETINAL[c]'
'CPD-17278[c]'
'RETINOATE[c]'
'Alpha-linolenoyl-groups[c]'
'CPD-3[c]'
'3-Oxo-Delta-4-Steroids[c]'
'3-Oxo-5-Alpha-Steroids[c]'
'CPD-8123[c]'
'Mercapturates[c]'
'S-Substituted-L-Cysteines[c]'
'L-GAMMA-GLUTAMYLCYSTEINE[c]'
'3-Beta-Hydroxysterols[c]'
'CPD-18246[c]'
'Malonyl-acp-methyl-ester[c]'
'3-Ketoglutaryl-ACP-methyl-ester[c]'
'ILE[c]'
'2-KETO-3-METHYL-VALERATE[c]'
'PROPIONYL-COA[c]'
'D-METHYL-MALONYL-COA[c]'
'LEU[c]'
'FADH2[c]'
'2K-4CH3-PENTANOATE[c]'
'FAD[c]'
'GLYCOLLATE[c]'
'Folates[c]'
'P-COUMAROYL-COA[c]'
'CAFFEOYL-COA[c]'
'CARNITINE[c]'
'CPD-19737[c]'
'CPD66-29[c]'
'BUTYRYL-COA[c]'
'CPD-19738[c]'
'3-BETA-HYDROXYANDROST-5-EN-17-ONE[c]'
'TETRADECANOYL-COA[c]'
'CPD-19740[c]'
'CPD-10267[c]'
'CPD-19741[c]'
'STEAROYL-COA[c]'
'CPD-19742[c]'
'17-BETA-HYDROXY-5ALPHA-ANDROSTAN-3-O[c]'
'Oleoyl-ACPs[c]'
'ACETYL-ACP[c]'
'11Z-3-oxo-icos-11-enoyl-ACPs[c]'
'CPD-4126[c]'
'L-GLYCERALDEHYDE-3-PHOSPHATE[c]'
'Cytidine-34-tRNAmet[c]'
'CPD-4125[c]'
'CPD-4127[c]'
'Elongator-tRNAMet-acetylcytidine[c]'
'CPD-4142[c]'
'DEHYDRO-3-DEOXY-L-RHAMNONATE[c]'
'CPD-706[c]'
'CPD-4141[c]'
'LACTALD[c]'
'CPD-4143[c]'
'CPD-707[c]'
'Red-NADPH-Hemoprotein-Reductases[c]'
'CPD-3943[c]'
'DIMETHYL-D-RIBITYL-LUMAZINE[c]'
'Myelin-L-arginines[c]'
'S-NORCOCLAURINE[c]'
'S-COCLAURINE[c]'
'DIHYDROSIROHYDROCHLORIN[c]'
'CPD-642[c]'
'METHIONINE-SYNTHASE-METHYLCOBALAMIN[c]'
'Methionine-synthase-cob-II-alamins[c]'
'2-Hexadecenoyl-ACPs[c]'
'Palmitoyl-ACPs[c]'
'GLYOX[c]'
'CPD-11411[c]'
'CPD-11404[c]'
'CPD-11412[c]'
'CPD-11410[c]'
'METHYLENE-THF-GLU-N[c]'
'5-10-METHENYL-THF-GLU-N[c]'
'CPD-1130[c]'
'CPD-3618[c]'
'L-DIHYDROXY-PHENYLALANINE[c]'
'COUMARATE[c]'
'Release-factor-L-glutamine[c]'
'Release-factor-N5-Methyl-L-glutamine[c]'
'CPD-15896[c]'
'CPD-17487[c]'
'DGTP[c]'
'CPD-674[c]'
'2-COUMARATE[c]'
'P3I[c]'
'DEOXYGUANOSINE[c]'
'2-3-DIHYDROXYBENZOATE[c]'
'CPD-664[c]'
'DELTA1-PYRROLINE\_2-CARBOXYLATE[c]'
'3-Oxosteroids[c]'
'CPD-8124[c]'
'CPD-4161[c]'
'L-GULONO-1-4-LACTONE[c]'
'CPD-8134[c]'
'ASCORBATE[c]'
'CPD-8155[c]'
'CPD-7061[c]'
'4-AMINO-BUTYRATE[c]'
'CPD-8157[c]'
'CPD-8158[c]'
'ETOH[c]'
'TYR[c]'
'P-HYDROXY-PHENYLPYRUVATE[c]'
'Peptides-holder[c]'
'CPD-11495[c]'
'PHENYLACETATE[c]'
'CPD0-2244[c]'
'CPD0-2123[c]'
'Beta-D-glucosides[c]'
'Non-Glucosylated-Glucose-Acceptors[c]'
'5-METHYL-THF-GLU-N[c]'
'FORMYL-THF-GLU-N[c]'
'D-glucopyranose-6-phosphate[c]'
'Beta-D-Galactosides[c]'
'Non-Galactosylated-Galactose-Acceptors[c]'
'THF[c]'
'D-galactopyranose[c]'
'LAUROYLCOA-CPD[c]'
'LYS-tRNAs[c]'
'CPD-19743[c]'
'Charged-LYS-tRNAs[c]'
'CHOLINE[c]'
'GLYCOLALDEHYDE[c]'
'CPD-7682[c]'
'4-HYDROXY-BUTYRYL-COA[c]'
'OH-CROTONYL-COA[c]'
'3-Hydroxyglutaryl-ACP-methyl-ester[c]'
'3-P-SERINE[c]'
'CPD-19754[c]'
'ECTOINE[c]'
'CPD-19757[c]'
'CPD-10663[c]'
'CPD-19760[c]'
'2-Lysophosphatidylcholines[c]'
'HYDROQUINONE[c]'
'Enoylglutaryl-ACP-methyl-esters[c]'
'Carboxylates[c]'
'L-1-GLYCERO-PHOSPHORYLCHOLINE[c]'
'26-DICHLORO-P-HYDROQUINONE[c]'
'VAL[c]'
'2-KETO-ISOVALERATE[c]'
'PYRROLINE-HYDROXY-CARBOXYLATE[c]'
'4-HYDROXY-L-PROLINE[c]'
'ACYL-COA[c]'
'TESTOSTERONE[c]'
'3R-11Z-3-hydroxy-icos-11-enoyl-ACPs[c]'
'CPD66-23[c]'
'2E-11Z-icosa-2-11-dienoyl-ACPs[c]'
'11Z-icos-11-enoyl-ACPs[c]'
'CPD66-27[c]'
'CYS-tRNAs[c]'
'CPD-698[c]'
'ACETYLSERINE[c]'
'CPD-3945[c]'
'CPD-4162[c]'
'CPD-4181[c]'
'4-ALPHA-METHYL-5-ALPHA[c]'
'CPD-763[c]'
'METHYLARSONATE[c]'
'DIMETHYLARSINATE[c]'
'METHYLARSONITE[c]'
'CPD-4124[c]'
'CPD-225[c]'
'E-2-METHOXYCARBONYLMETHYLBUTENEDIOAT[c]'
'L-1-PHOSPHATIDYL-ETHANOLAMINE[c]'
'CPD-10260[c]'
'DIHYDRO-DIOH-BENZOATE[c]'
'CPD-10261[c]'
'CPD-10262[c]'
'THF-GLU-N[c]'
'CPD-9956[c]'
'DIHYDRO-NEO-PTERIN[c]'
'DIHYDRONEOPTERIN-P3[c]'
'OCTAPRENYL-METHYL-OH-METHOXY-BENZQ[c]'
'DIHYDROFOLATE[c]'
'Stearoyl-ACPs[c]'
'2-CHLOROETHANOL[c]'
'GERANYLGERANYL-PP[c]'
'LysW-L-glutamate[c]'
'LysW-L-glutamate-5-phosphate[c]'
'18-HYDROXYOLEATE[c]'
'LysW-L-glutamate-5-semialdehyde[c]'
'Lipid-hydroxy-fatty-acids[c]'
'CPD-21340[c]'
'A-LIPID-HYDROPEROXIDE[c]'
'910-EPOXY-18-HYDROXYSTEARATE[c]'
'GERANYL-PP[c]'
'DELTA3-ISOPENTENYL-PP[c]'
'CPD-11444[c]'
'COPROPORPHYRINOGEN\_I[c]'
'LysW-L-ornithine[c]'
'ALL-TRANS-HEXAPRENYL-DIPHOSPHATE[c]'
'CPD-15900[c]'
'3-HYDROXYPIMELYL-COA[c]'
'CPD-11447[c]'
'L-ARABINOSE[e]'
'CPD-7733[c]'
'L-ARABINOSE[c]'
'CPD-15913[c]'
'Cis-Delta5-dodecenoyl-ACPs[c]'
'L-GULONATE[c]'
'D-GLUCURONOLACTONE[c]'
'2-OXOBUTANOATE[c]'
'2-ACETO-2-HYDROXY-BUTYRATE[c]'
'Charged-GLT-tRNAs[c]'
'GLT-tRNAs[c]'
'5-HYDROXY-FERULIC-ACID[c]'
'SINAPATE[c]'
'DOPAMINE[c]'
'ARABINOSE-5P[c]'
'RIBULOSE-5P[c]'
'Saturated-Fatty-Acyl-CoA[c]'
'G3P[c]'
'CPD-8620[c]'
'CPD-8619[c]'
'CPD-8621[c]'
'NOREPINEPHRINE[c]'
'L-EPINEPHRINE[c]'
'DCDP[c]'
'DCTP[c]'
'CPD-621[c]'
'CPD-8065[c]'
'CPD-170[c]'
'CPD-8066[c]'
'CPD-1099[c]'
'SUCROSE[c]'
'CPD-8073[c]'
'CPD-8074[c]'
'CPD-8075[c]'
'CPD-8076[c]'
'CPD-12335[c]'
'CPD-12336[c]'
'CPD-12334[c]'
'CPD-14468[c]'
'E-11-TETRADECENOYL-COA[c]'
'CPD-17811[c]'
'CPD-17814[c]'
'CPD-17813[c]'
'L-LACTATE[c]'
'CPD-17815[c]'
'3-OXOPALMITOYL-COA[c]'
'CPD-17464[c]'
'CPD-358[c]'
'tRNA-Containing-N2-Dimethylgua-26-Gua27[c]'
'tRNA-Containing-N2-Methylgua-26-Gua27[c]'
'tRNA-Containing-N2-Dimetgua-26-MeGua27[c]'
'tRNA-Containing-N2-DiMeGua-26-DiMeGua27[c]'
'Guanine26-Guanine27-in-tRNAs[c]'
'Dodecanoyl-ACPs[c]'
'3-oxo-myristoyl-ACPs[c]'
'CPD-15684[c]'
'CPD-15685[c]'
'CPD-19273[c]'
'CPD-15686[c]'
'CPD-15687[c]'
'CPD-15688[c]'
'SO3[c]'
'S2O3[c]'
'CPD-15689[c]'
'CPD-15690[c]'
'NYSTOSE[c]'
'1-KESTOTRIOSE[c]'
'CPD-15692[c]'
'CPD-15691[c]'
'H2CO3[c]'
'IMIDAZOLE\_ACETALDEHYDE[c]'
'CPD-14465[c]'
'CPD-14459[c]'
'CPD-14467[c]'
'4-IMIDAZOLEACETATE[c]'
'CPD-10847[c]'
'CPD-12358[c]'
'CPD-14471[c]'
'CPD-10844[c]'
'ACETOACETYL-COA[c]'
'2-Oxo-carboxylates[c]'
'L-GLUTAMATE\_GAMMA-SEMIALDEHYDE[c]'
'INOSITOL-1-4-5-TRISPHOSPHATE[c]'
'INOSITOL-1-4-BISPHOSPHATE[c]'
'PRO[c]'
'D-MYO-INOSITOL-4-PHOSPHATE[c]'
'PHOSPHATIDYLINOSITOL-345-TRIPHOSPHATE[c]'
'PHOSPHATIDYL-MYO-INOSITOL-45-BISPHOSPHA[c]'
'GUANOSINE[c]'
'GUANINE[c]'
'BENZALDEHYDE[c]'
'BENZOATE[c]'
'5-METHYLTHIOADENOSINE[c]'
'ALPHA-L-GLUTAMYL-PHOSPHATE[c]'
'S-ADENOSYLMETHIONINAMINE[c]'
'CARBAMATE[c]'
'BENZOYLCOA[c]'
'CPD-20052[c]'
'3-CARBOXY-3-HYDROXY-ISOCAPROATE[c]'
'CPD-318[c]'
'CPD-20051[c]'
'DCMP[c]'
'DUMP[c]'
'GlgE-Glycogen[c]'
'2K-ADIPATE[c]'
'DAIDZEIN[c]'
'CPD-8646[c]'
'DESMOSTEROL-CPD[c]'
'Red-Thioredoxin[c]'
'CPD-465[c]'
'CPD-10204[c]'
'Ox-Thioredoxin[c]'
'GDP-MANNOSE[c]'
'GDP-L-GALACTOSE[c]'
'D-LACTATE[c]'
'4-hydroxybenzoate[c]'
'CPD-7875[c]'
'4-Hydroxy-3-polyprenylbenzoates[c]'
'Polyisoprenyl-Diphosphates[c]'
'2-3-CARBOXY-3-AMINOPROPYL-L-HISTIDINE[c]'
'DTDP-DEOH-DEOXY-GLUCOSE[c]'
'L-Galactopyranose[c]'
'CPD-13952[c]'
'2-3-CARBOXY-3-METHYLAMMONIOPROPYL-L-[c]'
'CPD-9326[c]'
'eEF-2-Histidines[c]'
'CPD-17870[c]'
'3-carboxy-3-dimethylammonio-propyl-L-his[c]'
'DIPHTINE[c]'
'CPD-17877[c]'
'EPOXYSQUALENE[c]'
'LANOSTEROL[c]'
'CPD-17876[c]'
'CPD-2189[c]'
'CPD-8078[c]'
'CPD-330[c]'
'CPD-8080[c]'
'CPD-2190[c]'
'CPD-8077[c]'
'CPD-8079[c]'
'CPD-6948[c]'
'CPD-9459[c]'
'CPD-259[c]'
'CPD-8130[c]'
'CPD-9777[c]'
'CPD-9775[c]'
'CPD-401[c]'
'CPD-1823[c]'
'B-ALANINE[c]'
'XANTHOSINE-5-PHOSPHATE[c]'
'XANTHINE[c]'
'PRPP[c]'
'URATE[c]'
'CPD-1103[c]'
'4-HYDROXYBENZALDEHYDE[c]'
'CPD-7207[c]'
'CPD-13371[c]'
'GERANIAL[c]'
'CPD-13376[c]'
'CPD-13375[c]'
'CPD-13377[c]'
'CELLULOSE[c]'
'Cellulose-D-glucono-1-5-lactone[c]'
'Cytochromes-B-Oxidized[c]'
'Cytochromes-B-Reduced[c]'
'Cellodextrins[c]'
'CPD-7043[c]'
'CPD-3746[c]'
'FMNH2[c]'
'FMN[c]'
'D-ALPHABETA-D-HEPTOSE-7-PHOSPHATE[c]'
'T2-DECENOYL-COA[c]'
'CPD-10849[c]'
'CPD-10845[c]'
'CPD-12777[c]'
'D-arabinofuranose[c]'
'D-arabinopyranose[c]'
'CPD-9152[c]'
'L-arabinofuranose[c]'
'CPD-10870[c]'
'L-arabinopyranose[c]'
'MANNOSE-6P[c]'
'CPD-15711[c]'
'CPD-1241[c]'
'CPD-15712[c]'
'CPD-10866[c]'
'CPD-9151[c]'
'DUTP[c]'
'D-CYSTEINE[c]'
'FORMONONETIN[c]'
'VESTITONE[c]'
'2-HYDROXYFORMONONETIN[c]'
'CPD-3441[c]'
'ACETYL-GLU[c]'
'CPD-217[c]'
'CPD-3402[c]'
'CPD-3502[c]'
'2-HYDROXYISOFLAVANONE[c]'
'OROTIDINE-5-PHOSPHATE[c]'
'GLN[c]'
'OROTATE[c]'
'2-DEOXY-D-GLUCOSE-6-PHOSPHATE[c]'
'2-DEOXY-D-GLUCOSE[c]'
'PYRIDOXAL[c]'
'D-BETA-D-HEPTOSE-1-P[c]'
'PYRIDOXAL\_PHOSPHATE[c]'
'CPD-9001[c]'
'CPD-9002[c]'
'CPD-85[c]'
'CPD-8999[c]'
'CPD-10637[c]'
'CPD0-1080[c]'
'CPD0-1081[c]'
'CPD0-1082[c]'
'2-METHYL-BUTYRYL-COA[c]'
'N-ACETYL-D-GLUCOSAMINE[c]'
'CPD0-882[c]'
'L-ALA-GAMMA-D-GLU-DAP[c]'
'D-ALANINE[c]'
'4-TRIMETHYLAMMONIOBUTANAL[c]'
'Benzoin[c]'
'CPD-9869[c]'
'3-HYDROXY-N6N6N6-TRIMETHYL-L-LYSINE[c]'
'CPD-9871[c]'
'PHENYLGLYOXYLATE[c]'
'CPD-17873[c]'
'CPD-17874[c]'
'CPD-8082[c]'
'CPD-8084[c]'
'CPD-16968[c]'
'CPD-17881[c]'
'CPD-17880[c]'
'CPD-17882[c]'
'CPD-8081[c]'
'CPD-8083[c]'
'CPD-9873[c]'
'CPD-4568[c]'
'LEU-tRNAs[c]'
'Charged-LEU-tRNAs[c]'
'Sphingoids[c]'
'Sphingoid-1-phosphates[c]'
'CPD0-1083[c]'
'CPD-12349[c]'
'L-GLYCERALDEHYDE[c]'
'CPD-460[c]'
'CPD-12352[c]'
'CPD-14594[c]'
'LINAMARIN[c]'
'CPD-19388[c]'
'CPD-15277[c]'
'CYS-GLY[c]'
'GLYCYLGLYCINE[c]'
'CPD-19395[c]'
'CPD-13031[c]'
'CPD-12702[c]'
'INDOLE-3-ACETALDOXIME[c]'
'CPD-13378[c]'
'R-3-hydroxymyristoyl-ACPs[c]'
'RIBOSE-1P[c]'
'XANTHOSINE[c]'
'CPD-13417[c]'
'CPD-13418[c]'
'CPD0-1202[c]'
'XYLOSE[c]'
'CPD-10868[c]'
'CPD-294[c]'
'VERY-LONG-CHAIN-FATTY-ACYL-COA[c]'
'CPD-15723[c]'
'CPD-10615[c]'
'3-phosphooligonucleotides[c]'
'3-Prime-Nucleoside-Monophosphates[c]'
'CPD-15728[c]'
'Oligonucleotides[c]'
'Nucleoside-Monophosphates[c]'
'24-DICHLOROPHENOL[c]'
'CPD-8924[c]'
'CPD-15741[c]'
'L-1-phosphatidyl-inositols[c]'
'CPD-1121[c]'
'ALTROSE[c]'
'CPD-15781[c]'
'CPD-397[c]'
'CPD-15801[c]'
'CPD-377[c]'
'25-DIDEHYDRO-D-GLUCONATE[c]'
'ALLO-THR[c]'
'DIHYDROXY-ACETONE-PHOSPHATE[c]'
'TARTRONATE-S-ALD[c]'
'DEOXYCYTIDINE[c]'
'BENZYL-ALCOHOL[c]'
'3-OH-BENZYL-ALCOHOL[c]'
'3-OH-BENZALDEHYDE[c]'
'LEUCOPELARGONIDIN-CMPD[c]'
'BETA-CYCLOPIAZONATE[c]'
'ALPHA-CYCLOPIAZONATE[c]'
'UMP[c]'
'ACETYLCHOLINE[c]'
'PHOSPHORIBULOSYL-FORMIMINO-AICAR-P[c]'
'NONANE-46-DIONE[c]'
'Acetate-esters[c]'
'D-ERYTHRO-IMIDAZOLE-GLYCEROL-P[c]'
'CPD-4573[c]'
'44-DIMETHYL-CHOLESTA-814-24-TRIENOL[c]'
'44-DIMETHYL-824-CHOLESTADIENOL[c]'
'CPD-622[c]'
'CPD-1136[c]'
'CPD-8087[c]'
'CPD-8086[c]'
'CATECHOL[c]'
'NITRITE[c]'
'CPD-12356[c]'
'CPD-12359[c]'
'CPD0-1905[c]'
'CPD-12365[c]'
'D-XYLONATE[c]'
'2-DH-3-DO-D-ARABINONATE[c]'
'D-XYLULOSE[c]'
'XYLULOSE-5-PHOSPHATE[c]'
'Very-Long-Chain-Aldehydes[c]'
'Secondary-Alcohols[c]'
'CARBON-MONOXIDE[c]'
'CPD-15798[c]'
'CPD-19475[c]'
'2-OXO-5-METHYLTHIOPENTANOIC-ACID[c]'
'CPD-8347[c]'
'PALMITATE[c]'
'CPDQT-40[c]'
'CPD-14596[c]'
'CPD-15800[c]'
'CPD-10277[c]'
'CPD-15742[c]'
'CPDQT-39[c]'
'CPD-14601[c]'
'CPD-14602[c]'
'CPD-19488[c]'
'PHOSPHATIDYLCHOLINE[c]'
'CPD-14604[c]'
'CPDQT-38[c]'
'CPD-19489[c]'
'Tetradec-2-enoyl-ACPs[c]'
'DEOXYADENOSINE[c]'
'DAMP[c]'
'DADP[c]'
'2-DEHYDRO-3-DEOXY-D-GLUCONATE[c]'
'2-KETO-3-DEOXY-6-P-GLUCONATE[c]'
'AICAR[c]'
'Charged-THR-tRNAs[c]'
'Cyclic-3-5-Nucleoside-Monophosphates[c]'
'THR[c]'
'CPD-35[c]'
'D-THREONINE[c]'
'Cyclic-2-3-Ribonucleoside-Monophosphates[c]'
'AMINO-OXOBUT[c]'
'2-Prime-Ribonucleoside-Monophosphates[c]'
'AMINO-ACETONE[c]'
'GLN-tRNAs[c]'
'3Z-dodec-3-enoyl-ACPs[c]'
'OXALATE[c]'
'OXALYL-COA[c]'
'CPD-1162[c]'
'GLC-6-P[c]'
'CPD-1181[c]'
'3R-5Z-3-hydroxy-tetradec-5-enoyl-ACPs[c]'
'5Z-3-oxo-tetradec-5-enoyl-ACPs[c]'
'CPD-173[c]'
'BUTYRIC\_ACID[c]'
'PENTAN-2-ONE[c]'
'Beta-D-Glucuronides[c]'
'Beta-Lactams[c]'
'CPD-8550[c]'
'CPD-448[c]'
'3-UREIDO-PROPIONATE[c]'
'N-ACETYL-GLUTAMYL-P[c]'
'CPD-4575[c]'
'CPD-4576[c]'
'CPD-667[c]'
'HOMO-CYS[c]'
'CPD-4577[c]'
'CPD-10254[c]'
'CPD-8892[c]'
'245-DNOL[c]'
'SUC-COA[c]'
'25-DDOL[c]'
'3-KETO-ADIPYL-COA[c]'
'CPD-258[c]'
'CPD-320[c]'
'BENZENE-NO2[c]'
'CPD-12364[c]'
'PHE[c]'
'CPD-12363[c]'
'CPD-12521[c]'
'CPD-12595[c]'
'CPD-12377[c]'
'CPD-19490[c]'
'MALEATE[c]'
'CPDQT-37[c]'
'CPD-11268[c]'
'CPD-568[c]'
'CPD-10687[c]'
'CPDQT-36[c]'
'CPD-19491[c]'
'CPD-19492[c]'
'ACYL-ACP[c]'
'ACYL-SN-GLYCEROL-3P[c]'
'CPD-3740[c]'
'CPD-19493[c]'
'Omega-methylthio-alkyl-glucosinolates[c]'
'CPD-30[c]'
'omega-methylsulfinylalkylglucosinolate[c]'
'CPD-6082[c]'
'L-DELTA1-PYRROLINE\_5-CARBOXYLATE[c]'
'Protein-Phosphothreonines[c]'
'Proteins-L-Threonines[c]'
'CPD-15834[c]'
'CPD-11712[c]'
'ALLYSINE[c]'
'CAAL-proteins[c]'
'Geranylgeranylated-CAAL-proteins[c]'
'CPD-9539[c]'
'L-DEHYDRO-ASCORBATE[c]'
'OXAMATE[c]'
'DGMP[c]'
'CPD-389[c]'
'CARBAMOYL-P[c]'
'CPD-316[c]'
'RIBOFLAVIN[c]'
'CPD-9973[c]'
'LONG-CHAIN-KETONE[c]'
'EIF5A-HYPUSINE[c]'
'Charged-GLN-tRNAs[c]'
'CPD-3617[c]'
'O-PHOSPHO-L-HOMOSERINE[c]'
'THYMIDINE[c]'
'Myristoyl-ACPs[c]'
'THYMINE[c]'
'D-SEDOHEPTULOSE-7-P[c]'
'D-RIBULOSE[c]'
'CPD-15567[c]'
'CPD-15568[c]'
'PROPIONAMIDE[c]'
'BUTYRAMIDE[c]'
'CPD-3707[c]'
'PROPIONATE[c]'
'CPD-8548[c]'
'CPD-8549[c]'
'CPD-369[c]'
'CPD-15616[c]'
'TETRACHLOROHYDROQUINONE[c]'
'236-TRICHLOROHYDROQUINONE[c]'
'Cytochromes-C-Oxidized[c]'
'Cytochromes-C-Reduced[c]'
'CPD-13025[c]'
'CPD-13223[c]'
'TRANS-2-HEXENOL[c]'
'TRANS-2-HEXENAL[c]'
'CIS-3-HEXENAL[c]'
'CIS-3-HEXENOL[c]'
'Pullulans[c]'
'7-O-ACETYLSALUTARIDINOL[c]'
'CPD-7710[c]'
'CPD-7712[c]'
'CPD-7713[c]'
'CODEINONE[c]'
'MORPHINONE[c]'
'CPD-10802[c]'
'CPD-10783[c]'
'METOH[e]'
'MYO-INOSITOL[e]'
'CPD-7836[e]'
'CPD-10784[c]'
'N-ACETYL-D-GLUCOSAMINE[e]'
'CPD-10803[c]'
'NADH-P-OR-NOP[e]'
'NADP[e]'
'CPD-10780[c]'
'NADPH[e]'
'CPD-10804[c]'
'NIACINAMIDE[e]'
'CPD-10785[c]'
'NIACINE[e]'
'NITRATE[e]'
'CPD-10805[c]'
'2-METHYL-6-SOLANYL-14-BENZOQUINONE[c]'
'MALONATE-S-ALD[c]'
'CPD-14553[c]'
'CPD0-935[c]'
'CPD0-936[c]'
'CPD0-937[c]'
'CPD0-938[c]'
'CPD-17722[c]'
'CPD-17747[c]'
'OLEATE-CPD[c]'
'CPD-17726[c]'
'CPD-17727[c]'
'CPD-17728[c]'
'Amino-Acids[c]'
'CPD-17729[c]'
'2-Oxo-Acids[c]'
'Deoxyhypusine-Synthase-Lysine[c]'
'N-4-aminobutylidene-enzyme-lysine[c]'
'CPD-17732[c]'
'EIF5A-LYSINE[c]'
'CPD-17733[c]'
'DODECANOATE[c]'
'N-4-aminobutylidene-eIF5A-lysine[c]'
'General-Protein-Substrates[c]'
'CPD-17741[c]'
'Decanoyl-ACPs[c]'
'DNA-with-3-prime-pp-5-prime-G-cap[c]'
'3-Prime-Phosphate-Terminated-DNAs[c]'
'GMP[c]'
'3-oxo-dodecanoyl-ACPs[c]'
'DNA-Ligase-L-lysine-adenylate[c]'
'DNA-Ligase-L-lysine[c]'
'A-5-prime-PP-5-prime-DNA[c]'
'O-SUCCINYLBENZOATE[c]'
'CPD-9923[c]'
'GLC-D-LACTONE[c]'
'GLUCONATE[c]'
'CPD-13469[c]'
'CPD-55[c]'
'FRUCTOSE-6P[c]'
'CPD-8611[c]'
'2-ACETO-LACTATE[c]'
'CPD-231[c]'
'CPD-8612[c]'
'CPD-2750[c]'
'D-Xylopyranose[c]'
'CPD-2742[c]'
'CPD-360[c]'
'CPD-409[c]'
'1-2-Diglycerides[c]'
'CPD-2752[c]'
'CPD-468[c]'
'XYLITOL[c]'
'ANDROST4ENE[c]'
'NITRITE[e]'
'CPD-8505[e]'
'OXYGEN-MOLECULE[e]'
'CPD-10174[c]'
'CPD-10174[e]'
'CPD-195[e]'
'OLEATE-CPD[e]'
'OXALATE[e]'
'OXALACETIC\_ACID[e]'
'PALMITATE[e]'
'PANTOTHENATE[e]'
'CPD-8462[e]'
'CPD-10902[e]'
'PHENYLACETATE[e]'
'PLASTOQUINONE[c]'
'Plastoquinols[c]'
'CPD-13205[c]'
'CELLOBIOSE[c]'
'Phosphatase-2A-leucine[c]'
'Phosphatase-2A-leucine-methyl-ester[c]'
'SARCOSINE[c]'
'CODEINE[c]'
'MORPHINE[c]'
'CPD-4592[c]'
'CPD-4592[e]'
'ACETAMIDE[c]'
'D-Galactopyranuronate[c]'
'CPD-15633[c]'
'CPD-219[c]'
'CPD-15666[c]'
'2-KETO-6-AMINO-CAPROATE[c]'
'CPD-10809[c]'
'DIAMINO-OH-PHOSPHORIBOSYLAMINO-PYR[c]'
'CPD-1086[c]'
'Lignoceroyl-ACPs[c]'
'3-oxo-cerotoyl-ACPs[c]'
'TRINAPHTAL-CPD[c]'
'CPD-17743[c]'
'CPD-17730[c]'
'S-HYDROXYMETHYLGLUTATHIONE[c]'
'CPD-17744[c]'
'CPD-17746[c]'
'CPD-17750[c]'
'CPD-548[c]'
'CPD-17757[c]'
'CPD-13122[c]'
'CPD-702[c]'
'CPD-703[c]'
'FARNESYL-PP[c]'
'CPD0-1028[c]'
'MALTOHEXAOSE[c]'
'MALTOTETRAOSE[c]'
'CPD-3745[c]'
'CPD-201[c]'
'CPD-202[c]'
'DIACYLGLYCEROL-PYROPHOSPHATE[c]'
'L-PHOSPHATIDATE[c]'
'DNA-N[c]'
'CHOLATE[c]'
'CPD-14388[c]'
'3-Hydroxy-Terminated-DNAs[c]'
'Deacetylated-Peptidoglycan[c]'
'NICOTINAMIDE\_NUCLEOTIDE[c]'
'CPD-9776[c]'
'DNA-Ligase-L-lysine-guanylate[c]'
'CPD-14389[c]'
'CPD1F-140[c]'
'tRNA-precursors[c]'
'SS-Oligoribonucleotides[c]'
'CPD-236[c]'
'5-Phospho-terminated-DNAs[c]'
'CPD-14390[c]'
'Pi[e]'
'CPD-2751[c]'
'D-GLUCOSAMINE-6-P[c]'
'CPD-205[e]'
'PROPIONATE[e]'
'N-ACETYL-D-GLUCOSAMINE-6-P[c]'
'CPD-2747[c]'
'PYRUVATE[e]'
'CPD-3188[c]'
'QUINATE[e]'
'CPD-1099[e]'
'CPD-8613[c]'
'Red-NADPH-Hemoprotein-Reductases[e]'
'CPD-2749[c]'
'RIBOFLAVIN[e]'
'R-3-hydroxydodecanoyl-ACPs[c]'
'OCTANOL[c]'
'CPD-371[c]'
'CPD-7616[c]'
'3-4-DIHYDROXYBENZOATE[c]'
'THZ-P[c]'
'AMINO-HYDROXYMETHYL-METHYLPYRIMIDINE-PP[c]'
'THIAMINE-P[c]'
'THIAMINE-PYROPHOSPHATE[c]'
'CPD-611[c]'
'THIAMINE[c]'
'THZ[c]'
'2-CARBOXY-D-ARABINITOL[c]'
'2-CARBOXY-D-ARABINITOL-1-PHOSPHATASE[c]'
'ACETYL-P[c]'
'3-KETOBUTYRATE[c]'
'L-PIPECOLATE[c]'
'Alpha-1-3-Branched-Arabinans[c]'
'L-RHAMNONATE[c]'
'L-RHAMNONO-14-LACTONE[c]'
'CPD-7850[c]'
'GERANIOL[c]'
'CPD-7857[c]'
'CPD-7860[c]'
'CPD-7849[c]'
'CPD-15637[c]'
'CPD-15653[c]'
'CPD-15668[c]'
'CPD-15667[c]'
'CPD-15654[c]'
'CPD-15655[c]'
'CPD-13665[c]'
'5-BETA-ANDROSTANE-317-DIONE[c]'
'CPD-125[c]'
'CPD-14077[c]'
'R-3-hydroxycerotoyl-ACPs[c]'
'NONAPRENYL-4-HYDROXYBENZOATE[c]'
'Trans-D2-hexacos-2-enoyl-ACPs[c]'
'CPD-107[c]'
'CPD-9863[c]'
'Cerotoyl-ACPs[c]'
'CPD-14392[c]'
'Sphingomyelins[e]'
'CPD-11541[c]'
'STEARIC\_ACID[e]'
'CPD-14018[c]'
'Sterols[e]'
'5Z8Z11Z14Z17Z-EICOSAPENTAENOATE[c]'
'Steryl-Esters[e]'
'SUC[e]'
'SUCROSE[e]'
'CPD-6951[c]'
'SULFATE[e]'
'FERULOYL-COA[c]'
'CPD-501[c]'
'UTP[c]'
'UDP-D-GALACTURONATE[c]'
'CPD-8633[c]'
'CPD-8634[c]'
'CPD-12231[c]'
'CPD-12261[c]'
'CPD-15377[c]'
'CPD-9868[c]'
'CPD-12279[c]'
'CPD-108[c]'
'CPD-9866[c]'
'RNA-DNA-hybrids[c]'
'DNA-Holder[c]'
'CPD-2961[c]'
'RNA-Containing-Guanosine[c]'
'RNA-3prime-Guanosine-3prime-P[c]'
'5Prime-OH-Terminated-RNAs[c]'
'G-5-prime-PP-5-prime-DNA[c]'
'3-KETOACYL-COA[c]'
'CPD-9872[c]'
'CPD-9870[c]'
'RNA-Ligase-L-lysine[c]'
'L-3-HYDROXYACYL-COA[c]'
'RNA-Ligase-L-lysine-adenylate[c]'
'PORPHOBILINOGEN[c]'
'5-Phospho-RNA[c]'
'HYDROXYMETHYLBILANE[c]'
'A-5-prime-PP-5-prime-RNA[c]'
'RNA-Holder[c]'
'3Prime-OH-Terminated-RNAs[c]'
'DATP[c]'
'ERYTHROSE-4P[c]'
'3-DEOXY-D-ARABINO-HEPTULOSONATE-7-P[c]'
'Oxidized-Flavoproteins[c]'
'Reduced-Flavoproteins[c]'
'ALPHA-GLUCOSE-16-BISPHOSPHATE[c]'
'CPD-8614[c]'
'CPD-3483[c]'
'CPD-3481[c]'
'GLC-D-LACTONE[e]'
'Glucopyranose[e]'
'CPD-4184[c]'
'2-DEHYDROPANTOATE[c]'
'L-PANTOATE[c]'
'HMP[c]'
'CROTONATE[c]'
'Dodec-2-enoyl-ACPs[c]'
'Thiocarboxyadenylated-ThiS-Proteins[c]'
'CPD-13575[c]'
'SO3[e]'
'ACETOIN[c]'
'L-RHAMNOFURANOSE[c]'
'DIACETYL[c]'
'TARTRATE[e]'
'THIAMINE[e]'
'Triacylglycerides[e]'
'UBIQUINONE-6[e]'
'UBIQUINONE-8[e]'
'URACIL[e]'
'CPD-10353[c]'
'UREA[e]'
'L-XYLULOSE[c]'
'URIDINE[e]'
'CPD-13357[c]'
'VALERATE[e]'
'CPD-7952[c]'
'CPD-7951[c]'
'STRICTOSIDINE-AGLYCONE[c]'
'CPD-21552[c]'
'GEISSOSCHIZINE[c]'
'POLYNEURIDINE-ALDEHYDE[c]'
'CPD-7117[c]'
'CPD-7139[c]'
'HIF-alpha-subunit-L-asparagines[c]'
'HIF-alpha-subunit-3S-OH-ASN[c]'
'CPD-8815[c]'
'CPD-15895[c]'
'4-METHYLCATECHOL[c]'
'CPD-12288[c]'
'CPD-10664[c]'
'Glucosyl-acyl-sphinganines[c]'
'Glucosyl-acyl-sphingosines[c]'
'R-6-HYDROXYNICOTINE[c]'
'3-Prime-Phosphate-Terminated-RNAs[c]'
'CPD-14407[c]'
'CPD-8120[c]'
'CPD0-2350[c]'
'Pre-tRNA-5-prime-half-molecules[c]'
'Pre-tRNA-3-prime-half-molecules[c]'
'CPD-17794[c]'
'CPD-17791[c]'
'CPD-14422[c]'
'CPD-14423[c]'
'CPD-14424[c]'
'CPD-17793[c]'
'Protein-Ser-or-Thr-phosphate[c]'
'Protein-L-serine-or-L-threonine[c]'
'CPD-14425[c]'
'CPD-14426[c]'
'CPD-13328[c]'
'SCOPOLETIN[c]'
'CPD-15656[c]'
'CPD-3041[c]'
'CPD-15657[c]'
'PYRIDOXAL[e]'
'CPD-3061[c]'
'CPD-15675[c]'
'MALONATE[c]'
'CPD-15651[c]'
'CPD-15652[c]'
'BUTANOL[c]'
'CPD-13346[c]'
'CPD-15677[c]'
'CPD-15676[c]'
'AMINO-HYDROXYMETHYL-METHYL-PYR-P[c]'
'DNA-3-methyladenines[c]'
'DNA-containing-aPurinic-Sites[c]'
'QUINOLINATE[e]'
'CPD-57[c]'
'RNA-with-3-prime-pp-5-prime-A-cap[c]'
'Cyclic-Phosphate-Terminated-RNAs[c]'
'RNA-3-prime-P-cyclase-L-histidine[c]'
'RNA-3-prime-P-cyclase-L-His-adenylate[c]'
'Nucleosides[c]'
'Ribonucleosides[c]'
'CPD-8617[c]'
'CPD-8618[c]'
'N-Acylsphingosine[c]'
'CPD-15530[c]'
'XANTHINE[e]'
'Xylans[e]'
'XYLITOL[e]'
'Amino-Acids-20[e]'
'Nucleosides[e]'
'2-HYDROXY-2-METHYLPROPANENITRILE[c]'
'ACETONE[c]'
'1-KETO-2-METHYLVALERATE[c]'
'BETAINE\_ALDEHYDE[c]'
'S-2-Haloacids[c]'
'PALMITALDEHYDE[c]'
'CPD-292[c]'
'CPD-5164[c]'
'Thi-S[c]'
'DEOXYXYLULOSE-5P[c]'
'CPD-17883[c]'
'CPD-5165[c]'
'CPD-17884[c]'
'CPD-5166[c]'
'D-ALA-D-ALA[c]'
'CPD-17885[c]'
'CPD-5167[c]'
'S-ADENOSYL-4-METHYLTHIO-2-OXOBUTANOATE[c]'
'CPD-17799[c]'
'5-AMINOPENTANOATE[c]'
'CPD-17787[c]'
'CPD-17800[c]'
'CPD-17887[c]'
'CPD-17801[c]'
'CPD-12303[c]'
'UDP-N-ACETYL-D-GLUCOSAMINE[c]'
'CPD-12304[c]'
'CPD-17802[c]'
'CPD-7993[c]'
'CPD-12258[c]'
'CPD-9646[c]'
'CPD-17805[c]'
'CPD-12311[c]'
'CPD-12310[c]'
'CPD-17888[c]'
'Z-11-TETRADECENOYL-COA[c]'
'CPD-5168[c]'
'CPD-17807[c]'
'CPD-17809[c]'
'COUMARIN[c]'
'DIHYDROCOUMARIN[c]'
'Uridine44-in-tRNA-Ser[c]'
'2-O-Methyluridine44-tRNASer[c]'
'Guanine10-in-tRNA[c]'
'tRNA-Containing-N2-Methylguanine-10[c]'
'Guanine26-in-tRNA[c]'
'tRNA-Containing-N2-Methylguanine-26[c]'
'tRNA-Containing-N2-dimethylguanine-26[c]'
'CPD-17858[c]'
'CPD-17894[c]'
'GDP[c]'
'L-CANALINE[c]'
'UREA[c]'
'CANAVANINE[c]'
'CPD-15661[c]'
'CPD-10825[c]'
'CPD-10826[c]'
'CPD-10832[c]'
'CPD-15662[c]'
'CPD-15678[c]'
'CPD-15663[c]'
'CPD-9407[c]'
'CPD-15658[c]'
'CPD0-1308[c]'
'CPD0-1074[c]'
'CPD-13792[c]'
'Short-Chain-Trans-23-Dehydroacyl-CoA[c]'
'Short-Chain-234-Saturated-acyl-CoAs[c]'
'Very-Long-Chain-Trans-23-Dehydroacyl-CoA[c]'
'CPD-19268[c]'
'Very-long-Chain-234-Saturated-acyl-CoAs[c]'
'12-DEHYDROTETRACYCLINE[c]'
'CPD-19274[c]'
'CPD-19272[c]'
'VAL-tRNAs[c]'
'Charged-VAL-tRNAs[c]'
'BETAINE[c]'
'DICARBOXYLIC-ACID-MONOAMIDES[c]'
'L-CITRULLINE[c]'
'CPD-19474[c]'
'Pyruvate-Dehydrogenase-Phosphoserine[c]'
'Pyruvate-dehydrogenase-L-serine[c]'
'CPD-535[c]'
'CPD-19486[c]'
'FRUCTOSE-2-PHOSPHATE[c]'
'CPD-22266[c]'
'HYDROXYBENZOQUINONE[c]'
'CPD-22267[c]'
'NITRATE[c]'
'Methyl-esterified-homogalacturonan[c]'
'1-4-alpha-D-galacturonosyl[c]'
'CPD-19487[c]'
'TRYPANOTHIONE-DISULFIDE[c]'
'TRYPANOTHIONE[c]'
'Dihydro-Lipoyl-Proteins[c]'
'Lipoyl-Protein-N6-lipoyllysine[c]'
'D-GLT[c]'
'D-Amino-Acids[c]'
'N-ACETYL-D-AMINO-ACID[c]'
'CPD-7418[c]'
'CPD-7417[c]'
'3-Hydroxy-octanoyl-ACPs[c]'
'3-Oxo-octanoyl-ACPs[c]'
'PHYTOSPINGOSINE[c]'
'CPD-15382[e]'
'tRNAPhe-Containing-4-demethylwyosine-37[c]'
'D-GALACTONO-1-4-LACTONE[e]'
'ALPHA-D-GALACTOSE[e]'
'yW-86[c]'
'Octadec-2-enoyl-ACPs[c]'
'GLUCOSAMINE[e]'
'yW-58[c]'
'tRNAPhe-wybutosine[c]'
'yW-72[c]'
'CPD-15438[e]'
'n-Alkanals[c]'
'MANNITOL[e]'
'Alk-2-enals[c]'
'2-HEXAPRENYL-6-METHOXY-14-BENZOQUINOL[c]'
'MANNOSE[e]'
'2-HEXAPRENYL-3-METHYL-6-METHOXY-14-BENZ[c]'
'OHyWstar-tRNA[c]'
'OHyW-58-tRNAPhe[c]'
'HYDANTOIN[c]'
'N-CARBAMOYLGLYCINE[c]'
'BUTANEDIOL[c]'
'CPD-111[c]'
'CPD-8782[c]'
'CPD-8781[c]'
'SULFO-CYSTEINE[c]'
'GDP-4-DEHYDRO-6-DEOXY-D-MANNOSE[c]'
'CPD-11281[c]'
'421-DEHYDROGEISSOSCHIZINE[c]'
'N-5S-5-AMINO-5-CARBOXYPENTANOYL-L-CY[c]'
'Apo-Propionyl-CoA-CO2-ligases[c]'
'Propionyl-CoA-CO2-ligases[c]'
'3-methylcrotonoyl-CoA-carboxylase-lysine[c]'
'Biotin-EC6-4-1-4[c]'
'CPD-14280[c]'
'CPD-10279[c]'
'CPD-14281[c]'
'CPD-10280[c]'
'CPD-14282[c]'
'NMNH[c]'
'CPD0-881[c]'
'CPD1G-277[c]'
'CPD-14283[c]'
'CPDQT-520[c]'
'CPD-2183[c]'
'CPD-14300[c]'
'CANAVANINOSUCCINATE[c]'
'CPD-479[c]'
'2-2-METHYLTHIOETHYLMALIC-ACID[c]'
'3-2-METHYLTHIOETHYLMALIC-ACID[c]'
'HOMOMETHIONINE[c]'
'STEARIC\_ACID[c]'
'N-Ac-L-methionyl-L-tyrosinyl-Protein[c]'
'N-Ac-N-terminal-L-valine[c]'
'N-terminal-L-valine[c]'
'N-terminal-L-alanine[c]'
'N-terminal-N-Ac-L-alanine[c]'
'16-HYDROXYPALMITATE[c]'
'N-terminal-L-cysteine[c]'
'N-terminal-N-Ac-L-cysteine[c]'
'CPD-9406[c]'
'CPD-17635[c]'
'N-terminal-glycine[c]'
'N-terminal-N-Ac-glycine[c]'
'N-terminal-N-Ac-L-Serine[c]'
'N-terminal-L-Serine[c]'
'CoM[c]'
'2-OXOPROPYL-COM[c]'
'PRENAL[c]'
'S-PRENYL-L-CYSTEINE[c]'
'Protein-L-methionine-R-S-oxides[c]'
'Protein-L-methionine[c]'
'CPD-8989[c]'
'RIBOSE[e]'
'D-Xylose[e]'
'CPD-16017[e]'
'ETOH[e]'
'CPD-320[e]'
'FERROCYTOCHROME-B5[e]'
'FMN[e]'
'Folates[e]'
'FORMATE[e]'
'OHyW-tRNAPhe[c]'
'ARG[c]'
'CPD-15413[c]'
'25S-rRNA-adenine-2142[c]'
'25S-rRNA-N1-methyladenine-2142[c]'
'25S-rRNA-adenine-645[c]'
'25S-rRNA-N1-methyladenine-645[c]'
'S-palmitoyl-L-cysteine-in-proteins[c]'
'PROT-CYS[c]'
'CPD-7556[c]'
'2-Octenoyl-ACPs[c]'
'apo-Transcarboxylases[c]'
'CPD-355[c]'
'CPD-569[c]'
'L-aspartyl-tRNAAsn[c]'
'Octanoyl-ACPs[c]'
'Charged-ASN-tRNAs[c]'
'CPD-356[c]'
'L-glutamyl-tRNAGln[c]'
'CPD-220[c]'
'CPD-13174[c]'
'CPD-13172[c]'
'SUPER-OXIDE[c]'
'5-HYDROXY-FERULOYL-COA[c]'
'4-SULFOBENZALDEHYDE[c]'
'CPD-257[c]'
'CPD-12180[c]'
'CPD-6602[c]'
'O-Long-Chain-Acyl-L-Carnitines[c]'
'SINAPOYL-COA[c]'
'3-HYDROXY-CISCIS-MUCONATE[c]'
'18S-rRNA-pseudouridine-1191[c]'
'3-HEXAPRENYL-4-HYDROXYBENZOATE[c]'
'18S-rRNA-N1-methylpseudouridine-1191[c]'
'CPD-21768[c]'
'VLC-Alpha-hydroxyphytoceramides[c]'
'IPC[c]'
'VLC-MIPC[c]'
'CPD-14305[c]'
'DI-H-OROTATE[c]'
'CPD-21769[c]'
'CPD-21770[c]'
'CPD-14304[c]'
'2-HEXAPRENYL-3-METHYL-5-HYDROXY-6-METHOX[c]'
'CPD-1108[c]'
'CPD-633[c]'
'3-METHYLTHIOPROPANALDOXIME[c]'
'CPD-7546[c]'
'3-METHYLTHIOPROPYLHYDROXAMIC-ACID[c]'
'3-METHYLTHIOPROPYL-DESULFO-GLUCOSINOLATE[c]'
'3-METHYLTHIOPROPYL-GLUCOSINOLATE[c]'
'3-METHYLSULFINYLPROPYL-GLUCOSINOLATE[c]'
'2-PROPENYL-GLUCOSINOLATE[c]'
'CPD-8990[c]'
'FUM[e]'
'4-AMINO-BUTYRATE[e]'
'Glucose[e]'
'GLUTATHIONE[e]'
'GLYCEROL[e]'
'Xylans[c]'
'GLY[e]'
'P-BENZOQUINONE[c]'
'Single-Stranded-DNAs[c]'
'Ribonucleoside-Triphosphates[c]'
'Glycogens[e]'
'ssDNA-RNA-primer-hybrid[c]'
'GLYCOLLATE[e]'
'GUANINE[e]'
'APS[c]'
'CAMP[c]'
'HYDROGEN-PEROXIDE[e]'
'CPD-7830[e]'
'L-ARABITOL[c]'
'CPD-1117[c]'
'CPD-1134[c]'
'O-UREIDOHOMOSERINE[c]'
'CPD-7652[c]'
'DIHYDROPTERIN-CH2OH-PP[c]'
'CPD-10766[c]'
'Protein-N-terminal-N-Ac-L-threonine[c]'
'Protein-N-terminal-L-threonine[c]'
'N-terminal-L-Serine-Histone-H2A[c]'
'N-terminal-N-Ac-L-Serine-Histone-2A[c]'
'Nucleotides[c]'
'N-terminal-L-Serine-Histone-H4[c]'
'N-terminal-N-Ac-L-Serine-Histone-4[c]'
'L-methionyl-L-lysyl-Protein[c]'
'N-Ac-L-methionyl-L-lysyl-Protein[c]'
'L-methionyl-L-valyl-Protein[c]'
'N-Ac-L-methionyl-L-valyl-Protein[c]'
'L-methionyl-L-alanyl-Protein[c]'
'N-Ac-L-methionyl-L-alanyl-Protein[c]'
'Nucleoside-Triphosphates[c]'
'FRUCTOSE-16-DIPHOSPHATE[c]'
'CPD-17640[c]'
'CPD-656[c]'
'CPD-17370[c]'
'CPD-7557[c]'
'CPD-7558[c]'
'CPD-15435[c]'
'N6-L-threonylcarbamoyladenine37-tRNAs[c]'
'tRNA-adenine-37[c]'
'CPD-15438[c]'
'L-rhamnopyranose[c]'
'CPD-15440[c]'
'CPD-15436[c]'
'Butanoyl-ACPs[c]'
'3-oxo-decanoyl-ACPs[c]'
'SULFOACETALDEHYDE[c]'
'CPD-10794[c]'
'3-HYDROXYADIPYL-COA[c]'
'TRANS-23-DEHYDROADIPYL-COA[c]'
'5-METHYLTHIOINOSINE[c]'
'1-PHOSPHATIDYL-1D-MYO-INOSITOL-34-BISPH[c]'
'2-PG[c]'
'CHOCOLA\_A[c]'
'CPD-13524[c]'
'CPD-13175[c]'
'MET[e]'
'CPD-452[c]'
'SALICYLALDEHYDE[c]'
'CPD-1113[c]'
'ERGOSTEROL[c]'
'CPD-11602[c]'
'CPD-882[c]'
'CPD-881[c]'
'CPD-14355[c]'
'CPD-160[c]'
'CPD-13955[c]'
'CPD-12199[c]'
'CPD-7619[e]'
'HYPOXANTHINE[e]'
'ARACHIDIC\_ACID[e]'
'Inulin[e]'
'THREO-DS-ISO-CITRATE[e]'
'CPD-468[e]'
'Alpha-lactose[e]'
'CPD-15972[e]'
'L-ALPHA-ALANINE[e]'
'L-ARABITOL[e]'
'ARG[e]'
'L-methionyl-L-seryl-Protein[c]'
'N-Ac-L-methionyl-L-seryl-Protein[c]'
'L-methionyl-L-threonyl-Protein[c]'
'N-Ac-L-methionyl-L-threonyl-Protein[c]'
'Aminopeptidase-Substrates[c]'
'L-methionyl-L-cysteinyl-Protein[c]'
'L-methionyl-glycyl-Protein[c]'
'CPD-8198[c]'
'IMINOASPARTATE[c]'
'L-Fucopyranoses[c]'
'METHYL-BETA-D-GALACTOSIDE[c]'
'CPD-730[c]'
'CPD-729[c]'
'1-3-beta-D-Glucans[c]'
'CPD-17642[c]'
'CPD-17644[c]'
'CPD-17641[c]'
'PHOSPHORIBOSYL-ATP[c]'
'CPD-17643[c]'
'CPD-17638[c]'
'CPD-17647[c]'
'CPD-9570[c]'
'CPD-357[c]'
'METHYL-GLYOXAL[c]'
'ASN[e]'
'CPD-7545[c]'
'L-ASPARTATE[e]'
'CPD1F-135[c]'
'CPD-15414[c]'
'DODECANOATE[e]'
'CPD-7424[c]'
'CPD-15423[c]'
'L-CITRULLINE[e]'
'CYS[e]'
'CPD-15502[c]'
'GLT[e]'
'CPD-15467[c]'
'GLN[e]'
'HIS[e]'
'INDOLE\_PYRUVATE[c]'
'TRP[c]'
'HOMO-CYS[e]'
'CPD-15522[c]'
'CPD-369[e]'
'CPD-15521[c]'
'CPD-7618[c]'
'CHOLANATE2[c]'
'LIPOIC-ACID[e]'
'CPD-10797[c]'
'CPD-10796[c]'
'CPD-15554[c]'
'CPD-335[c]'
'CPD-10781[c]'
'3-OXODODECANOATE[c]'
'TAGATOSE-1-6-DIPHOSPHATE[c]'
'CPD-7706[c]'
'O-Acyl-L-Carnitines[e]'
'O-Acyl-L-Carnitines[c]'
'Beta-hydroxydecanoyl-ACPs[c]'
'CPD-8606[c]'
'CPD-184[c]'
'4-OXALOMESACONATE[c]'
'Acetylxylan[c]'
'Feruloyl-polysaccharides[c]'
'4-OH-4-ACETYL-2-OXOGLUTARATE[c]'
'CPD-10600[c]'
'FERULIC-ACID[c]'
'CPD-13181[c]'
'CPD-13182[c]'
'CPD-3571[c]'
'HOMO-SER[c]'
'Enones[c]'
'DMPBQ[c]'
'CPD-13187[c]'
'CPD-13188[c]'
'16-EPIVELLOSIMINE[c]'
'MPBQ[c]'
'CPD-665[c]'
'DELTA-TOCOPHEROL[c]'
'CPD-19217[c]'
'S-NITROSOGLUTATHIONE[c]'
'CPD-13617[c]'
'Charged-ARG-tRNAs[c]'
'L-Glutamyl-Peptides[c]'
'Lipoyl-Protein-L-Lysine[c]'
'CPD-195[c]'
'Octanoylated-domains[c]'
'N-ETHYLMALEIMIDE[c]'
'CPD0-903[c]'
'ILE[e]'
'LEU[e]'
'5-KETO-4-DEOXY-D-GLUCARATE[c]'
'D-GLUCARATE[c]'
'LYS[e]'
'Very-long-chain-fatty-acids[c]'
'L-ORNITHINE[e]'
'CPD-17624[c]'
'PHE[e]'
'CPD-15382[c]'
'PRO[e]'
'L-RIBULOSE[e]'
'OCTADEC-9-ENE-118-DIOIC-ACID[c]'
'SER[e]'
'Phosphoacetylglucosamine-Mutase[c]'
'Phosphoacetylglucosamine-Mutase-P[c]'
'N-ACETYL-D-GLUCOSAMINE-16-BIS-P[c]'
'CPD-9570[e]'
'THR[e]'
'CPD-17714[c]'
'CPD-17701[c]'
'CPD-8607[c]'
'CPD-8608[c]'
'ALPHA-HYDROXY-915-DIOXOPROSTANOATE[c]'
'HYDROXY-915-DIOXOPROSTA-13-ENOATE[c]'
'CPD-308[c]'
'CPD-15524[c]'
'CPD-15523[c]'
'CPD-33[c]'
'DEHYDFUC-CPD[c]'
'CPD-15566[c]'
'TAGATOSE-6-PHOSPHATE[c]'
'CPD-1118[c]'
'CPD-183[c]'
'TARTRATE[c]'
'CPD-66[c]'
'CPD-7620[c]'
'CPD-7619[c]'
'CPD-10799[c]'
'CPD-10782[c]'
'THIOHYDROXIMATE-O-SULFATES[c]'
'CPD-558[c]'
'Alkenyl-Thiohydroximate-O-Sulfates[c]'
'CPD-10800[c]'
'CDPDIACYLGLYCEROL[c]'
'L-1-PHOSPHATIDYL-GLYCEROL[c]'
'CARDIOLIPIN[c]'
'CPD-10801[c]'
'S-RETICULINE[c]'
'12-DEHYDRORETICULINIUM[c]'
'ACROLEIN[c]'
'CPD-7600[c]'
'CPD-8847[c]'
'MEK[c]'
'CPD-13218[c]'
'CPD-13220[c]'
'CPD-13222[c]'
'TRP[e]'
'Acyl-homoserine-lactones[c]'
'Acyl-homoserines[c]'
'TYR[e]'
'VAL[e]'
'CPD-12364[e]'
'Deoxy-Ribonucleoside-Monophosphates[c]'
'Maltodextrins[e]'
'MALTOSE[e]'
'MALTOTRIOSE[e]'
'BETA-TOCOPHEROL[c]'
'CONIFERYL-ALCOHOL[c]'
'CONIFERYL-ALDEHYDE[c]'
'SINAPYL-ALCOHOL[c]'
'CPD-12829[c]'
'L-arginyl-L-Glutamyl-Peptides[c]'
'ARG-tRNAs[c]'
'Proteins-With-N-Terminal-Asp[c]'
'L-arginyl-L-aspartyl-Peptides[c]'
'TTP[c]'
'L-arginyl-3-sulfino-L-alaninyl-Peptides[c]'
'N-terminal-L-cysteine-sulfinate[c]'
'N-terminal-L-cysteine-sulfonate[c]'
'SER[c]'
'L-arginyl-3-sulfo-L-alaninyl-Peptides[c]'
'N-Ac-L-methionyl-L-asparaginyl-Protein[c]'
'N-terminal-asparagine[c]'
'CPD0-2015[c]'
'L-Glutaminyl-Peptides[c]'
'L-RIBULOSE-5-P[c]'
'N-Ac-L-methionyl-L-glutaminyl-Protein[c]'
'Trans-D2-decenoyl-ACPs[c]'
'L-RIBULOSE[c]'
'UROPORPHYRINOGEN-III[c]'
'DIMETHYL-GLYCINE[c]'
'5-DEHYDROGLUCONATE[c]'
'CPD-13473[c]'
'CPD-17723[c]'
'CPD-17724[c]'
'CPD-17725[c]'
'CPD-8609[c]'
'CPD-8610[c]'
'O-SUCCINYL-L-HOMOSERINE[c]'
'L-CYSTATHIONINE[c]'
'CPD-3187[c]'
'CPD-18798[c]'
'CPD-14092[c]'
'CPD-6972[c]'
'CPD-22025[c]'
'CPD-22027[c]'
'CPD-22028[c]'
'CPD-15189[c]'
'CPD-22029[c]'
'VLC-Ceramides[c]'
'CPD-10556[c]'
'Ultra-Long-Chain-Acyl-CoAs[c]'
'ULC-Cermaides[c]'
'CPD-13612[c]'
'CPD-22033[c]'
'CPD-18797[c]'
'3-OXO-5-BETA-CHOLANATE[c]'
'57222428-ERGOSTATETRAENOL[c]'
'CPD-18825[c]'
'ISO-PROPANOL[c]'
'SACCHAROPINE[c]'
'SECOLOGANIN-CPD[c]'
'CPD-7247[c]'
'ISOVALERYL-COA[c]'
'BCAA-dehydrogenase-3MB-DH-lipoyl[c]'
'CPD-18826[c]'
'CPD-18831[c]'
'CPD-18832[c]'
'CPD-12524[c]'
'CPD-17138[c]'
'CPD-9038[c]'
'Cytochromes-C-Reduced[e]'
'CPD-4211[c]'
'CPD-15265[c]'
'Cytochromes-C-Oxidized[e]'
'CPD-15263[c]'
'TREHALOSE[c]'
'CPD-1862[c]'
'CPD-15268[c]'
'ENT-COPALYL-DIPHOSPHATE[c]'
'CPD-15260[c]'
'CPD-602[c]'
'CPD-15259[c]'
'CPD-15261[c]'
'Ergothioneine[c]'
'CPD-19154[c]'
'CPD-19161[c]'
'CPD-11571[c]'
'CPD-19157[c]'
'CPD-12101[c]'
'CPD-19148[c]'
'3-oxo-hexanoyl-ACPs[c]'
'12-apo-Carotenals[c]'
'9-cis-Epoxycarotenoids[c]'
'CPD-7279[c]'
'CPD-7280[c]'
'CPD-7196[c]'
'3-OXOADIPATE-ENOL-LACTONE[c]'
'3-KETO-ADIPATE[c]'
'CPD1F-130[c]'
'R-3-hydroxyhexanoyl-ACPs[c]'
'CPD1F-131[c]'
'D-GALACTONO-1-4-LACTONE[c]'
'CPD-155[c]'
'GALACTOSE-1P[c]'
'N-SUCCINYL-2-AMINO-6-KETOPIMELATE[c]'
'N2-SUCCINYLORNITHINE[c]'
'CPD-822[c]'
'CPD-725[c]'
'L-methionyl-tRNAfmet[c]'
'NN-dimethyl-terminal-XPK[c]'
'NNN-trimethyl-terminal-XPK[c]'
'CPD-17434[c]'
'CYTIDINE[c]'
'D-Ribofuranose[c]'
'N-terminal-XPK[c]'
'CYTOSINE[c]'
'N-terminal-PPK[c]'
'NN-dimethyl-terminal-PPK[c]'
'CPD-17428[c]'
'N-methyl-terminal-PPK[c]'
'CPD-17052[c]'
'CPD-17453[c]'
'CPD66-40[c]'
'CPD-22034[c]'
'CPD-22035[c]'
'FECOSTEROL[c]'
'CPD-9965[c]'
'CPD-22036[c]'
'CPD-16352[c]'
'URIDINE[c]'
'CPD1F-133[c]'
'HISTIDINOL[c]'
'HIS[c]'
'CPD-7390[c]'
'CPD-7392[c]'
'Deoxy-Ribonucleoside-Diphosphates[c]'
'Ribonucleoside-Diphosphates[c]'
'COPROPORPHYRINOGEN\_III[c]'
'Oxidized-NrdH-Proteins[c]'
'Reduced-NrdH-Proteins[c]'
'CPD-205[c]'
'CPD-101[c]'
'CPD-15285[c]'
'CPD0-2298[c]'
'CPD-1091[c]'
'CPD-1863[c]'
'CPD-1881[c]'
'L-4-HYDROXYGLUTAMATE\_SEMIALDEHYDE[c]'
'3-DEHYDRO-SHIKIMATE[c]'
'UDP-D-GALACTO-14-FURANOSE[c]'
'L-Amino-Acids[c]'
'5-OXOPROLINE[c]'
'5-L-GLUTAMYL-PEPTIDE[c]'
'5-L-GLUTAMYL-AMINO-ACID[c]'
'Hex-2-enoyl-ACPs[c]'
'CPD-17455[c]'
'CPD-19150[c]'
'CPD-17053[c]'
'CPD-19151[c]'
'CPD-17457[c]'
'CPD-3631[c]'
'CPD-19153[c]'
'CPD-17458[c]'
'CPD-3944[c]'
'CPD-3942[c]'
'CPD-8678[c]'
'BETA-D-FRUCTOSE[c]'
'3-SULFINYL-PYRUVATE[c]'
'Kanamycin-3-phosphates[c]'
'Kanamycins[c]'
'DE-O-GLUCONATE[c]'
'DE-O-K-GLUCONATE[c]'
'CPD-16353[c]'
'CPD-22039[c]'
'CPD-14293[c]'
'CPD-22040[c]'
'D-3-HYDROXYACYL-COA[c]'
'CPD-22041[c]'
'CPD-14269[c]'
'CPD-22043[c]'
'CPD-15363[c]'
'3-oxo-stearoyl-ACPs[c]'
'HOMO-CIT[c]'
'HOMO-CIS-ACONITATE[c]'
'EPISTEROL[c]'
'CPD-307[c]'
'THIOMORPHOLINE-3-CARBOXYLATE[c]'
'34-DEHYDRO-14-THIOMORPHOLINE-3-CARBOXY[c]'
'R-RETICULINE[c]'
'CPD-2022[c]'
'CPD-102[c]'
'L-ERYTHRO-4-HYDROXY-GLUTAMATE[c]'
'CPD-637[c]'
'CPD-112[c]'
'CPD-15978[c]'
'CPD-15361[c]'
'L-LACTATE[e]'
'CPD-1072[c]'
'MAL[e]'
'1-3-beta-D-Glucans[e]'
'CPD-9446[e]'
'2-KETOGLUTARATE[e]'
'P-AMINO-BENZOATE[e]'
'CPD-1302[e]'
'6-DEMETHYLSTERIGMATOCYSTIN[e]'
'Red-Thioredoxin[e]'
'ACP[e]'
'SHIKIMATE[c]'
'CPDQT-273[c]'
'CPD-12140[c]'
'ARSENATE[c]'
'CPD1G-2[c]'
'CPD-12152[c]'
'CPD-12156[c]'
'NICOTINAMIDE\_RIBOSE[c]'
'Pyrimidine-Nucleosides[c]'
'CPD-17614[c]'
'Vernolates[c]'
'2-Acylglycero-Phosphocholines[c]'
'NARINGIN[c]'
'NARINGENIN-7-O-BETA-D-GLUCOSIDE[c]'
'CPD-7075[c]'
'HYPOTAURINE[c]'
'CPD-7073[c]'
'FRU1P[c]'
'BCAA-dehydrogenase-lipoyl[c]'
'5-PHOSPHO-RIBOSYL-GLYCINEAMIDE[c]'
'5-P-RIBOSYL-N-FORMYLGLYCINEAMIDE[c]'
'CPD-14925[c]'
'CPD-10793[c]'
'3-P-HYDROXYPYRUVATE[c]'
'Hexanoyl-ACPs[c]'
'CPD-15364[c]'
'CPD-22044[c]'
'CPD-14268[c]'
'CPD-22045[c]'
'CPD-22048[c]'
'CPD-14271[c]'
'CPD-22050[c]'
'CPD-10283[c]'
'CPD-18491[c]'
'CPD-782[c]'
'Nucleoside-Diphosphates[c]'
'UDP-SULFOQUINOVOSE[c]'
'apo-Peptidyl-carrier-proteins[c]'
'ACET[e]'
'L-2-AMINOPENTANOIC-ACID[c]'
'ADENOSINE[e]'
'TREHALOSE[e]'
'CPD-15699[e]'
'LINOLENIC\_ACID[e]'
'ETR-Quinols[e]'
'ETF-Oxidized[e]'
'ANTHRANILATE[e]'
'CPD-7400[c]'
'CYANURIC-ACID[c]'
'GLUTAMYL-GLX-TRNAS[c]'
'CPD-700[c]'
'GLX-tRNAs[c]'
'CPD-1075[c]'
'CPD-7409[c]'
'Phytosphingosines[c]'
'Very-Long-Chain-Phytoceramides[c]'
'CPD-15362[c]'
'CPD-17399[c]'
'CPD-17400[c]'
'CPD-15368[c]'
'CPD-3736[c]'
'Ferrihemoglobins[c]'
'Ferrohemoglobins[c]'
'CPD-19144[c]'
'CPD-19170[c]'
'AMINOMETHYLDIHYDROLIPOYL-GCVH[c]'
'Deoxy-Ribonucleoside-Triphosphates[c]'
'DIHYDROLIPOYL-GCVH[c]'
'3-KETOLACTOSE[c]'
'CPD-17496[c]'
'CPD-1242[c]'
'P-NITROPHENOL[c]'
'CPD-9000[c]'
'CPD-14274[c]'
'CPD-14273[c]'
'CPD-13381[c]'
'CPD-14275[c]'
'CPD-14736[c]'
'CPD-19168[c]'
'ANTHRANILATE[c]'
'MAP-Kinase-L-Tyr[c]'
'MAP-Kinase-L-Phosphotyrosine[c]'
'CPD-14276[c]'
'3-HYDROXY-L-KYNURENINE[c]'
'CPD-14277[c]'
'CELLULOSE[e]'
'CPD-321[c]'
'CPD-14133[c]'
'CPD-4462[c]'
'CPD-22265[c]'
'1-Acyl-sn-glycerols[c]'
'CPD-19186[c]'
'Protein-S-methyl-L-cysteine[c]'
'a-thymine-in-DNA[c]'
'CPD0-2500[c]'
'Protein-Red-Disulfides[c]'
'L-methionyl-L-asparaginyl-Protein[c]'
'Protein-Ox-Disulfides[c]'
'L-methionyl-L-glutaminyl-Protein[c]'
'N-Ac-L-methionyl-L-aspartyl-Protein[c]'
'Alkyl-acetyl-glycero-phosphocholines[c]'
'1-Alkyl-sn-glycero-3-phosphocholines[c]'
'CPD-19167[c]'
'3-OXOPIMELOYL-COA[c]'
'CPD-459[c]'
'Phytoceramides[c]'
'Alpha-hydroxyphytoceramides[c]'
'CPD-15369[c]'
'CPD-15370[c]'
'HYDRPHENYLAC-CPD[c]'
'CPD-15366[c]'
'CPD-15373[c]'
'CPD-12601[c]'
'Guanine37-in-tRNAPhe[c]'
'tRNAPhe-Containing-N1-Methylguanine-37[c]'
'3-UREIDO-ISOBUTYRATE[c]'
'CPD-471[c]'
'CPD-209[c]'
'C3[c]'
'DIHYDRO-THYMINE[c]'
'CH3-MALONATE-S-ALD[c]'
'CHITIN[e]'
'CPD-13545[e]'
'Chitosan[e]'
'CHOLINE[e]'
'CARBON-DIOXIDE[e]'
'CPD-69[e]'
'CPD-355[e]'
'CPD-3617[e]'
'BETA-D-FRUCTOSE[e]'
'CPD-182[c]'
'CPD-14278[c]'
'CPD-181[c]'
'L-methionyl-L-aspartyl-Protein[c]'
'N-Ac-L-methionyl-L-glutamyl-Protein[c]'
'CPD3DJ-82[c]'
'L-methionyl-L-glutamyl-Protein[c]'
'L-methionyl-L-leucyl-Protein[c]'
'N-Ac-L-methionyl-L-leucyl-Protein[c]'
'Sphinga-4E-8E-dienine-Ceramides[c]'
'D-BETA-D-HEPTOSE-17-DIPHOSPHATE[c]'
'9-Methyl-sphing-4-8-dienine-ceramides[c]'
'L-methionyl-L-isoleucyl-Protein[c]'
'N-Ac-L-methionyl-L-isoleucyl-Protein[c]'
'L-methionyl-L-phenylalanyl-Protein[c]'
'ADP-D-GLYCERO-D-MANNO-HEPTOSE[c]'
'N-Ac-L-methionyl-L-phenylalanyl-Protein[c]'
'Delta5-Delta7-Steroids[c]'
'L-methionyl-L-tryptophanyl-Protein[c]'
'N-Ac-L-methionyl-L-tryptophanyl-Protein[c]'
'Delta7-Steroids[c]'
'L-methionyl-L-tyrosinyl-Protein[c]'
'CPD0-2474[c]'
'2-D-THREO-HYDROXY-3-CARBOXY-ISOCAPROATE[c]'
'NITRIC-OXIDE[c]'
'UNDECAPRENYL-DIPHOSPHATE[c]'
'Primary-Aliphatic-Amides[c]'
'CPD-19172[c]'
'N5-Formyl-THF-Glu-N[c]'
'CPD-19171[c]'
'2-AMINOMUCONATE\_SEMIALDEHYDE[c]'
'2-AMINO-MUCONATE[c]'
'CPD-19169[c]'
'CPD-444[c]'
'CPD-10172[c]'
'CPD-1061[c]'
'CPD-9451[c]'
'CPD-10175[c]'
'CPD-10175[e]'
'CPD-10171[c]'
'ASP-tRNAs[c]'
'CPD-10176[c]'
'Charged-ASP-tRNAs[c]'
'CPD-4205[c]'
'CPD-4586[c]'
'CPD-302[c]'
'CPD-17312[c]'
'CPD-21823[c]'
'TRANS-D2-ENOYL-COA[c]'
'CPD-21826[c]'
'Trans-3-enoyl-CoAs[c]'
'CPD-21825[c]'
'GAMMA-LINOLENOYL-COA[c]'
'CPD-21816[c]'
'CPD-21828[c]'
'CPD-21817[c]'
'CPD-21814[c]'
'Long-Chain-Fatty-Acids[c]'
'ARACHIDONYL-COA[c]'
'CPD-21830[c]'
'Sterols[c]'
'TAURINE[c]'
'Charged-CYS-tRNAs[c]'
'VANILLATE[c]'
'CPD-15192[c]'
'CPD-15216[c]'
'Sterol-3-beta-D-glucosides[c]'
'Long-chain-cholesterol-esters[c]'
'CPD-629[c]'
'CPD-556[c]'
'CPD-255[c]'
'CPD-17387[c]'
'CPD-17386[c]'
'CPD-17388[c]'
'CPD-14165[c]'
'CPD-14447[c]'
'4-FUMARYL-ACETOACETATE[c]'
'CPD-17355[c]'
'CPD-10244[c]'
'MONOMETHYL-ESTER-OF-TRANS-ACONITATE[c]'
'Behenoyl-ACPs[c]'
'3-oxo-lignoceroyl-ACPs[c]'
'R-3-hydroxylignoceroyl-ACPs[c]'
'trans-delta2-lignoceroyl-ACPs[c]'
'4-HYDROXYPHENYLACETATE[c]'
'NICOTINATE\_NUCLEOTIDE[c]'
'CPD-110[c]'
'VANILLIN[c]'
'CPD-581[c]'
'CPD-4587[c]'
'CPD-4588[c]'
'CPD-10177[c]'
'CPD-402[c]'
'N-METHYLANTHRANILOYL-COA[c]'
'Long-Chain-Steryl-Esters[c]'
'3-Phosphomonucleotides[c]'
'CPD-22005[c]'
'CPD-22004[c]'
'3-OCTAPRENYL-4-HYDROXYBENZOATE[c]'
'CPD-22006[c]'
'CPD-22003[c]'
'3-HYDROXYBENZOATE[c]'
'CPD-22008[c]'
'CPD-22009[c]'
'3-MERCAPTO-PYRUVATE[c]'
'IMP[c]'
'L-ASPARTATE-SEMIALDEHYDE[c]'
'L-BETA-ASPARTYL-P[c]'
'CPD-5881[c]'
'CPD-14202[c]'
'TETRADEHYDROACYL-COA[c]'
'2-hydroxyacyl-glutathiones[c]'
'2-Hydroxy-carboxylates[c]'
'CPD-8050[c]'
'CPD-14808[c]'
'CPD0-934[c]'
'CPD-15237[c]'
'Demethylmenaquinols[c]'
'Menaquinols[c]'
'DNA-containing-a-Apyrimidinic-Sites[c]'
'N-ACETYL-5-METHOXY-TRYPTAMINE[c]'
'5-HYDROXYISOURATE[c]'
'CPD-12014[c]'
'N-ACETYL-SEROTONIN[c]'
'CPD-45[c]'
'STIPIT-CPD[c]'
'STRICTOSIDINE[c]'
'2-Phenyloxirane[c]'
'PHENYLACETALDEHYDE[c]'
'CPD-8900[c]'
'CPD-17282[c]'
'Protein-L-lysine[c]'
'CPD-17381[c]'
'44-DIMETHYL-5ALPHA-CHOLEST-7-EN-3BET[c]'
'CPD-17392[c]'
'CPD-5846[c]'
'CPD1F-132[c]'
'CPD1F-95[c]'
'CPD-692[c]'
'DNA-with-Uracils[c]'
'CPD-787[c]'
'CPD-786[c]'
'PHOSPHORYL-ETHANOLAMINE[c]'
'CPD3DJ-11366[c]'
'SPHINGOSINE[c]'
'ISOCHORISMATE[c]'
'CHORISMATE[c]'
'CPD-22010[c]'
'CPD-22007[c]'
'CPD-7214[c]'
'CPD-7087[c]'
'3S-CITRYL-COA[c]'
'CPD-22012[c]'
'CPD-22013[c]'
'CPD-7221[c]'
'CPD-7222[c]'
'Retinols[c]'
'CPD-22014[c]'
'A-3-OXO-ACID[c]'
'CPD-10189[c]'
'CPD-10188[c]'
'B-KETOACYL-ACP[c]'
'OH-ACYL-ACP[c]'
'CPD-19163[c]'
'CARBAMYUL-L-ASPARTATE[c]'
'CPD-19159[c]'
'CPD-216[c]'
'Protein-L-serines[c]'
'Protein-D-serines[c]'
'CYSTINE[c]'
'THIOCYSTEINE[c]'
'CPD-15240[c]'
'Alpha-6-alpha-14-glucans[c]'
'Sulfur-Carrier-Proteins-ThiI[c]'
'Sulfurylated-ThiI[c]'
'CPD-578[c]'
'N-SUCCINYLLL-2-6-DIAMINOPIMELATE[c]'
'LL-DIAMINOPIMELATE[c]'
'CPD-12015[c]'
'CPD-12017[c]'
'CPD-17395[c]'
'BCAA-dehydrogenase-2MP-DH-lipoyl[c]'
'CPD-17390[c]'
'CPD-17389[c]'
'CPD-17391[c]'
'CPD-17393[c]'
'CPD-17394[c]'
'ISOPENICILLIN-N[c]'
'5-HYDROXY-TRYPTOPHAN[c]'
'SEROTONIN[c]'
'CPD-7224[c]'
'CPD-22011[c]'
'CAPSORUBIN[c]'
'CPD-22016[c]'
'CPD-22017[c]'
'CAPSANTHIN[c]'
'CPD-22018[c]'
'CPD-22015[c]'
'CPD-22021[c]'
'PENICILLIN-N[c]'
'D-SERINE[c]'
'S-3-HYDROXYBUTANOYL-COA[c]'
'CPD-650[c]'
'ADENOSYL-P4[c]'
'ADP-L-GLYCERO-D-MANNO-HEPTOSE[c]'
'DEOXYURIDINE[c]'
'CPD-19160[c]'
'Acetoacetyl-ACPs[c]'
'CPD-10269[c]'
'CPD-19162[c]'
'Beta-3-hydroxybutyryl-ACPs[c]'
'CPD-15244[c]'
'CPD-12016[c]'
'CPD-12259[c]'
'CPD-12230[c]'
'CPD-12018[c]'
'CPD-12019[c]'
'Aldehydes[c]'
'CPD-425[c]'
'Alkanesulfonates[c]'
'CPD-18346[c]'
'CPD-1107[c]'
'CPD-1772[c]'
'PROPANOL[c]'
'OH-PYR[c]'
'CPD-17396[c]'
'CPD-17397[c]'
'CPD-15365[c]'
'CPD-17401[c]'
'UDP-D-XYLOSE[c]'
'DI-H-URACIL[c]'
'CPD-22022[c]'
'CPD-22023[c]'
'CPD-22020[c]'
'CPD-289[c]'
'ILE-tRNAs[c]'
'Charged-ILE-tRNAs[c]'
'CPD-22026[c]'
'HOMO-I-CIT[c]'
'CPD-15717[c]'
'S-2-METHYLACYL-COA[c]'
'R-2-METHYLACYL-COA[c]'
'Glucuronosylated-Glucuronoside-Acceptors[c]'
'CPD-1063[c]'
'CPD-415[c]'
'Cis-delta-3-decenoyl-ACPs[c]'
'LC-ACYL-SN-GLYCEROL-3P[c]'
'CPD-18382[c]'
'Crotonyl-ACPs[c]'
'CPD-15254[c]'
'CPD-4822[c]'
'Saturated-Fatty-Acyl-ACPs[c]'
'CPD-15590[c]'
'CPD-15266[c]'
'CPD-18384[c]'
'CPD-15267[c]'
'RIBITOL[c]'
'CPD-19158[c]'
'CPD-19155[c]'
'CPD-19147[c]'
'N-METHYLTRYPTOPHAN[c]'
'CPD-12991[c]'
'CPD-90[c]'
'6-Acetyl-Beta-D-Galactosides[c]'
'CPD-17402[c]'
'CPD-17403[c]'
'ALPHA-D-GALACTOSE[c]'
'CPD-15367[c]'
'3-B-D-GALACTOSYL-SN-GLYCEROL[c]'
'D-Galactosyl-12-diacyl-glycerols[c]'
'2-DEHYDRO-3-DEOXY...

-------------------------------------------------------------------------------

The chemical element **P** is present in the empirical formula of **1543 metabolites**

'GLC-1-P[c]'
'ALPHA-GLC-6-P[c]'
'CO-A[c]'
'OLEOYL-COA[c]'
'CTP[c]'
'L-1-LYSOPHOSPHATIDATE[c]'
'CDP[c]'
'NADP[c]'
'NADPH[c]'
'CPD-12575[c]'
'ACETYL-COA[c]'
'GLUCOSAMINE-1P[c]'
'N-ACETYL-D-GLUCOSAMINE-1-P[c]'
'Phospholipids[c]'
'ATP[c]'
'Odd-Saturated-Fatty-Acyl-CoA[c]'
'AMP[c]'
'PPI[c]'
'ADP[c]'
'NAD[c]'
'NADH[c]'
'2E-5Z-tetradeca-2-5-dienoyl-ACPs[c]'
'PHOSPHORYL-CHOLINE[c]'
'5Z-tetradec-5-enoyl-ACPs[c]'
'CPD-541[c]'
'D-MYO-INOSITOL-1-MONOPHOSPHATE[c]'
'CPD-171[c]'
'DOLICHOLP[c]'
'MALONYL-ACP[c]'
'7Z-3-oxo-hexadec-7-enoyl-ACPs[c]'
'ACP[c]'
'CPD-8091[c]'
'CPD-8092[c]'
'CPD-2181[c]'
'LINOLENOYL-COA[c]'
'CPD-2182[c]'
'CPD-8088[c]'
'CPD-8093[c]'
'NADH-P-OR-NOP[c]'
'UDP-GLUCURONATE[c]'
'UDP[c]'
'CPD-208[c]'
'CPD-196[c]'
'Pi[c]'
'DEOXY-D-RIBOSE-1-PHOSPHATE[c]'
'DNA-deoxycytidine-thymidine-dimer[c]'
'DNA-Cytidines[c]'
'DNA-thymidines[c]'
'GAP[c]'
'DEOXY-RIBOSE-5P[c]'
'GLUTARYL-COA[c]'
'MALONYL-COA[c]'
'HEXANOYL-COA[c]'
'CPD-14687[c]'
'CPD-14615[c]'
'GLUTACONYL-COA[c]'
'CPD-18[c]'
'CPD-235[c]'
'NAD-P-OR-NOP[c]'
'56-Dihydrouracil17-in-tRNAs[c]'
'Uracil17-in-tRNAs[c]'
'56-Dihydrouracil20-in-tRNAs[c]'
'Uracil20-in-tRNAs[c]'
'Long-Chain-234-Saturated-acyl-CoAs[c]'
'56-Dihydrouracil47-in-tRNAs[c]'
'Uracil47-in-tRNAs[c]'
'Long-Chain-Trans-23-Dehydroacyl-CoA[c]'
'CPD-8089[c]'
'CPD-8090[c]'
'1-183-2-183-SN-GLYCEROL-PHOSPHOCHOLINE[c]'
'3R-7Z-3-hydroxy-hexadec-7-enoyl-ACPs[c]'
'2E-7Z-hexadeca-2-7-dienoyl-ACPs[c]'
'7Z-hexadec-7-enoyl-ACPs[c]'
'9Z-3-oxo-octadec-9-enoyl-ACPs[c]'
'CPD0-1158[c]'
'CPD0-1162[c]'
'CPD0-1163[c]'
'Orthophosphoric-Monoesters[c]'
'Ribonucleoside-Monophosphates[c]'
'PALMITYL-COA[c]'
'CPD-17621[c]'
'CROTONYL-COA[c]'
'R-4-PHOSPHOPANTOTHENOYL-L-CYSTEINE[c]'
'PANTETHEINE-P[c]'
'PHTYOSPHINGOSINE-1-P[c]'
'SN-GLYCEROL-1-PHOSPHATE[c]'
'GLYCEROL-3P[c]'
'DEPHOSPHO-COA[c]'
'PHOSPHORIBOSYL-CARBOXY-AMINOIMIDAZOLE[c]'
'P-RIBOSYL-4-SUCCCARB-AMINOIMIDAZOLE[c]'
'CPD-19179[c]'
'GTP[c]'
'Guanine37-in-tRNA[c]'
'tRNA-Containing-N1-Methylguanine-37[c]'
'TMP[c]'
'ADENOSINE5TRIPHOSPHO5ADENOSINE[c]'
'Guanine9-in-tRNA[c]'
'tRNA-Containing-N1-Methylguanine-9[c]'
'carbo-me-ur-34-tRNA[c]'
'5-2-me-oxy-2-oxo-et-ur-34-tRNA[c]'
'DPG[c]'
'PRECURSOR-Z[c]'
'23-DIPHOSPHOGLYCERATE[c]'
'CPD-4[c]'
'2-METHYL-3-HYDROXY-BUTYRYL-COA[c]'
'CPD-1083[c]'
'tRNA-Adenine-58[c]'
'tRNA-Containing-N1-MethylAdenine-58[c]'
'Adenine57-Adenine58-tRNAs[c]'
'N1-MeAdenine57-MeAdenine58-tRNAs[c]'
'CPD-8122[c]'
'3R-9Z-3-hydroxy-octadec-9-enoyl-ACPs[c]'
'2E-9Z-octadeca-2-9-dienoyl-ACPs[c]'
'CPD-12449[c]'
'PSEUDOURIDINE-5-P[c]'
'CPD-15317[c]'
'Long-Chain-Acyl-CoAs[c]'
'DIHYDROXY-BUTANONE-P[c]'
'DNA-Cytosines[c]'
'DNA-N4-Methylcytosine[c]'
'4-P-PANTOTHENATE[c]'
'R-3-Hydroxypalmitoyl-ACPs[c]'
'PAPS[c]'
'3-5-ADP[c]'
'3-oxo-palmitoyl-ACPs[c]'
'CMP[c]'
'D-6-P-GLUCONO-DELTA-LACTONE[c]'
'RIBOSE-5P[c]'
'DGDP[c]'
'Carboxyadenylated-MPT-synthases[c]'
'ITP[c]'
'PHOSPHO-ENOL-PYRUVATE[c]'
'IDP[c]'
'Poly-ADP-Riboses[c]'
'ADENOSINE\_DIPHOSPHATE\_RIBOSE[c]'
'Peptidoglycans[c]'
'NAcMur-Peptide-Undecaprenols[c]'
'CPD-8123[c]'
'Malonyl-acp-methyl-ester[c]'
'3-Ketoglutaryl-ACP-methyl-ester[c]'
'PROPIONYL-COA[c]'
'D-METHYL-MALONYL-COA[c]'
'FADH2[c]'
'FAD[c]'
'P-COUMAROYL-COA[c]'
'CAFFEOYL-COA[c]'
'BUTYRYL-COA[c]'
'TETRADECANOYL-COA[c]'
'CPD-10267[c]'
'STEAROYL-COA[c]'
'Oleoyl-ACPs[c]'
'ACETYL-ACP[c]'
'11Z-3-oxo-icos-11-enoyl-ACPs[c]'
'L-GLYCERALDEHYDE-3-PHOSPHATE[c]'
'Cytidine-34-tRNAmet[c]'
'Elongator-tRNAMet-acetylcytidine[c]'
'METHIONINE-SYNTHASE-METHYLCOBALAMIN[c]'
'Methionine-synthase-cob-II-alamins[c]'
'2-Hexadecenoyl-ACPs[c]'
'Palmitoyl-ACPs[c]'
'CPD-15896[c]'
'CPD-17487[c]'
'DGTP[c]'
'P3I[c]'
'CPD-8124[c]'
'CPD-8157[c]'
'CPD-8158[c]'
'CPD0-2244[c]'
'CPD0-2123[c]'
'D-glucopyranose-6-phosphate[c]'
'LAUROYLCOA-CPD[c]'
'LYS-tRNAs[c]'
'Charged-LYS-tRNAs[c]'
'4-HYDROXY-BUTYRYL-COA[c]'
'OH-CROTONYL-COA[c]'
'3-Hydroxyglutaryl-ACP-methyl-ester[c]'
'3-P-SERINE[c]'
'2-Lysophosphatidylcholines[c]'
'Enoylglutaryl-ACP-methyl-esters[c]'
'L-1-GLYCERO-PHOSPHORYLCHOLINE[c]'
'ACYL-COA[c]'
'3R-11Z-3-hydroxy-icos-11-enoyl-ACPs[c]'
'2E-11Z-icosa-2-11-dienoyl-ACPs[c]'
'11Z-icos-11-enoyl-ACPs[c]'
'CYS-tRNAs[c]'
'L-1-PHOSPHATIDYL-ETHANOLAMINE[c]'
'CPD-10260[c]'
'CPD-10261[c]'
'CPD-10262[c]'
'DIHYDRONEOPTERIN-P3[c]'
'Stearoyl-ACPs[c]'
'GERANYLGERANYL-PP[c]'
'LysW-L-glutamate-5-phosphate[c]'
'CPD-21340[c]'
'GERANYL-PP[c]'
'DELTA3-ISOPENTENYL-PP[c]'
'ALL-TRANS-HEXAPRENYL-DIPHOSPHATE[c]'
'CPD-15900[c]'
'3-HYDROXYPIMELYL-COA[c]'
'Cis-Delta5-dodecenoyl-ACPs[c]'
'Charged-GLT-tRNAs[c]'
'GLT-tRNAs[c]'
'ARABINOSE-5P[c]'
'RIBULOSE-5P[c]'
'Saturated-Fatty-Acyl-CoA[c]'
'G3P[c]'
'DCDP[c]'
'DCTP[c]'
'CPD-12335[c]'
'CPD-12336[c]'
'CPD-12334[c]'
'E-11-TETRADECENOYL-COA[c]'
'CPD-17814[c]'
'CPD-17813[c]'
'CPD-17815[c]'
'3-OXOPALMITOYL-COA[c]'
'CPD-17464[c]'
'tRNA-Containing-N2-Dimethylgua-26-Gua27[c]'
'tRNA-Containing-N2-Methylgua-26-Gua27[c]'
'tRNA-Containing-N2-Dimetgua-26-MeGua27[c]'
'tRNA-Containing-N2-DiMeGua-26-DiMeGua27[c]'
'Guanine26-Guanine27-in-tRNAs[c]'
'Dodecanoyl-ACPs[c]'
'3-oxo-myristoyl-ACPs[c]'
'CPD-15684[c]'
'CPD-15685[c]'
'CPD-15686[c]'
'CPD-15687[c]'
'CPD-15688[c]'
'CPD-15689[c]'
'CPD-15690[c]'
'CPD-15692[c]'
'CPD-15691[c]'
'ACETOACETYL-COA[c]'
'INOSITOL-1-4-5-TRISPHOSPHATE[c]'
'INOSITOL-1-4-BISPHOSPHATE[c]'
'D-MYO-INOSITOL-4-PHOSPHATE[c]'
'PHOSPHATIDYLINOSITOL-345-TRIPHOSPHATE[c]'
'PHOSPHATIDYL-MYO-INOSITOL-45-BISPHOSPHA[c]'
'ALPHA-L-GLUTAMYL-PHOSPHATE[c]'
'BENZOYLCOA[c]'
'DCMP[c]'
'DUMP[c]'
'CPD-465[c]'
'GDP-MANNOSE[c]'
'GDP-L-GALACTOSE[c]'
'Polyisoprenyl-Diphosphates[c]'
'DTDP-DEOH-DEOXY-GLUCOSE[c]'
'CPD-13952[c]'
'CPD-17870[c]'
'CPD-17877[c]'
'XANTHOSINE-5-PHOSPHATE[c]'
'PRPP[c]'
'FMNH2[c]'
'FMN[c]'
'D-ALPHABETA-D-HEPTOSE-7-PHOSPHATE[c]'
'T2-DECENOYL-COA[c]'
'CPD-12777[c]'
'MANNOSE-6P[c]'
'CPD-15711[c]'
'CPD-1241[c]'
'CPD-15712[c]'
'DUTP[c]'
'OROTIDINE-5-PHOSPHATE[c]'
'2-DEOXY-D-GLUCOSE-6-PHOSPHATE[c]'
'D-BETA-D-HEPTOSE-1-P[c]'
'PYRIDOXAL\_PHOSPHATE[c]'
'CPD-9001[c]'
'CPD-8999[c]'
'2-METHYL-BUTYRYL-COA[c]'
'CPD-17882[c]'
'LEU-tRNAs[c]'
'Charged-LEU-tRNAs[c]'
'Sphingoid-1-phosphates[c]'
'R-3-hydroxymyristoyl-ACPs[c]'
'RIBOSE-1P[c]'
'VERY-LONG-CHAIN-FATTY-ACYL-COA[c]'
'3-phosphooligonucleotides[c]'
'3-Prime-Nucleoside-Monophosphates[c]'
'Oligonucleotides[c]'
'Nucleoside-Monophosphates[c]'
'L-1-phosphatidyl-inositols[c]'
'CPD-1121[c]'
'DIHYDROXY-ACETONE-PHOSPHATE[c]'
'UMP[c]'
'PHOSPHORIBULOSYL-FORMIMINO-AICAR-P[c]'
'D-ERYTHRO-IMIDAZOLE-GLYCEROL-P[c]'
'CPD-8087[c]'
'CPD-8086[c]'
'CPD0-1905[c]'
'CPD-12365[c]'
'XYLULOSE-5-PHOSPHATE[c]'
'CPD-8347[c]'
'PHOSPHATIDYLCHOLINE[c]'
'Tetradec-2-enoyl-ACPs[c]'
'DAMP[c]'
'DADP[c]'
'2-KETO-3-DEOXY-6-P-GLUCONATE[c]'
'AICAR[c]'
'Charged-THR-tRNAs[c]'
'Cyclic-3-5-Nucleoside-Monophosphates[c]'
'Cyclic-2-3-Ribonucleoside-Monophosphates[c]'
'2-Prime-Ribonucleoside-Monophosphates[c]'
'GLN-tRNAs[c]'
'3Z-dodec-3-enoyl-ACPs[c]'
'OXALYL-COA[c]'
'CPD-1162[c]'
'GLC-6-P[c]'
'CPD-1181[c]'
'3R-5Z-3-hydroxy-tetradec-5-enoyl-ACPs[c]'
'5Z-3-oxo-tetradec-5-enoyl-ACPs[c]'
'CPD-448[c]'
'N-ACETYL-GLUTAMYL-P[c]'
'CPD-10254[c]'
'SUC-COA[c]'
'3-KETO-ADIPYL-COA[c]'
'ACYL-ACP[c]'
'ACYL-SN-GLYCEROL-3P[c]'
'Protein-Phosphothreonines[c]'
'DGMP[c]'
'CARBAMOYL-P[c]'
'Charged-GLN-tRNAs[c]'
'O-PHOSPHO-L-HOMOSERINE[c]'
'Myristoyl-ACPs[c]'
'D-SEDOHEPTULOSE-7-P[c]'
'CPD-15567[c]'
'CPD-15568[c]'
'CPD-3707[c]'
'CPD-13025[c]'
'NADH-P-OR-NOP[e]'
'NADP[e]'
'NADPH[e]'
'CPD-14553[c]'
'CPD0-935[c]'
'CPD0-936[c]'
'CPD0-937[c]'
'CPD0-938[c]'
'CPD-17732[c]'
'CPD-17733[c]'
'CPD-17741[c]'
'Decanoyl-ACPs[c]'
'DNA-with-3-prime-pp-5-prime-G-cap[c]'
'3-Prime-Phosphate-Terminated-DNAs[c]'
'GMP[c]'
'3-oxo-dodecanoyl-ACPs[c]'
'DNA-Ligase-L-lysine-adenylate[c]'
'A-5-prime-PP-5-prime-DNA[c]'
'CPD-13469[c]'
'FRUCTOSE-6P[c]'
'CPD-15666[c]'
'CPD-10809[c]'
'DIAMINO-OH-PHOSPHORIBOSYLAMINO-PYR[c]'
'CPD-1086[c]'
'Lignoceroyl-ACPs[c]'
'3-oxo-cerotoyl-ACPs[c]'
'CPD-17743[c]'
'CPD-17744[c]'
'CPD-17746[c]'
'CPD-17750[c]'
'FARNESYL-PP[c]'
'CPD0-1028[c]'
'CPD-201[c]'
'CPD-202[c]'
'DIACYLGLYCEROL-PYROPHOSPHATE[c]'
'L-PHOSPHATIDATE[c]'
'DNA-N[c]'
'3-Hydroxy-Terminated-DNAs[c]'
'Deacetylated-Peptidoglycan[c]'
'NICOTINAMIDE\_NUCLEOTIDE[c]'
'DNA-Ligase-L-lysine-guanylate[c]'
'tRNA-precursors[c]'
'SS-Oligoribonucleotides[c]'
'5-Phospho-terminated-DNAs[c]'
'Pi[e]'
'D-GLUCOSAMINE-6-P[c]'
'N-ACETYL-D-GLUCOSAMINE-6-P[c]'
'R-3-hydroxydodecanoyl-ACPs[c]'
'THZ-P[c]'
'AMINO-HYDROXYMETHYL-METHYLPYRIMIDINE-PP[c]'
'THIAMINE-P[c]'
'THIAMINE-PYROPHOSPHATE[c]'
'CPD-611[c]'
'2-CARBOXY-D-ARABINITOL-1-PHOSPHATASE[c]'
'ACETYL-P[c]'
'CPD-15637[c]'
'CPD-15653[c]'
'CPD-15668[c]'
'CPD-15667[c]'
'CPD-15654[c]'
'CPD-15655[c]'
'R-3-hydroxycerotoyl-ACPs[c]'
'Trans-D2-hexacos-2-enoyl-ACPs[c]'
'Cerotoyl-ACPs[c]'
'CPD-14392[c]'
'Sphingomyelins[e]'
'CPD-14018[c]'
'FERULOYL-COA[c]'
'CPD-501[c]'
'UTP[c]'
'UDP-D-GALACTURONATE[c]'
'CPD-12231[c]'
'CPD-12261[c]'
'RNA-DNA-hybrids[c]'
'DNA-Holder[c]'
'CPD-2961[c]'
'RNA-Containing-Guanosine[c]'
'RNA-3prime-Guanosine-3prime-P[c]'
'5Prime-OH-Terminated-RNAs[c]'
'G-5-prime-PP-5-prime-DNA[c]'
'3-KETOACYL-COA[c]'
'L-3-HYDROXYACYL-COA[c]'
'RNA-Ligase-L-lysine-adenylate[c]'
'5-Phospho-RNA[c]'
'A-5-prime-PP-5-prime-RNA[c]'
'RNA-Holder[c]'
'3Prime-OH-Terminated-RNAs[c]'
'DATP[c]'
'ERYTHROSE-4P[c]'
'3-DEOXY-D-ARABINO-HEPTULOSONATE-7-P[c]'
'ALPHA-GLUCOSE-16-BISPHOSPHATE[c]'
'Dodec-2-enoyl-ACPs[c]'
'CPD-13575[c]'
'CPD-15895[c]'
'3-Prime-Phosphate-Terminated-RNAs[c]'
'CPD-14407[c]'
'CPD0-2350[c]'
'Pre-tRNA-5-prime-half-molecules[c]'
'Pre-tRNA-3-prime-half-molecules[c]'
'CPD-17794[c]'
'CPD-14422[c]'
'CPD-14423[c]'
'CPD-14424[c]'
'Protein-Ser-or-Thr-phosphate[c]'
'CPD-14425[c]'
'CPD-14426[c]'
'CPD-13328[c]'
'CPD-15656[c]'
'CPD-15657[c]'
'CPD-15675[c]'
'CPD-15651[c]'
'CPD-15652[c]'
'CPD-15677[c]'
'CPD-15676[c]'
'AMINO-HYDROXYMETHYL-METHYL-PYR-P[c]'
'DNA-3-methyladenines[c]'
'DNA-containing-aPurinic-Sites[c]'
'RNA-with-3-prime-pp-5-prime-A-cap[c]'
'Cyclic-Phosphate-Terminated-RNAs[c]'
'RNA-3-prime-P-cyclase-L-His-adenylate[c]'
'CPD-5164[c]'
'DEOXYXYLULOSE-5P[c]'
'CPD-17883[c]'
'CPD-5165[c]'
'CPD-5166[c]'
'CPD-5167[c]'
'CPD-12303[c]'
'UDP-N-ACETYL-D-GLUCOSAMINE[c]'
'CPD-12304[c]'
'CPD-17802[c]'
'CPD-12258[c]'
'CPD-9646[c]'
'CPD-12311[c]'
'CPD-12310[c]'
'Z-11-TETRADECENOYL-COA[c]'
'CPD-5168[c]'
'Uridine44-in-tRNA-Ser[c]'
'2-O-Methyluridine44-tRNASer[c]'
'Guanine10-in-tRNA[c]'
'tRNA-Containing-N2-Methylguanine-10[c]'
'Guanine26-in-tRNA[c]'
'tRNA-Containing-N2-Methylguanine-26[c]'
'tRNA-Containing-N2-dimethylguanine-26[c]'
'CPD-17858[c]'
'CPD-17894[c]'
'GDP[c]'
'CPD-15661[c]'
'CPD-10832[c]'
'CPD-15662[c]'
'CPD-15678[c]'
'CPD-15663[c]'
'CPD-9407[c]'
'CPD-15658[c]'
'CPD0-1308[c]'
'CPD0-1074[c]'
'Short-Chain-Trans-23-Dehydroacyl-CoA[c]'
'Short-Chain-234-Saturated-acyl-CoAs[c]'
'Very-Long-Chain-Trans-23-Dehydroacyl-CoA[c]'
'Very-long-Chain-234-Saturated-acyl-CoAs[c]'
'VAL-tRNAs[c]'
'Charged-VAL-tRNAs[c]'
'Pyruvate-Dehydrogenase-Phosphoserine[c]'
'CPD-535[c]'
'FRUCTOSE-2-PHOSPHATE[c]'
'3-Hydroxy-octanoyl-ACPs[c]'
'3-Oxo-octanoyl-ACPs[c]'
'tRNAPhe-Containing-4-demethylwyosine-37[c]'
'yW-86[c]'
'Octadec-2-enoyl-ACPs[c]'
'yW-58[c]'
'tRNAPhe-wybutosine[c]'
'yW-72[c]'
'OHyWstar-tRNA[c]'
'OHyW-58-tRNAPhe[c]'
'GDP-4-DEHYDRO-6-DEOXY-D-MANNOSE[c]'
'CPD-14280[c]'
'CPD-10279[c]'
'CPD-14281[c]'
'CPD-10280[c]'
'CPD-14282[c]'
'NMNH[c]'
'CPD0-881[c]'
'CPD1G-277[c]'
'CPD-14283[c]'
'CPDQT-520[c]'
'CPD-2183[c]'
'CPD-14300[c]'
'CPD-9406[c]'
'CPD-17635[c]'
'FMN[e]'
'OHyW-tRNAPhe[c]'
'25S-rRNA-adenine-2142[c]'
'25S-rRNA-N1-methyladenine-2142[c]'
'25S-rRNA-adenine-645[c]'
'25S-rRNA-N1-methyladenine-645[c]'
'2-Octenoyl-ACPs[c]'
'L-aspartyl-tRNAAsn[c]'
'Octanoyl-ACPs[c]'
'Charged-ASN-tRNAs[c]'
'L-glutamyl-tRNAGln[c]'
'5-HYDROXY-FERULOYL-COA[c]'
'CPD-12180[c]'
'SINAPOYL-COA[c]'
'18S-rRNA-pseudouridine-1191[c]'
'18S-rRNA-N1-methylpseudouridine-1191[c]'
'IPC[c]'
'VLC-MIPC[c]'
'CPD-1108[c]'
'Single-Stranded-DNAs[c]'
'Ribonucleoside-Triphosphates[c]'
'ssDNA-RNA-primer-hybrid[c]'
'APS[c]'
'CAMP[c]'
'CPD-7652[c]'
'DIHYDROPTERIN-CH2OH-PP[c]'
'CPD-10766[c]'
'Nucleoside-Triphosphates[c]'
'FRUCTOSE-16-DIPHOSPHATE[c]'
'CPD-17370[c]'
'CPD-15435[c]'
'N6-L-threonylcarbamoyladenine37-tRNAs[c]'
'tRNA-adenine-37[c]'
'CPD-15436[c]'
'Butanoyl-ACPs[c]'
'3-oxo-decanoyl-ACPs[c]'
'CPD-10794[c]'
'3-HYDROXYADIPYL-COA[c]'
'TRANS-23-DEHYDROADIPYL-COA[c]'
'1-PHOSPHATIDYL-1D-MYO-INOSITOL-34-BISPH[c]'
'2-PG[c]'
'CPD-452[c]'
'CPD-1113[c]'
'CPD-160[c]'
'CPD-12199[c]'
'CPD-17641[c]'
'PHOSPHORIBOSYL-ATP[c]'
'CPD-17638[c]'
'TAGATOSE-1-6-DIPHOSPHATE[c]'
'Beta-hydroxydecanoyl-ACPs[c]'
'CPD-10600[c]'
'Charged-ARG-tRNAs[c]'
'CPD-17624[c]'
'Phosphoacetylglucosamine-Mutase-P[c]'
'N-ACETYL-D-GLUCOSAMINE-16-BIS-P[c]'
'CPD-15566[c]'
'TAGATOSE-6-PHOSPHATE[c]'
'CPD-558[c]'
'CDPDIACYLGLYCEROL[c]'
'L-1-PHOSPHATIDYL-GLYCEROL[c]'
'CARDIOLIPIN[c]'
'Deoxy-Ribonucleoside-Monophosphates[c]'
'ARG-tRNAs[c]'
'TTP[c]'
'L-RIBULOSE-5-P[c]'
'Trans-D2-decenoyl-ACPs[c]'
'CPD-6972[c]'
'CPD-22025[c]'
'CPD-10556[c]'
'Ultra-Long-Chain-Acyl-CoAs[c]'
'ISOVALERYL-COA[c]'
'CPD-18831[c]'
'CPD-18832[c]'
'CPD-4211[c]'
'ENT-COPALYL-DIPHOSPHATE[c]'
'CPD-602[c]'
'CPD-19154[c]'
'CPD-19161[c]'
'CPD-19157[c]'
'CPD-19148[c]'
'3-oxo-hexanoyl-ACPs[c]'
'R-3-hydroxyhexanoyl-ACPs[c]'
'GALACTOSE-1P[c]'
'L-methionyl-tRNAfmet[c]'
'CPD-17434[c]'
'CPD-9965[c]'
'Deoxy-Ribonucleoside-Diphosphates[c]'
'Ribonucleoside-Diphosphates[c]'
'UDP-D-GALACTO-14-FURANOSE[c]'
'Hex-2-enoyl-ACPs[c]'
'CPD-19150[c]'
'CPD-19151[c]'
'CPD-19153[c]'
'Kanamycin-3-phosphates[c]'
'CPD-22039[c]'
'CPD-14293[c]'
'CPD-22040[c]'
'D-3-HYDROXYACYL-COA[c]'
'CPD-22041[c]'
'CPD-14269[c]'
'CPD-22043[c]'
'CPD-15363[c]'
'3-oxo-stearoyl-ACPs[c]'
'CPD-15978[c]'
'CPD-15361[c]'
'ACP[e]'
'2-Acylglycero-Phosphocholines[c]'
'FRU1P[c]'
'5-PHOSPHO-RIBOSYL-GLYCINEAMIDE[c]'
'5-P-RIBOSYL-N-FORMYLGLYCINEAMIDE[c]'
'CPD-14925[c]'
'CPD-10793[c]'
'3-P-HYDROXYPYRUVATE[c]'
'Hexanoyl-ACPs[c]'
'CPD-15364[c]'
'CPD-22044[c]'
'CPD-22045[c]'
'CPD-22048[c]'
'CPD-14271[c]'
'CPD-22050[c]'
'CPD-10283[c]'
'CPD-18491[c]'
'Nucleoside-Diphosphates[c]'
'UDP-SULFOQUINOVOSE[c]'
'GLUTAMYL-GLX-TRNAS[c]'
'GLX-tRNAs[c]'
'CPD-15362[c]'
'CPD-15368[c]'
'CPD-19144[c]'
'CPD-19170[c]'
'Deoxy-Ribonucleoside-Triphosphates[c]'
'CPD-14274[c]'
'CPD-14273[c]'
'CPD-13381[c]'
'CPD-14275[c]'
'CPD-19168[c]'
'MAP-Kinase-L-Phosphotyrosine[c]'
'CPD-14276[c]'
'CPD-14277[c]'
'CPD-14133[c]'
'CPD-19186[c]'
'a-thymine-in-DNA[c]'
'Alkyl-acetyl-glycero-phosphocholines[c]'
'1-Alkyl-sn-glycero-3-phosphocholines[c]'
'CPD-19167[c]'
'3-OXOPIMELOYL-COA[c]'
'CPD-15369[c]'
'CPD-15370[c]'
'CPD-15366[c]'
'Guanine37-in-tRNAPhe[c]'
'tRNAPhe-Containing-N1-Methylguanine-37[c]'
'CPD-209[c]'
'C3[c]'
'CPD-14278[c]'
'D-BETA-D-HEPTOSE-17-DIPHOSPHATE[c]'
'ADP-D-GLYCERO-D-MANNO-HEPTOSE[c]'
'CPD0-2474[c]'
'UNDECAPRENYL-DIPHOSPHATE[c]'
'CPD-19172[c]'
'CPD-19171[c]'
'CPD-19169[c]'
'CPD-444[c]'
'ASP-tRNAs[c]'
'Charged-ASP-tRNAs[c]'
'CPD-4205[c]'
'CPD-17312[c]'
'TRANS-D2-ENOYL-COA[c]'
'Trans-3-enoyl-CoAs[c]'
'GAMMA-LINOLENOYL-COA[c]'
'CPD-21828[c]'
'ARACHIDONYL-COA[c]'
'Charged-CYS-tRNAs[c]'
'CPD-17387[c]'
'CPD-17386[c]'
'CPD-17388[c]'
'Behenoyl-ACPs[c]'
'3-oxo-lignoceroyl-ACPs[c]'
'R-3-hydroxylignoceroyl-ACPs[c]'
'trans-delta2-lignoceroyl-ACPs[c]'
'NICOTINATE\_NUCLEOTIDE[c]'
'N-METHYLANTHRANILOYL-COA[c]'
'3-Phosphomonucleotides[c]'
'CPD-22005[c]'
'CPD-22004[c]'
'CPD-22006[c]'
'CPD-22003[c]'
'CPD-22008[c]'
'CPD-22009[c]'
'IMP[c]'
'L-BETA-ASPARTYL-P[c]'
'TETRADEHYDROACYL-COA[c]'
'CPD0-934[c]'
'CPD-15237[c]'
'DNA-containing-a-Apyrimidinic-Sites[c]'
'DNA-with-Uracils[c]'
'PHOSPHORYL-ETHANOLAMINE[c]'
'CPD3DJ-11366[c]'
'CPD-22010[c]'
'CPD-22007[c]'
'3S-CITRYL-COA[c]'
'CPD-22012[c]'
'CPD-22013[c]'
'CPD-7221[c]'
'CPD-7222[c]'
'CPD-22014[c]'
'B-KETOACYL-ACP[c]'
'OH-ACYL-ACP[c]'
'CPD-19163[c]'
'CPD-19159[c]'
'CPD-22011[c]'
'CPD-22016[c]'
'CPD-22017[c]'
'CPD-22018[c]'
'CPD-22015[c]'
'CPD-22021[c]'
'S-3-HYDROXYBUTANOYL-COA[c]'
'CPD-650[c]'
'ADENOSYL-P4[c]'
'ADP-L-GLYCERO-D-MANNO-HEPTOSE[c]'
'CPD-19160[c]'
'Acetoacetyl-ACPs[c]'
'CPD-10269[c]'
'CPD-19162[c]'
'Beta-3-hydroxybutyryl-ACPs[c]'
'CPD-15244[c]'
'CPD-12259[c]'
'CPD-12230[c]'
'CPD-18346[c]'
'CPD-1107[c]'
'CPD-15365[c]'
'CPD-17401[c]'
'UDP-D-XYLOSE[c]'
'CPD-22022[c]'
'CPD-22023[c]'
'CPD-22020[c]'
'ILE-tRNAs[c]'
'Charged-ILE-tRNAs[c]'
'S-2-METHYLACYL-COA[c]'
'R-2-METHYLACYL-COA[c]'
'CPD-1063[c]'
'CPD-415[c]'
'Cis-delta-3-decenoyl-ACPs[c]'
'LC-ACYL-SN-GLYCEROL-3P[c]'
'Crotonyl-ACPs[c]'
'Saturated-Fatty-Acyl-ACPs[c]'
'CPD-19158[c]'
'CPD-19155[c]'
'CPD-19147[c]'
'CPD-17402[c]'
'CPD-17403[c]'
'CPD-15367[c]'
'Initiation-tRNAmet[c]'
'N-ACETYL-D-MANNOSAMINE-6P[c]'
'3-oxo-arachidoyl-ACPs[c]'
'Arachidoyl-ACPs[c]'
'trans-delta2-arachidoyl-ACPs[c]'
'cis-cis-19-31-dicyclopropyl-C52-ACPs[c]'
'LIPOYL-AMP[c]'
'CPD1G-124[c]'
'PARATHION[c]'
'DIETHYLTHIOPHOSPHATE[c]'
'Aryl-Dialkyl-Phosphate[c]'
'Dialkyl-phosphates[c]'
'CPD-4750[c]'
'1-Alkyl-2-acyl-glycerol-3-phosphate[c]'
'1-Alkyl-sn-glycerol-3-phosphates[c]'
'CDP-ETHANOLAMINE[c]'
'1-Alkyl-2-acyl-glycerol-P-Etn[c]'
'CPD-514[c]'
'CPD0-2171[c]'
'CPD0-2105[c]'
'4-Phosphooxy-L-aspartyl-tRNAAsn[c]'
'CPD-14916[c]'
'CPD0-2106[c]'
'5-Phosphooxy-L-glutamyl-tRNAGlln[c]'
'CPD0-2108[c]'
'5-PHOSPHORIBOSYL-N-FORMYLGLYCINEAMIDINE[c]'
'Reduced-flavodoxins[c]'
'Oxidized-flavodoxins[c]'
'CPD-11700[c]'
'CPD-11937[c]'
'CPD-11939[c]'
'MI-HEXAKISPHOSPHATE[c]'
'D-SORBITOL-6-P[c]'
'CPD-15709[c]'
'DITP[c]'
'DIMP[c]'
'XTP[c]'
'5-BETA-L-THREO-PENTAPYRANOSYL-4-ULOSE-[c]'
'CPD-867[c]'
'CPD-868[c]'
'TRANS-D2-ENOYL-ACP[c]'
'DNA-containing-abasic-Sites[c]'
'3-terminal-unsaturated-sugars[c]'
'CPD-17346[c]'
'CDP-CHOLINE[c]'
'Plasmanylcholine[c]'
'CPD-17347[c]'
'Alkyl-enyl-acyl-gly-P-EtOH-amines[c]'
'CPD-17348[c]'
'CPD-12646[c]'
'1-Alkenylglycerophosphoethanolamines[c]'
'cis-19-CP-37-Mex-38-Me-C59-ACPs[c]'
'CPD1G-204[c]'
'CPD0-2121[c]'
'UDP-4-AMINO-4-DEOXY-L-ARABINOSE[c]'
'CPD1G-332[c]'
'R-3-hydroxy-cis-vaccenoyl-ACPs[c]'
'5-DIPHOSPHO-1D-MYO-INOSITOL-12346P[c]'
'CPD-6681[c]'
'CPD-505[c]'
'ACRYLYL-COA[c]'
'CPD-20[c]'
'CPD-14795[c]'
'DEAMIDO-NAD[c]'
'CPD-17324[c]'
'CPD-17367[c]'
'CPD-17368[c]'
'CPD-17323[c]'
'CPD-71[c]'
'CPD-7275[c]'
'CPD-17372[c]'
'PLASMENYLCHOLINE[c]'
'CPD-563[c]'
'3-Oxoacyl-CoAs[c]'
'2-HYDROXY-3-KETO-5-METHYLTHIO-1-PHOSPHOP[c]'
'CPD-649[c]'
'holo-VibB[c]'
'CARBOXYPHENYLAMINO-DEOXYRIBULOSE-P[c]'
'INDOLE-3-GLYCEROL-P[c]'
'b-Keto-cis-D5-dodecenoyl-ACPs[c]'
'CPD-17371[c]'
'CPD-17373[c]'
'UDP-OHMYR-ACETYLGLUCOSAMINE[c]'
'OCTAPRENYL-DIPHOSPHATE[c]'
'CPD-12173[c]'
'1-Alkenyl-phosphoglycerol[c]'
'ISOBUTYRYL-COA[c]'
'IMIDAZOLE-ACETOL-P[c]'
'CPD-11975[c]'
'Sphingomyelins[c]'
'b-Hydroxy-cis-D5-dodecenoyl-ACPs[c]'
'Trans-D3-cis-D5-dodecenoyl-ACPs[c]'
'CPD-17382[c]'
'CPD-17332[c]'
'CPD-17383[c]'
'tRNA-Sec[c]'
'2-Acyl-sn-glycerol-3-phosphates[c]'
'cis-cis-D19-31-C50-2-ACPs[c]'
'UDP-N-ACETYLMURAMATE[c]'
'UDP-ACETYL-CARBOXYVINYL-GLUCOSAMINE[c]'
'3-oxo-behenoyl-ACPs[c]'
'DUDP[c]'
'R-3-hydroxybehenoyl-ACPs[c]'
'ASN-tRNAs[c]'
'CPD-645[c]'
'PHOSPHORIBOSYL-FORMAMIDO-CARBOXAMIDE[c]'
'2-trans-4-cis-dienoyl-CoAs[c]'
'PENTANOYLCOA-CPD[c]'
'CPD-13851[c]'
'CPD-15199[c]'
'CPD-9444[c]'
'CPD-11984[c]'
'trans-delta2-behenoyl-ACPs[c]'
'CPD-11997[c]'
'CPD-11998[c]'
'L-seryl-SEC-tRNAs[c]'
'CPD-17331[c]'
'CPD-17385[c]'
'3-METHYL-CROTONYL-COA[c]'
'CPD-14394[c]'
'CPD0-2231[c]'
'Very-Long-Chain-oxoacyl-CoAs[c]'
'Very-Long-Chain-3-Hydroxyacyl-CoAs[c]'
'CPD-21527[c]'
'3-HYDROXY-3-4-METHYLPENT-3-EN-1-YLG-COA[c]'
'CPD-12897[c]'
'RNA-3-Guanosine-23-Cyclophosphate[c]'
'CPD-7243[c]'
'SER-tRNAs[c]'
'Charged-SER-tRNAs[c]'
'CPD-6701[c]'
'CPD-14405[c]'
'CPD-14406[c]'
'CPD1G-772[c]'
'CPD1G-773[c]'
'5-P-BETA-D-RIBOSYL-AMINE[c]'
'CPD1G-774[c]'
'CPD-17313[c]'
'CPD0-2113[c]'
'Medium-Chain-Acyl-CoAs[c]'
'Medium-Chain-234-Saturated-acyl-CoAs[c]'
'dihomogammalinolenoyl-acp[c]'
'MANNOSE-1P[c]'
'CPD-14419[c]'
'CPD-534[c]'
'CPD-14420[c]'
'CPD-12647[c]'
'CPD-6702[c]'
'1-PHOSPHATIDYL-1D-MYO-INOSITOL-35-BISPH[c]'
'1-PHOSPHATIDYL-1D-MYO-INOSITOL-5-PHOSPHA[c]'
'INOSITOL-1-3-4-TRIPHOSPHATE[c]'
'D-MYO-INOSITOL-13-BISPHOSPHATE[c]'
'CPD-206[c]'
'2-HYDROXYPHYTANOYL-COA[c]'
'cis-cis-D19-37-C56-2-ACPs[c]'
'cis-D19-37-OH-38-Me-C57-1-ACPs[c]'
'1-Stearoyl-L-Phosphatidate[c]'
'N-5-PHOSPHORIBOSYL-ANTHRANILATE[c]'
'1-gamma-Linolenoyl-L-Phosphatidate[c]'
'1-stearidonoyl-L-Phosphatidate[c]'
'1-Alpha-Linolenoyl-L-Phosphatidate[c]'
'Phosphoserines[c]'
'1-Linoleoyl-L-Phosphatidate[c]'
'pppGp-his-tRNAs[c]'
'pGp-his-tRNAs[c]'
'CPD-9999[c]'
'L-PHOSPHINOTHRICIN[c]'
'DIPHOSPHO-1D-MYO-INOSITOL-TETRAKISPHOSPH[c]'
'CPD-11938[c]'
'3-HYDROXY-DOCOSAPENTAENOYL-ACP[c]'
'3-OXO-EICOSAPENTAENOYL-ACP[c]'
'3-HYDROXY-3-METHYL-GLUTARYL-COA[c]'
'cis-D19-37-MOH-38-Me-C57-1-ACPs[c]'
'R-3-hydroxyarachidoyl-ACPs[c]'
'trans-D18-37-OH-38-Me-C58-1-ACPs[c]'
'trans-D18-37-MOH-38-Me-C58-1-ACPs[c]'
'trans-18-CP-37-Mex-38-Me-C60-ACPs[c]'
'3OH-4P-OH-ALPHA-KETOBUTYRATE[c]'
'4-PHOSPHONOOXY-THREONINE[c]'
'CPD0-1423[c]'
'3-HYDROXY-ISOVALERYL-COA[c]'
'CPD-17319[c]'
'CPD-10284[c]'
'CPD-13691[c]'
'CPD-19066[c]'
'CINNAMOYL-COA[c]'
'tRNA-uridines[c]'
'tRNA-Dihydrouridines[c]'
'tRNA-with-7-aminomethyl-7-deazaguanine[c]'
'Guanine34-in-tRNAs[c]'
'CPD-11561[c]'
'CPD-11932[c]'
'DOCOSAPENTAENOYL-ACP[c]'
'cis-cis-D21-39-C58-2-ACPs[c]'
'cis-D21-39-OH-40-Me-C59-1-ACPs[c]'
'cis-D21-39-oxo-40-Me-C59-1-ACPs[c]'
'cis-21-CP-39-keto-40-Me-C60-ACPs[c]'
'trans-D20-39-oxo-40-Me-C60-1-ACPs[c]'
'trans-20-CP-22-Me-39-keto-40-Me-C61-ACPs[c]'
'CPD-653[c]'
'CPD0-2232[c]'
'1-ALKYL-GLYCERONE-3-PHOSPHATE[c]'
'CPD0-2117[c]'
'Adenylated-ThiS-Proteins[c]'
'ssDNA-DNA-primer-hybrid[c]'
'UDP-MANNAC[c]'
'UDP-MANNACA[c]'
'CPD-194[c]'
'tRNAs-with-queuine[c]'
'TRANS-3-METHYL-GLUTACONYL-COA[c]'
'CPD-13109[c]'
'7-METHYLGUANOSINE-5-PHOSPHATE[c]'
'CPD-8630[c]'
'm7G5-pppR-mRNAs[c]'
'ADENYLOSUCC[c]'
'CPD-17206[c]'
'CPD-339[c]'
'TDP[c]'
'2-AMINOBENZOYL-COA[c]'
'2-AMINO-5-OXOCYCLOHEX-1-ENECARBOXYL-COA[c]'
'CPD-17262[c]'
'CPD-17263[c]'
'CPD-17264[c]'
'CPD-21416[c]'
'CPD-21415[c]'
'CPD-21417[c]'
'CPD-21418[c]'
'Cis-2-enoyl-CoAs[c]'
'Protein-tyrosine-phosphates[c]'
'CPD-17273[c]'
'CPD-21419[c]'
'CPD-21420[c]'
'Long-Chain-Polyphosphate[c]'
'D-SEDOHEPTULOSE-1-7-P2[c]'
'CPD-177[c]'
'2-Palmitoyl-L-Phosphatidate[c]'
'CPD-10712[c]'
'D-MYO-INOSITOL-34-BISPHOSPHATE[c]'
'CPD-193[c]'
'CPD-13954[c]'
'CPD-11915[c]'
'PRO-tRNAs[c]'
'Charged-PRO-tRNAs[c]'
'12-PROPANEDIOL-1-PHOSPHATE[c]'
'HYDROXYACETONE-PHOSPHATE[c]'
'CPD-207[c]'
'CPD-11740[c]'
'1-CARBOXYVINYL-CARBOXYPHOSPHONATE[c]'
'CPD-17328[c]'
'CPD-18492[c]'
'CPD-18493[c]'
'CPD-17365[c]'
'CPD-18494[c]'
'L-HISTIDINOL-P[c]'
'Acetoacetyl-ACP[c]'
'PHOSPHORIBOSYL-AMP[c]'
'PHOSPHORIBOSYL-FORMIMINO-AICAR-P[c]'
'OLIGOPHOSPHATE[c]'
'Polyphosphates[c]'
'Long-Chain-3S-Hydroxyacyl-CoAs[c]'
'Long-Chain-oxoacyl-CoAs[c]'
'CPD0-2472[c]'
'MAP-Kinase-L-Phosphothreonine[c]'
'CPD-13775[c]'
'L-1-PHOSPHATIDYL-SERINE[c]'
'CPD-9771[c]'
'CPD-9772[c]'
'3-HYDROHYDROXYPHOSPHORYLPYRUVATE[c]'
'CPD-10011[c]'
'CPD-11746[c]'
'Cytidine-34-tRNAIle2[c]'
'Lysidine-tRNA-Ile2[c]'
'HIS-tRNAs[c]'
'Charged-HIS-tRNAs[c]'
'CIS-DELTA3-ENOYL-COA[c]'
'CPD-13776[c]'
'CPD-13777[c]'
'CPD-472[c]'
'Actinorhodin-Intermediate-2[c]'
'Hepta-oxo-hexadecanoyl-ACPs[c]'
'ADP-D-GLUCOSE[c]'
'TREHALOSE-6P[c]'
'ALL-TRANS-HEPTAPRENYL-DIPHOSPHATE[c]'
'Aryl-Carrier-Proteins[c]'
'QXC-ACP[c]'
'CPD-18550[c]'
'ALL-TRANS-PENTAPRENYL-DIPHOSPHATE[c]'
'AcDMPT-L-Alanyl-PhsB[c]'
'PhsC[c]'
'Guanine1575-in-18StRNAs[c]'
'Holo-EntB[c]'
'CPD-10505[c]'
'PROPIONYL-P[c]'
'AcDMPT-L-Alanyl-L-Alanyl-PhsC[c]'
'PhsB[c]'
'AcDMPT-L-Alanyl-L-Leucyl-PhsC[c]'
'CPD-2185[c]'
'N-Substituted-Aminoacyl-tRNA[c]'
'N-Acylphosphatidylethanolamines[c]'
'N7-methylGuanine1575-in-18StRNAs[c]'
'Cytosine2870-in-25S-rRNA[c]'
'5-methylcytosine2870-in-25S-rRNA[c]'
'Cytosine2278-in-25S-rRNA[c]'
'5-methylcytosine2278-in-25S-rRNA[c]'
'25S-rRNA-uracil-2843[c]'
'25S-rRNA-N3-methyl-uracil-2843[c]'
'25S-rRNA-uracil-2634[c]'
'25S-rRNA-N3-methyl-uracil-2634[c]'
'CPD-10588[c]'
'CPD-72[c]'
'CPD-10589[c]'
'CPD-10590[c]'
'CPD-10591[c]'
'ERYTHRONATE-4P[c]'
'CPD-606[c]'
'CPD-609[c]'
'Sugar-1-Phosphate[c]'
'UDP-sugar[c]'
'ssRNAs[c]'
'CPD-13781[c]'
'CPD-13841[c]'
'CPD-13783[c]'
'CPD-13845[c]'
'CPD-13846[c]'
'CPD-13847[c]'
'CPD-13853[c]'
'tRNAs[c]'
'AMINO-PARATHION[c]'
'CPD-2186[c]'
'Protein-pi-phospho-L-histidines[c]'
'CPD-9610[c]'
'ALA-tRNAs[c]'
'glycyl-tRNAAla[c]'
'D-tyrosyl-tRNA-Tyr[c]'
'TYR-tRNAs[c]'
'D-aspartyl-tRNA-Asp[c]'
'D-Tryptophanyl-tRNA-Trp[c]'
'TRP-tRNAs[c]'
'Holo-EntF[c]'
'CPD-17136[c]'
'CPD-13855[c]'
'5-P-purine-mRNAs[c]'
'CPD-190[c]'
'LYSOSOMAL-ENZYME-N-ETCETERA-MANNOSE[c]'
'CPD-8559[c]'
'All-holo-ACPs[c]'
'CPD0-1137[c]'
'CPD-607[c]'
'CPD-13713[c]'
'G5-pppR-mRNAs[c]'
'1-L-MYO-INOSITOL-1-P[c]'
'CPD-15683[c]'
'5-PHOSPHORIBOSYL-5-AMINOIMIDAZOLE[c]'
'Protein-phospho-ribulosamines[c]'
'INOSITOL-1456-TETRAKISPHOSPHATE[c]'
'Spliced-tRNA-precursor[c]'
'CPD-9007[c]'
'2-phospho-ligated-tRNA[c]'
'CPD-15635[c]'
'CPD-14723[c]'
'GDP-TP[c]'
'GUANOSINE-5DP-3DP[c]'
'CPD-14724[c]'
'tRNA-uridine-38-39[c]'
'tRNA-pseudouridine-38-39[c]'
'L-1-GLYCEROPHOSPHORYLETHANOL-AMINE[c]'
'Protein-phospho-psicosamines[c]'
'Protein-phospho-fructosamines[c]'
'Protein-phospho-erythrulosamines[c]'
'CPD-14432[c]'
'2-methylbutanoyl-LovF[c]'
'Holo-LovF[c]'
'CPD-7302[c]'
'DIHYDRONEOPTERIN-P[c]'
'CPD-506[c]'
'CPD-8563[c]'
'CPD-13754[c]'
'CPD-13755[c]'
'Citrate-Lyase-Citryl-Form[c]'
'CITRATE-LYASE[c]'
'CPD-6661[c]'
'an-iNisup1sup-ethyladenine-in-DNA[c]'
'CARBOXYMETHYL-HYDROXYPHENYLPROPCOA[c]'
'DNA-Adenines[c]'
'E-PHENYLITACONYL-COA[c]'
'CPD-6746[c]'
'CPD-13013[c]'
'UDP-AA-GLUTAMATE[c]'
'CPD-13018[c]'
'CPD-16954[c]'
'CPD-1776[c]'
'4-MHA-THR-VAL-AcmD-Proteins[c]'
'4-HMA-AcmD-Proteins[c]'
'CPD-3705[c]'
'CPD-3709[c]'
'CPD-3713[c]'
'CPD-3710[c]'
'CPD-3723[c]'
'CPD-3725[c]'
'CPD-264[c]'
'CPD-16955[c]'
'2-METHYL-ACETO-ACETYL-COA[c]'
'Protein-L-Ser-or-L-Thr-P-L-Pro[c]'
'MAPKK-Ser-or-Thr-phosphate[c]'
'Receptor-Protein-Ser-or-Thr-phosphate[c]'
'CGMP[c]'
'D-HEXOSE-6-PHOSPHATE[c]'
'BENZOYLSUCCINYL-COA[c]'
'CPD-19339[c]'
'PYRIDOXINE-5P[c]'
'CPD-13757[c]'
'CPD-13758[c]'
'Long-Chain-Acyl-ACPs[c]'
'CPD-17329[c]'
'CPD-21411[c]'
'CPD-18489[c]'
'CPD-18490[c]'
'Myosin-heavy-chain-phosphates[c]'
'Protein-Phosphoserines[c]'
'P-Nitrophenyl-5-Nucleotides[c]'
'CPD0-2107[c]'
'CPD-3724[c]'
'CPD-9443[c]'
'DTDP-D-GLUCOSE[c]'
'Cyclic-N6-threonylcarbamoyl-A37-tRNAs[c]'
'CPD-18350[c]'
'1-PALMITOYLGLYCEROL-3-PHOSPHATE[c]'
'Palmitoleoyl-ACPs[c]'
'CPD-18348[c]'
'CPD-18351[c]'
'Cis-vaccenoyl-ACPs[c]'
'CPD-18352[c]'
'CPD-18353[c]'
'CPDQT-400[c]'
'Phospholipids[e]'
'CPD-499[c]'
'CPD-641[c]'
'D-RIBULOSE-15-P2[c]'
'CPD1G-768[c]'
'CPD1G-771[c]'
'METHYL-MALONYL-COA[c]'
'CPD-1106[c]'
'CPD-551[c]'
'CPD-12896[c]'
'CPD-1827[c]'
'CPD-12902[c]'
'CPD-15650[c]'
'CPD-18379[c]'
'BIO-5-AMP[c]'
'CPD-18380[c]'
'PYRIDOXAMINE-5P[c]'
'CPD-3708[c]'
'CPD-3706[c]'
'CPD-12904[c]'
'CPD-12905[c]'
'CPD-12906[c]'
'CPD0-1425[c]'
'CPD-18390[c]'
'CPD-13692[c]'
'CPD-13694[c]'
'CPD-13695[c]'
'CPD-13696[c]'
'CPD0-2253[c]'
'CPD-18195[c]'
'CPD0-2030[c]'
'CPD-18171[c]'
'1-Acyl-sn-glycero-3-phosphocholines[c]'
'1-ACYL-2-OLEOYL-SN-GLYCERO-3-PHOSPHOCHOL[c]'
'holo-Peptidyl-carrier-proteins[c]'
'CPD-20036[c]'
'CPD-9387[c]'
'CPD-14205[c]'
'CPD-627[c]'
'METHYLENETETRAHYDROMETHANOPTERIN[c]'
'THMPT[c]'
'CPD-13698[c]'
'CPD-13699[c]'
'CPD-13700[c]'
'ATP[e]'
'ADP[e]'
'3-Phosphopolynucleotides[c]'
'5-Dephospho-DNA[c]'
'5-Phospho-DNA[c]'
'5-ppp-Pur-mRNA[c]'
'Holo-Aryl-Carrier-Proteins[c]'
'2-C-METHYL-D-ERYTHRITOL-4-PHOSPHATE[c]'
'C4[c]'
'CPD-7695[c]'
'CPD-1137[c]'
'3-ENOLPYRUVYL-SHIKIMATE-5P[c]'
'CPD0-181[c]'
'CPD-17414[c]'
'CPD-15979[c]'
'C5[c]'
'C1[c]'
'L-1-PHOSPHATIDYL-GLYCEROL-P[c]'
'5-Methylcytosine-DNA[c]'
'CPD-12706[c]'
'CPD-464[c]'
'CPD-20681[c]'
'CPD-20682[c]'
'Charged-TYR-tRNAs[c]'
'N-formyl-L-methionyl-tRNAfmet[c]'
'CPD-14332[c]'
'CPD-18240[c]'
'CPD-18265[c]'
'Charged-ALA-tRNAs[c]'
'CPD-18209[c]'
'CPD-18266[c]'
'1-ACYL-2-LINOLEOYL-SN-GLYCERO-3-PHOSPHOC[c]'
'CPD-20684[c]'
'CPD-20735[c]'
'CPD-20741[c]'
'DNA-pyrimidines[c]'
'CPD-20742[c]'
'GDP-D-GLUCOSE[c]'
'2-ALPHA-HYDROXYETHYL-THPP[c]'
'Glutamine-synthetase-adenylyl-Tyr[c]'
'CPD-10699[c]'
'CPD-8973[c]'
'CPD-8974[c]'
'METHACRYLYL-COA[c]'
'N-ACETYL-D-GALACTOSAMINE-6-PHOSPHATE[c]'
'D-GALACTOSAMINE-6-PHOSPHATE[c]'
'CPD-11643[c]'
'CPD-20743[c]'
'CPD-20736[c]'
'CPD-20737[c]'
'CPD-20744[c]'
'DNA-thymidine-deoxycytidine-dimer[c]'
'CPDQT-4[c]'
'CPD-557[c]'
'SHIKIMATE-5P[c]'
'tRNA-pseudouridine55[c]'
'tRNA-uridine55[c]'
'tRNA-uridine65[c]'
'tRNA-pseudouridine65[c]'
'tRNA-pseudouridine13[c]'
'DIETHYLPHOSPHATE[c]'
'PARAOXON[c]'
'CPD-8978[c]'
'ADENOSYLCOBALAMIN-5-P[c]'
'ADENOSYLCOBALAMIN[c]'
'2-LYSOPHOSPHATIDYLETHANOLAMINES[c]'
'3-HYDROXY-PROPIONYL-COA[c]'
'SN-GERANYLGERANYLGLYCERYL-1-PHOSPHATE[c]'
'tRNA-uridine13[c]'
'tRNA-pseudouridine32[c]'
'Uridine32-in-tRNA[c]'
'23S-rRNA-uridine-2552[c]'
'23S-rRNA-2-O-methyluridine2552[c]'
'5-METHYLCYTOSINE-34-TRNA-PRECURSORS[c]'
'Cytosine-34-tRNA-Precursors[c]'
'Cytosine-40-tRNA-Precursors[c]'
'5-Methylcytosine-40-tRNA-Precursors[c]'
'5-Methylcytosine-48-tRNAs[c]'
'Cytosine-48-tRNAs[c]'
'CPD-19133[c]'
'Phosphorylated-phosphoglucomutase[c]'
'GLYCEROPHOSPHOGLYCEROL[c]'
'CPD-3711[c]'
'Cytosine-49-tRNAs[c]'
'5-Methylcytosine-49-tRNAs[c]'
'Cytidine-32-tRNAs[c]'
'2-O-Methylcytidine-32-tRNAs[c]'
'2-O-MeGuan-34-tRNAs[c]'
'guanosine-34-tRNAs[c]'
'CPD-9445[c]'
'Dolichol-PP[c]'
'Diribonucleotide[c]'
'CPD-14762[c]'
'Glycerophosphodiesters[c]'
'MANNITOL-1P[c]'
'2-ACYL-GPE[c]'
'CPD-13852[c]'
'CPD-11525[c]'
'CPD-11526[c]'
'CPD-11529[c]'
'ADP-SUGARS[c]'
'N-acyl-sphingosylphosphorylcholine[c]'
'R-3-hydroxystearoyl-ACPs[c]'
'PHE-tRNAs[c]'
'Charged-PHE-tRNAs[c]'
'Adenine-37-tRNA-Alas[c]'
'Hypoxanthine-37-In-tRNA-Alanines[c]'
'Adenine-34-in-tRNAs[c]'
'CPD-12645[c]'
'56-Dihydrouracil16-in-tRNAs[c]'
'Uracil16-in-tRNAs[c]'
'tRNAs-with-CCA[c]'
'Guanine46-in-tRNA[c]'
'tRNA-Containing-N7-Methylguanine-46[c]'
'tRNA-pseudouridine-38-40[c]'
'tRNA-uridine-38-40[c]'
'Uracil-54-in-tRNA[c]'
'tRNA-containing-5Me-uridine54[c]'
'CPD0-1422[c]'
'Guanidinoacetyl-PKS[c]'
'Aminomalonyl-PCP[c]'
'Loaded-GdnH[c]'
'holo-Seryl-Carrier-Proteins[c]'
'CPD-166[c]'
'CPD-18076[c]'
'Hypoxanthine-In-tRNAs-34s[c]'
'Cytosine-32-tRNA-Thrs[c]'
'N3-Methylcytosine-32-tRNA-Thr[c]'
'Cytosine-32-In-tRNAs-Sers[c]'
'N3-Methylcytosine-32-tRNA-Sers[c]'
'BENZYLSUCCINYL-COA[c]'
'CPD-18238[c]'
'NN-DIACETYLCHITOBIOSYLDIPHOSPHODOLICHO[c]'
'ALPHA-D-MANNOSYLCHITOBIO[c]'
'DNA-thymidine-dimer[c]'
'CPD-12677[c]'
'CPD-16526[c]'
'Charged-TRP-tRNAs[c]'
'CPD-13576[c]'
'DNA-deoxycytidine-dimer[c]'
'CPD-18011[c]'
'medium-Chain-Trans-23-Dehydroacyl-CoA[c]'
'CPD-67[c]'
'Elongation-tRNAMet[c]'
'Charged-MET-tRNAs[c]'
'CPD-18260[c]'
'CPD-11519[c]'
'CPD-11518[c]'
'CPD-11520[c]'
'CPD-13533[c]'
'CPD-7246[c]'
'2-3-4-Saturated-L-Phosphatidates[c]'
'an-Nsup1sup-methyladenine-in-DNA[c]'
'T2-C4-DECADIENYL-COA[c]'
'an-Nsup3sup-methylcytosine-in-DNA[c]'
'CPD-20035[c]'
'CPD-18059[c]'
'Alkylated-DNAs[c]'
'DNA-containing-diamino-hydro-formamidops[c]'
'ADP-D-ribosyl-nitrogen-reductases[c]'
'Lysophospholipids[c]'
'CPD-13534[c]'
'CPD-14729[c]'
'CPD-14772[c]'
'CPD-14828[c]'
'Charged-tRNAs[c]'
'GLY-tRNAs[c]'
'K-HEXANOYL-COA[c]'
'Charged-GLY-tRNAs[c]'
'CPD-14928[c]'
'CPD-11521[c]'
'CPD-11524[c]'
'21S-rRNA-uridine-2791[c]'
'21S-rRNA-2-O-methyluridine2791[c]'
'Holo-LYS2-peptidyl-carrier-protein[c]'
'2-Aminoadipyl-LYS2[c]'
'CPD-21867[c]'
'Rifamycin-Undecaketide-Acps[c]'
'Saturated-2-Lysophosphatidates[c]'
'2-Me-Branched-234-Sat-Fatty-Acyl-CoA[c]'
'CPD-14675[c]'
'OH-HEXANOYL-COA[c]'
'OLIGOSACCHARIDE-DIPHOSPHODOLICHOL[c]'
'CPD-20211[c]'
'CPD-18078[c]'
'CPD-11528[c]'
'CPD-11523[c]'
'Guanosine-2922-in-27S-pre-rRNA[c]'
'2-O-methylguanosine-2922-in-27S-pre-rRNA[c]'
'23S-rRNA-guanine-2551[c]'
'23S-rRNA-2-O-methylguanosine2251[c]'
'23S-rRNA-adenine-1618[c]'
'CPD-5161[c]'
'23S-rRNA-N6-m-adenine1618[c]'
'CPD-5162[c]'
'CPD-15923[c]'
'CPD-405[c]'
'CPD-7257[c]'
'CDP-2-3-4-Saturated-Diacylglycerols[c]'
'tRNA-Adenosines-37[c]'
'6-Dimethylallyladenosine37-tRNAs[c]'
'tRNA-containing-5-taurinomethyluridine[c]'
'CPD-5169[c]'
'CPD-11527[c]'
'CPD-11522[c]'
'16S-rRNA-adenine1518-adenine1519[c]'
'16S-rRNA-N6-dimethyladenine1518-1519[c]'
'18S-rRNA-N6-dimethyladenine1779-1780[c]'
'1-2-DIPALMITOYLPHOSPHATIDYLCHOLINE[c]'
'3-oxo-cis-vaccenoyl-ACPs[c]'
'CPD-5170[c]'
'NAcMur-Peptide-NAcGlc-Undecaprenols[c]'
'NAcMur-4Peptide-NAcGlc-Undecaprenols[c]'
'UDP-MURNAC-TETRAPEPTIDE[c]'
'18S-rRNA-adenine1779-adenine1780[c]'
'CPD-8159[c]'
'L-GLUTAMATE-5-P[c]'
'3-PHOSPHATIDYL-3-O-L-LYSYLGLYCEROL[c]'
'CPD-15716[c]'
'CPD-4201[c]'
'Glutaryl-ACP-methyl-esters[c]'
'CPD-17927[c]'
'CPD-17926[c]'
'CPD-4203[c]'
'3-Ketopimeloyl-ACP-methyl-esters[c]'
'Petroselinoyl-ACPs[c]'
'3-oxo-cis-D7-tetradecenoyl-ACPs[c]'
'3-hydroxy-cis-D7-tetraecenoyl-ACPs[c]'
'tRNA-guanosine18[c]'
'CPD-8355[c]'
'2-O-Methylguanosine18[c]'
'CPD-8291[c]'
'D-aminoacyl-tRNAs[c]'
'Delta4-hexadecenoyl-ACPs[c]'
'3-oxo-petroselinoyl-ACPs[c]'
'Uridine31-in-tRNA[c]'
'tRNA-pseudouridine-31[c]'
'p-his-tRNAS[c]'
'App-his-tRNAs[c]'
'CPD-4206[c]'
'R-3-hydroxypetroselinoyl-ACPs[c]'
'3-hydroxypimeloyl-ACP-methyl-esters[c]'
'PALMITOYLGLYCERONE-PHOSPHATE[c]'
'DNA-6-O-Methyl-Guanines[c]'
'DNA-Guanines[c]'
'DNA-Containing-N6-Methyladenine[c]'
'CPD-8268[c]'
'CPD-15924[c]'
'Trans-D2-cis-D7-tetradecenoyl-ACPs[c]'
'Cis-Delta7-tetradecenoyl-ACPs[c]'
'3-oxo-cis-D9-hexadecenoyl-ACPs[c]'
'3-HYDROXY-ISOBUTYRYL-COA[c]'
'THR-tRNAs[c]'
'DNA-With-GO[c]'
'Enoylpimeloyl-ACP-methyl-esters[c]'
'Pimeloyl-ACP-methyl-esters[c]'
'Pimeloyl-ACPs[c]'
'cis-vaccen-2-enoyl-ACPs[c]'
'CPD-8343[c]'
'3-Methyl-Saturated-Fatty-Acyl-CoA[c]'
'2-OH-3-Methyl-Saturated-Fatty-Acyl-CoA[c]'
'3-hydroxy-cis-D9-hexaecenoyl-ACPs[c]'
'Trans-D3-cis-D9-hexadecenoyl-ACPs[c]'
'CPD-14767[c]'
'CPD-14768[c]'
'D-RIBULOSE-1-P[c]'
'CPD-19839[c]'
'CPD-19840[c]'
'cis-5-enoyl-CoA[c]'
'trans-2-cis-5-dienoyl-CoA[c]'
'trans-3-cis-5-dienoyl-CoA[c]'
'4812-TRIMETHYLTRIDECANOYL-COA[c]'
'3-OXOPRISTANOYL-COA[c]'
'CPD-12461[c]'
'CPD-17932[c]'
'CPD-17983[c]'
'CPD-17997[c]'
'CPD-15961[c]'
'CPD-15962[c]'
'Holo-AsbD-Proteins[c]'
'Glycerol-1-phosphate[c]'
'CPD-11517[c]'
'VLC-MIP2C[c]'
'CPD-10225[c]'
'CPD-10226[c]'
'CPD-10227[c]'
'Ansamycin-PKS-ACP-Domain[c]'
'Amino-Hydroxybenzoyl-Acps[c]'
'Amino-Hydroxybenzoyl-Rifamycin-PKS[c]'
'Rifamycin-Tetraketide-Acps[c]'
'CPD-14404[c]'
'Holo-SfmC-peptidyl-carrier-protein[c]'
'3O-4-dimethyl-5-OH-L-tyr-SfmC[c]'
'Acyl-alanylglycinyl-SfmB[c]'
'Holo-SfmB-peptidyl-carrier-protein[c]'
'CPD-18035[c]'

-------------------------------------------------------------------------------

The chemical element **S** is present in the empirical formula of **1196 metabolites**

'CO-A[c]'
'OLEOYL-COA[c]'
'ACETYL-COA[c]'
'Odd-Saturated-Fatty-Acyl-CoA[c]'
'2E-5Z-tetradeca-2-5-dienoyl-ACPs[c]'
'5Z-tetradec-5-enoyl-ACPs[c]'
'SULFATE[c]'
'MALONYL-ACP[c]'
'7Z-3-oxo-hexadec-7-enoyl-ACPs[c]'
'ACP[c]'
'CHONDROITIN-4-SULFATE[c]'
'Heparan-sulfate-L-iduronate[c]'
'Heparan-sulfate-L-IdoA-2S[c]'
'Heparan-NAc-Glc-6S[c]'
'LINOLENOYL-COA[c]'
'CPD-12384[c]'
'CPD-12385[c]'
'S-ADENOSYLMETHIONINE[c]'
'CPD-12388[c]'
'ADENOSYL-HOMO-CYS[c]'
'CPD-12387[c]'
'CPD-12390[c]'
'CPD-12391[c]'
'CPD-12393[c]'
'CPD-12139[c]'
'CPD-19502[c]'
'Elemental-Sulfur[c]'
'CPD-19504[c]'
'CPD-19503[c]'
'CPD-208[c]'
'PROTEIN-LIPOYLLYSINE[c]'
'MET[c]'
'Oxidized-2Fe-2S-Ferredoxins[c]'
'Sulfurated-Sulfur-Acceptors[c]'
'Reduced-2Fe-2S-Ferredoxins[c]'
'CPD-196[c]'
'GLUTARYL-COA[c]'
'MALONYL-COA[c]'
'HEXANOYL-COA[c]'
'CPD-14687[c]'
'CPD-14615[c]'
'GLUTACONYL-COA[c]'
'CPD-18[c]'
'Long-Chain-234-Saturated-acyl-CoAs[c]'
'Long-Chain-Trans-23-Dehydroacyl-CoA[c]'
'biotin-L-lysine-in-BCCP-dimers[c]'
'carboxybiotin-L-lysine-in-BCCP-dimers[c]'
'BIOTIN[c]'
'BCCP-biotin-L-lysine[c]'
'3R-7Z-3-hydroxy-hexadec-7-enoyl-ACPs[c]'
'2E-7Z-hexadeca-2-7-dienoyl-ACPs[c]'
'7Z-hexadec-7-enoyl-ACPs[c]'
'9Z-3-oxo-octadec-9-enoyl-ACPs[c]'
'CPD0-1158[c]'
'CPD0-1162[c]'
'Oxo-glutarate-dehydrogenase-DH-lipoyl[c]'
'CPD0-1163[c]'
'CPD-12394[c]'
'CPD-12397[c]'
'CPD-12396[c]'
'CPD-12399[c]'
'CPD-12400[c]'
'CPD-12402[c]'
'CPD-12403[c]'
'CPD-12405[c]'
'CPD-12406[c]'
'BCAA-dehydrogenase-DH-lipoyl[c]'
'PHENYLACETOTHIOHYDROXIMATE[c]'
'PROTEIN-C-TERMINAL-S-ETC-CYSTEINE[c]'
'PROTEIN-C-TERMINAL-S-FARNESYL-L-CYSTEINE[c]'
'DIHYDROLIPOAMIDE[c]'
'LIPOAMIDE[c]'
'PALMITYL-COA[c]'
'CPD-17621[c]'
'CROTONYL-COA[c]'
'GLUTATHIONE[c]'
'OXIDIZED-GLUTATHIONE[c]'
'R-4-PHOSPHOPANTOTHENOYL-L-CYSTEINE[c]'
'PANTETHEINE-P[c]'
'Pyruvate-dehydrogenase-lipoate[c]'
'Oxo-glutarate-dehydrogenase-lipoyl[c]'
'DEPHOSPHO-COA[c]'
'CPD-14704[c]'
'CPD-14705[c]'
'CPD-14706[c]'
'CPD-4[c]'
'2-METHYL-3-HYDROXY-BUTYRYL-COA[c]'
'CPD-1083[c]'
'Thiocarboxylated-MPT-synthases[c]'
'CPD-8122[c]'
'3R-9Z-3-hydroxy-octadec-9-enoyl-ACPs[c]'
'2E-9Z-octadeca-2-9-dienoyl-ACPs[c]'
'CPD-1861[c]'
'CPD-12449[c]'
'CPD66-21[c]'
'LEUKOTRIENE-C4[c]'
'7E9E11Z14Z-5S6R-6-CYSTEIN-S-YL[c]'
'Long-Chain-Acyl-CoAs[c]'
'CPD-239[c]'
'3-SULFINOALANINE[c]'
'L-CYSTEATE[c]'
'R-3-Hydroxypalmitoyl-ACPs[c]'
'PAPS[c]'
'CPD-11407[c]'
'CPD-11408[c]'
'3-oxo-palmitoyl-ACPs[c]'
'CYS[c]'
'L-Cysteine-Desulfurase-persulfide[c]'
'Cysteine-Desulfurase-L-cysteine[c]'
'CPD-8123[c]'
'Mercapturates[c]'
'S-Substituted-L-Cysteines[c]'
'L-GAMMA-GLUTAMYLCYSTEINE[c]'
'Reduced-adrenal-ferredoxins[c]'
'Oxidized-adrenal-ferredoxins[c]'
'Malonyl-acp-methyl-ester[c]'
'3-Ketoglutaryl-ACP-methyl-ester[c]'
'PROPIONYL-COA[c]'
'D-METHYL-MALONYL-COA[c]'
'P-COUMAROYL-COA[c]'
'CAFFEOYL-COA[c]'
'BUTYRYL-COA[c]'
'TETRADECANOYL-COA[c]'
'CPD-10267[c]'
'STEAROYL-COA[c]'
'Oleoyl-ACPs[c]'
'ACETYL-ACP[c]'
'11Z-3-oxo-icos-11-enoyl-ACPs[c]'
'2-Hexadecenoyl-ACPs[c]'
'Palmitoyl-ACPs[c]'
'CPD-15896[c]'
'CPD-17487[c]'
'CPD-8124[c]'
'CPD0-2244[c]'
'CPD0-2123[c]'
'LAUROYLCOA-CPD[c]'
'4-HYDROXY-BUTYRYL-COA[c]'
'OH-CROTONYL-COA[c]'
'3-Hydroxyglutaryl-ACP-methyl-ester[c]'
'CPD-19760[c]'
'Enoylglutaryl-ACP-methyl-esters[c]'
'ACYL-COA[c]'
'3R-11Z-3-hydroxy-icos-11-enoyl-ACPs[c]'
'2E-11Z-icosa-2-11-dienoyl-ACPs[c]'
'11Z-icos-11-enoyl-ACPs[c]'
'HS[c]'
'CPD-10260[c]'
'CPD-10261[c]'
'CPD-10262[c]'
'Stearoyl-ACPs[c]'
'CPD-15900[c]'
'3-HYDROXYPIMELYL-COA[c]'
'Cis-Delta5-dodecenoyl-ACPs[c]'
'Saturated-Fatty-Acyl-CoA[c]'
'Sulfhydryls[c]'
'CPD-8529[c]'
'Methyl-thioethers[c]'
'Thiols[c]'
'Reduced-ferredoxins[c]'
'Oxidized-ferredoxins[c]'
'E-11-TETRADECENOYL-COA[c]'
'CPD-17814[c]'
'CPD-17813[c]'
'CPD-17815[c]'
'3-OXOPALMITOYL-COA[c]'
'CPD-17464[c]'
'Dodecanoyl-ACPs[c]'
'3-oxo-myristoyl-ACPs[c]'
'CPD-15684[c]'
'CPD-15685[c]'
'CPD-15686[c]'
'CPD-15687[c]'
'CPD-15688[c]'
'SO3[c]'
'S2O3[c]'
'CPD-15689[c]'
'CPD-15690[c]'
'CPD-15692[c]'
'CPD-15691[c]'
'ACETOACETYL-COA[c]'
'5-METHYLTHIOADENOSINE[c]'
'S-ADENOSYLMETHIONINAMINE[c]'
'BENZOYLCOA[c]'
'CPD-20052[c]'
'CPD-20051[c]'
'Thiopurine-Methylethers[c]'
'Thiopurines[c]'
'Red-Thioredoxin[c]'
'Ox-Thioredoxin[c]'
'CPD-17870[c]'
'CPD-17877[c]'
'CPD-3746[c]'
'T2-DECENOYL-COA[c]'
'CPD-12777[c]'
'D-CYSTEINE[c]'
'CPD-85[c]'
'CPD-8999[c]'
'2-METHYL-BUTYRYL-COA[c]'
'HSCN[c]'
'CPD-17882[c]'
'CPD-19388[c]'
'CPD-15277[c]'
'CYS-GLY[c]'
'R-3-hydroxymyristoyl-ACPs[c]'
'VERY-LONG-CHAIN-FATTY-ACYL-COA[c]'
'CPD-15781[c]'
'CPD-397[c]'
'CPD-19475[c]'
'2-OXO-5-METHYLTHIOPENTANOIC-ACID[c]'
'CPDQT-40[c]'
'CPDQT-39[c]'
'CPD-19488[c]'
'CPDQT-38[c]'
'CPD-19489[c]'
'Tetradec-2-enoyl-ACPs[c]'
'3Z-dodec-3-enoyl-ACPs[c]'
'OXALYL-COA[c]'
'3R-5Z-3-hydroxy-tetradec-5-enoyl-ACPs[c]'
'5Z-3-oxo-tetradec-5-enoyl-ACPs[c]'
'HOMO-CYS[c]'
'CPD-10254[c]'
'SUC-COA[c]'
'3-KETO-ADIPYL-COA[c]'
'CPD-19490[c]'
'CPDQT-37[c]'
'CPDQT-36[c]'
'CPD-19491[c]'
'ACYL-ACP[c]'
'CPD-3740[c]'
'CPD-19493[c]'
'Omega-methylthio-alkyl-glucosinolates[c]'
'omega-methylsulfinylalkylglucosinolate[c]'
'CAAL-proteins[c]'
'Geranylgeranylated-CAAL-proteins[c]'
'Myristoyl-ACPs[c]'
'CPD-15567[c]'
'CPD-15568[c]'
'Cytochromes-C-Oxidized[c]'
'Cytochromes-C-Reduced[c]'
'CPD-17732[c]'
'CPD-17733[c]'
'CPD-17741[c]'
'Decanoyl-ACPs[c]'
'3-oxo-dodecanoyl-ACPs[c]'
'CPD-15666[c]'
'Lignoceroyl-ACPs[c]'
'3-oxo-cerotoyl-ACPs[c]'
'CPD-17743[c]'
'S-HYDROXYMETHYLGLUTATHIONE[c]'
'CPD-17744[c]'
'CPD-17746[c]'
'CPD-17750[c]'
'CPD-548[c]'
'CPD-17757[c]'
'CPD-3745[c]'
'CPD-201[c]'
'CPD-202[c]'
'Reduced-ferredoxins[e]'
'R-3-hydroxydodecanoyl-ACPs[c]'
'THZ-P[c]'
'THIAMINE-P[c]'
'THIAMINE-PYROPHOSPHATE[c]'
'CPD-611[c]'
'THIAMINE[c]'
'THZ[c]'
'CPD-15637[c]'
'CPD-15653[c]'
'CPD-15668[c]'
'CPD-15667[c]'
'CPD-15654[c]'
'CPD-15655[c]'
'CPD-13665[c]'
'R-3-hydroxycerotoyl-ACPs[c]'
'Trans-D2-hexacos-2-enoyl-ACPs[c]'
'Cerotoyl-ACPs[c]'
'CPD-14392[c]'
'CPD-14018[c]'
'SULFATE[e]'
'HS[e]'
'FERULOYL-COA[c]'
'3-KETOACYL-COA[c]'
'L-3-HYDROXYACYL-COA[c]'
'Dodec-2-enoyl-ACPs[c]'
'Thiocarboxyadenylated-ThiS-Proteins[c]'
'CPD-13575[c]'
'SO3[e]'
'Elemental-Sulfur[e]'
'THIAMINE[e]'
'CPD-14407[c]'
'CPD-17794[c]'
'CPD-14422[c]'
'CPD-14423[c]'
'CPD-14424[c]'
'CPD-14425[c]'
'CPD-14426[c]'
'CPD-13328[c]'
'CPD-15656[c]'
'CPD-15657[c]'
'CPD-15675[c]'
'CPD-15651[c]'
'CPD-15652[c]'
'CPD-15677[c]'
'CPD-15676[c]'
'CPD-17883[c]'
'S-ADENOSYL-4-METHYLTHIO-2-OXOBUTANOATE[c]'
'CPD-17802[c]'
'Z-11-TETRADECENOYL-COA[c]'
'CPD-15661[c]'
'CPD-10832[c]'
'CPD-15662[c]'
'CPD-15678[c]'
'CPD-15663[c]'
'CPD-9407[c]'
'CPD-15658[c]'
'Short-Chain-Trans-23-Dehydroacyl-CoA[c]'
'Short-Chain-234-Saturated-acyl-CoAs[c]'
'Very-Long-Chain-Trans-23-Dehydroacyl-CoA[c]'
'Very-long-Chain-234-Saturated-acyl-CoAs[c]'
'CPD-19474[c]'
'CPD-19486[c]'
'CPD-19487[c]'
'TRYPANOTHIONE-DISULFIDE[c]'
'TRYPANOTHIONE[c]'
'Dihydro-Lipoyl-Proteins[c]'
'Lipoyl-Protein-N6-lipoyllysine[c]'
'3-Hydroxy-octanoyl-ACPs[c]'
'3-Oxo-octanoyl-ACPs[c]'
'Octadec-2-enoyl-ACPs[c]'
'SULFO-CYSTEINE[c]'
'CPD-11281[c]'
'N-5S-5-AMINO-5-CARBOXYPENTANOYL-L-CY[c]'
'Propionyl-CoA-CO2-ligases[c]'
'Biotin-EC6-4-1-4[c]'
'CPD-14280[c]'
'CPD-10279[c]'
'CPD-14281[c]'
'CPD-10280[c]'
'CPD-14282[c]'
'CPD1G-277[c]'
'CPD-14283[c]'
'CPD-14300[c]'
'CPD-479[c]'
'2-2-METHYLTHIOETHYLMALIC-ACID[c]'
'3-2-METHYLTHIOETHYLMALIC-ACID[c]'
'HOMOMETHIONINE[c]'
'N-Ac-L-methionyl-L-tyrosinyl-Protein[c]'
'N-terminal-L-cysteine[c]'
'N-terminal-N-Ac-L-cysteine[c]'
'CPD-9406[c]'
'CPD-17635[c]'
'CoM[c]'
'2-OXOPROPYL-COM[c]'
'S-PRENYL-L-CYSTEINE[c]'
'Protein-L-methionine-R-S-oxides[c]'
'Protein-L-methionine[c]'
'CPD-8989[c]'
'S-palmitoyl-L-cysteine-in-proteins[c]'
'PROT-CYS[c]'
'2-Octenoyl-ACPs[c]'
'Octanoyl-ACPs[c]'
'5-HYDROXY-FERULOYL-COA[c]'
'4-SULFOBENZALDEHYDE[c]'
'CPD-257[c]'
'CPD-12180[c]'
'SINAPOYL-COA[c]'
'3-METHYLTHIOPROPANALDOXIME[c]'
'CPD-7546[c]'
'3-METHYLTHIOPROPYLHYDROXAMIC-ACID[c]'
'3-METHYLTHIOPROPYL-DESULFO-GLUCOSINOLATE[c]'
'3-METHYLTHIOPROPYL-GLUCOSINOLATE[c]'
'3-METHYLSULFINYLPROPYL-GLUCOSINOLATE[c]'
'2-PROPENYL-GLUCOSINOLATE[c]'
'CPD-8990[c]'
'GLUTATHIONE[e]'
'APS[c]'
'L-methionyl-L-lysyl-Protein[c]'
'N-Ac-L-methionyl-L-lysyl-Protein[c]'
'L-methionyl-L-valyl-Protein[c]'
'N-Ac-L-methionyl-L-valyl-Protein[c]'
'L-methionyl-L-alanyl-Protein[c]'
'N-Ac-L-methionyl-L-alanyl-Protein[c]'
'CPD-17370[c]'
'CPD-15436[c]'
'Butanoyl-ACPs[c]'
'3-oxo-decanoyl-ACPs[c]'
'SULFOACETALDEHYDE[c]'
'3-HYDROXYADIPYL-COA[c]'
'TRANS-23-DEHYDROADIPYL-COA[c]'
'5-METHYLTHIOINOSINE[c]'
'MET[e]'
'CPD-12199[c]'
'L-methionyl-L-seryl-Protein[c]'
'N-Ac-L-methionyl-L-seryl-Protein[c]'
'L-methionyl-L-threonyl-Protein[c]'
'N-Ac-L-methionyl-L-threonyl-Protein[c]'
'L-methionyl-L-cysteinyl-Protein[c]'
'L-methionyl-glycyl-Protein[c]'
'CPD-17641[c]'
'CPD-17638[c]'
'CPD-7545[c]'
'CYS[e]'
'HOMO-CYS[e]'
'LIPOIC-ACID[e]'
'Beta-hydroxydecanoyl-ACPs[c]'
'CPD-10600[c]'
'CPD-19217[c]'
'S-NITROSOGLUTATHIONE[c]'
'CPD-7671[c]'
'CPD-17624[c]'
'CPD-17714[c]'
'CPD-17701[c]'
'CPD-15566[c]'
'THIOHYDROXIMATE-O-SULFATES[c]'
'CPD-558[c]'
'Alkenyl-Thiohydroximate-O-Sulfates[c]'
'EPITHIONITRILES[c]'
'L-arginyl-3-sulfino-L-alaninyl-Peptides[c]'
'N-terminal-L-cysteine-sulfinate[c]'
'N-terminal-L-cysteine-sulfonate[c]'
'L-arginyl-3-sulfo-L-alaninyl-Peptides[c]'
'N-Ac-L-methionyl-L-asparaginyl-Protein[c]'
'CPD0-2015[c]'
'N-Ac-L-methionyl-L-glutaminyl-Protein[c]'
'Trans-D2-decenoyl-ACPs[c]'
'L-CYSTATHIONINE[c]'
'CPD-6972[c]'
'CPD-22025[c]'
'CPD-10556[c]'
'Ultra-Long-Chain-Acyl-CoAs[c]'
'ISOVALERYL-COA[c]'
'BCAA-dehydrogenase-3MB-DH-lipoyl[c]'
'CPD-18831[c]'
'CPD-18832[c]'
'Cytochromes-C-Reduced[e]'
'Cytochromes-C-Oxidized[e]'
'CPD-1862[c]'
'Ergothioneine[c]'
'CPD-19154[c]'
'CPD-19161[c]'
'CPD-19157[c]'
'CPD-19148[c]'
'3-oxo-hexanoyl-ACPs[c]'
'R-3-hydroxyhexanoyl-ACPs[c]'
'L-methionyl-tRNAfmet[c]'
'CPD-17434[c]'
'CPD-17052[c]'
'CPD-17453[c]'
'CPD-9965[c]'
'Oxidized-NrdH-Proteins[c]'
'Reduced-NrdH-Proteins[c]'
'CPD-1863[c]'
'CPD-1881[c]'
'Hex-2-enoyl-ACPs[c]'
'CPD-17455[c]'
'CPD-19150[c]'
'CPD-17053[c]'
'CPD-19151[c]'
'CPD-19153[c]'
'3-SULFINYL-PYRUVATE[c]'
'CPD-22039[c]'
'CPD-14293[c]'
'CPD-22040[c]'
'D-3-HYDROXYACYL-COA[c]'
'CPD-22041[c]'
'CPD-14269[c]'
'CPD-22043[c]'
'CPD-15363[c]'
'3-oxo-stearoyl-ACPs[c]'
'THIOMORPHOLINE-3-CARBOXYLATE[c]'
'34-DEHYDRO-14-THIOMORPHOLINE-3-CARBOXY[c]'
'CPD-15361[c]'
'Red-Thioredoxin[e]'
'ACP[e]'
'CPDQT-273[c]'
'CPD-12140[c]'
'CPD1G-2[c]'
'CPD-12152[c]'
'HYPOTAURINE[c]'
'BCAA-dehydrogenase-lipoyl[c]'
'CPD-14925[c]'
'Hexanoyl-ACPs[c]'
'CPD-15364[c]'
'CPD-22044[c]'
'CPD-22045[c]'
'CPD-22048[c]'
'CPD-14271[c]'
'CPD-22050[c]'
'CPD-10283[c]'
'CPD-18491[c]'
'UDP-SULFOQUINOVOSE[c]'
'Oxidized-ferredoxins[e]'
'CPD-15362[c]'
'CPD-15368[c]'
'CPD-3736[c]'
'CPD-19144[c]'
'CPD-19170[c]'
'AMINOMETHYLDIHYDROLIPOYL-GCVH[c]'
'DIHYDROLIPOYL-GCVH[c]'
'CPD-14274[c]'
'CPD-14273[c]'
'CPD-14275[c]'
'CPD-19168[c]'
'CPD-14276[c]'
'CPD-14277[c]'
'Protein-S-methyl-L-cysteine[c]'
'Protein-Red-Disulfides[c]'
'L-methionyl-L-asparaginyl-Protein[c]'
'Protein-Ox-Disulfides[c]'
'L-methionyl-L-glutaminyl-Protein[c]'
'N-Ac-L-methionyl-L-aspartyl-Protein[c]'
'CPD-19167[c]'
'3-OXOPIMELOYL-COA[c]'
'CPD-15369[c]'
'CPD-15370[c]'
'CPD-15366[c]'
'CPD-14278[c]'
'L-methionyl-L-aspartyl-Protein[c]'
'N-Ac-L-methionyl-L-glutamyl-Protein[c]'
'L-methionyl-L-glutamyl-Protein[c]'
'L-methionyl-L-leucyl-Protein[c]'
'N-Ac-L-methionyl-L-leucyl-Protein[c]'
'L-methionyl-L-isoleucyl-Protein[c]'
'N-Ac-L-methionyl-L-isoleucyl-Protein[c]'
'L-methionyl-L-phenylalanyl-Protein[c]'
'N-Ac-L-methionyl-L-phenylalanyl-Protein[c]'
'L-methionyl-L-tryptophanyl-Protein[c]'
'N-Ac-L-methionyl-L-tryptophanyl-Protein[c]'
'L-methionyl-L-tyrosinyl-Protein[c]'
'CPD-19172[c]'
'CPD-19171[c]'
'CPD-19169[c]'
'CPD-444[c]'
'CPD-17312[c]'
'TRANS-D2-ENOYL-COA[c]'
'Trans-3-enoyl-CoAs[c]'
'GAMMA-LINOLENOYL-COA[c]'
'CPD-21828[c]'
'ARACHIDONYL-COA[c]'
'TAURINE[c]'
'Charged-CYS-tRNAs[c]'
'CPD-17387[c]'
'CPD-17386[c]'
'CPD-17388[c]'
'Behenoyl-ACPs[c]'
'3-oxo-lignoceroyl-ACPs[c]'
'R-3-hydroxylignoceroyl-ACPs[c]'
'trans-delta2-lignoceroyl-ACPs[c]'
'N-METHYLANTHRANILOYL-COA[c]'
'CPD-22005[c]'
'CPD-22004[c]'
'CPD-22006[c]'
'CPD-22003[c]'
'CPD-22008[c]'
'CPD-22009[c]'
'3-MERCAPTO-PYRUVATE[c]'
'TETRADEHYDROACYL-COA[c]'
'2-hydroxyacyl-glutathiones[c]'
'CPD-22010[c]'
'CPD-22007[c]'
'3S-CITRYL-COA[c]'
'CPD-22012[c]'
'CPD-22013[c]'
'CPD-7221[c]'
'CPD-7222[c]'
'CPD-22014[c]'
'B-KETOACYL-ACP[c]'
'OH-ACYL-ACP[c]'
'CPD-19163[c]'
'CPD-19159[c]'
'CYSTINE[c]'
'THIOCYSTEINE[c]'
'Sulfur-Carrier-Proteins-ThiI[c]'
'Sulfurylated-ThiI[c]'
'CPD-12015[c]'
'CPD-12017[c]'
'BCAA-dehydrogenase-2MP-DH-lipoyl[c]'
'ISOPENICILLIN-N[c]'
'CPD-22011[c]'
'CPD-22016[c]'
'CPD-22017[c]'
'CPD-22018[c]'
'CPD-22015[c]'
'CPD-22021[c]'
'PENICILLIN-N[c]'
'S-3-HYDROXYBUTANOYL-COA[c]'
'CPD-650[c]'
'CPD-19160[c]'
'Acetoacetyl-ACPs[c]'
'CPD-10269[c]'
'CPD-19162[c]'
'Beta-3-hydroxybutyryl-ACPs[c]'
'CPD-15244[c]'
'Alkanesulfonates[c]'
'CPD-18346[c]'
'CPD-15365[c]'
'CPD-17401[c]'
'CPD-22022[c]'
'CPD-22023[c]'
'CPD-22020[c]'
'S-2-METHYLACYL-COA[c]'
'R-2-METHYLACYL-COA[c]'
'CPD-1063[c]'
'Cis-delta-3-decenoyl-ACPs[c]'
'Crotonyl-ACPs[c]'
'Saturated-Fatty-Acyl-ACPs[c]'
'CPD-19158[c]'
'CPD-19155[c]'
'CPD-19147[c]'
'CPD-17402[c]'
'CPD-17403[c]'
'CPD-15367[c]'
'3-oxo-arachidoyl-ACPs[c]'
'Arachidoyl-ACPs[c]'
'trans-delta2-arachidoyl-ACPs[c]'
'cis-cis-19-31-dicyclopropyl-C52-ACPs[c]'
'LIPOYL-AMP[c]'
'PARATHION[c]'
'DIETHYLTHIOPHOSPHATE[c]'
'CPD-514[c]'
'CPD0-2171[c]'
'CPD0-2105[c]'
'CPD-14916[c]'
'CPD0-2106[c]'
'CPD0-2108[c]'
'CPD-17866[c]'
'CPD-867[c]'
'CPD-868[c]'
'TRANS-D2-ENOYL-ACP[c]'
'CPD-17346[c]'
'CPD-17347[c]'
'CPD-17348[c]'
'CPD1G-120[c]'
'CPD-12646[c]'
'cis-19-CP-37-Mex-38-Me-C59-ACPs[c]'
'Aryl-sulfates[c]'
'CPD0-2121[c]'
'CPD1G-332[c]'
'S-Substituted-Glutathione[c]'
'R-3-hydroxy-cis-vaccenoyl-ACPs[c]'
'S-Substituted-Glutathione[e]'
'ACRYLYL-COA[c]'
'CPD-19953[c]'
'CPD-20[c]'
'CPD-19953[e]'
'DEACETOXYCEPHALOSPORIN-C[c]'
'CPD-17324[c]'
'CPD-17367[c]'
'CPD-17368[c]'
'CPD-17323[c]'
'CPD-71[c]'
'CPD-7275[c]'
'Protein-S-Acyl-Cysteines[c]'
'3-Oxoacyl-CoAs[c]'
'Protein-S-Acetyl-Cysteines[c]'
'2-HYDROXY-3-KETO-5-METHYLTHIO-1-PHOSPHOP[c]'
'holo-VibB[c]'
'b-Keto-cis-D5-dodecenoyl-ACPs[c]'
'CPD-17371[c]'
'CPD-12173[c]'
'ISOBUTYRYL-COA[c]'
'CPD-21754[c]'
'b-Hydroxy-cis-D5-dodecenoyl-ACPs[c]'
'Trans-D3-cis-D5-dodecenoyl-ACPs[c]'
'CPD-17382[c]'
'CPD-17332[c]'
'CPD-17383[c]'
'cis-cis-D19-31-C50-2-ACPs[c]'
'3-oxo-behenoyl-ACPs[c]'
'Oxidized-Putidaredoxins[c]'
'Reduced-Putidaredoxins[c]'
'R-3-hydroxybehenoyl-ACPs[c]'
'2-trans-4-cis-dienoyl-CoAs[c]'
'PENTANOYLCOA-CPD[c]'
'trans-delta2-behenoyl-ACPs[c]'
'CPD-17331[c]'
'CPD-17385[c]'
'3-METHYL-CROTONYL-COA[c]'
'CPD-19029[c]'
'CPD-19031[c]'
'CPD-19032[c]'
'CPD-14394[c]'
'Very-Long-Chain-oxoacyl-CoAs[c]'
'Very-Long-Chain-3-Hydroxyacyl-CoAs[c]'
'3-HYDROXY-3-4-METHYLPENT-3-EN-1-YLG-COA[c]'
'CPD-12897[c]'
'CPD-7243[c]'
'CPD-14405[c]'
'CPD-14406[c]'
'CPD-17313[c]'
'Reduced-Cys2-Peroxiredoxins[c]'
'2Cys-Peroxiredoxins-With-HydroxyCys[c]'
'Cys2-Peroxiredoxin-Disulfide[c]'
'Medium-Chain-Acyl-CoAs[c]'
'Medium-Chain-234-Saturated-acyl-CoAs[c]'
'CPD-7036[c]'
'dihomogammalinolenoyl-acp[c]'
'CPD-14419[c]'
'CPD-14420[c]'
'CPD-12647[c]'
'CPD-206[c]'
'2-HYDROXYPHYTANOYL-COA[c]'
'DEACETYLCEPHALOSPORIN-C[c]'
'cis-cis-D19-37-C56-2-ACPs[c]'
'cis-D19-37-OH-38-Me-C57-1-ACPs[c]'
'ESTRONE-SULFATE[c]'
'Ubiquitin-activating-protein-E1-L-cys[c]'
'Geranylgeranylated-XXCC-proteins[c]'
'CPD-7037[c]'
'3-HYDROXY-DOCOSAPENTAENOYL-ACP[c]'
'3-OXO-EICOSAPENTAENOYL-ACP[c]'
'3-HYDROXY-3-METHYL-GLUTARYL-COA[c]'
'CPD-367[c]'
'CPD-380[c]'
'cis-D19-37-MOH-38-Me-C57-1-ACPs[c]'
'R-3-hydroxyarachidoyl-ACPs[c]'
'trans-D18-37-OH-38-Me-C58-1-ACPs[c]'
'trans-D18-37-MOH-38-Me-C58-1-ACPs[c]'
'trans-18-CP-37-Mex-38-Me-C60-ACPs[c]'
'3-HYDROXY-ISOVALERYL-COA[c]'
'CPD-10284[c]'
'XXCC-proteins[c]'
'Protein-Cysteine-Hemithioacetal[c]'
'XCXC-proteins[c]'
'Geranylgeranylated-XCXC-proteins[c]'
'CPD-13691[c]'
'CPD-19066[c]'
'CCXX-proteins[c]'
'Geranylgeranylated-CCXX-proteins[c]'
'CINNAMOYL-COA[c]'
'Oxo-glutarate-dehydro-suc-DH-lipoyl[c]'
'DOCOSAPENTAENOYL-ACP[c]'
'cis-cis-D21-39-C58-2-ACPs[c]'
'cis-D21-39-OH-40-Me-C59-1-ACPs[c]'
'cis-D21-39-oxo-40-Me-C59-1-ACPs[c]'
'cis-21-CP-39-keto-40-Me-C60-ACPs[c]'
'trans-D20-39-oxo-40-Me-C60-1-ACPs[c]'
'trans-20-CP-22-Me-39-keto-40-Me-C61-ACPs[c]'
'SULFOQUINOVOSYLDIACYLGLYCEROL[c]'
'CPD-21685[c]'
'CPD-19111[c]'
'CPD0-2232[c]'
'CPD0-2117[c]'
'4-SULFOBENZYL-ALCOHOL[c]'
'S-ubiquitinyl-UAP-E1-L-cysteine[c]'
'CPD-21345[c]'
'TRANS-3-METHYL-GLUTACONYL-COA[c]'
'CPD-13109[c]'
'2-AMINOBENZOYL-COA[c]'
'2-AMINO-5-OXOCYCLOHEX-1-ENECARBOXYL-COA[c]'
'CPD-17262[c]'
'CPD-17263[c]'
'CPD-17264[c]'
'CPD-5662[c]'
'CPD-21416[c]'
'CPD-21415[c]'
'CPD-21417[c]'
'CPD-21418[c]'
'Cis-2-enoyl-CoAs[c]'
'Oxidized-cytochromes-c553[c]'
'Reduced-cytochromes-c553[c]'
'CPD-211[c]'
'3-SULFOCATECHOL[c]'
'2-AMINOBENZENESULFONATE[c]'
'CPD-12565[c]'
'CPD-21419[c]'
'CPD-21420[c]'
'BIOTIN[e]'
'4-DEOXY-BETA-D-GLUC-4-ENURONOSYL-6S[c]'
'CPD-7672[c]'
'Farnesylated-CAAX-proteins[c]'
'CAAX-proteins[c]'
'L-SELENOCYSTEINE[c]'
'SE-2[c]'
'CPD-10420[c]'
'CPD-207[c]'
'CPD-17328[c]'
'CPD-18492[c]'
'CPD-18493[c]'
'CPD-17365[c]'
'CPD-18494[c]'
'CPD-8876[c]'
'Acetoacetyl-ACP[c]'
'CPD-21199[c]'
'CPD-21199[e]'
'CPD-21162[c]'
'Long-Chain-3S-Hydroxyacyl-CoAs[c]'
'CPD-21201[c]'
'CPD-21202[c]'
'Long-Chain-oxoacyl-CoAs[c]'
'S-METHYLGLUTATHIONE[c]'
'CPD-7670[c]'
'CPD-10472[c]'
'SS-DIMETHYL-BETA-PROPIOTHETIN[c]'
'CPD-9771[c]'
'CPD-9772[c]'
'CPD-21210[c]'
'CPD-21212[c]'
'Oxidized-CycA1-cytochromes[e]'
'Reduced-CycA1-cytochromes[e]'
'LIPOIC-ACID[c]'
'CIS-DELTA3-ENOYL-COA[c]'
'Actinorhodin-Intermediate-2[c]'
'Hepta-oxo-hexadecanoyl-ACPs[c]'
'Aryl-Carrier-Proteins[c]'
'QXC-ACP[c]'
'AcDMPT-L-Alanyl-PhsB[c]'
'PhsC[c]'
'CPD-21241[c]'
'Reduced-cytochromes-c551[e]'
'CPD-21134[c]'
'Oxidized-cytochromes-c551[e]'
'Holo-EntB[c]'
'CPD-10505[c]'
'Red-Glutaredoxins[c]'
'Ox-Glutaredoxins[c]'
'AcDMPT-L-Alanyl-L-Alanyl-PhsC[c]'
'PhsB[c]'
'AcDMPT-L-Alanyl-L-Leucyl-PhsC[c]'
'Persulfurated-L-cysteine-desulfurases[c]'
'L-Cysteine-Desulfurases[c]'
'2-R-HYDROXYPROPYL-COM[c]'
'CPD-10588[c]'
'CPD-72[c]'
'CPD-10589[c]'
'CPD-10590[c]'
'CPD-10591[c]'
'CPD-601[c]'
'S-Alkyl-L-Cysteines[c]'
'Alkyl-Thiols[c]'
'AMINO-PARATHION[c]'
'CPD-21344[c]'
'Holo-EntF[c]'
'CPD-17136[c]'
'All-holo-ACPs[c]'
'Cytochromes-c[c]'
'HEME\_C[c]'
'CPD-11665[c]'
'CPD-21118[c]'
'CPD-21110[c]'
'SELENATE[c]'
'CPD-13713[c]'
'CPD-21115[c]'
'CPD-15683[c]'
'6-AMINOPENICILLANATE[c]'
'6-AMINOPENICILLANATE[e]'
'CPDQT-281[c]'
'CPDQT-280[c]'
'CPDQT-284[c]'
'CPDQT-420[c]'
'CPDQT-288[c]'
'CPDQT-295[c]'
'CPDQT-296[c]'
'CPDQT-299[c]'
'CPDQT-286[c]'
'CPD-15635[c]'
'CPD-17046[c]'
'CPD-17047[c]'
'CPD-17048[c]'
'CPD-17049[c]'
'CPD-14723[c]'
'CPD-17050[c]'
'CPD-17051[c]'
'CPD-678[c]'
'CPD-14724[c]'
'PENICILLIN-N[e]'
'CPD-13717[c]'
'SELENOHOMOCYSTEINE[c]'
'CPD-21122[c]'
'CPD-21116[c]'
'CPD-21126[c]'
'CPD-21128[c]'
'CPD-9300[c]'
'CPD-10467[c]'
'CPD-21160[c]'
'CPD-21166[c]'
'CPDQT-300[c]'
'CPDQT-340[c]'
'Iron-Sulfur-Cluster-Scaffold-Proteins[c]'
'CPD-7046[c]'
'CPDQT-429[c]'
'CPD-13006[c]'
'GLUCOTROPEOLIN[c]'
'CPD-16923[c]'
'CPD-14432[c]'
'Lovastatin-nonaketide-synthase[c]'
'2-methylbutanoyl-LovF[c]'
'Holo-LovF[c]'
'CPD-7302[c]'
'RBR-Ubiquitin-carrier-protein-E3-L-cys[c]'
'Ubiquitin-carrier-protein-E2-L-cysteine[c]'
'S-ubiquitinyl-UCP-RBR-E3-L-cysteine[c]'
'SELENOMETHIONINE[c]'
'CPD-13754[c]'
'CPD-13755[c]'
'CPDQT-434[c]'
'CPD-21172[c]'
'CPD-21171[c]'
'CPD-21170[c]'
'S-ubiquitinyl-UCP-E2-L-cysteine[c]'
'CPD-21169[c]'
'Citrate-Lyase-Citryl-Form[c]'
'CITRATE-LYASE[c]'
'CARBOXYMETHYL-HYDROXYPHENYLPROPCOA[c]'
'E-PHENYLITACONYL-COA[c]'
'CPD-65[c]'
'CPD0-1720[c]'
'CPD-16950[c]'
'CPD-1776[c]'
'CPD-11674[c]'
'PENICILLIN-G[c]'
'4-MHA-THR-VAL-AcmD-Proteins[c]'
'4-HMA-AcmD-Proteins[c]'
'CPD-21178[c]'
'CPD-7547[c]'
'CPD-21179[c]'
'CPD-20053[c]'
'N-5S-5-AMINO-5-CARBOXYPENTANOYL-L-CY[e]'
'CPD-264[c]'
'PENICILLIN-G[e]'
'2-METHYL-ACETO-ACETYL-COA[c]'
'BENZOYLSUCCINYL-COA[c]'
'ISOPENICILLIN-N[e]'
'CPD-21192[c]'
'CPD-21194[c]'
'CYSTINE[e]'
'CPD-9122[e]'
'CPD-9196[e]'
'CPD-21195[c]'
'CPD-21107[c]'
'CPD-13757[c]'
'CPD-13758[c]'
'CPD-9196[c]'
'Long-Chain-Acyl-ACPs[c]'
'CPD-17329[c]'
'CPD-21411[c]'
'CPD-18489[c]'
'CPD-18490[c]'
'CPD-19219[c]'
'CPD-19221[c]'
'CPDQT-35[c]'
'CPDQT-41[c]'
'CPD0-2107[c]'
'CPDQT-27[c]'
'CPDQT-28[c]'
'CPDQT-404[c]'
'CPDQT-29[c]'
'CPDQT-349[c]'
'CPDQT-30[c]'
'CPDQT-256[c]'
'CPDQT-277[c]'
'CPD-6262[c]'
'Palmitoleoyl-ACPs[c]'
'Cis-vaccenoyl-ACPs[c]'
'CPDQT-400[c]'
'CPDQT-341[c]'
'CPDQT-343[c]'
'CPDQT-405[c]'
'N-terminal-specific-UCP-E2-L-cysteine[c]'
'S-ubi-N-term-specific-UCP-E2-L-cysteine[c]'
'METHYL-MALONYL-COA[c]'
'BCAA-dehydrogenase-2MB-DH-lipoyl[c]'
'CPD-12896[c]'
'CPD-1827[c]'
'CPD-12902[c]'
'CPD-15650[c]'
'BIO-5-AMP[c]'
'CPD-6402[c]'
'CPD-6401[c]'
'CPD-16817[c]'
'CPD-16819[c]'
'CPD-16825[c]'
'CPDQT-350[c]'
'CPDQT-262[c]'
'CPDQT-406[c]'
'CPDQT-407[c]'
'CPD-543[c]'
'CPD-9122[c]'
'CPD-12904[c]'
'CPD-12905[c]'
'CPD-12906[c]'
'CPDQT-408[c]'
'CPDQT-409[c]'
'CPDQT-410[c]'
'CPDQT-411[c]'
'CPDQT-412[c]'
'CPDQT-413[c]'
'CPD-13692[c]'
'CPD-13694[c]'
'CPD-13695[c]'
'CPD-13696[c]'
'CPD0-2253[c]'
'CPD-18195[c]'
'holo-Peptidyl-carrier-proteins[c]'
'CPD-14205[c]'
'CPD-627[c]'
'CPDQT-414[c]'
'CPDQT-415[c]'
'CPDQT-416[c]'
'CPDQT-285[c]'
'CPDQT-417[c]'
'CPDQT-418[c]'
'CPD-16867[c]'
'CPD-16868[c]'
'CPD-296[c]'
'CPD-13698[c]'
'CPD-13699[c]'
'CPD-13700[c]'
'Alpha-factor[c]'
'Alpha-factor[e]'
'Holo-Aryl-Carrier-Proteins[c]'
'CPD-21078[c]'
'CPD-21108[c]'
'CPD-21114[c]'
'CPD-21113[c]'
'CPD-1137[c]'
'CPD-17414[c]'
'CPDQT-419[c]'
'CPD-7649[c]'
'CPD-20681[c]'
'CPD-20682[c]'
'CPD-942[c]'
'CPD-943[c]'
'CPD-944[c]'
'N-formyl-L-methionyl-tRNAfmet[c]'
'CPD-18240[c]'
'CPD-18265[c]'
'CPD-18209[c]'
'CPD-18266[c]'
'CPD-21109[c]'
'CPD-21119[c]'
'CPD-21120[c]'
'CPD-21111[c]'
'CPD-20684[c]'
'CPD-21121[c]'
'2-ALPHA-HYDROXYETHYL-THPP[c]'
'CPD-921[c]'
'CPD-8973[c]'
'CPD-8974[c]'
'METHACRYLYL-COA[c]'
'ALBENDAZOLE[c]'
'ALBENDAZOLE-S-OXIDE[c]'
'CPDQT-31[c]'
'GLUTATHIONYLSPERMIDINE[c]'
'S-24-DINITROPHENYLGLUTATHIONE[c]'
'CPD-13390[c]'
'CPD-18199[c]'
'3-HYDROXY-PROPIONYL-COA[c]'
'CPDQT-32[c]'
'CPDQT-33[c]'
'HECT-Ubiquitin-carrier-protein-E3-L-cys[c]'
'S-ubiquitinyl-HECT-E3-UCP-L-cysteine[c]'
'CPD0-2511[c]'
'CPDQT-34[c]'
'CPD-11525[c]'
'CPD-11526[c]'
'CPD-11529[c]'
'CPD-17948[c]'
'CPD-18198[c]'
'CPD-17940[c]'
'S-LACTOYL-GLUTATHIONE[c]'
'R-3-hydroxystearoyl-ACPs[c]'
'CPD-12634[c]'
'CPD-12635[c]'
'CPD-12645[c]'
'Guanidinoacetyl-PKS[c]'
'Aminomalonyl-PCP[c]'
'Loaded-GdnH[c]'
'holo-Seryl-Carrier-Proteins[c]'
'CPD-10246[c]'
'BENZYLSUCCINYL-COA[c]'
'CPD-15035[c]'
'CPD-15036[c]'
'BENZYL-DESULFOGLUCOSINOLATE[c]'
'CPD-20305[c]'
'CPD-11799[c]'
'CPD-13576[c]'
'Oxidized-Disulfide-Carrier-Proteins[c]'
'Reduced-Disulfide-Carrier-Proteins[c]'
'CPD-12692[c]'
'medium-Chain-Trans-23-Dehydroacyl-CoA[c]'
'CPD0-1958[c]'
'CPD-9612[c]'
'CPD-15301[c]'
'CPD-3744[c]'
'CPD-13393[c]'
'Protein-L-methionine-S-S-oxides[c]'
'endothelin-1[c]'
'Charged-MET-tRNAs[c]'
'CPD-11519[c]'
'CPD-11518[c]'
'CPD-11520[c]'
'CPD-13533[c]'
'T2-C4-DECADIENYL-COA[c]'
'S-NEDD8-yl-NEDD8-E2-L-cysteine[c]'
'NEDD8-conjugating-proteins-E2-L-cys[c]'
'CPD0-1306[c]'
'CPD-20047[c]'
'S-ACETYLDIHYDROLIPOAMIDE[c]'
'CPD-18059[c]'
'CPD-564[c]'
'CPD-13534[c]'
'CPD-14729[c]'
'CPD-14772[c]'
'K-HEXANOYL-COA[c]'
'CPD-14928[c]'
'CPD-11521[c]'
'Dermatan-sulfate-L-IdoA2S[c]'
'Pyruvate-dehydrogenase-dihydrolipoate[c]'
'Keratan-sulfate-NAcGlcN6S[c]'
'CPD-11524[c]'
'Pyruvate-dehydrogenase-acetylDHlipoyl[c]'
'Holo-LYS2-peptidyl-carrier-protein[c]'
'2-Aminoadipyl-LYS2[c]'
'CPD-21867[c]'
'Rifamycin-Undecaketide-Acps[c]'
'Protein-With-N-Terminal-Met[c]'
'2-Me-Branched-234-Sat-Fatty-Acyl-CoA[c]'
'CPD-14675[c]'
'OH-HEXANOYL-COA[c]'
'PHOTINUS-LUCIFERIN[c]'
'CPD-20211[c]'
'CPD-18078[c]'
'CPD-11528[c]'
'CPD-11523[c]'
'Dermatan-Sulfate[c]'
'Dermatan-sulfate-disaccharides[c]'
'CEPHALOSPORIN-C[c]'
'CPD-7257[c]'
'CPD0-1885[c]'
'2-MERCAPTOETHANOL[c]'
'tRNA-containing-5-taurinomethyluridine[c]'
'CPD-11527[c]'
'CPD-11522[c]'
'Chondroitin-sulfates[c]'
'Chondroitin-sulfate-disaccharides[c]'
'CPD-12581[c]'
'3-oxo-cis-vaccenoyl-ACPs[c]'
'CPD-19761[c]'
'Glutaryl-ACP-methyl-esters[c]'
'CPD-19763[c]'
'3-Ketopimeloyl-ACP-methyl-esters[c]'
'Petroselinoyl-ACPs[c]'
'3-oxo-cis-D7-tetradecenoyl-ACPs[c]'
'3-hydroxy-cis-D7-tetraecenoyl-ACPs[c]'
'DIMETHYLSULFONIOACETATE[c]'
'S-METHYLTHIOGLYCOLATE[c]'
'Delta4-hexadecenoyl-ACPs[c]'
'3-oxo-petroselinoyl-ACPs[c]'
'CPD-511[c]'
'R-3-hydroxypetroselinoyl-ACPs[c]'
'3-hydroxypimeloyl-ACP-methyl-esters[c]'
'Trans-D2-cis-D7-tetradecenoyl-ACPs[c]'
'Cis-Delta7-tetradecenoyl-ACPs[c]'
'3-oxo-cis-D9-hexadecenoyl-ACPs[c]'
'3-HYDROXY-ISOBUTYRYL-COA[c]'
'Enoylpimeloyl-ACP-methyl-esters[c]'
'Pimeloyl-ACP-methyl-esters[c]'
'Pimeloyl-ACPs[c]'
'cis-vaccen-2-enoyl-ACPs[c]'
'3-Methyl-Saturated-Fatty-Acyl-CoA[c]'
'2-OH-3-Methyl-Saturated-Fatty-Acyl-CoA[c]'
'3-hydroxy-cis-D9-hexaecenoyl-ACPs[c]'
'Trans-D3-cis-D9-hexadecenoyl-ACPs[c]'
'CPD-14746[c]'
'CPD-14756[c]'
'CPD-8132[c]'
'CPD-14757[c]'
'CPD-14758[c]'
'CPD-14760[c]'
'Oxidized-NapC-proteins[e]'
'Reduced-NapC-proteins[e]'
'cis-5-enoyl-CoA[c]'
'trans-2-cis-5-dienoyl-CoA[c]'
'trans-3-cis-5-dienoyl-CoA[c]'
'4812-TRIMETHYLTRIDECANOYL-COA[c]'
'3-OXOPRISTANOYL-COA[c]'
'CPD-17983[c]'
'CPD-17997[c]'
'Holo-AsbD-Proteins[c]'
'CPD-11517[c]'
'an-oxidized-NrfB-protein[e]'
'a-reduced-NrfB-protein[e]'
'SAMP-C-Terminal-thiol[c]'
'CPD-19971[c]'
'TUM1-L-cysteine[c]'
'TUM1-S-sulfanylcysteine[c]'
'Ansamycin-PKS-ACP-Domain[c]'
'Amino-Hydroxybenzoyl-Acps[c]'
'Amino-Hydroxybenzoyl-Rifamycin-PKS[c]'
'Rifamycin-Tetraketide-Acps[c]'
'CPD-14404[c]'
'Holo-SfmC-peptidyl-carrier-protein[c]'
'3O-4-dimethyl-5-OH-L-tyr-SfmC[c]'
'Acyl-alanylglycinyl-SfmB[c]'
'Holo-SfmB-peptidyl-carrier-protein[c]'
'CPD-18035[c]'

-------------------------------------------------------------------------------

The chemical element **Se** is present in the empirical formula of **8 metabolites**

'L-SELENOCYSTEINE[c]'
'SE-2[c]'
'SELENATE[c]'
'CPD-13713[c]'
'CPD-678[c]'
'CPD-13717[c]'
'SELENOHOMOCYSTEINE[c]'
'SELENOMETHIONINE[c]'

-------------------------------------------------------------------------------

The chemical element **Zn** is present in the empirical formula of **2 metabolites**

'ZN+2[e]'
'ZN+2[c]'

-------------------------------------------------------------------------------

fprintf('The following <strong>%d</strong> compounds have no empirical formula:\n\n%s',...

length(iPrub22\_reconstruction.mets(cellfun('isempty',iPrub22\_reconstruction.metFormulas))),...

strjoin(iPrub22\_reconstruction.mets(cellfun('isempty',iPrub22\_reconstruction.metFormulas)),'\n'))

The following **192** compounds have no empirical formula:
Starch[c]
Large-branched-glucans[c]
Long-linear-glucans[c]
Acceptor[c]
Poly-D-galactosamine[c]
Oligomers-Of-Galactosamines[c]
Oligosaccharides[c]
Methylated-Ribosomal-Protein-L11s[c]
Nonmethylated-Ribosomal-Protein-L11s[c]
MPP-processed-mitochonrial-proteins[c]
Processed-Mitochondrial-Proteins[c]
Sialyloligosaccharides[c]
Benzosemiquinones[c]
Glucans[c]
Polynucleotide-Holder[c]
5-Phosphomononucleotides[c]
5-phosphooligonucleotides[c]
Unspecified-Degradation-Products[c]
CPD0-2354[c]
Tau-proteins[c]
O-phospho-tau-proteins[c]
Palmitoyl-proteins[c]
DNA-Combined-With-Exogenous-DNA[c]
Resolution-of-Recombinational-Junction[c]
mature-tRNA[c]
RNASE-III-MRNA-PROCESSING-SUBSTRATE[c]
Starch[e]
RNASE-III-PROCESSING-PRODUCT-MRNA[c]
mRNA-Fragments[c]
CPD0-2352[c]
CPD0-2353[c]
tRNA-fragment[c]
tRNA-Introns[c]
Halide-Anions[c]
Benzenediols[c]
dicarboxylate[c]
D-Hexoses[e]
dicarboxylate[e]
Alpha-tubulins[c]
holo-Transcarboxylases[c]
Polysaccharides[c]
Cutins[c]
Cutin-Monomers[c]
RNASE-II-DEGRADATION-SUBSTRATE-MRNA[c]
mRNAs-With-PolyA-Tails[c]
mRNAs[c]
Mannans[e]
Proteins-with-incorrect-disulfides[c]
Proteins-with-correct-disulfides[c]
Negatively-super-coiled-DNAs[c]
Relaxed-DNAs[c]
Double-Stranded-DNAs[c]
Unwound-RNA[c]
Supercoiled-Duplex-DNAs[c]
Sugar[c]
Sugar-Phosphate[c]
COF[c]
Large-peptides[c]
Small-peptides[c]
CPD0-2351[c]
Detyrosinated-alpha--tubulins[c]
DNA-directed-RNA-polymerases[c]
Phospho-DNA-directed-RNA-polymerases[c]
Soluble-Heteroglycans[c]
DNA-with-mismatch[c]
Damaged-DNA-Pyrimidine[c]
Alpha-Amyloses[c]
S-CD-Apo-SP-Complex[c]
CD-S-SP-Complex[c]
S-CD-S-SP-Complex[c]
MG-PROTOPORPHYRIN-MONOMETHYL-ESTER[c]
DIVINYL-PROTOCHLOROPHYLLIDE-A[c]
CD-2S-SP-Complex[c]
CD-SP-2Fe2S-Complex[c]
Chap-ADP-apo-SP-Complex[c]
Apo-FeS-cluster-proteins[c]
FeS-Cluster-Co-Chaperones[c]
2Fe-2S-proteins[c]
Chap-ATP-Co-chaperone-SP-2Fe2S-Complex[c]
D-form-FeS-Cluster-Scaffold-Proteins[c]
FeS-Cluster-Chaperones-ATP[c]
Lipoprotein-signal-peptide[c]
Wound-RNA[c]
CPD-15015[c]
Myo-inositol-monophosphates[c]
Intracellular-Sulfur[c]
MG-PROTOPORPHYRIN[c]
Biomass[c]
Biomass[e]
Reduced-hemoproteins[c]
Oxidized-hemoproteins[c]
N-acetylarylamines[c]
CHLOROPHYLLIDE-A[c]
CHLOROPHYLL-A[c]
D-Hexoses[c]
ADP-D-Ribosyl-Acceptors[c]
CPD-7015[c]
CPD-19759[c]
CPD0-2340[c]
Cyclodextrins[c]
PROTEIN[c]
RNA[c]
DNA[c]
Protein-phospho-L-histidines[c]
AAPOOL[c]
PLIPIDS[c]
Sugar[e]
CPD-8579[c]
Apocytochromes-c[c]
Myosin-Actin[c]
Dynein-Microtubles-Complex[c]
MUTATED-TRNA[c]
CPD0-2227[c]
Tubulin-Heterodimers[c]
Microtubules[c]
Kinesin-Microtubules-Complex[c]
Fe4S4-Cluster-Protein[c]
Membrane-Compartments[c]
PHOSPHORHODOPSIN[c]
Beta-adrenergic-receptors-P[c]
G-protein-coupled-receptors[c]
Phos-G-protein-coupled-receptors[c]
Phosphorylase-b[c]
Phosphorylase-a[c]
CELLWALL[c]
Branched-chain-2-keto-acid-deHase[c]
Branched-chain-2-keto-acid-deH-P[c]
Tyrosine-3-monooxygenases[c]
Phosphorylated-tyrosine-3-monooxygenases[c]
Pyrophosphate-inositol-phosphates[c]
Myo-inositol-polyphosphates[c]
Sugar-alcohols[c]
Mannans[c]
Polyamines[c]
Polyamines[e]
Xenobiotic[c]
Xenobiotic[e]
Di-trans-poly-cis-polyprenyl-PP[c]
3-H-3-M-GLUTARYL-COA-REDUCTASES[c]
3-H-3-M-GLUTARYL-COA-REDUCTASES-P[c]
Hexadecenoates[c]
CPD0-2186[c]
Adjacent-pyrimidine-dimer-in-DNA[c]
DNA-Adjacent-Pyrimidines[c]
Nonadjacent-pyrimidine-dimer-in-DNA[c]
RX[c]
Monoacylglycerols[c]
Chlorophylls[c]
Chlorophyllides[c]
RNASE-R-DEGRADATION-SUBSTRATE-RNA[c]
Alpha-D-aldose-1-phosphates[c]
Type-1-transmemberane-domains[c]
Cleaved-type-1-transmembrane-domains[c]
CRPB-all-trans-Retinol[c]
CRPB-all-trans-Retinal[c]
Peptides-with-Leader-Sequence[c]
Leader-Sequences[c]
SUMO-propeptides[c]
SUMO-peptides[c]
HEME\_O[c]
Cellular-Retinol-Binding-Proteins[c]
TRANS-POLYISOPRENYL-PP[c]
RAD21-Cohesin-Subunits[c]
Octapeptides[c]
Mitochondrial-Preproteins[c]
big-endothelin[c]
Peptidoglycan-With-Pentaglycine-Bridges[c]
Peptidoglycan-With-Cle-Pentagly-Bridges[c]
Diamines[c]
Alkylated-Bases[c]
Peptidyl-tRNAs[c]
Peptidyl-AminoAcyl-tRNAs[c]
Inositol-Phosphoceramides[c]
MIPC[c]
DMIPC[c]
GDMIPC[c]
DGDMIPC[c]
TGDMIPC[c]
Lysophosphatidylcholines[c]
Lysophosphatidylethanolamines[c]
Sophorosyloxy-FAs-O-6-Diacet[c]
Sophorosyloxy-FAs-O-6-Acet[c]
Sophorosyloxy-Fatty-Acids[c]
RNASE-II-POLY-A-SUBSTRATE-MRNA[c]
RNASE-II-SUBSTRATE-WITH-NO-POLY-A-TAIL[c]
Mitochondrial-tRNA-Pseudouridines-27-28[c]
Mitochondrial-tRNA-Uridines-27-28[c]
Aliphatic-Alpha-Omega-Diamines[c]
Aliphatic-N-Acetyl-Diamines[c]
Fructooligosaccharides[c]
Fructans[c]
a-galctotetrose[c]

- SMILES (▲) and molecular mass

SMILES and molecular weight are encoded in the notes tag.

% Visualisation

for i = 1:5

fprintf('Metabolite name: <strong>%s</strong>\n%s\n\n',...

iPrub22\_reconstruction.metNames{i},iPrub22\_reconstruction.metNotes{i})

end

Metabolite name: **&alpha;-D-glucopyranose 1-phosphate**
mass: 258.119901
smiles: OC[C@H]1O[C@H](OP([O-])([O-])=O)[C@H](O)[C@@H](O)[C@@H]1O
Metabolite name: **&alpha;-D-glucose 6-phosphate**
mass: 258.119901
smiles: O[C@H]1O[C@H](COP([O-])([O-])=O)[C@@H](O)[C@H](O)[C@H]1O
Metabolite name: **2-oleoylglycerol**
mass: 356.5399
smiles: CCCCCCCC\C=C/CCCCCCCC(=O)OC(CO)CO
Metabolite name: **1,2-dioleoylglycerol**
mass: 620.98598
smiles: CCCCCCCC\C=C/CCCCCCCC(=O)OC[C@H](CO)OC(=O)CCCCCCC\C=C/CCCCCCCC
Metabolite name: **coenzyme A**
mass: 763.503643
smiles: CC(C)(COP([O-])(=O)OP([O-])(=O)OC[C@H]1O[C@H]([C@H](O)[C@@H]1OP([O-])([O-])=O)n1cnc2c(N)ncnc12)[C@@H](O)C(=O)NCCC(=O)NCCS

%Sum up

fprintf(['Of the %d metabolites present in the reconstruction:\n\n' ...

' %d have a <strong>molecular weight</strong> (%.1f%%)\n %d have a <strong>SMILES identifier</strong>(%.1f%%)'],...

length(iPrub22\_reconstruction.metNotes),...

sum(count(iPrub22\_reconstruction.metNotes,"mass")),sum(count(iPrub22\_reconstruction.metNotes,"mass"))\*100/length(iPrub22\_reconstruction.metNotes),...

sum(count(iPrub22\_reconstruction.metNotes,"smiles")),sum(count(iPrub22\_reconstruction.metNotes,"smiles"))\*100/length(iPrub22\_reconstruction.metNotes))

Of the 5464 metabolites present in the reconstruction:
5270 have a **molecular weight** (96.4%)
5276 have a **SMILES identifier**(96.6%)

#### 1.2.4 At least one database identifier from a reliable resource (▲)

More cross-references are better because:

1. allows for the unambiguous identification of the entity concerned
2. allows to work more freely with different databases and facilitates exchanges

- BioCyc
- MetanetX
- (ChEBI, PubChem, ChemSpider, CAS)
- (BiGG, KEGG, ModelSEED, SABIO-RK)
- (LIPIDMAPS, SwissLipids, DrugBank, KNApSAcK, MetaboLights, UM-BBD, HMDB)

Metabolites = iPrub22\_reconstruction.mets;

BioCyc = iPrub22\_reconstruction.metBioCycID(~cellfun('isempty',iPrub22\_reconstruction.metBioCycID));

ChEBI = iPrub22\_reconstruction.metChEBIID(~cellfun('isempty',iPrub22\_reconstruction.metChEBIID));

Pubchem = iPrub22\_reconstruction.metPubChemID(~cellfun('isempty',iPrub22\_reconstruction.metPubChemID));

Chemspider = iPrub22\_reconstruction.metischemspiderID(~cellfun('isempty',iPrub22\_reconstruction.metischemspiderID));

MetaNetX = iPrub22\_reconstruction.metMetaNetXID(~cellfun('isempty',iPrub22\_reconstruction.metMetaNetXID));

BiGG = iPrub22\_reconstruction.metBiGGID(~cellfun('isempty',iPrub22\_reconstruction.metBiGGID));

KEGG = iPrub22\_reconstruction.metKEGGID(~cellfun('isempty',iPrub22\_reconstruction.metKEGGID));

KEGG\_drug = iPrub22\_reconstruction.metiskegg\_\_46\_\_drugID(~cellfun('isempty',iPrub22\_reconstruction.metiskegg\_\_46\_\_drugID));

KEGG\_glycan = iPrub22\_reconstruction.metiskegg\_\_46\_\_glycanID(~cellfun('isempty',iPrub22\_reconstruction.metiskegg\_\_46\_\_glycanID));

ModelSEED = iPrub22\_reconstruction.metSEEDID(~cellfun('isempty',iPrub22\_reconstruction.metSEEDID));

LIPIDSMAPS = iPrub22\_reconstruction.metLIPIDMAPSID(~cellfun('isempty',iPrub22\_reconstruction.metLIPIDMAPSID));

SABIORK = iPrub22\_reconstruction.metSABIORKID(~cellfun('isempty',iPrub22\_reconstruction.metSABIORKID));

SwissLipids = iPrub22\_reconstruction.metSLMID(~cellfun('isempty',iPrub22\_reconstruction.metSLMID));

CAS = iPrub22\_reconstruction.metiscasID(~cellfun('isempty',iPrub22\_reconstruction.metiscasID));

DrugBank = iPrub22\_reconstruction.metisdrugbankID(~cellfun('isempty',iPrub22\_reconstruction.metisdrugbankID));

KNApSAcK = iPrub22\_reconstruction.metisknapsackID(~cellfun('isempty',iPrub22\_reconstruction.metisknapsackID));

MetaboLights = iPrub22\_reconstruction.metismetabolightsID(~cellfun('isempty',iPrub22\_reconstruction.metismetabolightsID));

UMBBD = iPrub22\_reconstruction.metisumbbd\_\_46\_\_compoundID(~cellfun('isempty',iPrub22\_reconstruction.metisumbbd\_\_46\_\_compoundID));

HMDB = iPrub22\_reconstruction.metHMDBID(~cellfun('isempty',iPrub22\_reconstruction.metHMDBID));

% Writing table (number of entities and percentage)

Database = ["BioCyc", 'ChEBI','Pubchem','Chemspider','MetaNetX','BiGG','KEGG', 'ModelSEED', 'LIPIDSMAPS','SABIORK','SwissLipids','CAS',...

'DrugBank','KNApSAcK','MetaboLights','UMBBD','HMDB'];

Number\_of\_annotations = [length(BioCyc) round(length(BioCyc)\*100/length(iPrub22\_reconstruction.mets),3,'significant') ;...

length(ChEBI) round(length(ChEBI)\*100/length(iPrub22\_reconstruction.mets),2,'significant') ;...

length(Pubchem) round(length(Pubchem)\*100/length(iPrub22\_reconstruction.mets),2,'significant') ; ...

length(Chemspider) round(length(Chemspider)\*100/length(iPrub22\_reconstruction.mets),2,'significant');...

length(MetaNetX) round(length(MetaNetX)\*100/length(iPrub22\_reconstruction.mets),2,'significant') ; ...

length(BiGG) round(length(BiGG)\*100/length(iPrub22\_reconstruction.mets),2,'significant') ; ...

length(KEGG)+length(KEGG\_drug)+length(KEGG\_glycan) round((length(KEGG)+length(KEGG\_drug)+length(KEGG\_glycan))\*100/length(iPrub22\_reconstruction.mets),2,'significant');...

length(ModelSEED) round(length(ModelSEED)\*100/length(iPrub22\_reconstruction.mets),2,'significant');...

length(LIPIDSMAPS) round(length(LIPIDSMAPS)\*100/length(iPrub22\_reconstruction.mets),2,'significant'); ...

length(SABIORK) round(length(SABIORK)\*100/length(iPrub22\_reconstruction.mets),2,'significant'); ...

length(SwissLipids) round(length(SwissLipids)\*100/length(iPrub22\_reconstruction.mets),2,'significant');...

length(CAS) round(length(CAS)\*100/length(iPrub22\_reconstruction.mets),2,'significant'); ...

length(DrugBank) round(length(DrugBank)\*100/length(iPrub22\_reconstruction.mets),2,'significant'); ...

length(KNApSAcK) round(length(KNApSAcK)\*100/length(iPrub22\_reconstruction.mets),2,'significant');...

length(MetaboLights) round(length(MetaboLights)\*100/length(iPrub22\_reconstruction.mets),2,'significant'); ...

length(UMBBD) round(length(UMBBD)\*100/length(iPrub22\_reconstruction.mets),2,'significant'); ...

length(HMDB) round(length(HMDB)\*100/length(iPrub22\_reconstruction.mets),2,'significant')];

Number\_of\_unique\_annotations = [length(unique(BioCyc)) round(length(unique(BioCyc))\*100/length(unique(iPrub22\_reconstruction.mets)),2,'significant') ;...

length(unique(ChEBI)) round(length(unique(ChEBI))\*100/length(unique(iPrub22\_reconstruction.mets)),2,'significant') ;...

length(unique(Pubchem)) round(length(unique(Pubchem))\*100/length(unique(iPrub22\_reconstruction.mets)),2,'significant');...

length(unique(Chemspider)) round(length(unique(Chemspider))\*100/length(unique(iPrub22\_reconstruction.mets)),2,'significant');...

length(unique(MetaNetX)) round(length(unique(MetaNetX))\*100/length(unique(iPrub22\_reconstruction.mets)),2,'significant');...

length(unique(BiGG)) round(length(unique(BiGG))\*100/length(unique(iPrub22\_reconstruction.mets)),2,'significant');...

length(unique(KEGG))+length(unique(KEGG\_drug))+length(unique(KEGG\_glycan))...

round((length(unique(KEGG))+length(unique(KEGG\_drug))+length(unique(KEGG\_glycan)))\*100/length(unique(iPrub22\_reconstruction.mets)),2,'significant');...

length(unique(ModelSEED)) round(length(unique(ModelSEED))\*100/length(unique(iPrub22\_reconstruction.mets)),2,'significant'); ...

length(unique(LIPIDSMAPS)) round(length(unique(LIPIDSMAPS))\*100/length(unique(iPrub22\_reconstruction.mets)),2,'significant');...

length(unique(SABIORK)) round(length(unique(SABIORK))\*100/length(unique(iPrub22\_reconstruction.mets)),2,'significant'); ...

length(unique(SwissLipids)) round(length(unique(SwissLipids))\*100/length(unique(iPrub22\_reconstruction.mets)),2,'significant');...

length(unique(CAS)) round(length(unique(CAS))\*100/length(unique(iPrub22\_reconstruction.mets)),2,'significant');...

length(unique(DrugBank)) round(length(unique(DrugBank))\*100/length(unique(iPrub22\_reconstruction.mets)),2,'significant');...

length(unique(KNApSAcK)) round(length(unique(KNApSAcK))\*100/length(unique(iPrub22\_reconstruction.mets)),2,'significant');...

length(unique(MetaboLights)) round(length(unique(MetaboLights))\*100/length(unique(iPrub22\_reconstruction.mets)),2,'significant');...

length(unique(UMBBD)) round(length(unique(UMBBD))\*100/length(unique(iPrub22\_reconstruction.mets)),2,'significant');...

length(unique(HMDB)) round(length(unique(HMDB))\*100/length(unique(iPrub22\_reconstruction.mets)),2,'significant')];

disp(table(Number\_of\_annotations, Number\_of\_unique\_annotations, 'RowNames',Database))

**Number\_of\_annotations** **Number\_of\_unique\_annotations**
**\_\_\_\_\_\_\_\_\_\_\_\_\_\_\_\_\_\_\_\_\_** **\_\_\_\_\_\_\_\_\_\_\_\_\_\_\_\_\_\_\_\_\_\_\_\_\_\_\_\_**
**BioCyc**  5441 99.6 5171 95
**ChEBI**  3568 65 3311 61
**Pubchem**  3693 68 3484 64
**Chemspider**  1487 27 1331 24
**MetaNetX**  5436 99 5079 93
**BiGG**  717 13 591 11
**KEGG**  2371 43 2176 40
**ModelSEED**  4 0.073 2 0.037
**LIPIDSMAPS**  138 2.5 136 2.5
**SABIORK**  12 0.22 10 0.18
**SwissLipids**  26 0.48 24 0.44
**CAS**  959 18 816 15
**DrugBank**  237 4.3 202 3.7
**KNApSAcK**  96 1.8 80 1.5
**MetaboLights** 1052 19 909 17
**UMBBD**  50 0.92 45 0.82
**HMDB**  1549 28 1390 25

#### 1.2.5 SBO terms (▲)

fprintf('%d metabolites are annotated with <strong>%s</strong>',...

length(iPrub22\_reconstruction.metSBOTerms(~cellfun('isempty',iPrub22\_reconstruction.metSBOTerms))),...

char(unique(iPrub22\_reconstruction.metSBOTerms)));

5464 metabolites are annotated with **SBO:0000247**

- SBO:0000247 stands for 'simple chemical'

### 1.3. Biochemical reactions

Reactions in iPrub22

- Reactions with a MetaCyc-compatible identifier
- Reactions from iAL1006
- Modelling reactions (artificial) (Transport\_\d{3} - Uptake\_\d{3} - Demand\_\d{3} - Sink\_\d{3} - Production\_\d{3})
- Specific reactions (NGAM - Biomass\_rxn - Transport\_Biomass - Exchange\_Biomass)

The iPrub22 reactions come either from the MetaCyc database (in which case these identifiers are standardised and eligible for enrichment) or from iAL1006 (2013 reconstruction - identifier in the form of r[0-9]{4}).

fprintf(['Number of total reactions: <strong>%d</strong>\n\n' ...

' <strong>%d</strong> reactions with MetaCyc Id\n' ...

' <strong>%d</strong> reactions from iAL1006\n' ...

' <strong>%d</strong> Transport reactions\n' ...

' <strong>%d</strong> Uptake reactions\n' ...

' <strong>%d</strong> Demand reactions\n' ...

' <strong>%d</strong> Sink reactions\n' ...

' <strong>%d</strong> Production reactions\n'...

' <strong>%d</strong> specific reactions\n'],...

length(iPrub22\_reconstruction.rxns) ,...

length(iPrub22\_reconstruction.rxns)-(280+185+37+1+208+42+3),... ### a reprendre la somme fait 5902

sum(~cellfun('isempty',(cellfun(@(x)regexp(x, 'r[0-9]{4}'),iPrub22\_reconstruction.rxns,'UniformOutput',false)))),...

sum(~cellfun('isempty',(cellfun(@(x)regexp(x, 'Transport\_[0-9]{3}'),iPrub22\_reconstruction.rxns,'UniformOutput',false)))),...

sum(~cellfun('isempty',(cellfun(@(x)regexp(x, 'Uptake\_[0-9]{3}'),iPrub22\_reconstruction.rxns,'UniformOutput',false)))),...

sum(~cellfun('isempty',(cellfun(@(x)regexp(x, 'Demand\_[0-9]{3}'),iPrub22\_reconstruction.rxns,'UniformOutput',false)))),...

sum(~cellfun('isempty',(cellfun(@(x)regexp(x, 'Sink\_[0-9]{3}'),iPrub22\_reconstruction.rxns,'UniformOutput',false)))),...

sum(~cellfun('isempty',(cellfun(@(x)regexp(x, 'Production\_[0-9]{3}'),iPrub22\_reconstruction.rxns,'UniformOutput',false)))),...

length({'NGAM','Biomass\_rxn','Exchange\_Biomass'}))

Number of total reactions: **5919**
**5163** reactions with MetaCyc Id
**280** reactions from iAL1006
**208** Transport reactions
**185** Uptake reactions
**37** Demand reactions
**1** Sink reactions
**42** Production reactions
**3** specific reactions

#### 1.3.1 Metadata (▲)

As for the metabolites, to ensure traceability and accessibility of data, the reactions are provided with the following features:

- ID
- Name
- Formulae
- EC number
- GPRs associations
- Reconstruction sources

Each reaction is associated with an identifier, a name and a formula. Most of the identifiers come from the MetaCyc database, and a MetaNetX has been done to enrich these data. Each reaction has a common name describing its nature, or failing that, gene name leading to its catalyses. Enzyme Commission number is s a numerical classification for enzymes based on the chemical reactions they compartmentation. Therefore, not all reactions of a model (i.e. exchange reactions, artificial transport) are bound to be annotated by this type of object. Similarly, not all reactions are linked to GPR associations. Reaction justification in the network (sources) is encoded in the notes tag. ANNOTATION and ORTHOLOGY mean that this reaction is supported by their respective subnetwork. On the other hand, the MANUAL tag may represent data from external sources, gap-filling or manual curations performed.

% Visualisation

formulas = printRxnFormula(iPrub22\_reconstruction,'printFlag',false, 'gprFlag',true) ;

for i = 1:5

% GPRs

genes = regexp(iPrub22\_reconstruction.rules{i},'x\(([0-9]+)\)', 'tokens');

rule = iPrub22\_reconstruction.rules{i} ;

for j = 1:length(genes)

rule = regexprep(rule,'x\([0-9]+\)','${iPrub22\_reconstruction.genes{str2num(char(genes{j}))}}','once') ;

end

rule = regexprep(rule,'\|','or'); rule = regexprep(rule,'&','and'); rule = regexprep(rule,'( ','('); rule = regexprep(rule,' )',')') ;

% Others data

fprintf(['Reaction <strong>ID</strong>: %s\n' ...

'Reaction <strong>Name</strong>: %s\n' ...

'Reaction <strong>Formula</strong>: %s\n' ...

'<strong>Lower bound</strong>: %d\n', ...

'<strong>Upper bound</strong>: %d\n', ...

'<strong>EC number(s)</strong>: %s\n' ...

'<strong>GPR associations</strong>: %s\n',...

'<strong>Source(s)</strong>: %s\n\n'],...

iPrub22\_reconstruction.rxns{i},...

iPrub22\_reconstruction.rxnNames{i},...

formulas{i},...

iPrub22\_reconstruction.lb(i),...

iPrub22\_reconstruction.ub(i),...

iPrub22\_reconstruction.rxnECNumbers{i},...

rule,...

iPrub22\_reconstruction.rxnNotes{i})

end

Reaction **ID**: 1-ACYLGLYCEROL-3-P-ACYLTRANSFER-RXN
Reaction **Name**: Pc12g13010\_product
Reaction **Formula**: ACYL-ACP[c] + ACYL-SN-GLYCEROL-3P[c] -> ACP[c] + L-PHOSPHATIDATE[c]
**Lower bound**: 0
**Upper bound**: 1000
**EC number(s)**: 2.3.1.51
**GPR associations**: (gp\_Pc13g04040 or (gp\_Pc16g02170 and gp\_Pc16g09860 and gp\_Pc16g07520) or gp\_Pc20g00970 or gp\_Pc12g04190 or gp\_Pc16g08280 or gp\_Pc18g02500 or gp\_Pc12g13010)
**Source(s)**: CATEGORIES: ANNOTATION and ORTHOLOGY
Reaction **ID**: 1-PHOSPHATIDYLINOSITOL-3-KINASE-RXN
Reaction **Name**: Pc21g00590\_product
Reaction **Formula**: ATP[c] + L-1-phosphatidyl-inositols[c] -> PROTON[c] + ADP[c] + CPD-177[c]
**Lower bound**: 0
**Upper bound**: 0
**EC number(s)**: 2.7.1.137
**GPR associations**: (gp\_Pc21g00590 or gp\_Pc22g20500)
**Source(s)**: CATEGORIES: ANNOTATION and ORTHOLOGY
Reaction **ID**: 1-PHOSPHATIDYLINOSITOL-KINASE-RXN
Reaction **Name**: Pc12g11590\_product
Reaction **Formula**: ATP[c] + L-1-phosphatidyl-inositols[c] -> PROTON[c] + ADP[c] + CPD-1108[c]
**Lower bound**: 0
**Upper bound**: 0
**EC number(s)**: 2.7.1.67
**GPR associations**: (gp\_Pc20g09090 or gp\_Pc12g11590)
**Source(s)**: CATEGORIES: ANNOTATION and ORTHOLOGY
Reaction **ID**: 1.1.1.117-RXN
Reaction **Name**: D-arabinose 1-dehydrogenase [NAD(P)+]
Reaction **Formula**: NAD-P-OR-NOP[c] + D-arabinofuranose[c] -> PROTON[c] + NADH-P-OR-NOP[c] + CPD-356[c]
**Lower bound**: 0
**Upper bound**: 1000
**EC number(s)**: 1.1.1.117
**GPR associations**: gp\_Pc21g04610
**Source(s)**: CATEGORIES: ANNOTATION
Reaction **ID**: 1.1.1.127-RXN
Reaction **Name**: Pc16g12940\_product
Reaction **Formula**: NAD[c] + 2-DEHYDRO-3-DEOXY-D-GLUCONATE[c] <=> PROTON[c] + NADH[c] + CPD-343[c]
**Lower bound**: -1000
**Upper bound**: 1000
**EC number(s)**: 1.1.1.125; 1.1.1.127
**GPR associations**: gp\_Pc16g12940
**Source(s)**: CATEGORIES: ANNOTATION

%Sum up

fprintf(['Of the <strong>%d</strong> reactions present in the reconstruction:\n\n',...

' %d are <strong>reversible</strong> (%.1f%%)\n',...

' %d have at least one <strong>EC number</strong> associated (%.1f%%)\n',...

' %d are associated with <strong>GPR</strong> (%.1f%%)\n',...

' %d are <strong>sourced</strong> (%.1f%%)'],...

length(iPrub22\_reconstruction.rxns),...

length(find(iPrub22\_reconstruction.lb ~= 0 & iPrub22\_reconstruction.ub ~= 0)),...

length(find(iPrub22\_reconstruction.lb ~= 0 & iPrub22\_reconstruction.ub ~= 0))\*100/length(iPrub22\_reconstruction.rxns),...

sum(~cellfun('isempty',iPrub22\_reconstruction.rxnECNumbers)),sum(~cellfun('isempty',iPrub22\_reconstruction.rxnECNumbers))\*100/length(iPrub22\_reconstruction.rxns),...

sum(~cellfun('isempty',iPrub22\_reconstruction.rules)),sum(~cellfun('isempty',iPrub22\_reconstruction.rules))\*100/length(iPrub22\_reconstruction.rxns),...

sum(~cellfun('isempty',iPrub22\_reconstruction.rxnNotes)),sum(~cellfun('isempty',iPrub22\_reconstruction.rxnNotes))\*100/length(iPrub22\_reconstruction.rxns))

Of the **5919** reactions present in the reconstruction:
603 are **reversible** (10.2%)
4343 have at least one **EC number** associated (73.4%)
5502 are associated with **GPR** (93.0%)
5919 are **sourced** (100.0%)

disp(groupsummary(table(iPrub22\_reconstruction.rxnNotes, 'VariableNames',{'Sources'}),'Sources'))

**Sources** **GroupCount**
**\_\_\_\_\_\_\_\_\_\_\_\_\_\_\_\_\_\_\_\_\_\_\_\_\_\_\_\_\_\_\_\_\_\_\_\_\_\_** **\_\_\_\_\_\_\_\_\_\_**
'CATEGORIES: ANNOTATION' 1151
'CATEGORIES: ANNOTATION and ORTHOLOGY' 2054
'CATEGORIES: MANUAL' 1776
'CATEGORIES: MANUAL and ORTHOLOGY' 7
'CATEGORIES: ORTHOLOGY' 931

#### 1.3.2 At least one database identifier from a reliable resource

More cross-references are better because:

1. allows for the unambiguous identification of the entity concerned
2. allows to work more freely with different databases and facilitates exchanges

- BioCyc
- MetanetX
- (BiGG, KEGG, ModelSEED, Rhea)
- (BRENDA)

BioCyc = iPrub22\_reconstruction.rxnBioCycID(~cellfun('isempty',iPrub22\_reconstruction.rxnBioCycID));

MetanetX = iPrub22\_reconstruction.rxnMetaNetXID(~cellfun('isempty',iPrub22\_reconstruction.rxnMetaNetXID));

BiGG = iPrub22\_reconstruction.rxnBiGGID(~cellfun('isempty',iPrub22\_reconstruction.rxnBiGGID));

KEGG = iPrub22\_reconstruction.rxnKEGGID(~cellfun('isempty',iPrub22\_reconstruction.rxnKEGGID));

Seed = iPrub22\_reconstruction.rxnisseed\_\_46\_\_reactionID(~cellfun('isempty',iPrub22\_reconstruction.rxnisseed\_\_46\_\_reactionID));

Rhea = iPrub22\_reconstruction.rxnRheaID(~cellfun('isempty',iPrub22\_reconstruction.rxnRheaID));

Brenda = iPrub22\_reconstruction.rxnBRENDAID(~cellfun('isempty',iPrub22\_reconstruction.rxnBRENDAID));

% Writing table (number of entities and associated percentage)

Database = ["BioCyc", 'MetaNetX','BiGG','KEGG','Seed','Rhea','Brenda'];

Number\_of\_annotations = [length(BioCyc) round(length(BioCyc)\*100/length(iPrub22\_reconstruction.rxns),2,'significant') ; ...

length(MetaNetX) round(length(MetaNetX)\*100/length(iPrub22\_reconstruction.rxns),2,'significant') ; ...

length(BiGG) round(length(BiGG)\*100/length(iPrub22\_reconstruction.rxns),2,'significant') ; ...

length(KEGG) round(length(KEGG)\*100/length(iPrub22\_reconstruction.rxns),2,'significant') ; ...

length(Seed) round(length(Seed)\*100/length(iPrub22\_reconstruction.rxns),2,'significant') ; ...

length(Rhea) round(length(Rhea)\*100/length(iPrub22\_reconstruction.rxns),2,'significant') ; ...

length(Brenda) round(length(Brenda)\*100/length(iPrub22\_reconstruction.rxns),2,'significant')];

disp(table(Number\_of\_annotations, 'RowNames',Database))

**Number\_of\_annotations**
**\_\_\_\_\_\_\_\_\_\_\_\_\_\_\_\_\_\_\_\_\_**
**BioCyc**  5162 87
**MetaNetX** 5436 92
**BiGG**  4 0.068
**KEGG**  2470 42
**Seed**  1329 22
**Rhea**  2873 49
**Brenda**  4343 73

#### 1.3.3 Balance

iPrub22\_reconstruction = findSExRxnInd(iPrub22\_reconstruction) ;

[~, imBalancedMass, imBalancedCharge, imBalancedRxnBool, ~, missingFormulaeBool, balancedMetBool] = checkMassChargeBalance(iPrub22\_reconstruction);

fprintf(['Number of reactions: %d\n',...

' Reactions with mass imbalance: %d (%.1f%%)\n',...

' Reactions with charge imbalance: %d (%.1f%%)\n',...

' Imbalance reactions (exchange reactions are included): %d (%.1f%%)\n',...

' Number of reactions heuristically though to be mass balanced: %d (%.1f%%)\n\n', ...

'Number of metabolites: %d\n',...

' Metabolites without formulae: %d (%.1f%%)\n',...

' Metabolites exclusively involved in balanced reactions: %d (%.1f%%)\n',...

' Number of metabolites heuristically thought to be involved in mass-balanced reactions: %d (%.1f%%)\n', ...

' Number of metabolites heuristically thought only to be involved in mass balanced reactions: %d (%.1f%%)\n', ...

' Number of metabolites heuristically thought to be involved in mass-imbalanced reactions: %d (%.1f%%)\n', ...

' Number of metabolites heuristically thought only to be involved in mass imbalanced reactions: %d (%.1f%%)\n'], ...

length(iPrub22\_reconstruction.rxns),...

length(imBalancedMass(~cellfun('isempty',imBalancedMass))),length(imBalancedMass(~cellfun('isempty',imBalancedMass)))\*100/length(iPrub22\_reconstruction.rxns),...

sum(imBalancedCharge ~= 0),sum(imBalancedCharge ~= 0)\*100/length(iPrub22\_reconstruction.rxns),...

sum(imBalancedRxnBool),sum(imBalancedRxnBool)\*100/length(iPrub22\_reconstruction.rxns),...

sum(iPrub22\_reconstruction.SIntRxnBool),sum(iPrub22\_reconstruction.SIntRxnBool)\*100/length(iPrub22\_reconstruction.rxns), ...

length(iPrub22\_reconstruction.mets),sum(missingFormulaeBool),sum(missingFormulaeBool)\*100/length(iPrub22\_reconstruction.mets),...

sum(balancedMetBool),sum(balancedMetBool)\*100/length(iPrub22\_reconstruction.mets),...

sum(iPrub22\_reconstruction.SIntMetBool),sum(iPrub22\_reconstruction.SIntMetBool)\*100/length(iPrub22\_reconstruction.mets),...

sum(iPrub22\_reconstruction.SOnlyIntMetBool),sum(iPrub22\_reconstruction.SOnlyIntMetBool)\*100/length(iPrub22\_reconstruction.mets),...

sum(iPrub22\_reconstruction.SExMetBool),sum(iPrub22\_reconstruction.SExMetBool)\*100/length(iPrub22\_reconstruction.mets),...

sum(iPrub22\_reconstruction.SOnlyExMetBool),sum(iPrub22\_reconstruction.SOnlyExMetBool)\*100/length(iPrub22\_reconstruction.mets))

Number of reactions: 5919
Reactions with mass imbalance: 1398 (23.6%)
Reactions with charge imbalance: 969 (16.4%)
Imbalance reactions (exchange reactions are included): 1402 (23.7%)
Number of reactions heuristically though to be mass balanced: 5689 (96.1%)
Number of metabolites: 5464
Metabolites without formulae: 192 (3.5%)
Metabolites exclusively involved in balanced reactions: 3517 (64.4%)
Number of metabolites heuristically thought to be involved in mass-balanced reactions: 5464 (100.0%)
Number of metabolites heuristically thought only to be involved in mass balanced reactions: 5226 (95.6%)
Number of metabolites heuristically thought to be involved in mass-imbalanced reactions: 238 (4.4%)
Number of metabolites heuristically thought only to be involved in mass imbalanced reactions: 0 (0.0%)

#### 1.3.4 SBO terms (▲)

SBO terms for reactions are extremely useful to clearly distinguish a few categories of reactions without having to rely on naming conventions.

- SBO:0000167 stands for 'biochemical or transport reaction'
- SBO:0000176 stands for 'biochemical reaction'
- SBO:0000185 stands for 'translocation reaction'
- SBO:0000627 stands for 'exchange reaction'
- SBO:0000628 stands for 'demand reaction'
- SBO:0000629 stands for 'biomass production'
- SBO:0000630 stands for 'ATP maintenance'
- SBO:0000632 stands for 'sink reaction'
- SBO:0000655 stands for 'transport reaction'
- SBO:0000657 stands for 'active transport' (child term of SBO:0000655)
- SBO:0000658 stands for 'passive transport' (child term of SBO:0000655)
- SBO:0000659 stands for 'symporter-mediated transport' (grandchild term of SBO:0000655)
- SBO:0000660 stands for 'antiporter-mediated transport' (grandchild term of SBO:0000655)
- SBO:0000672 stands for 'spontaneous reaction'

sbo = unique(iPrub22\_reconstruction.rxnSBOTerms);

for i = 1:length(sbo)

fprintf('%d reactions are annotaded with <strong>%s</strong>\n',sum(cellfun(@(x) isequal(x,sbo{i}(:,:)), iPrub22\_reconstruction.rxnSBOTerms)), sbo{i}(:,:));

end

3 reactions are annotaded with **SBO:0000167**
5280 reactions are annotaded with **SBO:0000176**
60 reactions are annotaded with **SBO:0000185**
228 reactions are annotaded with **SBO:0000627**
37 reactions are annotaded with **SBO:0000628**
1 reactions are annotaded with **SBO:0000629**
1 reactions are annotaded with **SBO:0000630**
1 reactions are annotaded with **SBO:0000632**
53 reactions are annotaded with **SBO:0000655**
38 reactions are annotaded with **SBO:0000657**
108 reactions are annotaded with **SBO:0000658**
21 reactions are annotaded with **SBO:0000659**
2 reactions are annotaded with **SBO:0000660**
86 reactions are annotaded with **SBO:0000672**

#### 1.3.5 Model preparation: objective function

The objective function used in iPrub22 is an adaptation of the one in iAL1006 (https://doi.org/10.1371/journal.pcbi.1002980), which was obtained from experimental data.

fprintf(['Reaction ID: <strong>%s</strong>\n\n',...

'Reaction Formula: <strong>%s</strong>\n\n',...

'Objective sense: <strong>%s</strong>\n',...

' Lower bound: <strong>%d</strong>\n',...

' Upper bound: <strong>%d</strong>\n'],...

iPrub22\_reconstruction.rxns{find(iPrub22\_reconstruction.c==1)},...

formulas{find(iPrub22\_reconstruction.c==1)},...

iPrub22\_reconstruction.osenseStr, ...

iPrub22\_reconstruction.lb(find(iPrub22\_reconstruction.c==1)),...

iPrub22\_reconstruction.ub(find(iPrub22\_reconstruction.c==1)))

Reaction ID: **Biomass\_rxn**
Reaction Formula: **104 WATER[c] + 104 ATP[c] + 0.0001 COF[c] + 0.45 PROTEIN[c] + 0.08 RNA[c] + 0.01 DNA[c] + 0.04 AAPOOL[c] + 0.035 PLIPIDS[c] + 0.25 CELLWALL[c] -> 104 ADP[c] + 104 Pi[c] + Biomass[c]** 
Objective sense: **max**
Lower bound: **0**
Upper bound: **1000**

### 1.4. Genes

The gene identifiers for P. chrysogenum Wisconsin 54-1255 are in the form of Pc\d{2}g\d{5} where the first number is the contig number and the second is the gene number. Of the 6,171 genes referenced in the reconstruction, 468 are artificial. They have been added to the reconstruction to differentiate them from gap-filling reactions and to target specific reaction types :

- spontaneous (ID format: s\d{3})
- transport (ID format: t\d{3})
- demand (ID format: d\d{3})
- sink (ID format: sk\d{3})
- uptake (ID format: u\d{3})
- production (ID format: p\d{3})

fprintf(['<strong>Total "Genes" number in the reconstruction:</strong> %d\n\n <strong>Actual genes number:</strong> %d\n ' ...

'<strong>Artificial genes number:</strong> %d\n\n Spontaneous reactions: %d\n Transport reactions: %d\n Demand reactions: %d\n' ...

' Sink reactions: %d\n Uptake reactions: %d\n Production reactions: %d\n'],...

length(iPrub22\_reconstruction.genes),sum(count(iPrub22\_reconstruction.geneNames,"Pc")),length(regexp(strcat(iPrub22\_reconstruction.geneNames{:}),'s|t|d|u|p')),...

sum(count(iPrub22\_reconstruction.geneNames,"s"))-sum(count(iPrub22\_reconstruction.geneNames,"sk")),sum(count(iPrub22\_reconstruction.geneNames,"t")),...

sum(count(iPrub22\_reconstruction.geneNames,"d")),sum(count(iPrub22\_reconstruction.geneNames,"sk")), sum(count(iPrub22\_reconstruction.geneNames,"u")),...

sum(count(iPrub22\_reconstruction.geneNames,"p")))

**Total "Genes" number in the reconstruction:** 6171
**Actual genes number:** 5703
**Artificial genes number:** 468
Spontaneous reactions: 86
Transport reactions: 117
Demand reactions: 37
Sink reactions: 1
Uptake reactions: 185
Production reactions: 42

#### 1.4.1 Name (▲)

%Genes names

Genes\_Types = ["Penicillium\_chrysogenum\_genes","Spontaneous\_genes","Transport\_genes","Demand\_genes","Sink\_genes","Uptake\_genes","Production\_genes"] ;

Pc=iPrub22\_reconstruction.geneNames(~cellfun('isempty',(strfind(iPrub22\_reconstruction.geneNames,'Pc'))));

S=iPrub22\_reconstruction.geneNames(~cellfun('isempty',(strfind(iPrub22\_reconstruction.geneNames,'s'))));

T=iPrub22\_reconstruction.geneNames(~cellfun('isempty',(strfind(iPrub22\_reconstruction.geneNames,'t'))));

D=iPrub22\_reconstruction.geneNames(~cellfun('isempty',(strfind(iPrub22\_reconstruction.geneNames,'d'))));

SK=iPrub22\_reconstruction.geneNames(~cellfun('isempty',(strfind(iPrub22\_reconstruction.geneNames,'sk')))); SK = [SK(1);{' '};{' '}] ;% Only one sink recation in iPrub22

U=iPrub22\_reconstruction.geneNames(~cellfun('isempty',(strfind(iPrub22\_reconstruction.geneNames,'u'))));

P=iPrub22\_reconstruction.geneNames(~cellfun('isempty',(strfind(iPrub22\_reconstruction.geneNames,'p'))));

disp(table(Pc(1:3),S(1:3),T(1:3),D(1:3),SK,U(1:3),P(1:3),'VariableNames', Genes\_Types))

**Penicillium\_chrysogenum\_genes** **Spontaneous\_genes** **Transport\_genes** **Demand\_genes** **Sink\_genes** **Uptake\_genes** **Production\_genes**
**\_\_\_\_\_\_\_\_\_\_\_\_\_\_\_\_\_\_\_\_\_\_\_\_\_\_\_\_\_** **\_\_\_\_\_\_\_\_\_\_\_\_\_\_\_\_\_** **\_\_\_\_\_\_\_\_\_\_\_\_\_\_\_** **\_\_\_\_\_\_\_\_\_\_\_\_** **\_\_\_\_\_\_\_\_\_\_** **\_\_\_\_\_\_\_\_\_\_\_\_** **\_\_\_\_\_\_\_\_\_\_\_\_\_\_\_\_**
'Pc13g04040' 's007' 't001' 'd001' 'sk001' 'u001' 'p001'
'Pc16g02170' 's001' 't002' 'd002' ' ' 'u002' 'p002'
'Pc16g09860' 's008' 't003' 'd003' ' ' 'u003' 'p003'

#### 1.4.2 Identifier (▲)

%Genes and Genes Products identifiers

fprintf(['<strong>Genes</strong>\n %d genes have an <strong>NCBI annotation</strong> of the form: %s\n',...

' %d genes have a <strong>KEGG annotation</strong> of the form: %s\n\n',...

'<strong>Gene Products</strong>\n %d gene products have an <strong>NCBI annotation</strong> of the form: %s\n', ...

' %d gene products have a <strong>UNIPROT annotation</strong> of the form: %s'],...

length(iPrub22\_reconstruction.proteinisncbigeneID(~cellfun('isempty',iPrub22\_reconstruction.proteinisncbigeneID))), iPrub22\_reconstruction.proteinisncbigeneID{1},...

length(iPrub22\_reconstruction.geneKEGGID(~cellfun('isempty',iPrub22\_reconstruction.geneKEGGID))),iPrub22\_reconstruction.geneKEGGID{1},...

length(iPrub22\_reconstruction.proteinisncbiproteinID(~cellfun('isempty',iPrub22\_reconstruction.proteinisncbiproteinID))), iPrub22\_reconstruction.proteinisncbiproteinID{1},...

length(iPrub22\_reconstruction.proteinisuniprotID(~cellfun('isempty',iPrub22\_reconstruction.proteinisuniprotID))),iPrub22\_reconstruction.proteinisuniprotID{1})

**Genes**
5703 genes have an **NCBI annotation** of the form: 8304049
5703 genes have a **KEGG annotation** of the form: pcs:Pc13g04040
**Gene Products**
5703 gene products have an **NCBI annotation** of the form: XP\_002558840
5703 gene products have a **UNIPROT annotation** of the form: B6H266

#### 1.4.3 SBO terms (▲)

SBOTerms descriptors are not yet supported as model features by Cobra Toolbox. In order to discretise the artificial reactions needed for modelling or those without associated genomic sequences from those resulting from the gap-filling steps, we decided to add an artificial gene to them (i.e. spontaneous, transport, demand, uptake and production reactions). Our reconstruction is therefore annotated with the following two terms:

- SBO:0000243 stands for 'gene' - 5,703 instances
- SBO:0000291 stands for 'empty set' - 468 instances

#### 1.4.4 Gene products compartmentation

The proposed reconstruction is not compartmentalised at the intracellular level. Nevertheless, an annotation related to the detection of signal peptides (SignalP - v4.1g), transmembrane domains (TMHMM - v2.0c), and the prediction of the subcellular localisation of proteins (DeepLoc - v1.0) has been made. As access to this information is not supported by CobraToolbox, it is included in the notes tag.

## 2. Model

From a reconstruction viewpoint, obtaining a consistent flux model requires some adjustments (e.g. reversibility, redundancy and non-reducibility being common issues). Therefore, the model presented below represents a specification of the reconstruction. We have chosen not to suppress any reactions, so to remedy the problems commonly encountered, we have set up our model to block them. Section 2.1 of this document summarises the actions taken (i.e. modifications have been directly incorporated into iPrub22; this document serves as a comprehensive reference for tracking and documenting these changes), and section 2.2 outlines some characteristics of the resulting model.

% Environnement definition

solverName = 'gurobi' ;

solverType = 'LP' ;

changeCobraSolver(solverName,solverType) ;

> changeCobraSolver: Gurobi interface added to MATLAB path.

iPrub22\_model = iPrub22\_reconstruction ;

%sparse format accelerates computations with large networks

iPrub22\_model.S = sparse(iPrub22\_model.S) ;

### 2.1 Reconstruction modifications: from reconstruction to model

SBOTerm - model

- SBO:0000624 stands for 'flux balance framework': "Modelling approach, typically used for metabolic models, where the flow of metabolites (flux) through a network can be calculated. This approach will generally produce a set of solutions (solution space), which may be reduced using objective functions and constraints on individual fluxes".

#### 2.1.1 List of parameters

For improved usability, we have added human-readable labels to identify the boundaries of the relevant reactions. We hope these labels enhance the user experience by providing easily understandable information about the scope and context of the reactions of interest.

SBOTerm - listOfParameters

- SBO:0000626 stands for 'default flux bound'
- SBO:0000625 stands for 'flux bound'

...

To facilitate the tracking of changes and their justifications, we have introduced four distinct labels within the list of parameters. In cases where a reaction had multiple labels, we have selected the most informative label to ensure clarity and transparency in documenting the modifications.

- blocked\_BOUND - see 2.1.2 Errors during model loading & 2.1.4 Reconcilation and duplication
- correction\_reversibility\_BOUND - see 2.1.3 Reversibility
- duplicate\_reaction\_BOUND - see 2.1.4 Reconcilation and duplication
- imbalanced\_reaction\_BOUND - see 2.1.5 Unbalanced reactions

Parameter\_Id = ['default\_ub';'default\_lb';"irrLow";'Exchange\_Biomass\_lb';'Exchange\_Biomass\_ub';'NGAM\_BOUND';'correction\_reversibility\_BOUND';'blocked\_BOUND';...

'imbalanced\_reaction\_BOUND';'duplicate\_reaction\_BOUND';'ub\_uptake\_...';'ub\_production\_...'];

Value = [1000;-1000;0;0;1000;1;0;0;0;0;"depends on media modelling conditions";"depends on media modelling conditions"];

Number\_of\_ub = [4358;0;0;0;1;1;180;111;961;80;185;42];

Number\_of\_lb = [0;618;3914;1;0;1;233;111;961;80;0;0];

disp(table(Parameter\_Id,Value,Number\_of\_ub,Number\_of\_lb))

**Parameter\_Id** **Value** **Number\_of\_ub** **Number\_of\_lb**
**\_\_\_\_\_\_\_\_\_\_\_\_\_\_\_\_\_\_\_\_\_\_\_\_\_\_\_\_\_\_\_\_** **\_\_\_\_\_\_\_\_\_\_\_\_\_\_\_\_\_\_\_\_\_\_\_\_\_\_\_\_\_\_\_\_\_\_\_\_\_\_\_** **\_\_\_\_\_\_\_\_\_\_\_\_** **\_\_\_\_\_\_\_\_\_\_\_\_**
"default\_ub" "1000" 4358 0
"default\_lb" "-1000" 0 618
"irrLow" "0" 0 3914
"Exchange\_Biomass\_lb" "0" 0 1
"Exchange\_Biomass\_ub" "1000" 1 0
"NGAM\_BOUND" "1" 1 1
"correction\_reversibility\_BOUND" "0" 180 233
"blocked\_BOUND" "0" 111 111
"imbalanced\_reaction\_BOUND" "0" 961 961
"duplicate\_reaction\_BOUND" "0" 80 80
"ub\_uptake\_..." "depends on media modelling conditions" 185 0
"ub\_production\_..." "depends on media modelling conditions" 42 0

fprintf("As a reminder, iPrub22 is composed of %d reactions and must therefore have as many reference tags for fbc:upperFluxBound="".\*"" (%d) and fbc:lowerFluxBound="".\*"" (%d)",...

length(iPrub22\_model.rxns),sum(Number\_of\_ub),sum(Number\_of\_lb))

As a reminder, iPrub22 is composed of 5919 reactions and must therefore have as many reference tags for fbc:upperFluxBound=".\*" (5919) and fbc:lowerFluxBound=".\*" (5919)

#### 2.1.2 Inconsistency during model loading

- 2.1.2.1 Errors in the stoichiometric matrix

When loading the reconstruction on MatLab, there are 61 differences between the stoichiometric matrices from the sbml output and the one generated from AuReMe. The matrix comparison highlights a problem related to the compound's management which is both reactant and product. In this case, the expected equation should be the following (), which will become (). However, in these 62 cases, the observed equation is of the form (. As illustrated by the 13-BETA-GLUCAN-SYNTHASE-RXN reaction below, these differences can be explained by the incorporation of overly generic and poorly encoded reactions (stochiometry carried by the metabolite name). As a result, 56 reactions are blocked (mostly A is a compound belonging to a class or super class such as protein class, RNA class, and compound class, and their associated reactions are unclear). Only 5 reactions are "corrected".

Reaction blocked (reactions annotated with the blocked\_BOUND tag)

Concerned\_rxns = {'13-BETA-GLUCAN-SYNTHASE-RXN';'2.7.7.8-RXN';'3.1.11.2-RXN';'3.1.4.1-RXN';'3.2.1.116-RXN';'3.2.1.143-RXN';'3.2.1.165-RXN';'3.2.1.3-RXN';...

'3.2.1.39-RXN';'3.2.1.55-RXN';'3.2.1.58-RXN';'3.2.1.59-RXN';'3.2.1.80-RXN';'3.2.1.84-RXN';'3.4.11.9-RXN';'3.6.4.1-RXN';'3.6.4.2-RXN';'3.6.4.4-RXN';...

'3.6.4.5-RXN';'3.6.4.6-RXN';'4.2.2.2-RXN';'ALPHA-13-GLUCAN-SYNTHASE-RXN';'ALPHA-AMYL-RXN';'ARYLSULFATE-SULFOTRANSFERASE-RXN';...

'CELLULOSE-SYNTHASE-UDP-FORMING-RXN';'CHITIN-SYNTHASE-RXN';'DNA-DIRECTED-DNA-POLYMERASE-RXN';'DNA-DIRECTED-RNA-POLYMERASE-RXN';...

'EXOPOLYPHOSPHATASE-RXN';'FOLYLPOLYGLUTAMATESYNTH-RXN';'FORMYLTHFGLUSYNTH-RXN';'GALACTURAN-14-ALPHA-GALACTURONIDASE-RXN';'GLYCOGENSYN-RXN';...

'MALTODEXGLUCOSID-RXN';'NAD+-ADP-RIBOSYLTRANSFERASE-RXN';'POLYNUCLEOTIDE-ADENYLYLTRANSFERASE-RXN';'POLYPHOSPHATE-KINASE-RXN';...

'RNA-DIRECTED-DNA-POLYMERASE-RXN';'RNA-DIRECTED-RNA-POLYMERASE-RXN';'RNA-URIDYLYLTRANSFERASE-RXN';'RXN-12171';'RXN-12193';'RXN-12391';'RXN-12392';....

'RXN-14353';'RXN-14354';'RXN-18082';'RXN-18085';'RXN-1826';'RXN-19781';'RXN-2103';'RXN-6341';'RXN-7668';'RXN0-2921';'RXN0-5181';'RXN0-5184'};

printRxnFormula(iPrub22\_model,Concerned\_rxns);

13-BETA-GLUCAN-SYNTHASE-RXN CPD-12575[c] -> UDP[c] + 1-3-beta-D-Glucans[c]
2.7.7.8-RXN Pi[c] -> Nucleoside-Diphosphates[c] + ssRNAs[c]
3.1.11.2-RXN WATER[c] -> DNA-N[c] + Deoxy-Ribonucleoside-Monophosphates[c]
3.1.4.1-RXN WATER[c] -> Oligonucleotides[c] + Nucleoside-Monophosphates[c]
3.2.1.116-RXN WATER[c] -> 1-4-alpha-D-Glucan[c] + MALTOTRIOSE[c]
3.2.1.143-RXN WATER[c] -> Poly-ADP-Riboses[c] + ADENOSINE\_DIPHOSPHATE\_RIBOSE[c]
3.2.1.165-RXN WATER[c] -> Chitosan[c] + GLUCOSAMINE[c]
3.2.1.3-RXN WATER[c] -> Glucopyranose[c] + Glycogens[c]
3.2.1.39-RXN WATER[c] -> Glucopyranose[c] + 1-3-beta-D-Glucans[c]
3.2.1.55-RXN WATER[c] -> 1-5-L-Arabinooligosaccharides[c] + CPD-12045[c]
3.2.1.58-RXN WATER[c] -> ALPHA-GLUCOSE[c] + 1-3-beta-D-Glucans[c]
3.2.1.59-RXN WATER[c] -> Glucopyranose[c] + 1-3-alpha-D-Glucans[c]
3.2.1.80-RXN WATER[c] -> BETA-D-FRUCTOSE[c] + Fructans[c]
3.2.1.84-RXN WATER[c] -> Glucopyranose[c] + 1-3-alpha-D-Glucans[c]
3.4.11.9-RXN WATER[c] -> Amino-Acids-20[c] + Peptides-holder[c]
3.6.4.1-RXN WATER[c] + ATP[c] -> PROTON[c] + ADP[c] + Pi[c] + Myosin-Actin[c]
3.6.4.2-RXN WATER[c] + ATP[c] -> PROTON[c] + ADP[c] + Pi[c] + Dynein-Microtubles-Complex[c]
3.6.4.4-RXN WATER[c] + ATP[c] -> PROTON[c] + ADP[c] + Pi[c] + Kinesin-Microtubules-Complex[c]
3.6.4.5-RXN WATER[c] + ATP[c] -> PROTON[c] + ADP[c] + Pi[c] + Kinesin-Microtubules-Complex[c]
3.6.4.6-RXN WATER[c] + ATP[c] -> PROTON[c] + ADP[c] + Pi[c] + 2 Membrane-Compartments[c]
4.2.2.2-RXN -> 1-4-alpha-D-galacturonosyl[c] + CPD-11244[c]
ALPHA-13-GLUCAN-SYNTHASE-RXN CPD-12575[c] -> UDP[c] + 1-3-alpha-D-Glucans[c]
ALPHA-AMYL-RXN WATER[c] -> 1-4-alpha-D-Glucan[c]
ARYLSULFATE-SULFOTRANSFERASE-RXN -> Phenols[c] + Aryl-sulfates[c]
CELLULOSE-SYNTHASE-UDP-FORMING-RXN CPD-12575[c] -> UDP[c] + CELLULOSE[c]
CHITIN-SYNTHASE-RXN UDP-N-ACETYL-D-GLUCOSAMINE[c] -> UDP[c] + CHITIN[c]
DNA-DIRECTED-DNA-POLYMERASE-RXN Deoxy-Ribonucleoside-Triphosphates[c] -> PPI[c] + DNA-N[c]
DNA-DIRECTED-RNA-POLYMERASE-RXN Nucleoside-Triphosphates[c] -> PPI[c] + RNA-Holder[c]
EXOPOLYPHOSPHATASE-RXN WATER[c] -> Pi[c] + Long-Chain-Polyphosphate[c]
FOLYLPOLYGLUTAMATESYNTH-RXN ATP[c] + GLT[c] -> ADP[c] + Pi[c] + THF-GLU-N[c]
FORMYLTHFGLUSYNTH-RXN ATP[c] + GLT[c] -> ADP[c] + Pi[c] + FORMYL-THF-GLU-N[c]
GALACTURAN-14-ALPHA-GALACTURONIDASE-RXN WATER[c] -> D-Galactopyranuronate[c] + 1-4-alpha-D-galacturonosyl[c]
GLYCOGENSYN-RXN ADP-D-GLUCOSE[c] -> ADP[c] + 1-4-alpha-D-Glucan[c]
MALTODEXGLUCOSID-RXN WATER[c] -> Glucopyranose[c] + 1-4-alpha-D-Glucan[c]
NAD+-ADP-RIBOSYLTRANSFERASE-RXN NAD[c] -> PROTON[c] + NIACINAMIDE[c] + ADP-D-Ribosyl-Acceptors[c]
POLYNUCLEOTIDE-ADENYLYLTRANSFERASE-RXN ATP[c] -> PPI[c] + RNA-Holder[c]
POLYPHOSPHATE-KINASE-RXN ATP[c] -> ADP[c] + Long-Chain-Polyphosphate[c]
RNA-DIRECTED-DNA-POLYMERASE-RXN Deoxy-Ribonucleoside-Triphosphates[c] -> PPI[c] + DNA-N[c]
RNA-DIRECTED-RNA-POLYMERASE-RXN Nucleoside-Triphosphates[c] -> PPI[c] + RNA-Holder[c]
RNA-URIDYLYLTRANSFERASE-RXN UTP[c] -> PPI[c] + RNA-Holder[c]
RXN-12171 Pi[c] -> GLC-1-P[c] + Maltodextrins[c]
RXN-12193 Glucopyranose[c] -> MALTOSE[c] + Maltodextrins[c]
RXN-12391 MALTOTRIOSE[c] -> Glucopyranose[c] + Glucans[c]
RXN-12392 Pi[c] -> GLC-1-P[c] + Linear-Malto-Oligosaccharides[c]
RXN-14353 Pi[c] -> GLC-1-P[c] + Soluble-Heteroglycans[c]
RXN-14354 Glucopyranose[c] -> MALTOSE[c] + Soluble-Heteroglycans[c]
RXN-18082 WATER[c] -> CHITIN[c] + N-acetyl-D-glucosamine[c]
RXN-18085 OXYGEN-MOLECULE[c] + Donor-H2[c] -> WATER[c] + Acceptor[c] + Cellulose-D-glucono-1-5-lactone[c] + CPD-7043[c]
RXN-1826 Pi[c] -> GLC-1-P[c] + Long-linear-glucans[c]
RXN-19781 -> Methyl-esterified-homogalacturonan[c] + CPD-12980[c]
RXN-2103 WATER[c] -> 1-4-alpha-D-galacturonosyl[c]
RXN-6341 ATP[c] + GLT[c] -> ADP[c] + Pi[c] + N5-Formyl-THF-Glu-N[c]
RXN-7668 CPD-12575[c] -> UDP[c] + POLY-GLUCOSYLATED-GLYCOGENINS[c]
RXN0-2921 ATP[c] + GLT[c] -> ADP[c] + Pi[c] + METHYLENE-THF-GLU-N[c]
RXN0-5181 WATER[c] -> 1-4-alpha-D-Glucan[c] + MALTOHEXAOSE[c]
RXN0-5184 Pi[c] -> GLC-1-P[c] + 1-4-alpha-D-Glucan[c]

% verification of the closure of these reactions

if sum(iPrub22\_model.ub(findRxnIDs(iPrub22\_model,(Concerned\_rxns)))) == 0

fprintf('The upper bounds of the <strong>%d reactions</strong> are closed (associated label is <strong>blocked\_BOUND</strong>)',length(Concerned\_rxns));

end

The upper bounds of the **56 reactions** are closed (associated label is **blocked\_BOUND**)

if sum(iPrub22\_model.lb(findRxnIDs(iPrub22\_model,(Concerned\_rxns)))) == 0

fprintf('The lower bounds of the <strong>%d reactions</strong> are closed (associated label is <strong>blocked\_BOUND</strong>)',length(Concerned\_rxns));

end

The lower bounds of the **56 reactions** are closed (associated label is **blocked\_BOUND**)

Reaction modification

Equation reactions initially contained in the \*.sbml file:

- RXN-3522: PROTON + NADPH[c] + 2 CPD-318[c] -> 2 PROTON[c] + NADP[c] + 2 ASCORBATE[c]
- 2.7.1.152-RXN: PROTON + ATP + MI-HEXAKISPHOSPHATE[c] -> PROTON[c] + ADP[c] + 5-DIPHOSPHO-1D-MYO-INOSITOL-12346P[c]
- RXN-10972: PROTON + ATP + MI-HEXAKISPHOSPHATE[c] -> PROTON[c] + ADP[c] + CPD-1170[c]
- RXN-12440: 2 ASCORBATE[c] + PROTON [c] + HYDROGEN-PEROXIDE[c] -> 2 WATER[c] + ASCORBATE[c] + L-DEHYDRO-ASCORBATE[c]
- RXN-18819: GLY[c] + RIBOSE[c] -> 2 WATER[c] + GLY[c] + CPD-10204[c]

Equation reactions after loading the model in Maltlab:

- RXN-3522: NADPH[c] + 2 CPD-318[c] -> 2 PROTON[c] + NADP[c] + 2 ASCORBATE[c]
- 2.7.1.152-RXN: ATP + MI-HEXAKISPHOSPHATE[c] -> PROTON[c] + ADP[c] + 5-DIPHOSPHO-1D-MYO-INOSITOL-12346P[c]
- RXN-10972: ATP + MI-HEXAKISPHOSPHATE[c] -> PROTON[c] + ADP[c] + CPD-1170[c]
- RXN-12440: PROTON [c] + HYDROGEN-PEROXIDE[c] -> 2 WATER[c] + ASCORBATE[c] + L-DEHYDRO-ASCORBATE[c]
- RXN-18819: RIBOSE[c] -> 2 WATER[c] + GLY[c] + CPD-10204[c]

We have observed that for these 5 reactions, the difference between the number of products and reactants has not been accounted for. We made changes to the stoichiometry of compounds directly in our \*.sbml file. However, please note that these reactions are not balanced.

- 2.1.2.2 Intracellular transport

In the proposed reconstruction, intracellular compartmentation has been excluded. Consequently, reactions modelling transport between organelles and cytoplasm are no longer applicable since the compartmentation of the associated metabolites is also inconsistent (i.e. the molecules involved must not be in the extracellular medium). To resolve this inconsistency, we have blocked these few reactions (blocked\_BOUND tag) while retaining them as a source of information through their Gene-Protein-Reaction (GPR) associations.

internal\_transport = {'3.6.3.16-RXN';'3.6.3.23-RXN';'3.6.3.30-RXN';'3.6.4.7-RXN';'ABC-24-RXN';'ABC-25-RXN';'TRANS-RXN-177';'TRANS-RXN-227';'TRANS-RXN-230';'TRANS-RXN-247';'r0924'} ;

#### 2.1.3 Reversibility

- 2.1.3.1 Updating reversibility with MetaCyc

When the reaction directionality is unknown, it may be declared as reversible, leading to biases in model generation and understanding. Database annotation evolves, and knowledge improves. Consequently, the 967 reversible reactions of the initial reconstruction were uploaded to the MetaCyc server using the SmartTable to check for a possible update of their directionality (787 reactions had a match on the database). At the time of the query (July 2022), about 100 reversible reactions were corrected.

Update\_reversibility = table2cell(readtable('./Updating\_files/Updating\_reversibility.txt','readvariablenames', false)) ;

fprintf(['%d reversible reactions with a MetaCyc Id \n\n', ...

' <strong>%d</strong> with annotation label <strong>LEFT-TO-RIGHT</strong>\n', ...

' <strong>%d</strong> with annotation label <strong>PHYSIOL-LEFT-TO-RIGHT</strong>\n', ...

' <strong>%d</strong> with annotation label <strong>RIGHT-TO-LEFT</strong>\n', ...

' <strong>%d</strong> with annotation label <strong>PHYSIOL-RIGHT-TO-LEFT</strong>\n\n', ...

' %d with annotation label REVERSIBLE\n', ...

' %d without annotation label'],...

length(Update\_reversibility), ...

length(find(Update\_reversibility(:,3) == "LEFT-TO-RIGHT")),...

length(find(Update\_reversibility(:,3) == "PHYSIOL-LEFT-TO-RIGHT")) ,...

length(find(Update\_reversibility(:,3) == "RIGHT-TO-LEFT")) ,...

length(find(Update\_reversibility(:,3) == "PHYSIOL-RIGHT-TO-LEFT")),...

length(find(Update\_reversibility(:,3) == "REVERSIBLE")) ,...

sum(cellfun('isempty',Update\_reversibility(:,3))))

787 reversible reactions with a MetaCyc Id
**12** with annotation label **LEFT-TO-RIGHT**
**70** with annotation label **PHYSIOL-LEFT-TO-RIGHT**
**3** with annotation label **RIGHT-TO-LEFT**
**13** with annotation label **PHYSIOL-RIGHT-TO-LEFT**
524 with annotation label REVERSIBLE
165 without annotation label

Based on this information, the reaction reversibility has been updated. The 165 reactions without annotation labels (i.e. unknown directionality) have been closed. All these changes are labelled correction\_reversibility\_BOUND

% list of reactions concerned

block\_ub = {'HOMOACONITATE-HYDRATASE-RXN';'RXN-11737';'RXN0-1147';'1.1.1.188-RXN';'1.1.1.212-RXN';'1.8.4.8-RXN';'15-OXOPROSTAGLANDIN-13-REDUCTASE-RXN';'4.2.1.103-RXN';...

'FUMARATE-REDUCTASE-NADH-RXN';'HOLOCYTOCHROME-C-SYNTHASE-RXN';'PROSTAGLANDIN-E2-9-REDUCTASE-RXN';'RXN-11315';'RXN-14171';'RXN-14805';'RXN-8773';'RXN-9510'};

if sum(iPrub22\_model.ub(findRxnIDs(iPrub22\_model,(block\_ub)))) == 0

fprintf('The upper bounds of these %d reactions are closed',length(block\_ub));

end

The upper bounds of these 16 reactions are closed

block\_lb = {'1.10.2.2-RXN';'5.99.1.2-RXN';'ACETYLORNDEACET-RXN';'ACSERLY-RXN';'AICARSYN-RXN';'ALLYSINE-DEHYDROG-RXN';'HOMOCYSMET-RXN';'RXN-13139';'RXN-17599';...

'TRANS-RXN-101';'TRANS-RXN-20';'TRANS-RXN0-200';'1.1.1.141-RXN';'1.1.1.250-RXN';'2-ACYLGLYCEROL-O-ACYLTRANSFERASE-RXN';'2-HYDROXY-3-OXOADIPATE-SYNTHASE-RXN';...

'2.4.2.31-RXN';'2.5.1.69-RXN';'2.7.11.24-RXN';'2.7.11.25-RXN';'2.7.12.1-RXN';'3.6.1.52-RXN';'4.1.2.20-RXN';'5.99.1.3-RXN';'ACETYLSPERMIDINE-DEACETYLASE-RXN';...

'ACYL-LYSINE-DEACYLASE-RXN';'AMPSYN-RXN';'ANTHRANSYN-RXN';'ARGDECARBOX-RXN';'D-GLUTAMATE-CYCLASE-RXN';'D-RIBULOKIN-RXN';'FORMATETHFLIG-RXN';...

'GLUCURONOLACTONE-REDUCTASE-RXN';'GLYCEROL-2-DEHYDROGENASE-NADP+-RXN';'L-FUCONATE-HYDRATASE-RXN';'MALTOSE-PHOSPHORYLASE-RXN';'MANNITOL-2-DEHYDROGENASE-RXN';...

'MANNPGUANYLTRANGDP-RXN';'OMEGA-AMIDASE-RXN';'PABASYN-RXN';'PHOSPHOGLUCOKINASE-RXN';'RNA-POLYMERASE-SUBUNIT-KINASE-RXN';'RXN-10737';'RXN-10763';'RXN-10948';...

'RXN-10967';'RXN-11191';'RXN-11485';'RXN-11889';'RXN-11890';'RXN-11963';'RXN-12122';'RXN-12497';'RXN-12499';'RXN-12675';'RXN-12727';'RXN-12817';'RXN-12819';...

'RXN-12820';'RXN-12826';'RXN-13197';'RXN-13682';'RXN-13722';'RXN-13997';'RXN-14120';'RXN-14205';'RXN-14393';'RXN-14819';'RXN-14906';'RXN-15565';'RXN-15703';...

'RXN-16063';'RXN-16759';'RXN-17099';'RXN-17627';'RXN-18932';'RXN-7716';'RXN-7971';'RXN-9918';'RXN1G-460';'THIAMIN-PYRIDINYLASE-RXN';'TYROSINE-PHENOL-LYASE-RXN'};

if sum(iPrub22\_model.lb(findRxnIDs(iPrub22\_model,(block\_lb)))) == 0

fprintf('The lower bounds of these %d reactions are closed',length(block\_lb));

end

The lower bounds of these 82 reactions are closed

block\_both = {'1.1.1.13-RXN';'1.1.1.197-RXN';'1.1.1.252-RXN';'1.1.1.289-RXN';'1.1.2.5-RXN';'1.1.99.27-RXN';'1.17.99.3-RXN';'1.2.1.18-RXN';'1.8.1.12-RXN';...

'2-ACETOLACTATE-MUTASE-RXN';'2-HYDROXYGLUTARATE-DEHYDROGENASE-RXN';'2-MEBUCOA-FAD-RXN';'2.1.1.3-RXN';'2.3.1.43-RXN';'2.4.1.94-RXN';'2.6.1.82-RXN';...

'2.7.11.14-RXN';'2.7.11.15-RXN';'2.7.11.16-RXN';'2.7.11.18-RXN';'2.7.11.19-RXN';'2.7.11.4-RXN';'2.7.11.6-RXN';'2.7.11.7-RXN';'2.7.12.2-RXN';'2.7.4.10-RXN';...

'2.7.7.45-RXN';'2.7.8.23-RXN';'2.8.3.13-RXN';'3.11.1.3-RXN';'3.5.1.86-RXN';'3.5.2.16-RXN';'3.5.5.5-RXN';'3.5.5.7-RXN';'3.6.1.24-RXN';'4.1.1.64-RXN';...

'4.1.2.42-RXN';'4.3.1.16-RXN';'5.1.1.11-RXN';'5.1.1.16-RXN';'ACNEULY-RXN';'ADP-DEAMINASE-RXN';'ALDEHYDE-DEHYDROGENASE-NADORNOP+-RXN';...

'ARALKYLAMINE-N-ACETYLTRANSFERASE-RXN';'ATP-ADENYLYLTRANSFERASE-RXN';'ATP-DEAMINASE-RXN';'BENZOIN-ALDOLASE-RXN';'BLASTICIDIN-S-DEAMINASE-RXN';...

'CARBODEHYDRAT-RXN';'CARBONYL-REDUCTASE-NADPH-RXN';'CELLOBIOSE-DEHYDROGENASE-ACCEPTOR-RXN';'CELLOBIOSE-PHOSPHORYLASE-RXN';'CHLORDECONE-REDUCTASE-RXN';...

'CHOLESTENONE-5-BETA-REDUCTASE-RXN';'CHOLINE-O-ACETYLTRANSFERASE-RXN';'CIS-2-ENOYL-COA-REDUCTASE-NADPH-RXN';'CORTISONE-BETA-REDUCTASE-RXN';...

'COUMARATE-REDUCTASE-RXN';'CYANAMIDE-HYDRATASE-RXN';'D-AMINO-ACID-N-ACETYLTRANSFERASE-RXN';'D-ARGINASE-RXN';'D-IDITOL-2-DEHYDROGENASE-RXN';...

'D-THREO-ALDOSE-1-DEHYDROGENASE-RXN';'DOLICHYLDIPHOSPHATASE-RXN';'ENDOPOLYPHOSPHATASE-RXN';'ETHANOLAMINE-PHOSPHATE-PHOSPHO-LYASE-RXN';...

'FAD-PYROPHOSPHATASE-RXN';'GENTISATE-DECARBOXYLASE-RXN';'GLUCOSE-1-PHOSPHATE-PHOSPHODISMUTASE-RXN';'GLUCOSE-16-BISPHOSPHATE-SYNTHASE-RXN';...

'GLUTAMATE-1-KINASE-RXN';'GSCYSDEG-RXN';'GUANIDINOACETASE-RXN';'HALOACETATE-DEHALOGENASE-RXN';'HYDROXYACID-OXOACID-TRANSHYDROGENASE-RXN';...

'HYDROXYPYRROLINEDEH-RXN';'KETOPANTOALDOLASE-RXN';'L-PIPECOLATE-DEHYDROGENASE-RXN';'LINOLEATE-ISOMERASE-RXN';'LYSINE-6-DEHYDROGENASE-RXN';...

'MALEATE-HYDRATASE-RXN';'MANNITOL-2-DEHYDROGENASE-NADP+-RXN';'NADH-DEHYDROGENASE-RXN';'NN-DIMETHYLFORMAMIDASE-RXN';...

'O-PYROCATECHUATE-DECARBOXYLASE-RXN';'POLYNUCLEOTIDE-5-HYDROXYL-KINASE-RXN';'PROPANEDIOL-PHOSPHATE-DEHYDROGENASE-RXN';...

'RETINOL-O-FATTY-ACYLTRANSFERASE-RXN';'RXN-10024';'RXN-1026';'RXN-10674';'RXN-10963';'RXN-10965';'RXN-10975';'RXN-10976';'RXN-10977';'RXN-10978';...

'RXN-10994';'RXN-1124';'RXN-12003';'RXN-12004';'RXN-12006';'RXN-12332';'RXN-12492';'RXN-12582';'RXN-12757';'RXN-12898';'RXN-12957';'RXN-12958';...

'RXN-13279';'RXN-13363';'RXN-13425';'RXN-13996';'RXN-14037';'RXN-14051';'RXN-14098';'RXN-14099';'RXN-14103';'RXN-14106';'RXN-14146';'RXN-14183';...

'RXN-14204';'RXN-14209';'RXN-14226';'RXN-14535';'RXN-14636';'RXN-15115';'RXN-15722';'RXN-15753';'RXN-16030';'RXN-16317';'RXN-16378';'RXN-16512';...

'RXN-16560';'RXN-16567';'RXN-16761';'RXN-16993';'RXN-16994';'RXN-17103';'RXN-17175';'RXN-17229';'RXN-17560';'RXN-17607';'RXN-17773';'RXN-17897';...

'RXN-1882';'RXN-7186';'RXN-7972';'RXN-8643';'RXN-9034';'RXN-9346';'RXN-9356';'RXN-9388';'RXN-9702';'RXN-9722';'RXN0-276';'SCYTALONE-DEHYDRATASE-RXN';...

'SORBOSE-5-DEHYDROGENASE-NADP+-RXN';'SORBOSE-DEHYDROGENASE-RXN';'STEROID-DELTA-ISOMERASE-RXN';'STYRENE-OXIDE-ISOMERASE-RXN';'TARTRATE-DECARBOXYLASE-RXN';...

'TAU-PROTEIN-KINASE-RXN';'THREONINE-RACEMASE-RXN';'URACIL-5-CARBOXYLATE-DECARBOXYLASE-RXN'};

if sum(iPrub22\_model.ub(findRxnIDs(iPrub22\_model,(block\_both)))) == 0 && sum(iPrub22\_model.lb(findRxnIDs(iPrub22\_model,(block\_lb)))) == 0

fprintf('The upper and lower bounds of these %d reactions are closed',length(block\_both));

end

The upper and lower bounds of these 165 reactions are closed

- 2.1.3.2 Reversibility according to the rules of Thiele and Palsson

Thiele and Palsson (2010) introduced a benchmark protocol that has since become widely recognised for generating high-quality genome-scale metabolic reconstructions. This protocol encompasses essential steps in model construction, including reaction reversibility consideration. (Thiele I, Palsson BØ. A protocol for generating a high-quality genome-scale metabolic reconstruction. Nat Protoc. janv 2010;5(1):93‑121. )

- Rule 1: 'reactions involving transfer of phosphate from ATP to an acceptor molecule should be irreversible (with the exception of the ATP synthetase, which is known to occur in reverse)'

% Printing the equations for reversible reactions involving ATP

printRxnFormula(iPrub22\_model,intersect(iPrub22\_model.rxns(find(iPrub22\_model.lb ~= 0 & iPrub22\_model.ub ~= 0)), findRxnsFromMets(iPrub22\_model,{'ATP[c]','ATP[e]'}))) ;

ACETATEKIN-RXN ATP[c] + ACET[c] <=> ADP[c] + ACETYL-P[c]
ACETYLGLUTKIN-RXN ATP[c] + ACETYL-GLU[c] <=> ADP[c] + N-ACETYL-GLUTAMYL-P[c]
ADENYL-KIN-RXN ATP[c] + AMP[c] <=> 2 ADP[c]
ATPPHOSPHORIBOSYLTRANS-RXN PPI[c] + PHOSPHORIBOSYL-ATP[c] <=> PROTON[c] + ATP[c] + PRPP[c]
ATPSYN-RXN WATER[c] + 3 PROTON[c] + ATP[c] <=> ADP[c] + Pi[c] + 4 PROTON[e]
GALACTOKIN-RXN ATP[c] + ALPHA-D-GALACTOSE[c] <=> PROTON[c] + ADP[c] + GALACTOSE-1P[c]
GLYCEROL-KIN-RXN GLYCEROL[c] + ATP[c] <=> PROTON[c] + ADP[c] + GLYCEROL-3P[c]
GMKALT-RXN ATP[c] + DGMP[c] <=> ADP[c] + DGDP[c]
GUANYL-KIN-RXN ATP[c] + GMP[c] <=> ADP[c] + GDP[c]
MEVALONATE-KINASE-RXN ATP[c] + MEVALONATE[c] <=> PROTON[c] + ADP[c] + CPD-499[c]
NGAM 4.14 WATER[c] + 4.14 ATP[c] -> 4.14 ADP[c] + 4.14 Pi[c]
PEPSYNTH-RXN WATER[c] + PYRUVATE[c] + ATP[c] <=> 2 PROTON[c] + AMP[c] + Pi[c] + PHOSPHO-ENOL-PYRUVATE[c]
PHOSGLYPHOS-RXN ATP[c] + G3P[c] <=> ADP[c] + DPG[c]
PHOSPHOMEVALONATE-KINASE-RXN ATP[c] + CPD-499[c] <=> ADP[c] + CPD-641[c]
PHOSPHORIBULOKINASE-RXN ATP[c] + RIBULOSE-5P[c] <=> PROTON[c] + ADP[c] + D-RIBULOSE-15-P2[c]
PROPIONYL-COA-CARBOXY-RXN ATP[c] + HCO3[c] + PROPIONYL-COA[c] <=> PROTON[c] + ADP[c] + Pi[c] + D-METHYL-MALONYL-COA[c]
PROPKIN-RXN ATP[c] + PROPIONATE[c] <=> ADP[c] + PROPIONYL-P[c]
PRPPSYN-RXN ATP[c] + RIBOSE-5P[c] <=> PROTON[c] + AMP[c] + PRPP[c]
PYRUVATEORTHOPHOSPHATE-DIKINASE-RXN PYRUVATE[c] + ATP[c] + Pi[c] <=> PROTON[c] + AMP[c] + PPI[c] + PHOSPHO-ENOL-PYRUVATE[c]
RXN-11832 ATP[c] + CMP[c] <=> CDP[c] + ADP[c]
RXN-14196 ATP[c] + CARBAMATE[c] <=> ADP[c] + CARBAMOYL-P[c]
RXN-14228 ATP[c] + CPD0-2231[c] <=> ADP[c] + DITP[c]
RXN-14569 ATP[c] + HCO3[c] + THR[c] <=> WATER[c] + PPI[c] + CPD-15435[c]
SUCCCOASYN-RXN CO-A[c] + ATP[c] + SUC[c] <=> ADP[c] + Pi[c] + SUC-COA[c]
SULFATE-ADENYLYLTRANS-RXN PROTON[c] + ATP[c] + SULFATE[c] <=> PPI[c] + APS[c]

Regarding this rule, no action was undertaken.

- Rule 2: 'reactions involving quinones are generally irreversible'

At the time of the query (August 2022), 148 metabolites belong to the MetaCyc 'quinone' ontology compound, and 17 are present in the reconstruction.

quinones\_compounds = {'ALPHA-TOCOPHEROL[c]','BETA-TOCOPHEROL[c]','DELTA-TOCOPHEROL[c]','GAMA-TOCOPHEROL[c]','CPD-10174[c]','UBIQUINONE-6[c]','UBIQUINONE-8[c]',...

'CPD-12588[c]','CPD-10172[c]','CPD-10169[c]','CPD-9612[c]','2-METHYL-3-PHYTYL-14-NAPHTHOQUINONE[c]','CPD-15152[c]','CPD-15153[c]','CPD-3766[c]','DOPAQUINONE[c]',...

'CPD-8094[c]','ALPHA-TOCOPHEROL[e]','BETA-TOCOPHEROL[e]','DELTA-TOCOPHEROL[e]','GAMA-TOCOPHEROL[e]','CPD-10174[e]','UBIQUINONE-6[e]','UBIQUINONE-8[e]',...

'CPD-12588[e]','CPD-10172[e]','CPD-10169[e]','CPD-9612[e]','2-METHYL-3-PHYTYL-14-NAPHTHOQUINONE[e]','CPD-15152[e]','CPD-15153[e]','CPD-3766[e]',...

'DOPAQUINONE[e]','CPD-8094[e]'} ;

% Printing the equations for reactions involving a quinone

printRxnFormula(iPrub22\_model,findRxnsFromMets(iPrub22\_model,quinones\_compounds)) ;

1.5.5.1-RXN-YEAST ETF-Reduced[c] + UBIQUINONE-6[c] -> PROTON[c] + ETF-Oxidized[c] + UBIQUINOL-30[c]
Demand\_036 UBIQUINONE-6[e] -> UBIQUINONE-6[c]
Demand\_037 UBIQUINONE-8[e] -> UBIQUINONE-8[c]
GLYC3PDEHYDROG-RXN GLYCEROL-3P[c] + UBIQUINONE-8[c] -> CPD-9956[c] + DIHYDROXY-ACETONE-PHOSPHATE[c]
MONOPHENOL-MONOOXYGENASE-RXN OXYGEN-MOLECULE[c] + TYR[c] -> WATER[c] + DOPAQUINONE[c]
NADH-DEHYDROGENASE-QUINONE-RXN PROTON[c] + NADH[c] + CPD-3766[c] -> NAD[c] + MENADIOL[c]
Production\_040 CPD-10174[e] ->
RXN-11003 OXYGEN-MOLECULE[c] + ALPHA-TOCOPHEROL[c] + Red-NADPH-Hemoprotein-Reductases[c] -> WATER[c] + Ox-NADPH-Hemoprotein-Reductases[c] + CPD-11960[c]
RXN-11624 OXYGEN-MOLECULE[c] + CPD-12588[c] -> WATER[c] + CPD-10162[c]
RXN-13061 OXYGEN-MOLECULE[c] + 2 L-DIHYDROXY-PHENYLALANINE[c] -> 2 WATER[c] + 2 DOPAQUINONE[c]
RXN-14177 S-ADENOSYLMETHIONINE[c] + CPD-15152[c] -> PROTON[c] + ADENOSYL-HOMO-CYS[c] + CPD-15153[c]
RXN-15378 SUC[c] + CPD-9612[c] -> FUM[c] + CPD-15301[c]
RXN-19671 PROTON[c] + NADH-P-OR-NOP[c] + 2-METHYL-3-PHYTYL-14-NAPHTHOQUINONE[c] -> NAD-P-OR-NOP[c] + CPD-12831[c]
RXN-2562 S-ADENOSYLMETHIONINE[c] + DELTA-TOCOPHEROL[c] -> PROTON[c] + ADENOSYL-HOMO-CYS[c] + BETA-TOCOPHEROL[c]
RXN-8332 OXYGEN-MOLECULE[c] + 2 CPD-8098[c] -> 2 WATER[c] + 2 CPD-8094[c]
RXN-8483 DOPAQUINONE[c] -> PROTON[c] + CPD-8652[c]
RXN-9346 Acceptor[c] + CPD-9956[c] -> Donor-H2[c] + UBIQUINONE-8[c]
RXN-9479 5 PROTON[c] + 7 MALONYL-COA[c] + Hexanoyl-ACPs[c] -> 7 CO-A[c] + 2 WATER[c] + 7 CARBON-DIOXIDE[c] + ACP[c] + CPD-12588[c]
RXN-9489 NADPH[c] + CPD-10168[c] -> NADP[c] + CPD-10169[c]
RXN-9490 NADPH[c] + CPD-10170[c] -> NADP[c] + CPD-10172[c]
RXN-9491 PROTON[c] + NADPH[c] + CPD-10171[c] -> NADP[c] + CPD-10174[c]
RXN-9492 PROTON[c] + NADPH[c] + OXYGEN-MOLECULE[c] + CPD-10169[c] -> WATER[c] + NADP[c] + CPD-10172[c]
RXN-9493 WATER[c] + CPD-10172[c] -> ACET[c] + CPD-10174[c]
RXN0-5258-Yeast UBIQUINONE-6[c] + GLYCEROL-3P[c] -> UBIQUINOL-30[c] + DIHYDROXY-ACETONE-PHOSPHATE[c]
RXN0-5330-YEAST PROTON[c] + NADH[c] + UBIQUINONE-6[c] -> NAD[c] + UBIQUINOL-30[c]
SUCCINATE-DEHYDROGENASE-UBIQUINONE6-RXN UBIQUINONE-6[c] + SUC[c] -> UBIQUINOL-30[c] + FUM[c]
TOCOPHEROL-O-METHYLTRANSFERASE-RXN S-ADENOSYLMETHIONINE[c] + GAMA-TOCOPHEROL[c] -> PROTON[c] + ADENOSYL-HOMO-CYS[c] + ALPHA-TOCOPHEROL[c]
Transport\_206 CPD-10174[c] -> CPD-10174[e]
Uptake\_173 -> UBIQUINONE-6[e]
Uptake\_174 -> UBIQUINONE-8[e]
r0510 OXYGEN-MOLECULE[c] + 2-Methoxy-6-polyprenyl-phenols[c] -> WATER[c] + CPD-15152[c]
r0511 S-ADENOSYLMETHIONINE[c] + CPD-15152[c] -> ADENOSYL-HOMO-CYS[c] + CPD-15153[c]
r0512 NADPH[c] + OXYGEN-MOLECULE[c] + CPD-15153[c] -> WATER[c] + NADP[c] + OCTAPRENYL-METHYL-OH-METHOXY-BENZQ[c]

The only reversible reaction found in the network was: RXN-9346. It has been blocked in the following steps.

printRxnFormula(iPrub22\_model,'RXN-9346') ;

RXN-9346 Acceptor[c] + CPD-9956[c] -> Donor-H2[c] + UBIQUINONE-8[c]

fprintf("Upper bound: %d\nLower bound: %d", iPrub22\_model.ub(findRxnIDs(iPrub22\_model,'RXN-9346')),iPrub22\_model.lb(findRxnIDs(iPrub22\_model,'RXN-9346')));

Upper bound: 0
Lower bound: 0

Thus, there is no reversible reaction involving a quinone in iPrub22.

printRxnFormula(iPrub22\_model,intersect(iPrub22\_model.rxns(find(iPrub22\_model.lb ~= 0 & iPrub22\_model.ub ~= 0)),findRxnsFromMets(iPrub22\_model,quinones\_compounds))) ;

- 2.1.3.3 Reversibility in iPrub22

fprintf(['Model contains <strong>%d</strong> reversible reactions\n\n',...

' %d Transport reactions\n', ...

' %d iAL1006 reactions\n', ...

' %d with compatible MetaCyc identifiers\n'], ...

length(find(iPrub22\_model.lb ~= 0 & iPrub22\_model.ub ~= 0)),...

sum(~cellfun('isempty',(cellfun(@(x)regexp(x, 'Transport\_[0-9]{3}'),iPrub22\_model.rxns(find(iPrub22\_model.lb ~= 0 & iPrub22\_model.ub ~= 0)),'UniformOutput',false)))),...

sum(~cellfun('isempty',(cellfun(@(x)regexp(x, 'r[0-9]{4}'),iPrub22\_model.rxns(find(iPrub22\_model.lb ~= 0 & iPrub22\_model.ub ~= 0)),'UniformOutput',false)))),...

length(find(iPrub22\_model.lb ~= 0 & iPrub22\_model.ub ~= 0))-(126+7))

Model contains **603** reversible reactions
126 Transport reactions
7 iAL1006 reactions
470 with compatible MetaCyc identifiers

#### 

#### 2.1.4 Reconciliation and Duplication

This section deals with computational and biological/chemical redundancy contained in the reconstruction.

2.1.4.1 Pair of similar reactions

Within the reconstruction, we have identified groups (often pairs) of reactions that are similar in their biological sense. Duplicated reactions from the point of view of the stochiometric matrix are dealt with in the next section.

Here, we refer first to redundant reactions concerning metabolite naming (generic name vs specific name). For instance, if D-galactopyranose is a compound class composed of ALPHA-D-GALACTOSE and GALACTOSE, and if an interconversion reaction of alpha and beta form exists, it seems reasonable to replace D-galactopyranose by ALPHA-D-GALACTOSE. The second type of redundancy involves pair of reactions where one is a MetaCyc identifier and the other a homemade identifier. In such a case, we retained the more balanced reaction. Additionally, we ensured that the GPRs were identical or that all the genes from one reaction were included in the other. Finally, we have observed a third form of redundancy, which arises from the order in which compounds are written in the reaction equation (i.e.  and  ). However, it is worth noting that this case is rare, affecting only two reactions in the entire reconstruction.

similar\_reactions = {{'r0004','GLUCOKIN-RXN'}; ...

{'r0027','GLU6PDEHYDROG-RXN'}; ...

{'r0106','GLYCOLATE-REDUCTASE-RXN'}; ...

{'r0143','RXN-19329'}; ...

{'r0149','GLYOHMETRANS-RXN'}; ...

{'r0158','FORMALDEHYDE-TRANSKETOLASE-RXN'}; ...

{'r0165','RXN-8773'}; ...

{'r0177', '1.1.1.289-RXN'}; ...

{'r0178','MANNPISOM-RXN'}; ...

{'r0184','PHOSMANMUT-RXN'};...

{'r0195','TREHALA-RXN'}; ...

{'r0212','AMYLOMALT-RXN'}; ...

{'r0222','5.4.99.16-RXN'}; ...

{'r0224','RXN-12078'}; ...

{'r0257','GLYCEROL-KIN-RXN'}; ...

{'r0293','CDPDIGLYSYN-RXN'}; ...

{'r0294','PHOSPHASERSYN-RXN'}; ...

{'r0295', 'PHOSPHASERDECARB-RXN'}; ...

{'r0296', '2.1.1.17-RXN'}; ...

{'r0297', '2.1.1.71-RXN'}; ...

{'r0298', 'RXN4FS-2'}; ...

{'r0299', 'PHOSPHAGLYPSYN-RXN'}; ...

{'r0301','RXN-8141'}; ...

{'r0314', '3.1.4.2-RXN'}; ...

{'r0356','RXN0-1441'}; ...

{'r0374','ADENYL-KIN-RXN'}; ...

{'r0423','URIDINE-NUCLEOSIDASE-RXN'}; ...

{'r0453','NADH-KINASE-RXN'}; ...

{'r0458','NAD-KIN-RXN'}; ...

{'r0537','1-PHOSPHATIDYLINOSITOL-KINASE-RXN'}; ...

{'r0538','2.7.1.68-RXN'}; ...

{'r0539','1-PHOSPHATIDYLINOSITOL-3-KINASE-RXN'}; ...

{'r0551','GLUTAMATE-DEHYDROGENASE-RXN'}; ...

{'r0553','RXN-20084'}; ...

{'r0577','r0580','r0581','RXN66-546'}; ...

{'r0579','PYRROLINECARBREDUCT-RXN'}; ...

{'r0681', '3-HYDROXYISOBUTYRATE-DEHYDROGENASE-RXN'}; ...

{'r0683', 'HOMOCITRATE-SYNTHASE-RXN'}; ...

{'r0685','HOMOACONITATE-HYDRATASE-RXN'}; ...

{'r0757', 'RXN-12332'}; ...

{'SERINE--GLYOXYLATE-AMINOTRANSFERASE-RXN','SERINE-GLYOXYLATE-AMINOTRANSFERASE-RXN'}};

% Verification (displaying 3 pairs of reactions to illustrate)

formulas = printRxnFormula(iPrub22\_model,'printFlag',false, 'gprFlag',true) ;

for i = 1:3

for j = 1:length(similar\_reactions{i})

reaction\_index = findRxnIDs(iPrub22\_model,similar\_reactions{i}{j}) ;

genes = regexp(iPrub22\_model.rules{reaction\_index},'x\(([0-9]+)\)', 'tokens');

rule = iPrub22\_model.rules{reaction\_index};

for k = 1:length(genes)

rule = regexprep(rule,'x\([0-9]+\)','${iPrub22\_reconstruction.genes{str2num(char(genes{k}))}}','once') ;

end

rule = regexprep(rule,'\|','or'); rule = regexprep(rule,'&','and'); rule = regexprep(rule,'( ','('); rule = regexprep(rule,' )',')') ;

fprintf(['\nReaction <strong>ID</strong>: %s\n' ...

'Reaction <strong>Formulae</strong>: %s\n' ...

'<strong>GPR associations</strong>: %s\n'],...

iPrub22\_model.rxns{reaction\_index},...

formulas{reaction\_index},rule)

end

disp(' ');disp('---------------------------------------------------------------------------------------------------------------');disp(' ')

end

Reaction **ID**: r0004
Reaction **Formulae**: ALPHA-GLUCOSE[c] + ATP[c] -> ALPHA-GLC-6-P[c] + ADP[c]
**GPR associations**: (gp\_Pc22g08480 or gp\_Pc22g08490)
Reaction **ID**: GLUCOKIN-RXN
Reaction **Formulae**: ATP[c] + Glucopyranose[c] -> PROTON[c] + ADP[c] + D-glucopyranose-6-phosphate[c]
**GPR associations**: (gp\_Pc21g02550 or gp\_Pc20g13040 or gp\_Pc22g05190 or gp\_Pc21g20770 or gp\_Pc22g23550 or gp\_Pc22g08480)

---------------------------------------------------------------------------------------------------------------

Reaction **ID**: r0027
Reaction **Formulae**: ALPHA-GLC-6-P[c] + NADP[c] -> NADPH[c] + D-6-P-GLUCONO-DELTA-LACTONE[c]
**GPR associations**: (gp\_Pc20g03310 or gp\_Pc20g03330)
Reaction **ID**: GLU6PDEHYDROG-RXN
Reaction **Formulae**: NADP[c] + D-glucopyranose-6-phosphate[c] -> PROTON[c] + NADPH[c] + D-6-P-GLUCONO-DELTA-LACTONE[c]
**GPR associations**: gp\_Pc20g03330

---------------------------------------------------------------------------------------------------------------

Reaction **ID**: r0106
Reaction **Formulae**: NAD[c] + GLYCOLLATE[c] -> NADH[c] + GLYOX[c]
**GPR associations**: gp\_Pc21g22080
Reaction **ID**: GLYCOLATE-REDUCTASE-RXN
Reaction **Formulae**: NAD[c] + GLYCOLLATE[c] <=> PROTON[c] + NADH[c] + GLYOX[c]
**GPR associations**: ((gp\_Pc21g22040 and gp\_Pc20g15620) or gp\_Pc20g14820 or (gp\_Pc16g12980 and gp\_Pc21g22080 and gp\_Pc22g04940) or gp\_Pc22g04940 or gp\_Pc21g23650 or gp\_Pc20g10430)

---------------------------------------------------------------------------------------------------------------

redundant\_rxns = {'r0004';'r0027';'r0106';'r0143';'r0149';'r0158';'r0165';'r0177';'r0178';'r0184';'r0195';'r0212';'r0222';'r0224';'r0257';'r0293';'r0294';'r0295';'r0296';'r0297';...

'r0298';'r0299';'r0301';'r0314';'r0356';'r0374';'r0423';'r0453';'r0458';'r0537';'r0538';'r0539';'r0551';'r0553';'r0577';'r0579';'r0580';'r0581';'r0681';'r0683';'r0685';'r0757';...

'SERINE--GLYOXYLATE-AMINOTRANSFERASE-RXN'} ;

These reactions are annotated with the label blocked\_bound.

2.1.4.2 Duplicate reactions

As previously stated, our approach involved retaining all reactions within the model without removal. Instead, we opted to block the reactions identified by the checkDuplicateRxn function, which can be distinguished by the duplicate\_reaction\_BOUND tag.

duplicateRxnInd = ' ' ;

modelIn= iPrub22\_model ;

i=0 ;

while ~ isempty(duplicateRxnInd)

[modelOut, duplicateRxnInd, keptRxnInd] = checkDuplicateRxn(modelIn,'S') ;

printRxnFormula(modelIn, modelIn.rxns(duplicateRxnInd)) ;

modelIn = modelOut ;

i = i + length(duplicateRxnInd);

end

Warning: Reaction: 3.4.21.48-RXN has more than one replicate

Warning: Reaction: ACETATE--COA-LIGASE-RXN has more than one replicate

Warning: Reaction: ALANINE-AMINOTRANSFERASE-RXN has more than one replicate

Warning: Reaction: CATAL-RXN has more than one replicate

Warning: Reaction: RXN-14274 has more than one replicate

Warning: Reaction: RXN-14786 has more than one replicate

Warning: Reaction: RXN-14794 has more than one replicate

RXN-19748 MAL[c] + NAD[c] -> PYRUVATE[c] + CARBON-DIOXIDE[c] + NADH[c]
r0157 NAD[c] + FORMATE[c] -> CARBON-DIOXIDE[c] + NADH[c]
r0129 NAD[c] + METHYLENE-THF-GLU-N[c] -> NADH[c] + 5-10-METHENYL-THF-GLU-N[c]
r0652 2-ACETO-LACTATE[c] -> CPD-231[c]
3.2.1.84-RXN WATER[c] -> Glucopyranose[c] + 1-3-alpha-D-Glucans[c]
3.4.25.1-RXN WATER[c] + General-Protein-Substrates[c] -> 2 Peptides-holder[c]
3.6.4.5-RXN WATER[c] + ATP[c] -> PROTON[c] + ADP[c] + Pi[c] + Kinesin-Microtubules-Complex[c]
r0026 CO-A[c] + ATP[c] + ACET[c] -> ACETYL-COA[c] + AMP[c] + PPI[c]
ALKAPHOSPHA-RXN WATER[c] + Orthophosphoric-Monoesters[c] -> Pi[c] + Alcohols[c]
r0542 2-KETOGLUTARATE[c] + L-ALPHA-ALANINE[c] -> PYRUVATE[c] + GLT[c]
RXN-10817 WATER[c] + OXYGEN-MOLECULE[c] + PHENYLETHYLAMINE[c] -> HYDROGEN-PEROXIDE[c] + AMMONIUM[c] + PHENYLACETALDEHYDE[c]
RXN-13697 2-KETOGLUTARATE[c] + L-ASPARTATE[c] -> GLT[c] + OXALACETIC\_ACID[c]
r1010 CO-A[c] + ATP[c] + Fatty-Acids[c] -> AMP[c] + PPI[c] + ACYL-COA[c]
r0735 2 HYDROGEN-PEROXIDE[c] -> 2 WATER[c] + OXYGEN-MOLECULE[c]
r0327 WATER[c] + CHITIN[c] -> ACET[c] + Chitosan[c]
RXN-15830 8 PROTON[c] + OXYGEN-MOLECULE[c] + 4 Cytochromes-C-Reduced[e] -> 2 WATER[c] + 4 PROTON[e] + 4 Cytochromes-C-Oxidized[e]
RNA-DIRECTED-DNA-POLYMERASE-RXN Deoxy-Ribonucleoside-Triphosphates[c] -> PPI[c] + DNA-N[c]
RNA-DIRECTED-RNA-POLYMERASE-RXN Nucleoside-Triphosphates[c] -> PPI[c] + RNA-Holder[c]
r0130 NADP[c] + METHYLENE-THF-GLU-N[c] -> NADPH[c] + 5-10-METHENYL-THF-GLU-N[c]
r0330 WATER[c] + N-acetyl-D-glucosamine[c] -> ACET[c] + GLUCOSAMINE[c]
RXN-16133 NAD[c] + L-3-HYDROXYACYL-COA[c] -> PROTON[c] + NADH[c] + 3-KETOACYL-COA[c]
r1024 CO-A[c] + ATP[c] + CPD-195[c] -> AMP[c] + PPI[c] + CPD-196[c]
RXN-9940 WATER[c] + OXYGEN-MOLECULE[c] + N1-ACETYLSPERMINE[c] -> HYDROGEN-PEROXIDE[c] + SPERMIDINE[c] + CPD-10687[c]
r1059 CO-A[c] + ATP[c] + HEXANOATE[c] -> AMP[c] + PPI[c] + HEXANOYL-COA[c]
r0968 CO-A[c] + CPD0-2105[c] -> ACETYL-COA[c] + CPD-10267[c]
TRANS-RXN0-207 WATER[c] + ATP[c] + CU+[c] -> PROTON[c] + ADP[c] + Pi[c] + CU+[e]
r0944 WATER[c] + CPD-15656[c] -> CPD-15657[c]
r0991 OXYGEN-MOLECULE[c] + CPD-15651[c] -> HYDROGEN-PEROXIDE[c] + CPD-15675[c]
r0992 WATER[c] + CPD-15675[c] -> CPD-15652[c]
r1000 WATER[c] + CPD-15663[c] -> CPD-15658[c]
r0242 WATER[c] + GLYCEROL-3P[c] -> GLYCEROL[c] + Pi[c]
RXN-19355 Protein-L-lysine[c] + S-ubiquitinyl-UCP-E2-L-cysteine[c] -> PROTON[c] + Ubiquitin-carrier-protein-E2-L-cysteine[c] + PROTEIN-N-UBIQUITYL-LYSINE[c]
RXN-8618 WATER[c] + GERANYL-PP[c] -> PPI[c] + GERANIOL[c]
r1101 CO-A[c] + ATP[c] + BUTYRIC\_ACID[c] -> AMP[c] + PPI[c] + BUTYRYL-COA[c]
r1102 CO-A[c] + ATP[c] + VALERATE[c] -> AMP[c] + PPI[c] + PENTANOYLCOA-CPD[c]
r0514 CYS[c] + Cysteine-Desulfurase-L-cysteine[c] -> L-ALPHA-ALANINE[c] + L-Cysteine-Desulfurase-persulfide[c]
RXN1G-469 PROTON[c] + NADPH[c] + 3-oxo-behenoyl-ACPs[c] -> NADP[c] + R-3-hydroxybehenoyl-ACPs[c]
Transport\_028 NITRITE[c] -> NITRITE[e]
Transport\_061 L-CITRULLINE[c] -> L-CITRULLINE[e]
Transport\_057 L-ORNITHINE[c] -> L-ORNITHINE[e]
Transport\_042 Pi[c] -> Pi[e]
r0056 WATER[c] + ACETYL-COA[c] -> CO-A[c] + ACET[c]
r0112 FUM[c] + FADH2[c] -> SUC[c] + FAD[c]
r0150 WATER[c] + GLY[c] + METHYLENE-THF-GLU-N[c] -> THF[c] + SER[c]
r0377 2 ADP[c] -> ATP[c] + AMP[c]
r0552 WATER[c] + NAD[c] + GLT[c] -> NADH[c] + 2-KETOGLUTARATE[c] + AMMONIA[c]
r0975 WATER[c] + CPD-10262[c] -> CPD0-2253[c]
r0977 NAD[c] + CPD0-2253[c] -> NADH[c] + CPD-10260[c]
r0979 CO-A[c] + CPD-10260[c] -> ACETYL-COA[c] + PALMITYL-COA[c]
r0980 CO-A[c] + CPD-14271[c] -> ACETYL-COA[c] + STEAROYL-COA[c]
r0966 WATER[c] + CPD-7222[c] -> CPD0-2107[c]
r0967 NAD[c] + CPD0-2107[c] -> NADH[c] + CPD0-2105[c]
r0993 NAD[c] + CPD-15652[c] -> NADH[c] + CPD-15676[c]
r0994 CO-A[c] + CPD-15676[c] -> ACETYL-COA[c] + CPD-15655[c]
r1092 CO-A[c] + ATP[c] + ARACHIDIC\_ACID[c] -> AMP[c] + PPI[c] + CPD-9965[c]
r1054 CO-A[c] + ATP[c] + CPD-7830[c] -> AMP[c] + PPI[c] + CPD-14723[c]
r1049 CO-A[c] + ATP[c] + CPD-8462[c] -> AMP[c] + PPI[c] + CPD-14724[c]
r1080 CO-A[c] + ATP[c] + CPD-7836[c] -> AMP[c] + PPI[c] + TETRADECANOYL-COA[c]
r1107 PROPIONYL-COA[c] + CARNITINE[c] -> CO-A[c] + O-Acyl-L-Carnitines[c]
r1130 NADPH[c] + OXYGEN-MOLECULE[c] + EPISTEROL[c] -> 2 WATER[c] + NADP[c] + CPD-700[c]

Warning: Reaction: 3.4.21.48-RXN has more than one replicate

Warning: Reaction: CATAL-RXN has more than one replicate

3.4.24.73-RXN WATER[c] + General-Protein-Substrates[c] -> 2 Peptides-holder[c]
r0025 CO-A[c] + ATP[c] + ACET[c] -> ACETYL-COA[c] + AMP[c] + PPI[c]
RXN-13698 2-KETOGLUTARATE[c] + L-ALPHA-ALANINE[c] -> PYRUVATE[c] + GLT[c]
r0734 2 HYDROGEN-PEROXIDE[c] -> 2 WATER[c] + OXYGEN-MOLECULE[c]
r0914 CO-A[c] + CPD0-2105[c] -> ACETYL-COA[c] + CPD-10267[c]
r0940 WATER[c] + CPD-15675[c] -> CPD-15652[c]
r0948 WATER[c] + CPD-15663[c] -> CPD-15658[c]

Warning: Reaction: 3.4.21.48-RXN has more than one replicate

3.4.24.49-RXN WATER[c] + General-Protein-Substrates[c] -> 2 Peptides-holder[c]
r0733 2 HYDROGEN-PEROXIDE[c] -> 2 WATER[c] + OXYGEN-MOLECULE[c]

Warning: Reaction: 3.4.21.48-RXN has more than one replicate

3.4.24.39-RXN WATER[c] + General-Protein-Substrates[c] -> 2 Peptides-holder[c]

Warning: Reaction: 3.4.21.48-RXN has more than one replicate

3.4.24.37-RXN WATER[c] + General-Protein-Substrates[c] -> 2 Peptides-holder[c]

Warning: Reaction: 3.4.21.48-RXN has more than one replicate

3.4.24.15-RXN WATER[c] + General-Protein-Substrates[c] -> 2 Peptides-holder[c]

Warning: Reaction: 3.4.21.48-RXN has more than one replicate

3.4.24.11-RXN WATER[c] + General-Protein-Substrates[c] -> 2 Peptides-holder[c]

Warning: Reaction: 3.4.21.48-RXN has more than one replicate

3.4.23.5-RXN WATER[c] + General-Protein-Substrates[c] -> 2 Peptides-holder[c]

Warning: Reaction: 3.4.21.48-RXN has more than one replicate

3.4.23.3-RXN WATER[c] + General-Protein-Substrates[c] -> 2 Peptides-holder[c]

Warning: Reaction: 3.4.21.48-RXN has more than one replicate

3.4.23.27-RXN WATER[c] + General-Protein-Substrates[c] -> 2 Peptides-holder[c]

Warning: Reaction: 3.4.21.48-RXN has more than one replicate

3.4.23.25-RXN WATER[c] + General-Protein-Substrates[c] -> 2 Peptides-holder[c]

Warning: Reaction: 3.4.21.48-RXN has more than one replicate

3.4.23.19-RXN WATER[c] + General-Protein-Substrates[c] -> 2 Peptides-holder[c]

Warning: Reaction: 3.4.21.48-RXN has more than one replicate

3.4.23.18-RXN WATER[c] + General-Protein-Substrates[c] -> 2 Peptides-holder[c]

Warning: Reaction: 3.4.21.48-RXN has more than one replicate

3.4.23.1-RXN WATER[c] + General-Protein-Substrates[c] -> 2 Peptides-holder[c]

Warning: Reaction: 3.4.21.48-RXN has more than one replicate

3.4.21.92-RXN WATER[c] + General-Protein-Substrates[c] -> 2 Peptides-holder[c]

Warning: Reaction: 3.4.21.48-RXN has more than one replicate

3.4.21.63-RXN WATER[c] + General-Protein-Substrates[c] -> 2 Peptides-holder[c]

Warning: Reaction: 3.4.21.48-RXN has more than one replicate

3.4.21.61-RXN WATER[c] + General-Protein-Substrates[c] -> 2 Peptides-holder[c]
3.4.21.53-RXN WATER[c] + General-Protein-Substrates[c] -> 2 Peptides-holder[c]

duplicate\_rxns = {'3.2.1.84-RXN';'3.4.21.53-RXN';'3.4.21.61-RXN';'3.4.21.63-RXN';'3.4.21.92-RXN';'3.4.23.18-RXN';'3.4.23.19-RXN';'3.4.23.1-RXN';'3.4.23.25-RXN';...

'3.4.23.27-RXN';'3.4.23.3-RXN';'3.4.23.5-RXN';'3.4.24.11-RXN';'3.4.24.15-RXN';'3.4.24.37-RXN';'3.4.24.39-RXN';'3.4.24.49-RXN';'3.4.24.73-RXN';'3.4.25.1-RXN';...

'3.6.4.5-RXN';'ALKAPHOSPHA-RXN';'r0025';'r0026';'r0056';'r0112';'r0129';'r0130';'r0150';'r0157';'r0242';'r0327';'r0330';'r0377';'r0514';'r0542';'r0552';'r0652';...

'r0733';'r0734';'r0735';'r0914';'r0940';'r0944';'r0948';'r0966';'r0967';'r0968';'r0975';'r0977';'r0979';'r0980';'r0991';'r0992';'r0993';'r0994';'r1000';'r1010';...

'r1024';'r1049';'r1054';'r1059';'r1080';'r1092';'r1101';'r1102';'r1107';'r1130';'RNA-DIRECTED-DNA-POLYMERASE-RXN';'RNA-DIRECTED-RNA-POLYMERASE-RXN';'RXN-10817';...

'RXN-13697';'RXN-13698';'RXN-15830';'RXN-16133';'RXN-19355';'RXN-19748';'RXN1G-469';'RXN-8618';'RXN-9940';'Transport\_028';'Transport\_042';'Transport\_057';...

'Transport\_061';'TRANS-RXN0-207'} ;

NB. Reactions 3.2.1.84-RXN, 3.6.4.5-RXN and DNA-DIRECTED-DNA-POLYMERASE-RXN were associated with the blocked\_BOUND tag.

#### 2.1.5 Unbalanced reactions

[massImbalance, imBalancedMass, imBalancedCharge, imBalancedRxnBool, Elements, missingFormulaeBool, balancedMetBool] = checkMassChargeBalance(iPrub22\_model);

fprintf(['Number of reactions: <strong>%d\n</strong>',...

' Reactions with mass-imbalance: <strong>%d (%.2f%%)</strong>\n',...

' Reactions with charge imbalance: <strong>%d (%.2f%%)</strong>\n',...

' Imbalance reactions (exchange reactions are included): <strong>%d (%.2f%%)</strong>\n\n',...

'Number of metabolites: <strong>%d</strong>\n',...

' Metabolites without formulae: <strong>%d (%.2f%%)</strong>\n',...

' Metabolites exclusively involved in balanced reactions: <strong>%d (%.2f%%)</strong>\n'],...

length(iPrub22\_model.rxns),...

length(imBalancedMass(~cellfun('isempty',imBalancedMass))),length(imBalancedMass(~cellfun('isempty',imBalancedMass)))\*100/length(iPrub22\_model.rxns),...

sum(imBalancedCharge ~= 0),sum(imBalancedCharge ~= 0)\*100/length(iPrub22\_model.rxns),...

sum(imBalancedRxnBool),sum(imBalancedRxnBool)\*100/length(iPrub22\_model.rxns),...

length(iPrub22\_model.mets),sum(missingFormulaeBool),sum(missingFormulaeBool)\*100/length(iPrub22\_model.mets),sum(balancedMetBool),...

sum(balancedMetBool)\*100/length(iPrub22\_model.mets)) ;

Number of reactions: **5919** Reactions with mass-imbalance: **1398 (23.62%)**
Reactions with charge imbalance: **969 (16.37%)**
Imbalance reactions (exchange reactions are included): **1402 (23.69%)**
Number of metabolites: **5464**
Metabolites without formulae: **192 (3.51%)**
Metabolites exclusively involved in balanced reactions: **3517 (64.37%)**

% Remove the exchange reactions from the set of unbalanced reactions

Uptake = strmatch('Uptake',iPrub22\_model.rxns) ;

Production = strmatch('Production',iPrub22\_model.rxns) ;

Sink = strmatch('Sink',iPrub22\_model.rxns) ;

Exchange = [Uptake ; Production ; Sink ] ;

Imbalanced\_reactions = setdiff(iPrub22\_model.rxns(find(imBalancedRxnBool==1)),iPrub22\_model.rxns(Exchange)) ;

% Remove from the set of unbalanced reactions the biomass and its assimilated reactions

Biomass\_rxns\_and\_assimilate = {'Biomass\_rxn';'Exchange\_Biomass';'Transport\_Biomass';'r1455';'r1456';'r1457';'r1458';'r1459';'r1460';'r1465'} ;

Imbalanced\_reactions = setdiff(Imbalanced\_reactions,Biomass\_rxns\_and\_assimilate);

% Remove from the set of unbalanced reactions the set of reactions that have already been closed

Already\_blocked = unique([Concerned\_rxns ; internal\_transport ; block\_ub ; block\_lb ; block\_both ; duplicate\_rxns ; redundant\_rxns]) ;

Blocked\_in\_previous\_curation = intersect(Imbalanced\_reactions,Already\_blocked ) ;

Imbalanced\_reactions = setdiff(Imbalanced\_reactions,Blocked\_in\_previous\_curation);

% Remove from the set of unbalanced reactions the reactions involved in the biosynthesis of specialised metabolites (penicillin, roquefortin, isoepoxydon, toluquinol, ferrichrome, etc.)

Biosynthesis\_specialised\_metabolites = {'METHYLGLUTACONYL-COA-HYDRATASE-RXN';'RXN-15470';'RXN-15472';'RXN-15473';'RXN-15480';'RXN-15950';'RXN-16125';'RXN-16128';'RXN-16140';...

'RXN-16141';'RXN-16142';'RXN-16143';'RXN-16144';'RXN-16147';'RXN-8809';'RXN-9479';'RXN-9481';'RXN-9484';'RXN-9486';'RXN-9487';'RXN-9489';'RXN-9494';'RXN-9497'};

Imbalanced\_reactions = setdiff(Imbalanced\_reactions,Biosynthesis\_specialised\_metabolites);

% Remove from the set of unbalanced reactions those that prevent biomass production when all the uptakes are open

Retained\_reaction = {'ADENYLOSUCCINATE-SYNTHASE-RXN';'AIRCARBOXY-RXN';'AIRS-RXN';'ATPPHOSPHORIBOSYLTRANS-RXN';'DETHIOBIOTIN-SYN-RXN';'FGAMSYN-RXN';'GART-RXN';...

'HISTPRATPHYD-RXN';'PGPPHOSPHA-RXN';'RXN-19329';'RXN-20084';'RXN-6081';'RXN-8141';'RXN-9384';'RXN3O-130';'RXN3O-75';'SAICARSYN-RXN';'THIOSULFATE--THIOL-SULFURTRANSFERASE-RXN';...

'r0479';'r0480';'r0491';'r0729';'r0995';'r1125';'NGAM'} ;

Imbalanced\_reactions = setdiff(Imbalanced\_reactions,Retained\_reaction);

fprintf(['Of the <strong>%d</strong> unbalanced reactions detected by the checkMassChargeBalance function:\n\n',...

' <strong>%d</strong> are exchange reactions\n',...

' <strong>%d</strong> are the biomass and its assimilated reactions\n',...

' <strong>%d</strong> have been closed during previous curations\n',...

' <strong>%d</strong> are required to produce specialised metabolites\n',...

' <strong>%d</strong> are required to maintain biomass production\n',...

' <strong>%d</strong> are blocked and annotated with <strong>imbalanced\_reaction\_bound</strong> tag\n'],...

length(iPrub22\_model.rxns(find(imBalancedRxnBool==1))),...

length(Exchange),length(Biomass\_rxns\_and\_assimilate),length(Blocked\_in\_previous\_curation),length(Biosynthesis\_specialised\_metabolites),...

length(Retained\_reaction),length(Imbalanced\_reactions)) ;

Of the **1402** unbalanced reactions detected by the checkMassChargeBalance function:
**228** are exchange reactions
**10** are the biomass and its assimilated reactions
**170** have been closed during previous curations
**23** are required to produce specialised metabolites
**25** are required to maintain biomass production
**946** are blocked and annotated with **imbalanced\_reaction\_bound** tag

NB. To improve traceability, we preferred to keep mainly the most informative tags for previously closed reactions: correction\_reversibility\_BOUND, blocked\_BOUND, or duplicate\_reaction\_BOUND. It ensures a more comprehensive and informative representation of the modifications made to these reactions. Thus, of the 170 reactions blocked at previous stages, only 15 had their label changed to imbalanced\_reaction\_bound.

#### 

#### 2.1.6 Other modifications

Finally, the following modifications have been implemented in the model:

Addition of reaction r0144: This reaction, initially removed due to its localisation in the mitochondria, has been reintroduced as it plays an essential role in folate biosynthesis.

r0144 5,6,7,8-tetrahydrofolate:NADP+ oxidoreductase NADP(+)[m] + dihydrofolate[m] <=> NADPH[m] + folates[m] Pc13g03650;Pc20g03140 Folate biosynthesis (iAL1006)

printRxnFormula(iPrub22\_model,'r0144') ;

r0144 NAD[c] + DIHYDROFOLATE-GLU-N[c] <=> PROTON[c] + NADH[c] + Folates[c]

Modification of the THF cofactor: Reactions RXN-19329 and r0598 have been adjusted to utilise the more generic THF-GLU-N compound class. These changes prevent the mandatory (but artificial) uptake of Folates.

printRxnFormula(iPrub22\_model,{'RXN-19329','r0598'}) ;

RXN-19329 PROTON[c] + NADPH[c] + DIHYDROFOLATE[c] -> NADP[c] + THF-GLU-N[c]
r0598 5-METHYL-THF-GLU-N[c] + HOMO-CYS[c] -> MET[c] + THF-GLU-N[c]

Closure of the upper bound of RXN-2901: This closure unblocks the model's sensitivity to carbon sources addition. RXN-2901 was detected by digging reactions belonging to Erroneous Energy-generating Cycles.

printRxnFormula(iPrub22\_model,'RXN-2901') ;

RXN-2901 2-KETOGLUTARATE[c] + B-ALANINE[c] <=> GLT[c] + MALONATE-S-ALD[c]

→ Overall, a total of 1,607 reactions, which accounts for approximately 27% of the network, have undergone modifications to at least one of their bounds.

### 

### 2.2 Model Characteristics

In this section, we introduce three models:

- The default\_model icorresponds to the model as it is presented at the time of loading (parametrised reconstruction).
- The open\_model is a variant where all uptakes are open (arbitrary choice of 10 ).
- The closed\_model represents the closure of all uptakes, meaning that no external metabolites are allowed to enter the system.

% Exchange reactions

Uptake = strmatch('Uptake',iPrub22\_model.rxns) ;

Production = strmatch('Production',iPrub22\_model.rxns) ;

% Models definition

default\_model = iPrub22\_reconstruction ;

open\_model = iPrub22\_reconstruction;

open\_model = changeRxnBounds(open\_model , open\_model.rxns(Uptake), 10, 'u') ;

closed\_model = iPrub22\_reconstruction ;

closed\_model = changeRxnBounds(closed\_model, closed\_model.rxns(Uptake), 0, 'u') ;

closed\_model = changeRxnBounds(closed\_model, closed\_model.rxns(Production), 0, 'u') ;

#### 

By defining these three models, we can compare and analyse the impact of different uptake configurations on the behaviour and characteristics of the metabolic network in extreme cases.

#### 2.2.1 Numerical properties of a reconstruction

This section, extracted from the tutorial "Numerical Properties of a Reconstruction", delves into the numerical properties of a stoichiometric matrix. Analyzing the numerical properties is crucial for examining the metabolic reconstruction under consideration, selecting the appropriate solver, and identifying inconsistencies within the network. The tutorial was applied to the three GSMNs: iAL1006 [1], Prubens[2], and iPrub22 to compare the matrix evolution.

- [1] Agren, R. et al. The RAVEN Toolbox and Its Use for Generating a Genome-scale Metabolic Model for Penicillium chrysogenum. PLoS Computational Biology 9, 2013.
- [2] Prigent, S. et al. Reconstruction of 24 Penicillium genome scale metabolic models shows diversity based on their secondary metabolism. Biotechnology and Bioengineering 115, 2604–2612, 2018.

tic

iAL1006 = readCbModel('./Network/iAl1006\_v1.00.xml')

iAL1006 = struct with fields:

S: [2269×1660 double]
mets: {2269×1 cell}
b: [2269×1 double]
csense: [2269×1 char]
rxns: {1660×1 cell}
lb: [1660×1 double]
ub: [1660×1 double]
c: [1660×1 double]
osenseStr: 'max'
genes: {}
rules: {1660×1 cell}
compNames: {5×1 cell}
comps: {5×1 cell}
metFormulas: {2269×1 cell}
metNames: {2269×1 cell}
metNotes: {2269×1 cell}
metSBOTerms: {2269×1 cell}
rxnNames: {1660×1 cell}
rxnSBOTerms: {1660×1 cell}
subSystems: {1660×1 cell}
description: 'iAl1006\_v1.00.xml'
modelVersion: [1×1 struct]
modelName: 'Penicillium chrysogenum genome-scale model'
modelID: 'iAL1006'
modelAnnotation: '<annotation>↵ <rdf:RDF xmlns:rdf="http://www.w3.org/1999/02/22-rdf-syntax-ns#" xmlns:dc="http://purl.org/dc/elements/1.1/" xmlns:dcterms="http://purl.org/dc/terms/" xmlns:vCard="http://www.w3.org/2001/vcard-rdf/3.0#" xmlns:bqbiol="http://biomodels.net/biology-qualifiers/" xmlns:bqmodel="http://biomodels.net/model-qualifiers/">↵ <rdf:Description rdf:about="#metaid\_iAL1006">↵ <dc:creator rdf:parseType="Resource">↵ <rdf:Bag>↵ <rdf:li rdf:parseType="Resource">↵ <vCard:N rdf:parseType="Resource">↵ <vCard:Family>Agren</vCard:Family>↵ <vCard:Given>Rasmus</vCard:Given>↵ </vCard:N>↵ <vCard:EMAIL>rasmus.agren@chalmers.se</vCard:EMAIL>↵ <vCard:ORG>↵ <vCard:Orgname>Chalmers University of Technology, Gothenburg</vCard:Orgname>↵ </vCard:ORG>↵ </rdf:li>↵ </rdf:Bag>↵ </dc:creator>↵ <dcterms:created rdf:parseType="Resource">↵ <dcterms:W3CDTF>2013-02-07T16:48:52Z</dcterms:W3CDTF>↵ </dcterms:created>↵ <dcterms:modified rdf:parseType="Resource">↵ <dcterms:W3CDTF>2013-02-07T16:48:52Z</dcterms:W3CDTF>↵ </dcterms:modified>↵ <bqbiol:is>↵ <rdf:Bag>↵ <rdf:li rdf:resource="urn:miriam:taxonomy:5076"/>↵ </rdf:Bag>↵ </bqbiol:is>↵ </rdf:Description>↵ </rdf:RDF>↵</annotation>'
modelNotes: '<notes>↵ <body xmlns="http://www.w3.org/1999/xhtml">↵ <p>This is a reconstruction of the biochemical network of the filamentous fungi <i>Penicillium chrysogenum</i>.</p>↵ <p>Technical notes:<ul>↵ <li>The compartments included here have no volume defined; there are no reliable estimates available for those volumes yet.</li>↵ <li>There are no kinetic functions defined for the reactions because this model only represents the chemical structure of the network (stoichiometry).</li>↵ <li>Reactions for uptake and excretion are defined for some of the metabolites. All uptake reactions are constrained to zero flux and all excretion reactions are unconstrained.</li>↵ <li>All genes are assigned to the cytosol. This has no physiological meaning, but it is necessary for the structure of the model.</li>↵ </ul></p>↵ <p>This SBML representation of the Penicillium chrysogenum metabolic network is made available under the Creative Commons Attribution-Share Alike 3.0 Unported Licence (see www.creativecommons.org).</p>↵ </body>↵</notes>'

toc

Elapsed time is 8.874758 seconds.

tic

Prubens = readCbModel('./Network/Prubens.xml')

The model contains 5004 errors.
Error encountered during read.

Prubens = struct with fields:

S: [3045×2574 double]
mets: {3045×1 cell}
b: [3045×1 double]
csense: [3045×1 char]
rxns: {2574×1 cell}
lb: [2574×1 double]
ub: [2574×1 double]
c: [2574×1 double]
osenseStr: 'max'
genes: {1787×1 cell}
rules: {2574×1 cell}
geneNames: {1787×1 cell}
compNames: {13×1 cell}
comps: {13×1 cell}
proteins: {1787×1 cell}
metNames: {3045×1 cell}
rxnNames: {2574×1 cell}
subSystems: {2574×1 cell}
description: 'Prubens.xml'
modelVersion: [1×1 struct]
modelName: 'Prubens'
modelID: 'Prubens'

toc

Elapsed time is 18.895411 seconds.

Basic numerical characteristics

nMets = [length(iAL1006.mets); length(Prubens.mets); length(iPrub22\_model.mets)] ;

nRxns = [length(iAL1006.rxns); length(Prubens.rxns) ;length(iPrub22\_model.rxns)];

nElem = [numel(iAL1006.S); numel(Prubens.S); numel(iPrub22\_model.S)];

nNz = [nnz(iAL1006.S); nnz(Prubens.S); nnz(iPrub22\_model.S)] ;

Model = ["iAL1006","Prubens","iPrub22"] ;

Features = ["Number\_of\_Metabolites","Number\_of\_reactions","Number\_of\_elements\_in\_S","Number\_of\_nonzero\_elements\_in\_S"];

- Number of elements: represents the total number of entries in the stoichiometric matrix (including zero elements). This number is equivalent to the product of the number of reactions and the number of metabolites.
- Number of nonzero elements: represents the total number of nonzero entries in the stoichiometric matrix (excluding zero elements).

disp(table(nMets,nRxns,nElem,nNz,'VariableNames', Features,'RowNames', Model))

**Number\_of\_Metabolites** **Number\_of\_reactions** **Number\_of\_elements\_in\_S** **Number\_of\_nonzero\_elements\_in\_S**
**\_\_\_\_\_\_\_\_\_\_\_\_\_\_\_\_\_\_\_\_\_** **\_\_\_\_\_\_\_\_\_\_\_\_\_\_\_\_\_\_\_** **\_\_\_\_\_\_\_\_\_\_\_\_\_\_\_\_\_\_\_\_\_\_\_** **\_\_\_\_\_\_\_\_\_\_\_\_\_\_\_\_\_\_\_\_\_\_\_\_\_\_\_\_\_\_\_**
**iAL1006** 2269 1660 3.7665e+06 5963
**Prubens** 3045 2574 7.8378e+06 12009
**iPrub22** 5464 5919 3.2341e+07 26640

Rank

- Rank: the rank of a stoichiometric matrix is the maximum number of linearly independent rows and is equivalent to the number of linearly independent columns. The rank is a measurement of how many reactions and metabolites are linearly independent.

% Determine the rank of the stoichiometric matrix

rankS = [rank(full(iAL1006.S));rank(full(Prubens.S));rank(full(iPrub22\_model.S))];

- Rank deficiency: the rank deficiency of the stoichiometric matrix is a measure of how many reactions and metabolites are linearly dependent and expressed as the ratio of the rank of the stoichiometric matrix to the theoretical full rank.

% calculate the rank deficiency (in per cent)

for i = 1:3

rankDeficiencyS(i,:) = (1 - rankS(i) / min(nMets(i), nRxns(i))) \* 100 ;

end

disp(table(rankS,rankDeficiencyS,'RowNames', Model))

**rankS** **rankDeficiencyS**
**\_\_\_\_\_** **\_\_\_\_\_\_\_\_\_\_\_\_\_\_\_**
**iAL1006** 1228 26.024
**Prubens** 2208 14.219
**iPrub22** 4485 17.917

Sparsity and Density

- The Sparsity ratio: ratio of the number of zero elements and the total number of elements. The sparser the stoichiometric matrix, the fewer metabolites participate in each reaction. The sparsity ratio is particularly useful to compare models by how many metabolites participate in each reaction.

for i = 1:3

sparsityRatio(i,:) = [(1 - nNz(i) / nElem(i)) \* 100.0] ; % Determine the sparsity ratio of S (in per cent)

end

- The Complementary sparsity ratio: is calculated as the difference of 100 and the sparsity ratio expressed in per cent, and therefore, is a ratio of the number of nonzero elements and the total number of elements.

for i = 1:3

compSparsityRatio(i,:) = 100.0 - sparsityRatio(i) ; % Determine the complementary sparsity ratio (in per cent)

end

- The Average column density: corresponds to the ratio of the number of nonzero elements in each column (i.e. reaction) and the total number of metabolites. The average column density corresponds to the arithmetic average of all the column densities (the sum of all the column densities divided by the number of reactions). The average column density provides a measure of how many stoichiometric coefficients participate in each biochemical reaction on average.

%iAL1006

%% Add the number of non-zeros in each column (reaction)

colDensityAv = 0;

for j = 1:nRxns(1)

colDensityAv = colDensityAv + nnz(iAL1006.S(:, j));

end

%% Calculate the arithmetic average number of non-zeros in each column

colDensityAvs(1,:) = colDensityAv / nRxns(1) ;

%Prubens

colDensityAv = 0;

for j = 1:nRxns(2)

colDensityAv = colDensityAv + nnz(Prubens.S(:, j));

end

colDensityAvs(2,:) = colDensityAv / nRxns(2) ;

%iPrub22

colDensityAv = 0;

for j = 1:nRxns(1)

colDensityAv = colDensityAv + nnz(iPrub22\_model.S(:, j));

end

colDensityAvs(3,:) = colDensityAv / nRxns(3) ;

- The relative column density corresponds to the ratio of the number of nonzero elements in each column and the total number of metabolites. The relative column density corresponds to the average column density divided by the total number of metabolites (expressed in per cent). The relative column density may also be expressed as parts-per-million [ppm] for large-scale or huge-scale models. The relative column density indicates how many metabolites are being used on average in each reaction relative to the total number of metabolites in the metabolic network.

for i = 1:3

colDensityRel(i,:) = colDensityAvs(i) / nMets(i) \* 100 ; % determine the density proportional to the length of the column

end

disp(table(sparsityRatio,compSparsityRatio,colDensityAvs,colDensityRel,'RowNames', Model))

**sparsityRatio** **compSparsityRatio** **colDensityAvs** **colDensityRel**
**\_\_\_\_\_\_\_\_\_\_\_\_\_** **\_\_\_\_\_\_\_\_\_\_\_\_\_\_\_\_\_** **\_\_\_\_\_\_\_\_\_\_\_\_\_** **\_\_\_\_\_\_\_\_\_\_\_\_\_**
**iAL1006** 99.842 0.15832 3.5922 0.15832
**Prubens** 99.847 0.15322 4.6655 0.15322
**iPrub22** 99.918 0.082371 1.2629 0.023113

Sparsity Pattern (spy plot)

The visualisation of the sparsity pattern is useful to explore the matrix, spot inconsistencies, or identify patterns visually. In addition to the standard sparsity pattern, the magnitude of the elements of the stoichiometric matrix (stoichiometric coefficients) is shown as proportional to the size of the dot.

% iAl1006

figure

spyc(iAL1006.S, colormap(advancedColormap('cobratoolbox')));

% Prubens

figure

spyc(Prubens.S, colormap(advancedColormap('cobratoolbox')));

% iPrub22

figure

spyc(iPrub22\_model.S, colormap(advancedColormap('cobratoolbox')));

Singular Values and Condition Number

A singular value decomposition of the stoichiometric matrix is the decomposition into orthonormal matrices U (of dimension nMets by nMets) and V (of dimension nRxns by nRxns) and a matrix with nonnegative diagonal elements D such that .

Note that the calculation of singular values is numerically expensive, especially for large stoichiometric matrices.

% Calculate the singular values

svVect1 = svds(iAL1006.S, rankS(1));

svVect2 = svds(Prubens.S, rankS(2));

svVect3 = svds(iPrub22\_model.S, rankS(3));

The svds() function returns the number of singular values specified in the second argument of the function. As most stoichiometric matrices are rank deficient, some singular values are zero (or within numerical tolerances). The cut-off is located at the rank of the stoichiometric matrix.

% Determine the vector with all singular values (including zeros)

svVectAll1 = svds(iAL1006.S, min(nMets(1), nRxns(1)));

svVectAll2 = svds(Prubens.S, min(nMets(2), nRxns(2)));

svVectAll3 = svds(iPrub22\_model.S, min(nMets(3), nRxns(3)));

The singular values and their cut-off can be illustrated as follows:

%iAL1006

% Plot the singular values

figure;

% Plot the singular values up to rankS

semilogy(linspace(1, length(svVect1), length(svVect1)), svVect1, '\*');

% Plot all singular values

hold on;

semilogy(linspace(1, length(svVectAll1), length(svVectAll1)), svVectAll1, 'ro');

set(gca, 'fontsize', 14);

legend('svds (up to rankS)', 'svds (all)')

grid minor;

xlabel('Number of the singular value');

ylabel('Magnitude of the singular value');

hold off;

% Prubens

% Plot the singular values

figure;

% Plot the singular values up to rankS

semilogy(linspace(1, length(svVect2), length(svVect2)), svVect2, '\*');

% Plot all singular values

hold on;

semilogy(linspace(1, length(svVectAll2), length(svVectAll2)), svVectAll2, 'ro');

set(gca, 'fontsize', 14);

legend('svds (up to rankS)', 'svds (all)')

grid minor;

xlabel('Number of the singular value');

ylabel('Magnitude of the singular value');

hold off;

% iPrub22

% Plot the singular values

figure;

% Plot the singular values up to rankS

semilogy(linspace(1, length(svVect3), length(svVect3)), svVect3, '\*');

% Plot all singular values

hold on;

semilogy(linspace(1, length(svVectAll3), length(svVectAll3)), svVectAll3, 'ro');

set(gca, 'fontsize', 14);

legend('svds (up to rankS)', 'svds (all)')

grid minor;

xlabel('Number of the singular value');

ylabel('Magnitude of the singular value');

hold off;

Only singular values greater than zero (numbered from 1 to rank(S)) are of interest.

- The Maximum singular value: the largest element on the diagonal matrix obtained from singular value decomposition.
- The Minimum singular value: the smallest element on the diagonal matrix obtained from singular value decomposition.

% determine the maximum and minimum singular values

maxSingVal = [svVect1(1);svVect2(1);svVect3(1)] ; % first value of the vector with singular values

minSingVal = [svVect1(rankS(1)); svVect2(rankS(2)); svVect3(rankS(3))]; % smallest non-zero singular value

- The Condition number: the condition number of the stoichiometric matrix is the ratio of the maximum and minimum singular values. The higher this ratio, the more ill-conditioned the stoichiometric matrix is (numerical issues) and, generally, the longer the simulation time is.

% determine the condition number

for i = 1:3

condNumber(i,:) = maxSingVal(i) / minSingVal(i) ;

end

disp(table(maxSingVal,minSingVal, condNumber,'RowNames', Model))

**maxSingVal** **minSingVal** **condNumber**
**\_\_\_\_\_\_\_\_\_\_** **\_\_\_\_\_\_\_\_\_\_** **\_\_\_\_\_\_\_\_\_\_**
**iAL1006** 217.22 0.010721 20262
**Prubens** 110.94 0.0053052 20912
**iPrub22** 220.9 0.018844 11722

#### 2.2.2 Identify metabolic dead-ends

The detectDeadEnds function identifies dead-end metabolites that cannot be produced or consumed by any other reaction in the metabolic network. It checks the stoichiometric coefficients to determine if a metabolite is solely produced or solely consumed in a reaction. The function also detects metabolites involved in only one reaction, indicating a lack of support from other reactions in the network.

% Detection of dead-end metabolites:

DeadEnds\_close = closed\_model.mets(detectDeadEnds(closed\_model));

DeadEnds\_default = default\_model.mets(detectDeadEnds(default\_model));

DeadEnds\_open = open\_model.mets(detectDeadEnds(open\_model));

% Identification of associated reactions

[rxnList\_close, ~] = findRxnsFromMets(iPrub22\_model, DeadEnds\_close);

[rxnList\_default, ~] = findRxnsFromMets(iPrub22\_model, DeadEnds\_default);

[rxnList\_open, ~] = findRxnsFromMets(iPrub22\_model, DeadEnds\_open);

fprintf(['<strong>Number of dead-ends detected:</strong>\n Closed model: %d (%.1f%%)\n Default model: %d (%.1f%%)\n Open model: %d (%.1f%%)\n\n',...

'<strong>Number of associated reactions:</strong>\n Closed model: %d (%.1f%%)\n Default model: %d (%.1f%%)\n Open model: %d (%.1f%%)\n\n'],...

length(DeadEnds\_close),length(DeadEnds\_close)\*100/length(iPrub22\_model.mets),...

length(DeadEnds\_default),length(DeadEnds\_default)\*100/length(iPrub22\_model.mets),...

length(DeadEnds\_open),length(DeadEnds\_open)\*100/length(iPrub22\_model.mets),...

length(rxnList\_close),length(rxnList\_close)\*100/length(iPrub22\_model.rxns),...

length(rxnList\_default),length(rxnList\_default)\*100/length(iPrub22\_model.rxns),...

length(rxnList\_open),length(rxnList\_open)\*100/length(iPrub22\_model.rxns))

**Number of dead-ends detected:**
Closed model: 3411 (62.4%)
Default model: 3379 (61.8%)
Open model: 3336 (61.1%)
**Number of associated reactions:**
Closed model: 3173 (53.6%)
Default model: 3109 (52.5%)
Open model: 3023 (51.1%)

#### 2.2.3 Identify blocked reactions

The identifyBlockedRxns function detects reactions that are unable to generate flux within the constraints of the model. Blocked reactions provide information about the feasibility of the model. They highlight potential errors, connectivity issues, or gaps in our understanding of metabolism, as they cannot contribute to metabolic fluxes.

tic

BlockedReactions\_default = findBlockedReaction(default\_model);

BlockedReactions\_open = findBlockedReaction(open\_model);

toc

Elapsed time is 124.550720 seconds.

fprintf(['<strong>Number of blocked reactions:</strong>\n',...

' Default model: %d (percentage of active reactions: %.1f%%)\n',...

' Open model: %d (percentage of active reactions: %.1f%%)\n'],...

length(BlockedReactions\_default),100-length(BlockedReactions\_default)\*100/length(iPrub22\_model.rxns),...

length(BlockedReactions\_open),100-length(BlockedReactions\_open)\*100/length(iPrub22\_model.rxns))

**Number of blocked reactions:**
Default model: 4483 (percentage of active reactions: 24.3%)
Open model: 4252 (percentage of active reactions: 28.2%)

#### 2.2.4 Find leakage or siphons in the heuristically internal part using the bounds given by the model

Perform findMassLeaksAndSiphons function to determine whether molecular species can be generated from nothing (leak) or consumed without yielding anything (siphon) in the closed\_model (i.e. all exchange reactions are blocked). Entries in leakMetBool or siphonMetBool with non-zero values indicate that the associated molecular species can be generated or consumed independently without any requirement for additional input or output.

modelBoundsFlag = 0 ; % whith modelBoundsFlag == 0, the leak testing assumes that all internal reaction are reversible

[leakMetBool, leakRxnBool, siphonMetBool, siphonRxnBool, ~, ~, ~, ~] = findMassLeaksAndSiphons(closed\_model,true(length(closed\_model.mets),1),...

true(length(closed\_model.rxns),1),modelBoundsFlag);

Warning: optimizeCardinality: Maximum value of theta reached, at 12625.5841.

Warning: optimizeCardinality: Maximum value of theta reached, at 12625.5841.

Extract from the documentation of the findMassLeaksAndSiphons function:

OUTPUTS:

- leakRxnBool: `m` x 1 boolean of metabolites in a positive leakage mode
- leakRxnBool: `n` x 1 boolean of reactions exclusively involved in a positive leakage mode
- siphonMetBool: `m` x 1 boolean of metabolites in a negative leakage mode
- siphonRxnBool: `n` x 1 boolean of reactions exclusively involved in a negative leakage mode

% Results for metabolite leaks

leakMetBoolIndx = find(leakMetBool == 1) ;

for i=1:length(leakMetBoolIndx)

fprintf('<strong>Metabolite name:</strong> %s\n<strong>Id:</strong> %s\n<strong>Formula:</strong> %s\n<strong>Charge:</strong> %d\n\n',...

strjoin(iPrub22\_model.metNames(leakMetBoolIndx(i))), strjoin(iPrub22\_model.mets(leakMetBoolIndx(i))), strjoin(iPrub22\_model.metFormulas(leakMetBoolIndx(i))),...

iPrub22\_model.metCharges(leakMetBoolIndx(i))) ;

disp('---------------------------------------------------------------')

end

**Metabolite name:** [(1&rarr;4)-&beta;-D-glucosyl]n-1-(1&rarr;4)-D-glucono-1,5-lactone
**Id:** Cellulose-D-glucono-1-5-lactone[c]
**Formula:** C24H39O20
**Charge:** 0

---------------------------------------------------------------

**Metabolite name:** a [DNA]-3'-hydroxyl
**Id:** 3-Hydroxy-Terminated-DNAs[c]
**Formula:** C5H8O6P
**Charge:** -1

---------------------------------------------------------------

**Metabolite name:** a 5'-hydroxy-ribonucleotide-3'-[RNA]
**Id:** 5Prime-OH-Terminated-RNAs[c]
**Formula:** C5H8O7P
**Charge:** -1

---------------------------------------------------------------

**Metabolite name:** an [RNA]-3'-hydroxyl
**Id:** 3Prime-OH-Terminated-RNAs[c]
**Formula:** C5H8O7P
**Charge:** -1

---------------------------------------------------------------

**Metabolite name:** 5-deoxy-&alpha;-ribose 1-phosphate
**Id:** CPD-15199[c]
**Formula:** C5H9O7P
**Charge:** -2

---------------------------------------------------------------

**Metabolite name:** (3S,5R,6R)-3,5-dihydroxy-6,7-didehydro-5,6-dihydro-12'-apo-&beta;-caroten-12'-al
**Id:** CPD1F-4[c]
**Formula:** C25H34O3
**Charge:** 0

---------------------------------------------------------------

**Metabolite name:** a fructooligosaccharide
**Id:** Fructooligosaccharides[c]
**Formula:**
**Charge:** 0

---------------------------------------------------------------

**Metabolite name:** OH-
**Id:** OH[c]
**Formula:** H
**Charge:** -1

---------------------------------------------------------------

% Results for metabolite siphons

siphonMetBoolIndx = find(siphonMetBool == 1);

for i=1:length(siphonMetBoolIndx)

fprintf('<strong>Metabolite name:</strong> %s\n<strong>Id:</strong> %s\n<strong>Formula:</strong> %s\n<strong>Charge:</strong> %d\n\n',...

strjoin(iPrub22\_model.metNames(siphonMetBoolIndx(i))), strjoin(iPrub22\_model.mets(siphonMetBoolIndx(i))), strjoin(iPrub22\_model.metFormulas(siphonMetBoolIndx(i))),...

iPrub22\_model.metCharges(siphonMetBoolIndx(i))) ;

disp('---------------------------------------------------------------')

end

**Metabolite name:** [(1&rarr;4)-&beta;-D-glucosyl]n-1-(1&rarr;4)-D-glucono-1,5-lactone
**Id:** Cellulose-D-glucono-1-5-lactone[c]
**Formula:** C24H39O20
**Charge:** 0

---------------------------------------------------------------

**Metabolite name:** a [DNA]-3'-hydroxyl
**Id:** 3-Hydroxy-Terminated-DNAs[c]
**Formula:** C5H8O6P
**Charge:** -1

---------------------------------------------------------------

**Metabolite name:** a 5'-hydroxy-ribonucleotide-3'-[RNA]
**Id:** 5Prime-OH-Terminated-RNAs[c]
**Formula:** C5H8O7P
**Charge:** -1

---------------------------------------------------------------

**Metabolite name:** an [RNA]-3'-hydroxyl
**Id:** 3Prime-OH-Terminated-RNAs[c]
**Formula:** C5H8O7P
**Charge:** -1

---------------------------------------------------------------

**Metabolite name:** 5-deoxy-&alpha;-ribose 1-phosphate
**Id:** CPD-15199[c]
**Formula:** C5H9O7P
**Charge:** -2

---------------------------------------------------------------

**Metabolite name:** (3S,5R,6R)-3,5-dihydroxy-6,7-didehydro-5,6-dihydro-12'-apo-&beta;-caroten-12'-al
**Id:** CPD1F-4[c]
**Formula:** C25H34O3
**Charge:** 0

---------------------------------------------------------------

**Metabolite name:** a fructooligosaccharide
**Id:** Fructooligosaccharides[c]
**Formula:**
**Charge:** 0
[truncated: 49,334 more chars]
