# Supplementary material for: Reconciliation and evolution of Penicillium rubens genome-scale metabolic networks–What about specialised metabolism?
Source: PLoS One. 2023 Aug 30;18(8):e0289757. doi: 10.1371/journal.pone.0289757 (PMC10468094; doi:10.1371/journal.pone.0289757)
Supplement: Attachment — Submitted filename: Response to Reviewers.pdf [file pone.0289757.s010.pdf]

Revised Manuscript and Responses to Reviewers' Comments

To enhance readability and help reviewers easily identify corrections, we have formatted the reviewers' comments with a grey background, our responses are written in blue, and we use indentation to indicate corrected sentences from the manuscript.

Preamble

During the correction process, we obtained complementary results that led us to update some aspects of the manuscript. This yield to an improved model whose simulated growth possess improved sensitivity to nutrient variations. Below is a list of the modifications made:

- Updating and correcting all the numbers in the manuscript
- Addition of two figures containing the results of flux simulations - as asked by the reviewers - on growth (Fig 3) and the production of specific metabolites by *P. rubens* (Fig 6)
- Addition of a supplementary data file containing the bounds modifications carried out on the model and the results of the various simulations carried out.
- Modifications and reorganisation of the sections "Reconstruction transformation into a constrained model", Connectivity and metabolic coverage", and "Specialised metabolism through versions" (Results section)
- Reorganisation of the "From reconstruction to a model that simulates environmental exchanges" section (Discussion section)
- Update of "From reconstruction to models" and "Model functional capabilities: biomass and specialised metabolites production" (Material & Methods section)

Reviewer #1: Date: April 27, 2023

Title: Reconciliation and evolution of *Penicillium rubens* Genome-Scale Metabolic Networks – What about specialised metabolism?

Journal: PLOS ONE

In this paper "Reconciliation and evolution of *Penicillium rubens* Genome-Scale Metabolic Networks – What about specialised metabolism?", Nègre & Larhlimi *et al.* have mainly discussed in detail the reconstruction process, starting from draft reconstruction, manual curation, gap-filling and different versioning of GSMM (*i*Prub22) of *Penicillium rubens*, strain Wisconsin 54-1255 using various tools and sources. The whole paper is more focused on reconstruction process and providing community a new knowledge base. Although, authors have briefly given emphasis on specialized metabolism but then missed the opportunity to elaborate and demonstrate it with some case studies, by choosing target metabolites from the list that author have provided. This would have made the paper more useful and brought the novelty.

First of all, we would like to thank you for reviewing our manuscript, "Reconciliation and evolution of *Penicillium rubens* Genome-Scale Metabolic Networks – What about specialised metabolism?" We appreciate your thoughtful comments and the time you have taken to provide us with constructive feedback. We understand your point about the need to provide more emphasis on specialised metabolism and hope that the answers we have provided in this document will explain and clarify the choices we have made. Thus, we have addressed each of your comments and provided our responses below.

I am recommending this paper for major revision.

My suggestion for authors is to investigate specialized metabolism and select target metabolites, demonstrate it with 3-4 examples, show the simulation results, along with the complete metabolic pathways for the metabolites and validate your results with literature data if infrastructure for experimental validation is lacking.

While we understand the value of such an investigation, we believe that incorporating it in the current manuscript would significantly expand the scope and density of the work. However, to address the concern about specialised metabolites, we have included a column in our supplementary data (S3 file, formerly S4 file) indicating the topologically producible target metabolites. Furthermore, we have focused on ensuring the accurate simulation of growth prerequisites by the model, as demonstrated in Fig 3. Regarding specialised metabolite production, we have included some results in the manuscript (Fig 6). We acknowledge that these results need to be validated through experimental means, as suggested by the reviewer. We hope that by providing this foundation and indicating the producibility of specialised metabolites, our work lays the groundwork for future investigations and experimental validations.

Please find below some of the other minor comments below.

Abstract:

Line 15: remain silent in laboratory => you can use: are not exploited.

Line 18-19: … clever alternative? --> possible alternative

Line 22-27: In parallel ………specialized metabolites. Should be part of results section.

You have reconstructed the model which I agree is an important result itself, I am wondering you started the abstract discussing about the metabolites synthesized by biosynthetic gene clusters that have not been exploited which should have been the novelty of this study, and then you have given more stress on reconstruction process.

Thank you for your feedback on our abstract. Following your comments, we have restructured our abstract to make it more concise, accessible and, more accurate. We address each of your points in detail below.

First, we have changed "remain silent in laboratory" to " does not necessarily guarantee their expression under laboratory conditions" to emphasise the current challenges associated with exploiting these metabolites in the experimental field. For the second point, we agree that the word "clever" would be too impactful. We have therefore qualified this expression by " represents a promising approach".

Then, "In parallel, (1) an updated functional annotation of the *P. rubens* genome was carried out and supplemented by (2) an orthology search with different GSMNs templates. This first draft was enriched (3) by integrating data from *P. rubens* previous GSMN reconstructions and complemented (4) by manual curation steps targeting basal and specialised metabolites" may be confusing with results, whereas it represents the protocol we followed for the reconstruction. For the sake of clarity, we have reworded this sentence as follows: "Our reconstruction, *i*Prub22, adheres to current convention standards and quality criteria, incorporating updated functional annotations, orthology searches with different GSMN templates, data from previous reconstructions, and manual curation steps targeting basal and specialised metabolites."

Finally, given the major corrections that you propose to improve our manuscript, we understand your question concerning the beginning of our abstract. The exploitation of specialised metabolites of filamentous fungi is indeed the final objective and the reason for the proposal of our new reconstruction. Nevertheless, the reconstruction of a metabolic network and the analysis of the resulting constraint models are, from our point of view, two disciplinary fields that are certainly indissociable but also distinct. Although our manuscript provides preliminary insights into the presence and topological producibility and flux production of specialised metabolites, we have chosen to retain the concept of specialised metabolism of filamentous fungi as our catchphrase. We hope that the simplifications we made best reflect the content of the paper we are presenting, and we thank you again for the time you took to suggest improvements to the abstract of our manuscript.

> **Abstract**
> (Before) **Mining filamentous fungal genomes have revealed a high proportion of specialised metabolites in the past few years. However, many of these metabolites, produced by biosynthetic gene clusters, remain silent in the laboratory, and one hypothesis for soliciting their production lies in modifying their growth conditions. Though, it remains a time-consuming and costly process.** Therefore, the preliminary use of *in silico* modelling, such Genome-Scale Metabolic Network (GSMN), might be **a clever alternative** to explore and understand the production potential of a given organism. Consequently, *Penicillium rubens* Wisconsin 54-1255 strain was selected as the model organism. Following current convention standards and quality criteria, the proposed reconstruction results from the following four elements. **In parallel, (1) an updated functional annotation of the *P. rubens* genome was carried out and supplemented by (2) an orthology search with different GSMNs templates. This first draft was enriched (3) by integrating data from *P. rubens* previous**

> **GSMN reconstructions and complemented (4) by manual curation steps targeting basal and specialised metabolites.** The proposed high-quality GSMN, *i*Prub22, has a MEMOTE score of 68% and a metabolic coverage of 45%. The reconstruction is composed of 5,192 metabolites interconnected by 5,897 reactions, of which 5,033 are at least supported by a genomic sequence. *In fine*, the presence within *i*Prub22 of the specialised metabolisms highlights that many bottlenecks still exist. Solving such problems is the mandatory step to get access to specialised metabolic dedicated GSMN suitable for the exploration of metabolic regulation responsible for natural product synthesis.
> (After) **In recent years, genome sequencing of filamentous fungi has revealed a high proportion of specialised metabolites with growing pharmaceutical interest. However, detecting such metabolites through *in silico* genome analysis does not necessarily guarantee their expression under laboratory conditions. However, one plausible strategy for enabling their production lies in modifying the growth conditions. Devising a comprehensive experimental design testing in different culture environments is time-consuming and expensive. Therefore, using *in silico* modelling as a preliminary step, such as Genome-Scale Metabolic Network (GSMN), represents a promising approach to predicting and understanding the observed specialised metabolite production in a given organism. To address these questions, we reconstructed a new high-quality GSMN for the *Penicillium rubens* Wisconsin 54-1255 strain, a commonly used model organism. Our reconstruction, *i*Prub22, adheres to current convention standards and quality criteria, incorporating updated functional annotations, orthology searches with different GSMN templates, data from previous reconstructions, and manual curation steps targeting primary and specialised metabolites. With a MEMOTE score of 74% and a metabolic coverage of 45%, *i*Prub22 includes 5,192 unique metabolites interconnected by 5,919 reactions, of which 5,033 are supported by at least one genomic sequence. Of the metabolites present in *i*Prub22, 13% are categorised as belonging to specialised metabolism. While our high-quality GSMN provides a valuable resource for investigating known phenotypes expressed in *P. rubens*, our analysis identifies bottlenecks related, in particular, to the definition of what is a specialised metabolite, which requires consensus within the scientific community. It also points out the necessity of accessible, standardised and exhaustive databases of specialised metabolites. These questions must be addressed to fully unlock the potential of natural product production in *P. rubens* and other filamentous fungi. Our work represents a foundational step towards the objective of rationalising the production of natural products through GSMN modelling.**

Line 39,40,41: molecules – rather than use compounds/metabolites

We thank you for your attention to our manuscript to make it more rigorous in these definitions. The term "molecule" is generic, and the word "metabolites" refers directly to the products of metabolism. So, following your recommendations, we have changed two occurrences of the word "molecules" to "metabolites" to gain precision. However, we kept the first occurrence to avoid redundancy and underline, once again, that we are specifically interested in living organism products.

> **Lines 38 → 43**
> (Before) Natural **molecules**, synthesised by living organisms, can be categorised into two types based on their function within them: constitutive **molecules**, which are essential for the organism's growth, and specialised **molecules**, which provide competitive advantages towards the organism's environment, historically referred to as secondary metabolites.
> (After) Natural **molecules**, synthesised by living organisms, can be categorised into two types based on their function within them: constitutive **metabolites**, which are essential for the organism's growth, and specialised **metabolites**, which provide competitive advantages towards the organism's environment, historically referred to as secondary metabolites.

Line 49: relaunch --> study or investigate.

Thank you for your suggestion of simplification. However, with this sentence, we aim to emphasise the need to revitalise a research area that is losing momentum. To clarify our thinking, we have slightly modified our sentence by adding some contextual words and replacing the verb "relaunch" with "reinvigorate".

> **Lines 48 → 50**

> (Before) Given the increasing antibiotic resistance [3], it is essential **to relaunch** such NP research using more rational and predictive approaches.
> (After) Given the increasing antibiotic resistance [3], it is essential **to reinvigorate research on NPs, which has faltered in recent years,** using more rational and predictive approaches.

Line 84-85: properties of metabolism? organisms can be described… --> metabolic properties of an organisms can be described…

We thank you for your careful reading. We have followed your recommendations for the following two comments.

> **Lines 84 ➝ 86**
> (Before) In systems biology, the biochemical and physiological **properties of metabolism organisms** can be described by Genome-Scale Metabolic Networks (GSMNs) studies [15].
> (After) In systems biology, **an organism's biochemical and physiological metabolic properties** can be described by Genome-Scale Metabolic Networks (GSMNs) studies [15].

Line 91-92: enhancing our metabolism understanding at the system level [17]. --> enhancing our understanding of metabolism at the system level [17].

> **Lines 90 ➝ 92**
> (Before) Moreover, increasing model complexity aims to integrate and visualise heterogeneous knowledge-related metabolism more and more efficiently [16], thereby **enhancing our metabolism understanding** at the system level [17].
> (After) Moreover, increasing model complexity aims to integrate and visualise heterogeneous knowledge-related metabolism more and more efficiently [16], thereby **enhancing our understanding of metabolism** at the system level [17].

Line 96: whose non-exhaustive? list is presented…

With this comment, we understand that our sentence is not clear and that we do not convey the desired idea. For sake of clarity, we reversed the order of the propositions in this sentence to emphasise that, apart from the networks of model organisms (which are the result of multi-collaborative work over the years), there is a lack of this type of work on other organisms.

> **Lines 95 ➝ 97**
> (Before) **Apart from well-studied model organisms, whose non-exhaustive list is presented in Gu *et al.* [19], few examples of individual network reconciliation exist [20,21].**
> (After) **Unfortunately, only a few examples of individual network reconciliation are reported [19,20], aside from the well-studied model organisms presented in a non-exhaustive list by Gu *et al.* [21], which are exceptions.**

Line 104: (e.g., knowledge snapshot)?

Thank you for bringing this to our attention. By "knowledge snapshot," we mean a specific moment in time when the reconstruction of the metabolic network was created, which represents the best understanding of the organism's metabolism based on the available knowledge and data at that time. We have revised the sentence to clarify this point.

> **Lines 102 ➝ 104**
> (Before) Moreover, a GSMN represents a platform of organised and summarised resources intended to reflect the optimal metabolic capabilities of the target organism at the reconstruction time (*e.g.* **knowledge snapshot**).
> (After) Moreover, a GSMN represents a platform of organised and summarised resources intended to reflect the optimal metabolic capabilities of the target organism at **the time of reconstruction based on the available knowledge and data at that moment (*e.g.* knowledge snapshot).**

Line 106: pressing --> important.

We acknowledge that the term "pressing" might suggest a stronger sense of urgency than intended, and we appreciate your suggestion to use the word "important" instead. However, we have decided to keep the original wording as we believe it effectively conveys the urgency and criticality of maintaining up-to-date and standardised models, particularly given the constant and rapid evolution of data. We hope that this clarifies our reasoning behind the choice of language in the manuscript.

> **Lines 104 → 106**
> In this respect, given the constant and rapid evolution of data, the need to maintain these models up-to-date and standardised is increasingly becoming **pressing**.

Line 130-131: Fig 1 in S2 file Veen --> Venn

Thank you for your careful review of our additional data. We have corrected the grammatical error concerning the word "Venn" in the caption of Figure S1 in the S2 file.

Line 133-134: compared to the only benefit? of Trinotate

Thank you for pointing out the lack of clarity in our wording. We hope that the rephrasing will make it more readable.

> **Lines 132 → 134**
> (Before) Thus, the joint use of KAAS [30] and gene information stored in KEGG allows an 11% enrichment in EC numbers **compared to the only benefit of Trinotate [31] results**.
> (After) Thus, the **combined** use of KAAS [30] and gene information stored in KEGG **leads to** an 11% enrichment in EC numbers **compared to relying solely on Trinotate** [31] results

Line 157-158: Fig 4 A-B in the S2 file --> center align x-axis title. The Fig 4 A can be made better.

Fig 9, 10,11 in the S2 file, center align x-axis & y-axis title. Fig 8 in the S2 file, center align x-axis.

Regarding the figures formatting in the S2 file (rename in S1 file), we have made the necessary adjustments, including centring the axis captions where appropriate and standardising the font sizes for consistency. Additionally, we have followed your suggestion to rework Figure S4 into three panels, which has improved its readability. Finally, we have corrected the order of figures S7, S8, S9, and S10 to match the manuscript's original sequence.

Line 253: searchable model?

Thank you for your comment. We agree that our vision of reconstruction needs clarification. As we see it, the usefulness of a reconstruction goes beyond the predictive power of the resulting model. For instance, it can also provide valuable information for researchers studying the organism, such as identifying the genomic sequences associated with each enzyme through GPR associations. Therefore, we have added an emphasis to define our concept of a "searchable model" (a knowledge platform comprising accessible resources for a given organism at the instance of reconstruction).

> **Lines 252 → 253**
> (Before) Once the initial draft is produced, several modifications must be performed to obtain a functional and **searchable model** compatible with the experimental observations.
> (After) Once the initial draft is produced, several modifications must be performed to obtain a functional and **searchable model** compatible with the experimental observations **(*i.e.* a knowledge platform comprising a set of accessible resources for a given organism at the instance of reconstruction).**

Line 255: These Target compounds presence in the …. --> These Target compounds/metabolites present in the …

Thank you for bringing to our attention the lack of clarity in our sentence. We have revised it to clarify the two distinct logical aspects. While it is crucial for the target compounds to be present in the network and topologically producible, these factors alone are not sufficient for conducting flux studies but rather constitute a starting point. Therefore, these two points are essential and they have been explored in our reconstruction.

> **Lines 255 ➝ 256**
> (Before) **These target compounds' presence in the draft** and their topological producibility were then investigated.
> (After) **Initially, we investigated the presence of these target compounds in the draft and subsequently explored their topological producibility.**

Line 260: Title: Targets selection --> Target selection

Thank you for bringing the grammatical error in our title to our attention. We have taken your feedback into account and corrected it.

Line 279: a list of 47 compounds lacking…. S4 file sheet Orphans metabolites --> 46 metabolites, header is not a metabolite.

We appreciate the thoroughness with which you reviewed our additional data and we have corrected the incorrect data in Table 1. We correct this error and ensure the accuracy of our findings.

> **Line 279**
> (Before) During this process search, a list of **47** compounds lacking MetaCyc identifiers was also generated (*i.e.* orphan metabolites).
> (After) During this process search, a list of **46** compounds lacking MetaCyc identifiers was also generated (*i.e.* orphan metabolites).

Line 284-285: targets1 … 243 targets --> 237 mentioned in S4 file sheet Targets1. Check discrepancy between table S4 & Table 1

Line 296: Targets2 …35 --> 47 mentioned in S4 file sheet Targets2. Check discrepancy between table S4 & Table 1.

To clarify the points mentioned, we have included a README sheet at the beginning of our Excel workbook titled "S3_file.xlsx" (former S4 file) which provides detailed information on the selection of targets. Here are the specifics for the Targets1 and Targets2 sheets:

> The **"Targets1"** sheet contains the first set of target metabolites, which were selected based on information from the literature. These target metabolites belong indifferently to constitutive or specialised metabolism. The selection process involved two noteworthy publications: one that enabled accurate modelling of the sugar pathway in fungi (Aguilar-Pontes *et al.*) and another that resulted from manual curation of the penicillin biosynthesis pathway (Prauße *et al.*). The list of targets was complemented by metabolites associated with biomass function extracted from *i*AL1006 (Agren *et al.*) and information extracted from the SMASH tool suite. Please note that, out of the 237 identified metabolites presented, Xanthocillin and Pr-toxin have MetaCyc identifiers but are not associated with any reactions. Hence, within *i*Prub22, only 235 compounds with a MetaCyc Id are potentially exploitable. Finally, to model the biomass reaction, we have included eight homemade identifiers from *i*AL1006: AAPOOL, CELLWALL, COF, DNA, RNA, PROTEIN, PLIPIDS, and Biomass produced by reactions r1459, r1455, r1465, r1458, r1457, r1456, r1460, and Biomass_rxn, respectively. These identifiers represent artificial compounds that encompass a combination of metabolites. Although they are not listed in the Targets1 sheet, we have included them in our count of 243 targets because their producibility is crucial for ensuring network functionality.

> The **"Targets2"** sheet consists of metabolites obtained by querying the LOTUS database, a specific Natural Product database. Out of the 240 metabolites associated with *P. chrysogenum/P. rubens* in this database, only 47 are found in the MetaCyc database on the basis of a complete or incomplete InChIKey match. As we decided to retain only those metabolites that are known to be present in Wisconsin strain 54-1255, 11 of these compounds are excluded from our search set. Finally, as the patulin biosynthetic pathway is incomplete in *P. rubens*, patulin is also removed from our target set. However, these 12 metabolites are still presented in this Excel sheet to highlight the importance of being cautious and careful when selecting data. In the end, the Targets2 sheet includes 35 metabolites.

Align the titles for x-axis and y-axis for supp. And main figures in the paper to center

As previously stated, we have considered the feedback regarding the formatting of the figures included in the S2 file, and we appreciate your input. Nevertheless, since we did not observe any misaligned components in the main figures, we have left them unchanged.

add readme for tables in s3 file.

Thank you for recommending the inclusion of README for our additional data. We appreciate your input and want to let you know that we have followed your advice. Specifically, we have added a separate sheet to each of our Excel workbooks (S3 to S6 files) that provides a detailed explanation of the contents. We hope that the README inclusion will strengthen our work and greatly facilitate the understanding and interpretation of our data, allowing for better reproducibility and further analysis. Once again, we appreciate your suggestion and the opportunity to improve our work.

In conclusion, we appreciate the time and effort you invested in reviewing our manuscript and providing us with valuable feedback. While we understand and respect your suggestions for major revisions, we have decided to maintain the current direction of the manuscript. However, we want to assure you that we have taken your comments on the form of the manuscript and the figures seriously and have made the necessary adjustments to improve their clarity and readability. Your attention to detail has helped us enhance our work presentation, and we are grateful for your constructive feedback.

Reviewer #2:

This manuscript introduces a new semi-automatically built and manually curated genome-scale metabolic model for the organism *P. rubens* strain Wisconsin 54-1255 based on a newly annotated genome. The model is comparably large and follows common standards. The result is a well-standardised and reusable model with great potential for other researchers in systems biology. The authors should only improve a few aspects before publication.

We express our sincere appreciation for your careful review of our manuscript and thank you for the valuable comments you provided. Furthermore, we are grateful for your recognition of our efforts to propose a manually curated genome-scale metabolic model for *P. rubens* strain Wisconsin 54-1255 that follows common standards. Your comments have provided us with valuable insights, and they have helped us identify the areas that require improvement before publication. We are pleased to read that you find our new genome-scale metabolic model for *P. rubens* strain Wisconsin 54-1255 to be well-standardized and with great potential for other researchers in systems biology. Thank you for your time and valuable insights. Below, you will find our responses to the comments you provided.

1) Major Points

==========

<u>The SBML model</u>

The model is entirely valid, and its MEMOTE score is quite sound. Many sub-categories even reach the maximum of 100%. However, the 68% still have room for improvement. In particular, links to the BiGG Models database are scarce. Other works use the tool ModelPolisher (https://github.com/draeger-lab/ModelPolisher) to increase the model score. The authors could give it a try.

We thank you for underlining the reconstruction quality score and suggesting ways to improve it. We greatly appreciate the opportunity of being able to develop here some technical points that are not often mentioned in the manuscript. We hope that the following information will meet your concerns.

Firstly, we would like to explain the limited annotations of metabolites and reactions from the BiGG database in our reconstruction. Our mapping strategy, aimed at augmenting the reconstruction with annotations, relies on data from MetaNetX. Specifically, from the MetaCyc identifiers (our reference database for the reconstruction), we started to search for all the MetaNetX IDs. Subsequently, from these MetaNetX IDs, we retrieved all associated identifiers from other databases, including those from BiGG. The Venn diagram below represents all metabolites (orange) and reactions (purple) from the BiGG and MetaCyc databases that have a MetaNetX ID in version 4.1. The intersection shows that the entities sharing a MetaNetX ID represent a small proportion of the overall data. Consequently, the limited representation of BiGG IDs in our reconstruction reflects the extent of communication between MetaCyc and BiGG through the MetaNetX IDs at the time of reconstruction.

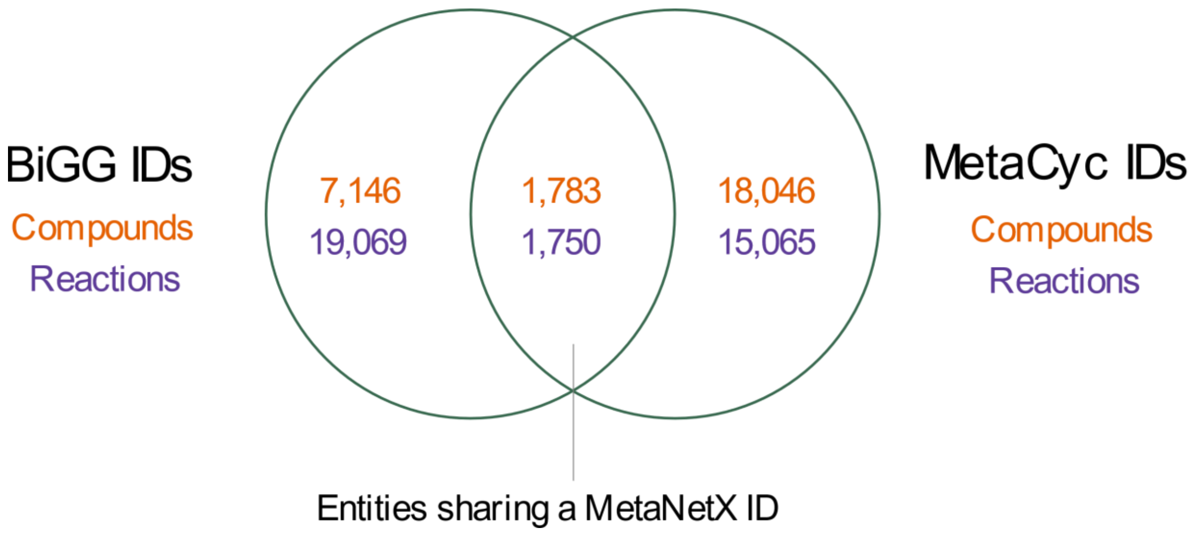

Thank you for suggesting ModelPolisher for improving annotation interoperability and enriching our model by identifying BiGG. We had considered this possibility, but knowing that our anchor for annotation enrichment is the MetaCyc annotations, this tool did not seem appropriate for our needs. Indeed, the description of this tool on https://github.com/draeger-lab/ModelPolisher indicates that "ModelPolisher accesses the BiGG Models knowledgebase to annotate and autocomplete SBML models. Thereby, the program mainly relies on BiGG identifiers for model components". Thus ModelPolisher allows reconstruction to be enriched from pre-existing BiGG identifiers in the reconstruction model, which is not our case.

Now, regarding the MEMOTE score, we fully agree that there is still room for improvement. That is why we are providing an actual screenshot below detailing its calculation (*i.e.* during the correction process, improvements were made to the model and are described at the beginning of this document). We have observed that improving the BiGG annotations would have had a minimal impact on the overall score (although we fully acknowledge that, in reality, their importance is crucial for widespread reconstruction use). Therefore, during the reconstruction, we have chosen to focus on gene and SBO term annotations since, after the mapping process, our annotations for reactions and metabolites were above 80% and seemed satisfactory to us.

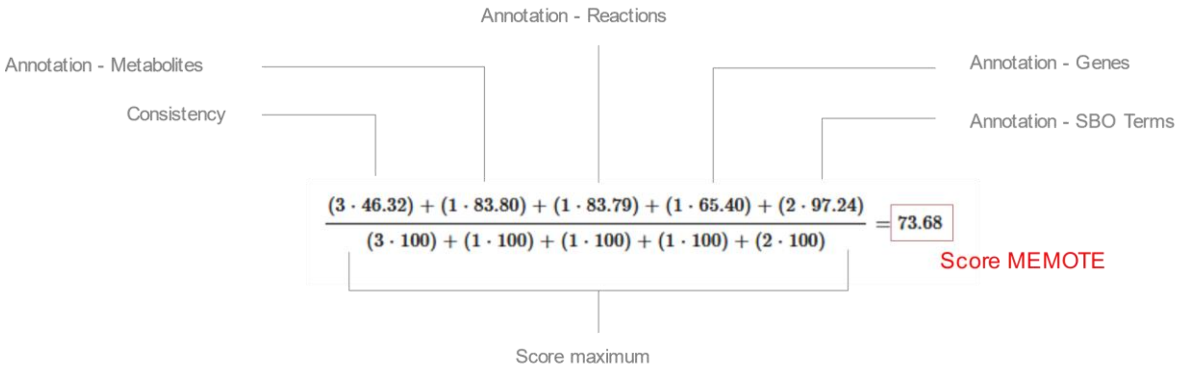

Moreover, without delving into the details of the MEMOTE score calculation, we would like to take advantage of your comment to address some clarifications regarding the results displayed in the report's different sections.

- **Consistency**
    - Stoichiometric Consistency 0%

Stoichiometric consistency is assessed through a binary scoring approach, where a score of 0 is assigned as soon as a metabolite is detected in either the unconserved metabolites or inconsistent minimal net stoichiometry section. As we have not curated the data extracted from MetaCyc, the information presented in the "Consistency" section accurately reflects the inherent characteristics of the elements derived from this database.

- **Annotation - Metabolites**
    - Uniform Metabolite Identifier Namespace 84.4%

The report specifies that "852 metabolite identifiers (15.59%) deviate from the largest found namespace (BioCyc)". After removing the compartment information from these identifiers, 720 unique metabolites remain, of which 645 map to MetaCyc. The last 75 are either id from the biomass function, compounds added from iAL006 or obsolete terms.

- **Annotation - Reactions**
    - Uniform Reaction Identifier Namespace 87.5%

The report specifies that "758 reaction identifiers (12.81%) deviate from the largest found namespace (BioCyc)". These identified reactions pertain to the modelling reactions (noted Uptake_\d{3}, Production_\d{3}, Transport_\d{3}, Demand\d{3}, Sink\d{3}) or to reactions coming from *i*AL1006 (r\d{4}).

- **Annotation - Genes**
    - Total Genes 6,171 and Gene Annotations Per Database at 92,4%.

In our reconstruction, to facilitate the exploration of the network, we have associated artificial genes with the modelling and spontaneous reactions, in particular, to differentiate them from the gap-filling reactions. More precisely, *P. rubens* genes follow the pattern Pc\d{2}\g\d{5}, while artificial genes are denoted as [s|u|p|d|t|sk]\d{3} (representing spontaneous, uptake, production, demand, transport and sink). As a result, the total number of genes is overestimated, leading to underestimated percentages of gene annotation presence in each database.

- **Annotation – SBO Terms**
    - Metabolic Reaction SBO:0000176

The report states that "A total of 91 metabolic reactions (1.70% of all purely metabolic reactions) lack annotation". These 91 reactions correspond to transport reactions, for which we have chosen to associate more specific SBO terms representing their respective categories.

- Transport Reaction SBO:0000185

The report specifies that "A total of 36 metabolic reactions (11.32% of all transport reactions) lack annotation". These 36 reactions refer to demand reactions (Demand_\d{3}), which are associated with their corresponding SBO term.

- Demand Reaction SBO:0000628

The report specifies that "A total of 4 reactions (9.76% of all demand reactions)". These 4 reactions mentioned are associated with the 'biochemical reaction' SBO term (SBO:0000176).

- Gene SBO:0000243

The report specifies that "A total of 468 genes (7.58% of all genes) lack annotation". These 468 genes correspond to the artificial genes mentioned above and are associated with an SBO linked to an artificial entity ("SBO:0000291: not monitored by MEMOTE).

We hope that this information will provide sufficient and necessary insight to understand both the reconstruction processes we have chosen and their consequential impact on the MEMOTE score. Once again, we would like to emphasise that we are presenting a reconstruction which provides a default model which requires further refinement (as indicated by the specific results of the MEMOTE report).

Also, the authors should upload the model to the BioModels database instead of providing it as a supplement for multiple reasons:

1. It will make their model more easily findable because modellers can search for models in one central place and find the model with the link to this publication.

2. Model revisions can be created, which is different for supplements. In particular, if, despite careful reconstruction, inaccuracies are found in the model later on, this versioning will be beneficial.

3. The BioModels team has professional curators who permanently update and improve models and ensure their reusability.

When doing so, it would be ideal if the authors could include a FROG analysis in their model and wrap it in an OMEX file (see https://www.ebi.ac.uk/biomodels/curation/fbc for details).

We thank you for highlighting the benefits of uploading our model to the BioModels database. We believe that this step will significantly enhance the dissemination and long-term accessibility of our work. We appreciate your insights, and we agree that there are several advantages to making the model more easily accessible through a central repository. Consequently, we are pleased to inform you that *i*Prub22 was registered in BioModels and given the identifier MODEL2306150001. In line with your suggestion, we will include a FROG analysis in our model and wrap it in an OMEX file. It facilitates the integration of our model with the BioModels database and ensures compliance with the guidelines provided on their website (https://www.ebi.ac.uk/biomodels/curation/fbc).

As a minor note: The model's annotation should link the NCBI taxon identifier using the hasTaxon qualifier instead of "is".

We greatly appreciate you taking the time to review in detail the model provided. As a result of your feedback, we have made the necessary modifications to the SBML file.

```
    <annotation>
      <rdf:RDF xmlns:rdf="http://www.w3.org/1999/02/22-rdf-syntax-ns#" xmlns:dcterms="http://purl.org/dc/terms/"
xmlns:vCard="http://www.w3.org/2001/vcard-rdf/3.0#" xmlns:vCard4="http://www.w3.org/2006/vcard/ns#" xmlns:bqbiol=
"http://biomodels.net/biology-qualifiers/" xmlns:bqmodel="http://biomodels.net/model-qualifiers/">
          <rdf:Description rdf:about="#meta_iPrub22_v1">
            <bqbiol:hasTaxon>
                <rdf:Bag>
                <rdf:li rdf:resource="https://identifiers.org/taxonomy/500485"/>
              </rdf:Bag>
            </bqbiol:hasTaxon>
            <bqbiol:is>
              <rdf:Bag>
                <rdf:li rdf:resource="https://identifiers.org/insdc.gca/GCA_000226395.1"/>
              </rdf:Bag>
            </bqbiol:is>
          </rdf:Description>
        </rdf:RDF>
    </annotation>
```

2) Minor Points

==========

References

We thank you for taking the time to provide us with timely suggestions for including additional missing references in our manuscript. We have made the necessary additions and changes, as per your recommendations, which are listed below. Then, to help the reader understand the concept of ontology, we have added to each mention of the term SBO, the article you suggest. Finally, we have updated the reference concerning the BiGG database. Thus, your feedback helps to ensure that our work is up-to-date and accurately reflects the most recent advances in the field.

* Please support the development of SBML by citing the specific SBML specification documents in addition to the review by Keating *et al.* (2020). In this case, references are necessary to the specification for SBML Level 3 Version 1 (https://doi.org/10.1515/jib-2017-0080) and the FBC package version 2 (https://doi.org/10.1515/jib-2017-0082).

> **Line 784**
> […] models that preferentially respond to the SBML3 FBC2 format **[59,74]**
>
> **Line 530-531**
> […] by upgrading the network distribution format to comply with SBML Level 3 **[59]**.
>
> **Line 1262**
> To comply with these requirements (SBML Level 3, FBC2 **[59,74]**) […]
>
> **Line 1271**
> The conversion to FBC2 **[74]** was performed […]

* The paper by Courtot *et al.* (2011) is a reference that describes all ontologies with relevance for SBML in one overview (see https://doi.org/10.1038/msb.2011.77) and is typically cited to refer to SBO and so forth.

> **Line 518**
> SBO annotations provide semantic information that characterises the model components [55,**56**].
>
> **Line 750**
> […] the annotation of the model entities in terms derived from the 'Systems Biology Ontology' (SBO) [55,**56**]

> **Line 1269**
> Each entity of the network was also associated with an SBO term [55,**56**]

* The newer article https://doi.org/10.1093/nar/gkz1054 supersedes reference number 100 and should be cited instead.

> **Line 1167**
> As data templates for orthology refer to other databases like KEGG [29] or BiGG **[103]** […]

<u>Style and other remarks</u>

* Please avoid starting sentences with lowercase letters, as this is done in line 121.

We want to thank you for your careful reading of our manuscript. Indeed, as the model name *i*Prub22 begins with a lowercase letter, we have reversed the order of the words on the line you mention, and lines 458 and 1101 are modified to gain precision.

> **Line 121**
> (Before) ***i*Prub22** GSMN reconstruction started with the automatic generation of two drafts generated using genome functional annotation and homology searches with preexisting models.
> (After) **The GSMN reconstruction *i*Prub22** started with the automatic generation of two drafts generated using genome functional annotation and homology searches with pre-existing models.
>
> **Line 458**
> (Before) ***i*Prub22** had InChIKeys identifiers for […]
> (After) **The *i*Prub22 reconstruction** had InChIKeys identifiers for […]
>
> **Line 1101**
> (Before) ***i*Prub22** is provided in the SBML community standard format […]
> (After) **The *i*Prub22 reconstruction** is provided in the SBML community standard format […]

* It is a custom that the words "Level" and "Version" are written with uppercase letters in conjunction with SBML rather than in lowercase.

Your attention to detail has helped us improve the accuracy and precision of our work. Following your feedback, we have made corrections to the case of the words "Version" and "Level" on lines 531, 789, 794, and 1262 to ensure consistency throughout the manuscript.

> **Line 530-531**
> (Before) […] by upgrading the network distribution format to comply with SBML **level 3**.
> (After) […] by upgrading the network distribution format to comply with SBML **Level 3 [59]**.
>
> **Line 789**
> (Before) […] as this notation was only introduced in **version 2** of the FBC package.
> (After) […] as this notation was only introduced in **Version 2** of the FBC package.
>
> **Line 794**
> (Before) Thus, the proposed model was standardised in the most recent **versions** of SBML […]
> (After) Thus, the proposed model was standardised in the most recent **Versions** of SBML […]
>
> **Line 1262**
> (Before) To comply with these requirements (SBML **level 3**, FBC2) […]
> (After) To comply with these requirements (SBML **Level 3**, FBC2 [59,74]) […]

* The COMBINE website is now reachable *via* HTTPS from https://co.mbine.org (no longer just HTTP).

Thank you for pointing out the inaccuracy with our hyperlink. The correction you suggested has made it possible for us to provide a more accurate and up-to-date link.

> **Line 1263-1264**
> (Before) […] on the COMBINE resource (**http:**//co.mbine.org […]
> (After) […] on the COMBINE resource (**https:**//co.mbine.org […]

Once again, we want to express our sincere gratitude for your thoughtful and insightful comments. Your comments have helped us to gain rigour, and to emphasise the importance of spreading our model within the community. Above all, they enabled us to pinpoint and find the aspects that had previously prevented us from obtaining a fully functional model. Thank you once again for your valuable contributions.

---

6. PLOS authors have the option to publish the peer review history of their article (what does this mean?). If published, this will include your full peer review and any attached files.

If you choose "no", your identity will remain anonymous but your review may still be made public.

Do you want your identity to be public for this peer review? For information about this choice, including consent withdrawal, please see our Privacy Policy.

Reviewer #1: Yes: Ab Rauf Shah

Reviewer #2: No
