# Supplementary material for: Reconciliation and evolution of Penicillium rubens genome-scale metabolic networks–What about specialised metabolism?
Source: PLoS One. 2023 Aug 30;18(8):e0289757. doi: 10.1371/journal.pone.0289757 (PMC10468094; doi:10.1371/journal.pone.0289757)
Supplement: Attachment — Submitted filename: ReviewReport_Negre_et-al_04_23.docx [file pone.0289757.s009.docx]

Date: April 27, 2023

Title: ***Reconciliation and evolution of Penicillium rubens Genome-Scale Metabolic Networks – What about specialised metabolism?***

Journal: PLOS ONE

In this paper “***Reconciliation and evolution of Penicillium rubens Genome-Scale Metabolic Networks – What about specialised metabolism?***”, Nègre & Larhlimi et al. have mainly discussed in detail the reconstruction process, starting from draft reconstruction, manual curation, gap-filling and different versioning of GSMM (*i*Prub22) of *Penicillium rubens*, strain Wisconsin 54-1255 using various tools and sources. The whole paper is more focused on reconstruction process and providing community a new knowledge base. Although, authors have briefly given emphasis on specialized metabolism but then missed the opportunity to elaborate and demonstrate it with some case studies, by choosing target metabolites from the list that author have provided. This would have made the paper more useful and brought the novelty.

***I am recommending this paper for major revision***.

My suggestion for authors is to investigate specialized metabolism and select target metabolites, demonstrate it with 3-4 examples, show the simulation results, along with the complete metabolic pathways for the metabolites and validate your results with literature data if infrastructure for experimental validation is lacking.

Please find below some of the other minor comments below.

**Abstract:**

Line 15: remain silent in laboratory => you can use: are not exploited.

Line 18-19: … clever alternative? 🡪 possible alternative

Line 22-27: *In parallel ………specialized metabolites*. Should be part of results section.

You have reconstructed the model which I agree is an important result itself, I am wondering you started the abstract discussing about the metabolites synthesized by biosynthetic gene clusters that have not been exploited which should have been the novelty of this study, and then you have given more stress on reconstruction process.

Line 39,40,41: *molecules* – rather than use compounds/metabolites

Line 49: *relaunch* 🡪 study or investigate.

Line 84-85: *properties of metabolism? organisms can be described…* 🡪 metabolic properties of an organisms can be described…

Line 91-92: *enhancing our metabolism understanding at the system level* [17]. 🡪 enhancing our understanding of metabolism at the system level [17].

Line 96: *whose non-exhaustive? list is presented…*

Line 104: *(e.g., knowledge snapshot)?*

Line 106: *pressing* 🡪 important.

Line 130-131: Fig 1 in S2 file *Veen* 🡪 Venn

Line 133-134: *compared to the only benefit? of Trinotate*

Line 157-158: Fig 4 A-B in the S2 file 🡪 center align x-axis title. The Fig 4 A can be made better.

Fig 9, 10,11 in the S2 file, center align x-axis & y-axis title. Fig 8 in the S2 file, center align x-axis.

Line 253: searchable model?

Line 255: *These Target compounds presence in the* …. 🡪 These Target compounds/metabolites present in the …

Line 260: Title: *Targets* *selection* 🡪 Target selection

Line 279: a list of 47 compounds lacking…. S4 file sheet *Orphans metabolites 🡪 46 metabolites, header is not a metabolite.*

Line 284-285: targets1 … 243 targets 🡪 237 mentioned in S4 file sheet *Targets1*. Check discrepancy between table S4 & Table 1

Line 296: Targets2 …35 🡪 47 mentioned in S4 file sheet *Targets2.* Check discrepancy between table S4 & Table 1.

Align the titles for x-axis and y-axis for supp. And main figures in the paper to center

add readme for tables in s3 file.
